# Supplementary material for: Cost-effectiveness of end-game strategies against sleeping sickness across the Democratic Republic of Congo
Source: PLoS Negl Trop Dis. 2026 Oct 22;20(10):e0014777. doi: 10.1371/journal.pntd.0014777 (PMC13719019; doi:10.1371/journal.pntd.0014777)
Supplement: Supplementary file 1 [file pntd.0014777.s001.pdf]

Supplementary Materials
Cost-effectiveness of end-game strategies against sleeping sickness across the Democratic Republic of Congo

Supplementary Methods

Table of Contents

S1.1 Key updates to the modelling in this study . . . 2
S1.2 New data extraction . . . 4
S1.2.1 Data . . . 4
S1.2.2 Post-processing of data in Bas-Uélé region health zones. . . . 7
S1.3 Locations . . . 7
S1.4 Model formulation . . . 12
S1.4.1 Assumptions about past interventions . . . 13
S1.5 Model fitting . . . 19
S1.6 Projections . . . 22
S1.7 Strategies and interventions . . . 24
S1.7.1 Interventions . . . 24
S1.7.2 Strategies . . . 28
S1.8 Cost-effectiveness analysis . . . 30
S1.9 Health outcomes denominated as disability-adjusted life-years . . . 33
S1.10 Treatment outcomes and cost functions . . . 34
S1.10.1 Active screening . . . 34
S1.10.2 Passive screening . . . 36
S1.10.3 Vector control . . . 38
S1.10.4 Treatment . . . 40
S1.10.5 Treatment and deaths in the pre-2026 period . . . 44
S1.11 Sensitivity analysis . . . 50
References for S1 Text . . . 51

S1.1 Key updates to the modelling in this study

↩ Return to the Supplement Table of Contents

Data extraction

We now have four more years of data (covering 2000–2020) compared to previous modelling studies for the DRC (2000–2016) [1, 2]. In the current data extraction, we have more places which have "unknown" active screening (AS) numbers. This occurs where the apparent prevalence in AS was >10% or the recorded number screened was <20. In previous studies [1, 2], we assumed that these new cases arose from people being recorded in their home health zone, although they were screened in a neighbouring health zone. We still believe this assumption to be appropriate, but we set the number of people screened in those "home health zones" to be "missing", which allowed us to impute the number of people that would have to be tested in that health zone, given the current prevalence, to generate the number of new active cases that was recorded. Data extraction is further described in the Supplementary Methods, Section S1.2.

The Bas-Uélé region of Isangi coordination comprises the health zones of Ango, Bili, Doruma, Ganga, Poko and Titule. Of these, only Ango and Doruma had sufficient data points in the final data set to be analysed. Médecins Sans Frontières (MSF) were active in the Bas-Uélé region from 2008–2014. Some modifications were made to the aggregated data in this region:

1. Based on information from an MSF report [3], data from Ango and Ganga in 2014 were modified to include only confirmed cases, with an assumed specificity of 100%.
2. Implausibly high numbers of cases were detected from passive screening in Ango (in 2008) and Ganga (in 2010 and 2011), we assumed that these arose from widespread screening by MSF within the health centres (effectively active screening) and consequently transferred them to the active screening data. This also required the number of people screened in those HZ–year combinations to be estimated within the model fitting process.
3. In early 2024, exploratory screening was performed in the Ango health zone. Information received from this screening indicated that no staging had taken place as part of MSF's activities. In light of this, staging information was removed from the data in the Bas-Uélé region.

Fitting assumptions and process

4. **Endemic Equilibrium.** We now assume no activity (and in particular no PS improvement) before the first detection year in all health zones where the first detection is after 2000. In all other health zones with detections in 2000, we assume some step improvement to PS in 1998 in line with our previous analyses [1, 2].
    4.1. In Kasaï Occidental and Kasaï Oriental coordination, the model assumed transmission remained at endemic equilibrium until the year when cases were first reported. This was because of a lack of accessibility preventing interventions from taking place in the early 2000s.
    4.2. In the Bas-Uélé region of Isangi coordination, passive screening (PS) began in 2004. As this region is difficult to access and hence no interventions were taking place in the early 2000s, we assumed that transmission was in equilibrium until 2004 instead of 1998.
5. **Specificity in AS.** Starting from the second published "Warwick gHAT model" [4] it was assumed that AS diagnostic specificity would increase to 100% due to additional cross-checking of confirmed cases at very low prevalence. A somewhat arbitrary threshold "fewer than 2 reported cases per 10,000 above the expected incidence of false positives based on the level of screening" was used in previous analyses if it was not clear when this might happen. More recently, the rollout of video confirmation, starting in Bandundu Nord and Sud coordinations, has provided a mechanism for this to happen. In this present study, we therefore assume fixed years when specificity would increase to 100% rather than using the previous threshold; this is 2015 in Yasa Bonga and Mosango health zones, 2018 for the remainder of Bandundu Nord and Sud coordinations, 2018 for Kongo Central and Kinshasa coordinations, and 2026 elsewhere.
    5.1. In previous modelling studies in the DRC [1, 2], we assumed that MSF were active in the health zones in the Bas-Uélé region (including in Ango and Doruma) of Isangi coordination from 2008 until 2012, and the PNLTHA was active for two years in 2013 and 2014. As a result of updated information, in this study MSF was assumed to be active from 2008 until 2014, with no AS by PNLTHA in this region. Two AS diagnostic algorithm specificity values were fitted: for MSF and PNLTHA activities, with the MSF specificity being lower. In this study,

the specificity of MSF and PNLTHA activity are included in the fitting, however values for the PNLTHA specificity – higher than that of the MSF specificity – result from the prior distribution (and were included solely to provide variable estimates for use in future projections). In 2014, MSF changed their diagnostic algorithm in Ango and Ganga and although some unconfirmed cases were reported (removed from the data used here), the specificity for the analysed (confirmed) cases in these HZs was taken to be 100%.

6. **Passive screening**

6.1. We now include gradual improvements in passive detection (from the start of activities to 2020) in more coordinations, expanding from Bandundu Nord, Bandundu Sud and Kongo Central coordinations in previous studies [1, 2], and now adding in Équateur Nord, Équateur Sud, Kinshasa, Kasaï Oriental.

6.2. Since 2014, there has been no AS and no PS (no tests available) in the Bas Uélé region and PS has been simulated as absent in the model to reflect this.

6.3. We are now using a smaller overdispersion value for passive case observations, which results in less predicted variation in case reporting. This applies to all the coordinations except for the Bas Uélé region, where we keep the previous overdispersion value (and have more uncertainty).

Projections

7. This study includes more granular details about vector control (VC), particularly for the simulation of partial coverage of health zones with Tiny Target deployments (both past and future).

8. We now use a stochastic model for doing projections which tracks integer numbers of infections and is better at capturing the distribution in elimination times as it has a clearly defined time when zero is reached.

9. In our GUI (https://hatmepp.warwick.ac.uk/DRCCEA/v8/ we now show aggregations for coordinations or the whole country as well as individual health zones. Aggregations are computed by summing up outcomes across different health zones based on the optimal strategy in each health zone to achieve the specific objective – e.g. not all health zones would have the same optimal strategy to achieve the EoT by 2030 objective.

10. In previous studies for the DRC we have often used only one model [1, 5, 6]. Here our default outputs are from an “ensemble” model which includes outputs from the models with and without transmission from animals based on the weighted model evidence for each health zone, as done in Crump et al. [2].

S1.2 New data extraction

↩ Return to the Supplement Table of Contents

The methods presented in this section are adapted from the previous DRC fitting study by Crump et al. [1] which used data from 2000–2016. Here we extend the same method, producing both a health-zone level and health-area level extraction to allow more flexibility for future work, and also to include data from 2000–2020.

S1.2.1 Data

HAT Atlas data The DRC WHO HAT Atlas data [7] were provided in a spreadsheet with rows representing case reports or screening events. Each record contains columns for the number of people screened, the number of cases reported at each stage, the type of screening method (active or passive), and the year. Each record also contains multiple fields describing the location: Province, Health Zone, Health Area, Location name, Neighbourhood, Quarter, Sector, Group, District, Territory, and a geolocation (latitude and longitude). In total, there were 153,876 records; of which 146,256 (95.0%) had their geolocation field populated. Note that health areas should be contained within exactly one health zone which is also contained in exactly one province and one coordination. Coordinations are not necessarily contained within one province, and vice versa.

Entries for PS with no cases detected, and entries for AS where both the number of people screened and the number of cases detected are either zero or missing, were dropped from the dataset. After removing these records, we are left with 125,045 case and screening records (119,669 of which had geolocation data, 95.7%). Among these entries, there were 24,568 unique combinations of values in the geolocation, province, health zone, health area, location and territory fields, of which 21,575 had geolocation information and 2,993 did not.

Recorded region identifiers:			Number
Province	Health zone	Health area	n
✓	✓	✓	120597
✓	✓		3213
✓		✓	14
✓			1221

Table A: Number of WHO HAT Atlas records with different combinations of the province, health zone and health area recorded.

Shapefiles To match geolocations to health zones (HZs) and health areas (HAs), we used two shapefiles provided by the American Red Cross (ARC) [8]. The first of these is a map of HZ boundaries, with 519 records covering the whole DRC. The second is a map of HAs covering most of the country (all the DRC except Lualaba and Kasaï-Central provinces, most of Sankuru and Nord-Kivu provinces, portions of the north of former Équateur province, and Poko and Monkoto HZs). Both shapefiles provided fields stating the name of the province and HZ, and, in the case of the area map, also the HA. The province and HZ information in both shape files were used to augment the data with the coordination. The ARC HA file has more granular boundaries (HAs are smaller than HZs), so wherever possible we used the HZ and HA data from this file, however, it does not cover the whole of the DRC. The ARC HZ file, on the other hand, has slightly less granular boundaries and some inconsistencies with the HA shapefile in places; by comparing both shapefiles to maps with rivers (which are often used as boundary definitions) it is assumed that the HA shapefile is also more accurate. Nevertheless, the HZ shapefile is complete and covers the whole country. We also used the combined information from these two shapefiles to identify smaller gaps in HZs which are partially mapped on the HA shapefile. In particular, any contiguous gaps of at least 50km^2 in the HA shapefile which fell into the same zone in the HZ file were combined into pseudo-HAs and numbered to catch the cases that fell into unmapped HAs.

Additional geographic information The following geographical information sourced from the Humanitarian Data Exchange was also used [8]:

- a HZ shapefile from the United Nations Office for the Coordination of Humanitarian Affairs (OCHA);
- an OCHA file of geolocations of localities; and
- a file of geolocations of health facilities from the Global Healthsite Mapping Project.

These data were used to assist in matching and locating the gHAT data, by providing alternative spellings of names and potential geolocations for non-geolocated gHAT locations. The locality and health facility lists were concatenated, and this enlarged locality set and the OCHA HZ map were assigned geographical identifiers as per our shapefile of choice.

Matching HAT Atlas records to DRC shapefile Similarly to [1], the names of all locations in both the data and the shapefiles were normalised to facilitate matching between different data sources. The normalisation process consisted of removing any diacritics, converting all names to lowercase, trimming superfluous whitespace, replacing Roman numerals with Arabic numerals, and normalising some spellings by removing leading 'm', 'n', or 'g' when followed by a consonant, and replacing leading 'ts' with 's'. Additionally, some alternative names and spellings were manually added as they were noticed.

Location records were then placed on the two shapefiles provided by the ARC, and the names of the matching HAs were stored. Locations were then matched in a series of stages, starting with the most precise matches and slowly becoming more permissive until the majority of data points had been matched.

1. Locations with known HZ and HA, which match a HA on the ARC HA shapefile, and where the recorded HZ and HA match the geolocated HZ and HA on the ARC HA shapefile. These locations were matched to the recorded HZ and HA. This matched 4,441 (34.5% of records with geolocation) locations.

2. Locations with known HZ and HA, which match a HA on the ARC HA shapefile, where the recorded HZ matches the geolocated HZ, and where the geolocation is within 5km of the recorded HA on the ARC HA shapefile. These locations were matched to the recorded HZ and HA. This matched a further 2,117 (9.8% of records with geolocation) locations. [Cumulatively: 9,558 or 44.3%]

3. Next, we used the location information. The long list of locations provided by the additional data sources was placed onto the same ARC shapefiles. Data records were then matched to any locations on this list with matching location and territory records (after normalisation), and when the record geolocation was within 10km of the geolocation associated with the location. Again, we only consider records where the recorded HZ matched the geolocated HZ on the ARC HA shapefile. This then gives three possible candidate HAs: the one stated in the record, the one matched on the HA shapefile, and the one matched by location. If two of these agreed we used this value, however, if all three still disagreed then we chose to trust the HA matched with by geolocation. These records were matched to the recorded HZ and the HA as described above. This matched a further 662 (3.1% of records with geolocation) locations. [Cumulatively: 10,220 or 47.4%]

4. The remaining records which match a HA on the ARC HA shapefile, and where the recorded HZ and geolocated HZ on the HA map agree, were matched according to their geolocation. The records were matched to the recorded HZ and the geolocated HA according to the ARC HA shapefile. This matched a further 5462 (25.3% of records with geolocation) locations. [Cumulatively: 15,682 or 72.7%]

5. Now we consider records where the recorded HZ does not match the geolocated HZ but is nearby. Similarly to step 2, we consider records where the geolocation falls within 5km of the recorded HZ according to the ARC HA shapefile. We then perform three matching steps analogous to steps 1–3 for these close HZ matches.

    - We then consider records where the recorded HA matches the geolocated HA. These records are matched to the recorded HZ and the recorded HA. This matched a further 74 (0.3% of records with geolocation) locations. [Cumulatively: 15,756 or 73.0%]
    - We then consider records where the geolocation is within 5km of the recorded HA according to the ARC HA shapefile. These records are matched to the recorded HZ and the recorded HA. This matched a further 446 (2.1% of records with geolocation) locations. [Cumulatively: 16,202 or 75.1%]

- We then consider records where the location matches a nearby location as in step 3 above. To match the HZ for these records, we apply the following steps. If the record has entries for both HZ and HA, we match to the recorded HZ. If not, we check whether the record's geolocation matched an area present on the ARC HA shapefile. If so, we match to the geolocated HZ using the ARC HA shapefile, if not we use the ARC HZ shapefile. To match the HA, then as above, if the recorded HA and the HA from the locality agree then we use this, otherwise we fall back to the geolocated HA according to the ARC HA shapefile. This matched a further 54 (0.3% of records with geolocation) locations. [Cumulatively: 16,256 or 75.3%]

6. After mapping all the records where the recorded health zone matched the ARC HA shapefile, we were left with the records which either fell in the gaps between health zone polygons or have inconsistent data.

   - First we considered locations where the recorded health zone matched the ARC HZ shapefile, and where they fall into an area which is not mapped on the ARC HA file. These records are matched to the recorded HZ and to the appropriate pseudo-HA based on their geolocation. This matched a further 2635 (12.2% of records with geolocation) locations. [Cumulatively: 18,891 or 87.6%]
   - Now we consider locations where the geolocation is within 5km of the recorded health zone according to the ARC HZ shapefile, and where the location does not map onto the ARC HA shapefile. These records are also matched to the recorded health zone. To assign a health area, we find the nearest health area (or pseudo-HA) which falls in the assigned health zone (according to its boundary in the ARC HZ shapefile), and use this. This matched a further 292 (1.4% of records with geolocation) locations. [Cumulatively: 19,183 or 88.9%]

7. For locations with inconsistent data:

   - Firstly, locations where the recorded health area matches the geolocated health area according to the ARC HA shapefile. For these, we used the recorded health area and used the geolocated health zone according to the ARC HA shapefile (to match the health area). This matched a further 192 (0.9% of records with geolocation) locations. [Cumulatively: 19,375 or 89.8%]
   - Next, we match to the locality list and find any locations where the locality and territory match and the geolocations are within 5km of each other. If the health area of the locality according to the ARC HA shapefile matches either the recorded HA or the geolocation HA then we select that as the health area. Note that this includes the possibility of the geolocation and locality both matching the same pseudo-HA. To match the health zone, we use the health zone of the health area (or pseudo-HA) we have just assigned it to, either from the ARC HA map if we matched a real health area, or from the ARC HZ map if the location is not on the ARC HA shapefile. This matched a further 196 (0.5% of records with geolocation) locations. [Cumulatively: 19,471 or 90.2%]

8. Finally, if we cannot reconcile the recorded data with the geolocations, on the advice of the WHO we accept the geolocations as the more authoritative source, matching the health area (or pseudo-HA) according to the ARC HA shapefile, and matching the health zone according to the ARC HA shapefile if possible, or the ARC HZ shapefile if not. This matched the final 2,104 locations with geolocation data present, giving a total of 21,575.

9. For records with no geolocation data available:

   - First, we try to match the locality data as above, matching the locality and territory columns. For any results with exactly one match, we use the geolocation from this locality to assign a health area or pseudo-HA from the ARC HA shapefile, and health zone from the same shapefile if possible or from the ARC HZ shapefile if not. This matched 49 (1.6% of records without geolocation) locations.
   - Now, we instead match by location (but not territory) and health zone, again keeping any unique matches and using the geolocation fromt he territory. This matched a further 11 (0.4% of records without geolocation) locations. [Cumulatively: 60 or 2.0%]
   - Now, we match by location and province. We match any locations from the location list with the same (normalised) name, and where the recorded province in the data record matches any of the former province name, province name, or co-ordination name of the location according to either of the ARC

shapefiles. Again, we filter for unique matches and match according to the geolocation of the matched location. This matched a further 19 (0.6% of records without geolocation) locations. [Cumulatively: 79 or 2.6%]

10. Finally, we matched the remaining records to the health zone and health area polygon according to the health zone or health area stated in WHO HAT Atlas. This allowed us to match 2,710 records (90.5% of records without a specific geolocation), giving a final cumulative total of 2,789 matched ungeolocated records, making 93.2% of non-geolocated records or 99.2% of all records. The remaining records (totalling 316 cases and 193,035 people screened) were missing the majority of location fields and were deemed unable to be matched, so were not included in our data.

Missing screening data In some HZs there are instances of years of ASs with either very high (or impossible) prevalences (e.g. >10% or with active case numbers exceeding the number of people recorded as having been screened) or where the total number of people actively screened is extremely low (i.e. <20 people). This occurs in 56 HZs for 1 or more years. For these screenings, if there are 1 or more active cases, we denote the number of people screened as "unknown" and this is later inferred during model fitting (see S1.5) as has been done in previous studies. If the number of people tested in screenings is <20 people, but no cases are found, we do not try to infer the missing number.

S1.2.2 Post-processing of data in Bas-Uélé region health zones.

Médecins Sans Frontières (MSF) were active in the Bas-Uélé region from 2007–2014, inclusive. This region comprises the Ango, Bili, Doruma, Ganga, Poko and Titule health zones. Based on information received from PNLTHA, patterns in the data, and a report by MSF [3] we modified the extracted and aggregated data for these health zones as follows:

1. All cases detected by passive screening in Ango in 2008 and Ganga in 2010 and 2011 were transferred to the active screening results. Information received indicated that active screening was effectively carried out in the medical facilities in these health zones, and disproportionately high numbers of PS cases were recorded in these years.

2. Information was received from PNLTHA that no staging was carried out in the Bas Uélé region. Therefore, all historical case records were treated as having an unknown stage regardless of whether they were recorded in active or passive screening.

3. In 2014, MSF active screenings in Ango and Ganga were more rigorous, with parasitological confirmation of some cases. However, the serological positives were still recorded as cases in data sent to WHO; the HAT Atlas does not have the split of parasitological or serological cases available. In our analysis, the 2014 active screening data in Ango and Ganga was modified to include only parasitologically confirmed cases as detailed in a report by MSF[3], still without staging information:

Health zone	Ango	Ganga
Number of AS parasitological cases in 2014	25	36
Number of people actively screened in 2014	6455	13672

As outlined in section S1.1, this differs from earlier studies, which used the unedited WHO HAT Atlas data for the Bas-Uélé region health zones and assumed that MSF was only present/active here until 2012. There has been no active screening or passive screening in these health zones since the end of MSF activities in 2014.

S1.3 Locations

↩ Return to the Supplement Table of Contents

Health zones included. We have included 165 health zones in which 37.6M people live, and which have records

of 131,941 cases in 2000–20. Our population data comes from the United Nations Office for the Coordination of Humanitarian Affairs censuses for national vaccine days [9].

Health zones excluded. We have omitted any health zones with less than 10 years of either AS or PS detection records. Therefore, our analysis omits 330 health zones, 69.9M people, and 3,789 cases – approximately 3% of all cases reported in 2000–20 (see Table B).

For the model with animal transmission, we needed at least 13 years of either AS or PS detection records. Therefore, for 10 health zones, that have been included in the overall analysis, simulations come from only the model without animal transmission.

Moreover, health zones with no major rivers or which are too urban to sustain tsetse populations are not simulated. These are primarily in urban Kinshasa, in Kasaï Orientale coordination around the area of Mbuji-Mayi, a mining town without any rivers, and Kikwit Sud in Bandundu Sud. The cases reported there are approximately 4217, approximately 3% of all cases reported in 2000–20 (see Table B). We believe that these cases were reported due to the presence of large hospitals capable of diagnosing and treating people, but that these individuals were infected with the disease in other regions that could sustain transmission.

Therefore, we have modelled transmission in the areas of DRC that constitute 94% of all the HAT Atlas cases in 2000-2020.

Population. It must be noted that estimating the population of the DRC is difficult, as the country has not held a census since 1984, and many other forms of population surveillance are inconsistent throughout the country. Our data on the population for each health zone comes from the Office for the Coordination of Humanitarian Affairs [9], and a summary of the population per health zone is shown in Table B. We have assumed that the population has a 3% annual growth rate, compounded annually. Our total population comes out to 116.2 million, which roughly aligns with the population estimate from the CIA World Factbook estimate for 2023 [10] although it is higher than the UN World Population Prospects [11].

Coordinations. For the purpose of organising gHAT control and elimination efforts, the country is partitioned into eleven regions known as coordinations. See Fig A.

Coordination	Total No. HZ	Included			Excluded - insufficient data			Excluded - urban locale		
		No. HZ	Cases 2000-2020	Populati (millions)	No. HZ	Cases 2000-2020	Populati (millions)	No. HZ	Cases 2000-2020	Populati (millions)
Bandundu Nord	20	18	36369	3.6	2	6	0.2	0	0	0.0
Bandundu Sud	32	19	30456	4.8	12	27	2.4	1	64	0.3
Equateur Nord	39	22	19340	4.7	17	21	3.6	0	0	0.0
Equateur Sud	30	9	1622	1.3	21	55	4.0	0	0	0.0
Isangi - Bas-Uélé	6	2	3297	0.2	4	3272	0.5	0	0	0.0
Isangi - Tschopo	29	4	2792	0.7	25	55	3.9	0	0	0.0
Kasai Occidental	45	18	6314	4.4	26	66	6.5	1	172	0.3
Kasai Oriental	35	22	17415	6.4	5	31	1.0	8	2874	3.1
Kinshasa	36	13	3027	4.2	9	108	1.8	14	1107	5.0
Kongo Central	30	17	4673	2.6	13	40	1.9	0	0	0.0
Maniema Katanga	29	13	4583	3.3	16	30	3.3	0	0	0.0
Sankuru	16	8	2053	1.3	8	17	1.1	0	0	0.0
No Coordination	172	NA	0	0.0	172	58	39.7	0	0	0.0
**Total**	**519**	**165**	**131941**	**37.6**	**330**	**3786**	**69.9**	**24**	**4217**	**8.7**

Table B: Summary of demographics characteristics and HAT case burden of health zones that were included compared to those excluded from the analysis. The health zones were omitted if there were fewer than 10 data points: in other words the years of AS reports available plus the years of PS reports available equalled less than 10, or if we did not believe that transmission could take place in the health zone because it was urban. Fewer than 3% of cases occurred in health zones with insufficient data and another 3% occurred in health zones that we have excluded because of their urban locale, where we believe there is no transmission. Abbreviations: HZ: health zones (zone de santé in the original French). AS: active screening, PS: passive screening.

Coordination	No. HZ	Pop. per HZ (thousands)	Pop. subtotal (millions)	2000-2020 Cases per HZ[a]	2000-2020 Sum cases	2016-2020 Cases per HZ[a]	2016-2020 Sum cases
Bandundu Nord	18	180 [110–367]	3.60	1748 [37–7186]	36369	66 [3–210]	1278
Bandundu Sud	19	249 [154–358]	4.80	592 [10–6827]	30456	36 [1–183]	1194
Equateur Nord	22	188 [84–427]	4.70	474 [25–4298]	19340	2 [0–29]	113
Equateur Sud	9	171 [45–212]	1.30	92 [19–550]	1622	2 [0–15]	34
Isangi - Bas-Uélé[b]	2	110 [88–133]	0.20	1648 [1390–1907]	3297	0 [0–0]	0
Isangi - Tschopo[b]	4	198 [106–208]	0.70	579 [41–1593]	2792	23 [0–55]	101
Kasai Occidental	18	230 [117–420]	4.40	180 [9–1982]	6314	12 [0–85]	446
Kasai Oriental	22	265 [153–543]	6.40	472 [56–4567]	17415	8 [1–58]	307
Kinshasa	13	338 [90–594]	4.20	98 [35–858]	3027	5 [0–43]	143
Kongo Central	17	133 [83–251]	2.60	249 [15–912]	4673	4 [0–51]	169
Maniema Katanga	13	237 [107–417]	3.30	391 [31–691]	4583	14 [0–74]	309
Sankuru	8	150 [95–239]	1.30	218 [13–773]	2053	20 [1–104]	331
**Total**	**165**	**210 [45-594]**	**37.58**	**342 [9-7186]**	**131941**	**9 [0-210]**	**4425**

[a] Cases are shown per health zone: median [minimum-maximum].

[b] Isangi coordination has been separated into two subregions in this analysis. Bas-Uélé is constituted of Ango health zone in Bas-Uélé Province and Doruma health zone in Haut-Uélé Province. Tshopo is constituted of Isangi, Yabaondo, Yahisuli, and Yakusu health zones in Tschopo province.

Table C: Summary of the demographic characteristics and recent vs complete case burden in health zones in the analysis, stratified by the coordinations delineated by the programme national de lutte contre la Trypanosomiase humaine africaine (PNLTHA-RDC). Abbreviation: HZ: health zone (zone de santé in the original French).

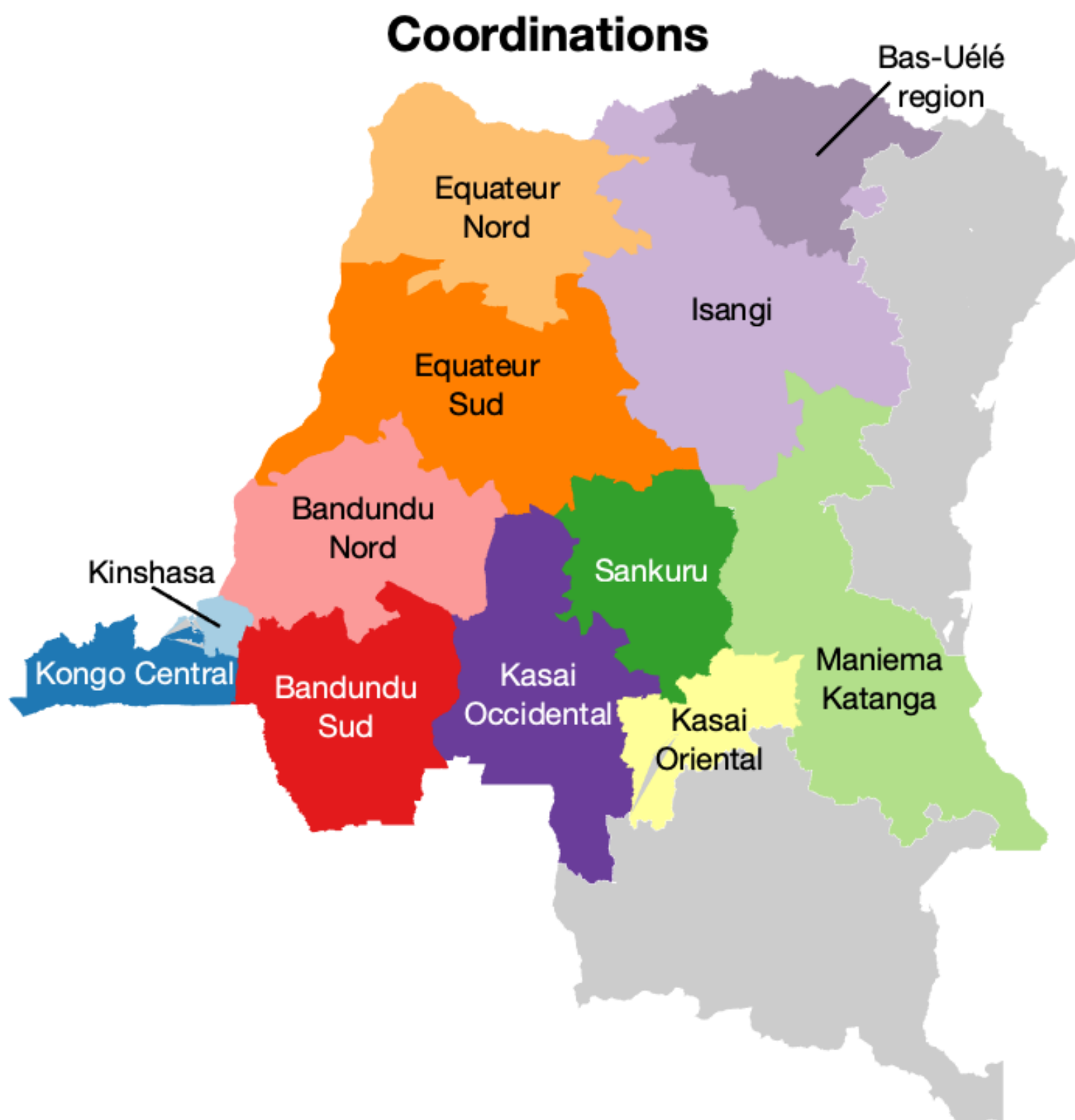

Fig A: Map of gHAT coordinations in the DRC. N.B. Isangi is a single coordination, however, we denote the region within Isangi we refer to as the Bas-Uélé region for context. Shapefiles used to produce this map were provided by Nicole Hoff and Cyrus Sinai under a CC-BY licence (current versions can be found at `https://data.humdata.org/dataset/drc-health-data`)

S1.4 Model formulation

↩ Return to the Supplement Table of Contents

In this study, we use two mechanistic transmission model variants, both of which have a group of people at high risk of gHAT infection who do not participate in AS and a low-risk group of people who participate at random. The model variants differ concerning the presence or absence of non-human animal transmission cycles [2]. Below, we show the diagram and model equations, both reproduced from [2].

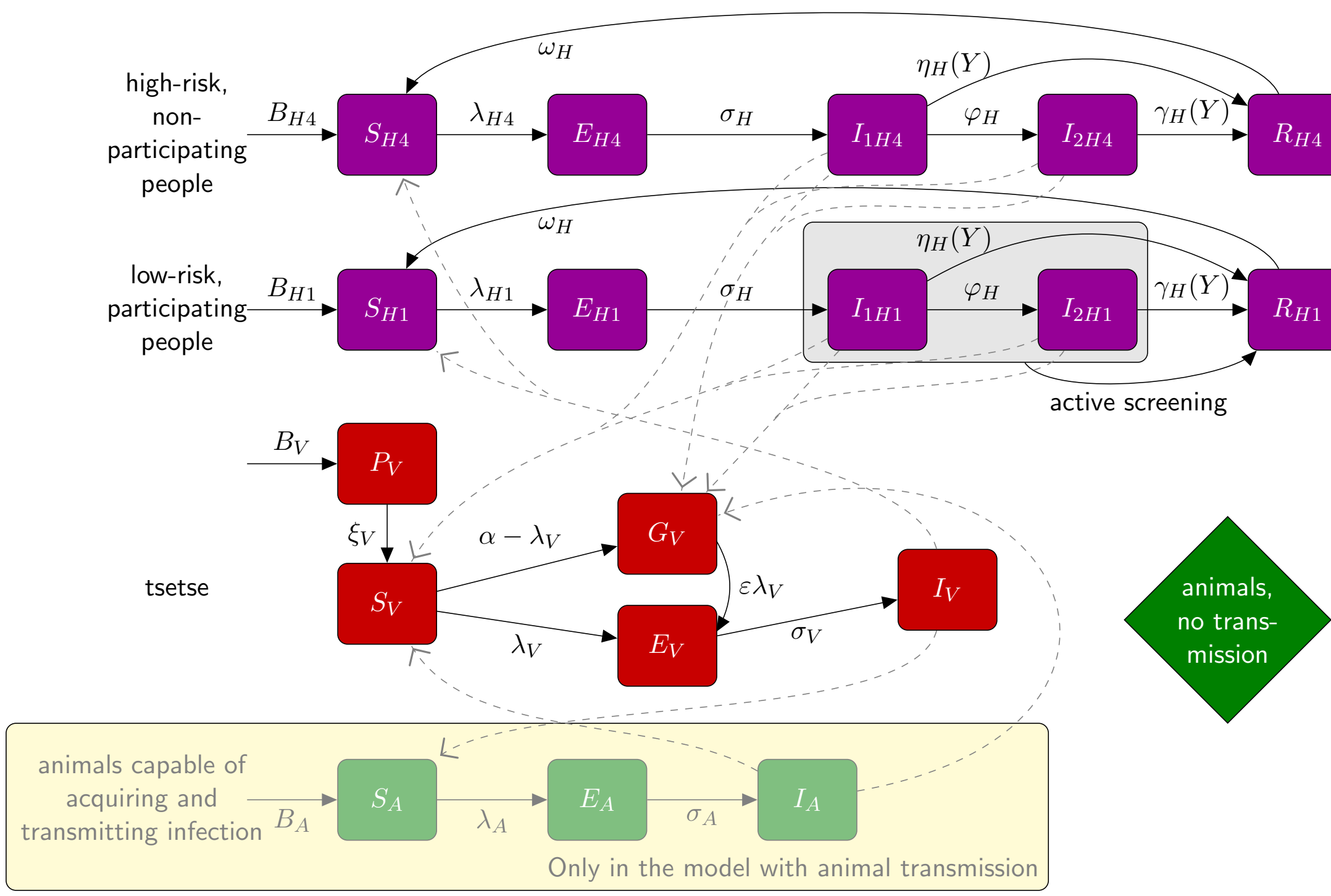

Fig B: Warwick gHAT intervention model compartmental diagram. Purple boxes denote human infection/risk compartments, red boxes denote tsetse infection compartments, and green boxes denote non-human animal infection compartments (only in the model variant with possible animal transmission). Solid lines represent the transition between infection states, and dashed lines are transmission pathways. Abbreviations and parameters: see text. Reproduced from [2] under a CC-BY licence.

For this set of model compartments, we can describe both deterministic and stochastic variations of the model. Below are the ODEs describing the deterministic dynamics. It was this version of the model which was used for fitting, whereas the analogous stochastic model, simulated using tau-leaping with a one-day time step, was used for sampling and projections into the future. More detail on the advantages and disadvantages of the deterministic and stochastic models is presented in Davis et al. [12], however, this previous work has demonstrated that using the deterministic model for fitting followed by the stochastic model for projections provides the speed of the deterministic fitting process but the integer outputs and stochastic variation generated by the stochastic model.

$$
\text{Humans}\quad
\begin{cases}
\dfrac{\mathrm{d}S_{Hi}}{\mathrm{d}t} &= \mu_H N_{Hi} + \omega_H R_{Hi} - \alpha m_{\text{eff}} f_i \dfrac{S_{Hi}}{N_{Hi}} I_V - \mu_H S_{Hi} \\
\dfrac{\mathrm{d}E_{Hi}}{\mathrm{d}t} &= \alpha m_{\text{eff}} f_i \dfrac{S_{Hi}}{N_{Hi}} I_V - (\sigma_H + \mu_H) E_{Hi} \\
\dfrac{\mathrm{d}I_{1Hi}}{\mathrm{d}t} &= \sigma_H E_{Hi} - (\varphi_H + \eta_H(Y) + \mu_H) I_{1Hi} \\
\dfrac{\mathrm{d}I_{2Hi}}{\mathrm{d}t} &= \varphi_H I_{1Hi} - (\gamma_H(Y) + \mu_H) I_{2Hi} \\
\dfrac{\mathrm{d}R_{Hi}}{\mathrm{d}t} &= \eta_H(Y) I_{1Hi} + \gamma_H(Y) I_{2Hi} - (\omega_H + \mu_H) R_{Hi}
\end{cases}
$$

$$
\text{Animals}\quad
\begin{cases}
\dfrac{\mathrm{d}S_A}{\mathrm{d}t} &= \mu_A N_A - \alpha m_{\text{eff}} f_A \dfrac{S_A}{N_A} I_V - \mu_A S_A \\
\dfrac{\mathrm{d}E_A}{\mathrm{d}t} &= \alpha m_{\text{eff}} f_A \dfrac{S_A}{N_A} I_V - (\sigma_A + \mu_A) E_A \\
\dfrac{\mathrm{d}I_A}{\mathrm{d}t} &= \sigma_A E_A - \mu_A I_A
\end{cases}
$$

$$
\text{Tsetse}\quad
\begin{cases}
\dfrac{\mathrm{d}P_V}{\mathrm{d}t} &= B_V N_H - (\xi_V + \frac{P_V}{K}) P_V \\
\dfrac{\mathrm{d}S_V}{\mathrm{d}t} &= \xi_V \mathbb{P}(\text{survive pupal stage}) P_V - \alpha S_V - \mu_V S_V \\
\dfrac{\mathrm{d}E_{1V}}{\mathrm{d}t} &= \alpha\big(1 - f_T(t)\big) p_V \big(\sum_i f_i \dfrac{(I_{1Hi} + I_{2Hi})}{N_{Hi}} + f_A \dfrac{I_A}{N_A} \big)(S_V + \varepsilon G_V) \\
& \qquad -(3\sigma_V + \mu_V + \alpha f_T(t)) E_{1V} \\
\dfrac{\mathrm{d}E_{2V}}{\mathrm{d}t} &= 3\sigma_V E_{1V} - (3\sigma_V + \mu_V + \alpha f_T(t)) E_{2V} \\
\dfrac{\mathrm{d}E_{3V}}{\mathrm{d}t} &= 3\sigma_V E_{2V} - (3\sigma_V + \mu_V + \alpha f_T(t)) E_{3V} \\
\dfrac{\mathrm{d}I_V}{\mathrm{d}t} &= 3\sigma_V E_{3V} - (\mu_V + \alpha f_T(t)) I_V \\
\dfrac{\mathrm{d}G_V}{\mathrm{d}t} &= \alpha(1 - f_T(t))\Big(1 - p_V\big(\sum_i f_i \dfrac{(I_{1Hi} + I_{2Hi})}{N_{Hi}} + f_A \dfrac{I_A}{N_A} \big)\Big) S_V \\
& \qquad -\alpha\Big(f_T(t) + \big(1 - f_T(t)\big) p_V \varepsilon \big(\sum_i f_i \dfrac{(I_{1Hi} + I_{2Hi})}{N_{Hi}} + f_A \dfrac{I_A}{N_A} \big)\Big) G_V \\
& \qquad -\mu_V G_V
\end{cases}
\tag{1}
$$

The function which describes the probability of a host-seeking tsetse both hitting a Tiny Target and dying as a result, f_T, is time-dependent (t, in days) from when the targets were first deployed:

$$
f_T(t) = f\text{max} \left(1 - \frac{1}{1 + \exp(-0.068(\text{mod}(t, 182.5) - 127.75))} \right) \tag{2}
$$

and $f\text{max}$ is the maximum daily probability of contacting a Tiny Target and dying as a result. f_T modifies all the bite rates α in our tsetse equations to produce an additional Tiny-Target-induced mortality for tsetse. The choice of parameterisation of this function in different locations is described in the next section.

S1.4.1 Assumptions about past interventions

Regions with no transmission

In the previous model fitting studies [1, 2], it was assumed that no transmission took place in the health zones that constituted urban Kinshasa. In this study we have added a few health zones into the analysis where we believe

Notation	Description	Value	
N_H*	Total human population size in 2015	Fixed for each health zone	[9]
μ_H	Natural human mortality rate	5.4795×10^{-5} days^{-1}	[13]
B_H	Total human birth rate	$= \mu_H N_H$	
σ_H	Human incubation rate	0.0833 days^{-1}	[14]
φ_H	Stage 1 to 2 progression rate	0.0019 days^{-1}	[15, 16]
ω_H	Recovery rate or waning-immunity rate	0.006 days^{-1}	[17]
Sens	Active screening diagnostic sensitivity	0.91	[18]
B_V†	Tsetse birth rate (per capita rate of depositing new pupae)	0.0505 days^{-1}	[4]
ξ_V	Rate of pupal development to adult flies	0.037 days $^{-1}$	[4]
K‡	Pupal carrying capacity	$= 111.09 N_H$	[4]
$\mathbb{P}$(pupating)	Probability of a pupa surviving to emerge as an adult fly	0.75	[4]
μ_V	Tsetse mortality rate	0.03 days^{-1}	[14]
σ_V	Tsetse incubation rate	0.034 days^{-1}	[19, 20]
α	Tsetse bite rate	0.333 days^{-1}	[21]
p_V	Probability of tsetse infection per single infective bite	0.065	[14]
ε	Reduced susceptibility factor for non-teneral (previously fed) flies	0.05	[22]
f_H	Proportion of blood-meals on humans	0.09	[23]
η_H^{pre}	Treatment rate from stage 1, pre-1998	0	Assumed
disp_{act}§	Overdispersion parameter for active detection	4×10^{-4}	[1]
$\text{disp}_{\text{pass}}$§	Overdispersion parameter for passive detection	1.68×10^{-5}¶	[1]
**Parameters specific to the model with animal transmission...**			
μ_A	Natural animal mortality rate	0.0014 days^{-1}	Assumed
σ_A	Animal incubation rate	0.0833 days^{-1}	[14]

*The model is internally scaled such that the population size in all years corresponds to the population in 2015 (outputs are back-transformed to reflect an assumed annual population growth rate of 3% across the DRC).
†The value of B_V was chosen to maintain constant population size in the absence of vector control interventions.
‡The value of K was chosen to reflect the observed bounce back rate.
§Over-dispersion values were originally chosen based on a comparison of the median of the distributions of log posterior probability from MCMC runs with ρ fixed at a range of values for two example health zones under the model without animal transmission [1].
¶The over-dispersion value for passive screening in the Bas-Uele region is 2.8×10^{-5}.

Table D: Model parameterisation (fixed parameters). Notation, a brief description, and the values used for fixed parameters. This table is updated from [1, 2].

Notation	Description	Prior distribution*	Percentiles of prior distribution [2.5, 50 & 97.5%]	Unit
R_0	Basic reproduction number (NGM approach)	$1 + \text{Exp}(10)$	[1.003, 1.069, 1.369]	-
r	Relative bites taken on high-risk humans	$1 + \Gamma(3.68, 1.09)$	[2.015, 4.654, 10.028]	-
k_1	Proportion of low-risk people	$\text{B}(16.97, 3.23)$	[0.6564, 0.8514, 0.9609]	-
$\eta_H^{\text{post}\dagger}$	Treatment rate from stage 1, post endemic equilibrium	$\Gamma\big(3.54, 5.32 \times 10^{-5}\big)$	[4.59, 17.1, 42.9]$\times 10^{-5}$	days^{-1}
γ_H^{post}	Combined treatment and disease-induced death rate from stage 2, 1998 onwards	$\Gamma(6.2082, 0.001)$	[2.33, 5.88, 12.0]$\times 10^{-3}$	days^{-1}
γ_H^{pre}	Combined treatment and disease-induced death rate from stage 2, pre-1998	$\Gamma(6.2082, 0.001)$	[2.33, 5.88, 12.0]$\times 10^{-3}$	days^{-1}
Spec	Active screening diagnostic specificity	$0.998 + (1 - 0.998)\,\text{B}(7.23, 2.41)$	[0.9989, 0.9995, 0.9999]	-
u	Proportion of stage 2 cases reported from passive screening	$\text{B}(20, 40)$	[0.2208, 0.3315, 0.4564]	-
$d_{\text{change}}{}^{\dagger}$	Midpoint year for passive improvement	$2000 + (2017 - 2000)\,\text{B}(5, 6)$	[2003.2, 2007.7, 2012.5]	year
$\eta_{H_{\text{amp}}}{}^{\dagger}$	Relative improvement in passive screening stage 1 detection rate	$\Gamma(2.5133, 1.3216)$	[0.556, 2.893, 8.509]	-
$\gamma_{H_{\text{amp}}}{}^{\dagger}$	Relative improvement in passive screening stage 2 detection rate	$\Gamma(2.3095, 0.5727)$	[0.198, 1.137, 3.493]	-
$d_{\text{steep}}{}^{\dagger}$	Speed of improvement in passive screening detection rate	$\Gamma(39.57, 0.0270)$	[0.761, 1.058, 1.424]	years^{-1}
**Parameters specific to the model with animal transmission...**				
f_A	Proportion of blood meals on reservoir animals	$\text{B}(1.3, 1.3)$	$[0.046, 0.5, 0.954]$	-
k_A	Relative size of animal reservoir population	$\Gamma(1.26, 19.3)$	$[1.18, 18.3, 81.4]$	-

*Where Exp$(.)$, $\Gamma(.)$ and B$(.)$ are the exponential, gamma (parameterised with shape and scale) and beta distributions, respectively.
†Former province-specific priors were originally used for η_H^{post}, d_{change}, $\eta_{H_{\text{amp}}}$, $\gamma_{H_{\text{amp}}}$ and d_{steep}; prior distributions and percentiles for the former province of Bandundu presented (i.e., Bandundu Nord and Bandundu Sud coordinations), see Table F for other coordinations.

Table E: Model parameterisation (fitted parameters). Notation, brief description, and information on the prior distributions for fitted parameters. This table is updated from Crump et al. [2].

Parameter		
**Coordination(s)** health zone(s)	**Prior distribution***	**Percentiles of prior distribution**
η_H^{post} – Treatment rate from stage 1, post endemic equilibrium		
**Bandundu Nord & B. Sud**	$\Gamma(3.54, 5.32 \times 10^{-5})$	$[4.59, 17.1, 42.9] \times 10^{-5}$
**Équateur Nord & É. Sud**	$\Gamma(4.92, 4.51 \times 10^{-5})$	$[7.12, 20.7, 45.7] \times 10^{-5}$
**Isangi**	$\Gamma(1.16, 9.27 \times 10^{-5})$	$[4.24, 79.0, 373] \times 10^{-6}$
**Kasaï Occidental**	$\Gamma(10.9, 3.03 \times 10^{-5})$	$[1.64, 3.20, 5.53] \times 10^{-4}$
**Kasaï Oriental**	$\Gamma(2.90, 5.87 \times 10^{-5})$	$[3.38, 15.1, 41.5] \times 10^{-5}$
**Kinshasa**	$\Gamma(1.26, 8.91 \times 10^{-5})$	$[5.44, 84.4, 376] \times 10^{-6}$
**Kongo Central**	$\Gamma(12.0, 2.89 \times 10^{-5})$	$[1.78, 3.36, 5.68] \times 10^{-4}$
**Maniema-Katanga**	$\Gamma(4.25, 4.85 \times 10^{-5})$	$[5.90, 19.0, 44.3] \times 10^{-5}$
Health zones in Katanga†	$\Gamma(1.29, 8.79 \times 10^{-5})$	$[5.88, 86.2, 376] \times 10^{-6}$
**Sankuru**	$\Gamma(2.90, 5.87 \times 10^{-5})$	$[3.38, 15.1, 41.5] \times 10^{-5}$
d_{change} – Midpoint year for improvement in passive detection rate		
**Bandundu Nord & B. Sud**	$2000 + (2017 - 2000)\,\text{B}(5, 6)$	$[2003.2, 2007.7, 2012.5]$
**Équateur Nord & É. Sud**	$2000+(2020 - 2000)\,\text{B}(2.79, 23.1)$	$[2000.4, 2002, 2005]$
**Kasaï Oriental**	$2000+(2020 - 2000)\,\text{B}(2.79, 23.1)$	$[2000.4, 2002, 2005]$
**Kinshasa**	$2000+(2020 - 2000)\,\text{B}(2.79, 23.1)$	$[2000.4, 2002, 2005]$
Masa health zone‡	Fixed parameter value, $d_{\text{change}} = 2015.5$	
**Kongo Central**	Fixed parameter value, $d_{\text{change}} = 2015.5$	
$\eta_{H_{\text{amp}}}$ – Relative improvement in passive stage 1 detection rate		
**Bandundu Nord & B. Sud**	$\Gamma(2.51, 1.32)$	$[0.556, 2.89, 8.51]$
**Équateur Nord & É. Sud**	$\Gamma(1, 2.17)$	$[0.055, 1.510, 8.010]$
**Kasaï Oriental**	$\Gamma(1, 2.17)$	$[0.055, 1.510, 8.010]$
**Kinshasa**	$\Gamma(1, 2.17)$	$[0.055, 1.510, 8.010$
Masa health zone	$\Gamma(2.51, 1.32)$	$[0.556, 2.89, 8.51]$
**Kongo Central**	$\Gamma(2.51, 1.32)$	$[0.556, 2.89, 8.51]$
$\gamma_{H_{\text{amp}}}$ – Relative improvement in passive stage 2 detection rate		
**Bandundu Nord & B. Sud**	$\Gamma(2.31, 0.57)$	$[0.198, 1.14, 3.49]$
**Équateur Nord & É. Sud**	$\Gamma(1, 1.0014)$	$[0.0254, 0.6943.69]$
**Kasaï Oriental**	$\Gamma(1, 1.0014)$	$[0.0254, 0.6943.69]$
**Kinshasa**	$\Gamma(1, 1.0014)$	$[0.0254, 0.6943.69]$
Masa health zone	$\Gamma(2.31, 0.57)$	$[0.198, 1.14, 3.49]$
**Kongo Central**	$\Gamma(2.31, 0.57)$	$[0.198, 1.14, 3.49]$
d_{steep} – Speed of improvement in passive detection rate		
**Bandundu Nord & B. Sud**	$\Gamma(39.6, 2.70 \times 10^{-2})$	$[0.761, 1.06, 1.42]$
**Équateur Nord & É. Sud**	$\Gamma(15.7, 0.51)$	$[4.55, 7.84, 12.4]$
**Kasaï Oriental**	$\Gamma(15.7, 0.51)$	$[4.55, 7.84, 12.4]$
**Kinshasa**	$\Gamma(15.7, 0.51)$	$[4.55, 7.84, 12.4]$
**Kongo Central**	$\Gamma(15.7, 0.51)$	$[4.55, 7.84, 12.4]$
Spec_{MSF} – Specificity of MSF active screening algorithm		
**Isangi** – Bas-Uele region§	$\text{B}(299, 2.87)$	$[0.977, 0.992, 0.998]$

*Where $\Gamma(.)$ and $\text{B}(.)$ are the gamma (parameterised with shape and scale) and beta distributions, respectively.
†Kabalo, Kongolo, Mbulula and Nyunzu health zones.
‡Masa health zone is in Kinshasa coordination but Kongo Central province. It was assumed to be subject to passive screening improvement in the same period as Kongo Central.
§Ango, Ganga and Doruma health zones, where intervention activities were carried out by MSF.

Table F: Model parameterisation (fitted parameters): coordination or health zone specific priors. Adapted from Crump et al. [2].

local transmission is possible due to tsetse presence: Nsele, Masa, Mont Ngafula 1 & 2, and Maluku 1 & 2. These are denoted as "no transmission" health zones in our graphical user interface maps https://hatmepp.warwick.ac.uk/DRCCEA/v8/.

Health zones with no data or insufficient data for fitting the model are excluded, as described in Section S1.2. There may be some infection in these locations, although we believe that most of the "no data" health zones are unlikely to have transmission. The "no data" and "<10 data point" health zones can be viewed in our graphical user interface maps https://hatmepp.warwick.ac.uk/DRCCEA/v8/.

Passive detection

The integration of gHAT case detection through peripheral health centres using RDTs is an area with limited documented evidence in the DRC [24, 25]. While positive effects have been observed in other countries, such as Chad [26, 27], the follow-up for infection confirmation among RDT-positive cases in the DRC is reported to have a high level of attrition if the health centre where someone was screened does not have immediate confirmation available [28]. Despite this, there is evidence, particularly from the passive case staging data over time, that time to detection through PS has decreased over time [1, 29].

In the same manner as our previous study [2], for improvements between the start of activities and 2020 we use the same following equations to describe transmission rates from infected classes:

$$\eta_H(Y) = \eta_H^{\text{post}} \left[1 + \frac{\eta_{H_{\text{amp}}}}{1 + \exp\left(-d_{\text{steep}}(Y - d_{\text{change}})\right)}\right] \tag{3}$$

$$\gamma_H(Y) = \gamma_H^{\text{post}} \left[1 + \frac{\gamma_{H_{\text{amp}}}}{1 + \exp\left(-d_{\text{steep}}(Y - d_{\text{change}})\right)}\right] \tag{4}$$

We assume that all stage 1 infections are either reported as cases or progress to stage two, but that some of the exits from stage 2 are due to death from gHAT disease. In 1998 the reporting probability for an exit from stage 2 is given by u, however as the exit rate from stage 2 increases this reporting probability does not stay constant, but increases (proportionally more people would be detected and treated with higher exit rates). When we compute reporting rates from stage 2 we therefore use the following:

$$\text{Death rate} = (1 - u)\gamma_H^{\text{post}} \tag{5}$$

$$\text{Stage 2 reporting incidence} = (\gamma_H(Y) - \text{Death rate})(I_{2H1} + I_{2H4}) \tag{6}$$

The Bas Uélé region

Previously, it was assumed that screening activities in the former province of Orientale (current-day Bas Uélé) had been carried out by Médecins Sans Frontières (MSF) until 2012, allowing us to differentiate between MSF-specific specificity and sensitivity parameters and those associated with PNLTHA activities in the subsequent years. The specificity in these two periods was fitted with the MSF-specific specificity constrained to be lower than the PNLTHA specificity, while the sensitivity values were assumed to have constant values (0.91 in all years). In these fits we have refined this assumption based on patterns in the data and information received from PNLTHA and from an MSF report [3], with MSF-lead activity being limited to some health zones in the Bas-Uélé region: Ango, Bili, Doruma, Ganga, Poko and Titule. MSF were active in these health zones between 2007–2014, and there have been no gHAT control activities, neither active nor PS, in this region after this. As a result, there is no information on the specificity of PNLTHA screening, and so this parameter reflects the prior distribution (our prior belief).

Historical VC

f_{max} in Equation 2 is chosen such that the tsetse population after one year is 80% multiplied by the proportion of recent gHAT cases coverage by the intervention, except for in Yasa Bonga in Bandundu Sud coordination which already has a measured 90% reduction in the intervention area [30].

To calculate an estimate of the case coverage of the intervention areas, we applied the following algorithm: First, we selected the river segments in the DRC where VC had previously been applied from the HydroRivers dataset

Health zone	Year started	Tsetse reduction after 1 year	Estimated coverage (based on 2016–2020 cases)
Mosango	2015.5	assumed 80%	11%
Yasa Bonga	2016.5	reported 90%	73%
Kenge	2017.5	assumed 80%	28%
Masi Manimba	2018	assumed 80%	53%
Bandundu	2019.5	assumed 80%	66%
Bolobo	2019.5	assumed 80%	14%
Kikongo	2019.5	assumed 80%	18%
Kwamouth	2019.5	assumed 80%	46%
Bulungu	2021	assumed 80%	28%
Kimputu	2021	assumed 80%	35%
Mokala	2021	assumed 80%	15%
Bagata	2021.5	assumed 80%	47%
Bokoro	2021.5	assumed 80%	15%
Ipamu	2021.5	assumed 80%	10%

Table G: List of health zones with previous vector control (VC) deployments. This table includes health zones which have had "inadvertent VC" as boundary rivers had VC due to official deployments in neighbouring health zones. The year started is our modelled start time to the nearest half year, the tsetse reduction after 1 year is assumed to be 80% in all health zones apart from Yasa Bonga where a 90% reduction was reported. Case coverage is estimated using geolocated case data from 2016–2020 as described above.

and then created a 5km buffer zone around these areas. For each health zone, we then intersected these buffer zones with the health zone and counted the number of cases that fell inside this buffer between 2016 and 2020. Any cases that fell into multiple buffers (either for the same or multiple health zones) were split evenly between these buffers and counted fractionally to the relevant health zones. The total number of cases inside the buffers was then compared to the total number of cases in the health zone for the same period to estimate a fractional case coverage. The 80% (90% in Yasa Bonga) reduction was then scaled by this coverage to produce a new estimate for f_{max}.

S1.5 Model fitting

↩ Return to the Supplement Table of Contents

An adaptive Metropolis-Hastings Markov chain Monte Carlo (MCMC) algorithm was used to fit two deterministic transmission model variants to epidemiological data as in previous modelling studies in the DRC [1, 2]. The model variants differ with regard to the presence or absence of animals contributing to gHAT transmission. Since these earlier studies, the data has been updated to span the years 2000 to 2020 (rather than 2000 to 2016) before aggregation at the health zone level within a year. As before, model fitting was carried out independently within each health zone.

Our ODE models were run from their endemic equilibrium. Two chains were run in the MCMC, and they were initialised using the fixed parameters and by random perturbations around supplied, individually valid, initial values of each parameter being fitted, rejecting those parameter sets that do not produce a valid posterior probability.

Likelihood

As described in Crump et al. [2], eight parameters; $R_0, r, \eta_H, \gamma_H, b_{\gamma_{H0}}, k_1, u,$ and Spec were fitted in all health zones for both models. A further two parameters; k_A and f_A, were fitted in all health zones for the model with animal transmission. Additional parameters were included as required (combinations of d_{change}, $\eta_{H_{\text{amp}}}$, $\gamma_{H_{\text{amp}}}$, d_{steep} and Spec_{MSF} as appropriate, see above).

For fitting the model to case data, we transform model ODE solutions (for S1.2.1) into annual case reporting denoted A_{M1}, A_{M2}, for active stage 1 and stage 2 and P_{M1}, P_{M2}, for passive stage 1 and 2. Since we always know the stage (1 or 2) in the model simulations, there is no requirement for a "U" (unknown stage) category for the model. These are computed using solutions to the ODEs for the given set of parameters aggregated across a year.

Detections relate to the transfer from infectious categories to the recovered category – the new annual reported case incidence. This is either by passive detection from stage 1 for year Y:

$$P_{M1}(Y) = \int_Y^{Y+1} \eta_H(Y)\big(I_{1H1}(t) + I_{1H4}(t)\big)\,\mathrm{d}t,$$

passive detection from stage 2

$$P_{M2}(Y) = \int_Y^{Y+1} (\gamma_H(Y) - \text{Death rate})\big((I_{2H1}(t) + I_{2H4}(t)\big)\,\mathrm{d}t,$$

or by AS from the low-risk ($H1$) group in year Y

$$A_{M1}(Y) = z(Y)\text{Sens}I_{1H1}(Y) + z(Y)(1 - \text{Spec})\big(k_1 N_H - I_{1H1}(Y) - I_{2H1}(Y)\big)$$

and

$$A_{M2}(Y) = z(Y) \times \text{Sens} \times I_{2H1}(Y)$$

with variable AS coverage by year, $z(Y)$ and fixed diagnostic sensitivity. A_{M1} also contains any false positives that may have been incorrectly identified from non-infected people based on the high but imperfect specificity of the AS algorithm. We assume in the DRC that all false positives would be assigned to be stage 1 and treated, however, in the model false positives stay in the susceptible category, unlike true cases which move to the recovered category.

The log-likelihood function used in the adaptive Metropolis-Hastings MCMC contained two terms in each year for which reported case numbers were available for each source of reported cases (active or PS). These were:

- a beta-binomial probability that the total number of cases reported in that year for that source came from the available population (either the reported number of people actively screened for AS or the health zone population for PS) with probability calculated from solving the ODE for the current set of parameters, and
- a binomial probability that reported stage 1 cases come from the total number of reported staged cases, where the probability parameter again comes from the solution of the ODE. In many years, staging is unknown, so this part of the log-likelihood will return zero and not contribute to our calculation. In some years, we have only partially known staging information.

This formulation allowed over-dispersion in the observed cases to be included, via the beta-binomial distribution, and any proportion of cases with reported disease stage to be appropriately accounted for (assuming that the reporting of staging information is independent of the disease stage). The log-likelihood function was as follows:

$$\begin{aligned} LL(\theta|x) =\ & \log(P(x|\theta)) \\ \propto & \sum_{i=2000}^{2016} \Bigg(\log\left[\mathrm{BetaBin}\left(A_{D1}(i) + A_{D2}(i) + A_{DU}(i); z(i), \frac{A_{M1}(i) + A_{M2}(i)}{z(i)}, \mathrm{disp}_{\mathrm{act}}\right)\right] \\ & + \log\left[\mathrm{Bin}\left(A_{D1}(i); A_{D1}(i) + A_{D2}(i), \frac{A_{M1}(i)}{A_{M1}(i) + A_{M2}(i)}\right)\right] \\ & + \log\left[\mathrm{BetaBin}\left(P_{D1}(i) + P_{D2}(i) + P_{DU}(i); N_H, \frac{P_{M1}(i) + P_{M2}(i)}{N_H}, \mathrm{disp}_{\mathrm{pass}}\right)\right] \\ & + \log\left[\mathrm{Bin}\left(P_{D1}(i); P_{D1}(i) + P_{D2}(i), \frac{P_{M1}(i)}{P_{M1}(i) + P_{M2}(i)}\right)\right] \Bigg) \end{aligned}$$

The model takes parameterisation θ, x is the data, $P_{Dj}(i)$ and $A_{Dj}(i)$ are the number of cases detected by passive or AS (of stage j, which may be 1, 2 or unknown, U) in a year i of the data. $P_{Mj}(i)$ and $A_{Mj}(i)$ are the number of cases detected by passive or AS (of stage j) in year i of the model, and $z(i)$ is the number of people actively screened in year i. $\mathrm{BetaBin}(m; n, p, \rho)$ gives the probability of obtaining m successes out of n trials with probability p and overdispersion parameter ρ. The overdispersion accounts for a larger variance than under the binomial. The probability density function of this distribution is given by:

$$\mathrm{BetaBin}(m; n, p, \rho) = \frac{\Gamma(n+1)\Gamma(m+a)\Gamma(n-m+b)\Gamma(a+b)}{\Gamma(n-m+1)\Gamma(n+a+b)\Gamma(a)\Gamma(b)}$$

where $a = p(1/\rho - 1)$ and $b = a(1-p)/p$.

Missing active screening numbers

There are instances in the data where the number of cases from within year t ($A_D(t) = A_{D1} + A_{D2}$) is not consistent with the number of people recorded as having been screened in that year for that health zone ($z(t)$), i.e. (i) $A_D(t) > z(t)$ or (ii) $A_D(t)/z(t)$ is a much higher prevalence than is biologically realistic for gHAT (e.g. more than 10%).

In the previous DRC modelling studies [1, 2], we did not take any action concerning the very high prevalence in some years within a health zone. Instead, we considered only two scenarios:

1. If $A_D(t) < 20$ and $z(t) \leq A_D(t)$ we assumed that these people attended a screening outside their home health zone and that the record has been allocated correctly to their home health zone. in this case we set $z(t) = A_D(t)$.
2. Where $z(t) = 0$ and $A_D(t) > 0$, then we imputed the number of negative tests.

In the current study, we have chosen to impute the number of negative tests in more situations. We still believe that where the number of cases reported is low these people probably attended screening elsewhere, but imputing a missing screening value, in our independent health zone analyses, is expected to reflect the model's underlying prevalence better. We, therefore, imputed the number of negative tests for a year within a health zone where:

1. the number of cases from was more than 10% of the number screened ($A_D(t) > 0.1 \times z(t)$;
2. the number screened was zero, or not recorded, or less than 20 ($z(t) \leq 20$).

Imputation of the number of negative tests takes place within the MCMC fitting procedure.

We use a Geometric prior for the number of negative screening tests in year t, $A_D^-(t) \sim \text{Geom}(\lambda_t)$, where $\lambda_t = \frac{1}{1+\bar{N}_t}$ and $\bar{N}_t = \frac{\sum_{j=2000, j\neq t}^{2020} N_j e^{-|t-j|}}{\sum_{j=2000, j\neq t}^{2020} e^{-|t-j|}}$, a weighted mean of the number of people screened in years other than t. The proposal distribution for $A_D^-(t)$ was a negative binomial distribution:

$$A_D^-(t)|A_D(t), p(t) \sim \text{NB}(A_D(t) + 1, 1 - (1 - p(t))(1 - \lambda_t))$$

where the probability of active case detection, $p(t)$, was sampled from the following Beta distribution:

$$p(t)|\theta \sim \text{Beta}\left(\hat{p}(t)\left(\frac{1}{\text{disp}_{\text{act}}(t)} - 1\right), (1 - \hat{p}(t))\left(\frac{1}{\text{disp}_{\text{act}}(t)} - 1\right)\right)$$

and $\hat{p}(t)$ is the probability of active case detection in year t calculated from the ODE outputs.

S1.6 Projections

↩ Return to the Supplement Table of Contents

Samples and future projections were run using a stochastic model analogous to the deterministic model used during the MCMC fitting. This approach allowed us to avoid the computational expense of fitting a stochastic model (e.g. via particle filter MCMC or approximate Bayesian computation), but still gain the advantages of stochastic model outputs, in particular being able to directly assess the model when elimination of transmission or elimination of infection has occurred. This is not possible in the deterministic framework without using a proxy threshold [5, 31]. Recent work by Davis et al. has demonstrated the very good alignment between posteriors from deterministic MCMC and stochastic pMCMC fitting of this gHAT model [32].

Model projections were carried out by taking 2,000 random samples from the joint posterior distribution of the model parameters, using these to simulate the stochastic model 10 times for each sample from the joint posterior distribution of the model parameters with observational samples for case reporting using the beta-binomial distributions described earlier. The stochastic model covered the period for which data were fitted (2000 to 2020) as well as projecting into the future under various intervention strategies (see Section S1.7.2).

Ensemble model

The results from the stochastic projections for the two model variants were combined into an ensemble model, with the proportion taken from each model based on the relative model evidence. The model evidence, or marginal likelihood, for each model, was estimated using the importance sampled estimator [33].

The following description of the important sampling method to estimate the model evidence has been adapted slightly from that in the Supplementary Information for Crump et al. [2].

The joint distribution of $(\boldsymbol{\theta}_m, \mathbf{x})$, for parameters $\boldsymbol{\theta}_m = (\theta_1, \theta_2, \ldots, \theta_{d_m})$ of model m and data $\mathbf{x} = (x_1, x_2, \ldots, x_n)$ satisfies

$$\pi(\boldsymbol{\theta}_m|\mathbf{x})\,\pi(\mathbf{x}|\mathbf{m}) = \pi(\mathbf{x}|\boldsymbol{\theta}_m)\,\pi(\boldsymbol{\theta}_m)\,, \tag{7}$$

where $\pi(\boldsymbol{\theta}_m|\mathbf{x})$ is the joint posterior distribution of parameters $1 \ldots d$, $\pi(\mathbf{x}|\mathbf{m})$ is the marginal likelihood or *evidence*; $\pi(\mathbf{x}|\boldsymbol{\theta}_m)$ is the likelihood, and $\pi(\boldsymbol{\theta}_m)$ is the prior distribution.

By use of MCMC methods to investigate the posterior distribution of the parameters, calculation of $\pi(\mathbf{x}|\mathbf{m})$ is avoided. Calculation of the evidence for use in model comparison requires computing the integral:

$$\pi(\mathbf{x}|\mathbf{m}) = \int \pi(\mathbf{x}|\boldsymbol{\theta}_m)\,\pi(\boldsymbol{\theta}_m)\ \mathrm{d}\boldsymbol{\theta}_m \tag{8}$$

$$= \int \pi(\mathbf{x}|\boldsymbol{\theta}_m)\,\frac{\pi(\boldsymbol{\theta}_m)}{q(\boldsymbol{\theta}_m)} q(\boldsymbol{\theta}_m)\ \mathrm{d}\boldsymbol{\theta}_m \tag{9}$$

Equation 8 cannot be calculated analytically except for some small set of tractable models. It can, however, be rewritten as equation 9, where $q(\boldsymbol{\theta}_m)$ is a d_m-dimensional probability density function. From this, an importance sampled estimator of $\pi(\mathbf{x}|\mathbf{m})$ is:

$$\hat{P}_q = \frac{1}{N}\sum_{i=1}^{N} \pi\left(\mathbf{x}|\boldsymbol{\theta}_{m,i}\right) \frac{\pi\left(\boldsymbol{\theta}_{m,i}\right)}{q\left(\boldsymbol{\theta}_{m,i}\right)}, \tag{10}$$

where the $\boldsymbol{\theta}_{m,i}$ are N samples drawn from q.

A defence mixture [34] was used for $q(\boldsymbol{\theta}_m)$:

$$q(\boldsymbol{\theta}_m) = p\phi\left(\boldsymbol{\theta}^*_m; n, \boldsymbol{\mu}_1 \ldots \boldsymbol{\mu}_n, \mathbf{C}_1 \ldots \mathbf{C}_n\right) \left|\frac{\boldsymbol{\theta}^*_m}{\boldsymbol{\theta}_m}\right| + (1-p)\,\pi(\boldsymbol{\theta}_m) \tag{11}$$

where $\phi(\cdot)$ is a mixture of n multivariate Gaussian distributions with vectors of means $\boldsymbol{\mu}_j$ ($j = \{1 \ldots n\}$), and covariance matrices $\mathbf{C}_j$, $\left|\frac{\boldsymbol{\theta}^*_m}{\boldsymbol{\theta}_m}\right|$ is the Jacobian transformation relating probability on transformed and original scales, and p is a mixing proportion ($p = 0.95$ was chosen for use, being a typical value [33]).

In each of our health-zone-level MCMC analyses of the models with and without animal transmission, $2\,000$ samples from the joint posterior distribution were generated and stored, and $\phi\big(\boldsymbol{\theta}_m; n, \boldsymbol{\mu}_1 \ldots \boldsymbol{\mu}_n, \mathbf{C}_1 \ldots \mathbf{C}_n\big)$ for each health zone and model was chosen using the Matlab `fitgmdist` function, selecting n based on Akaike's Information Criterion (AIC). To account for the high correlations between some of our model parameters, regularisation was applied to ensure that the covariance matrices, $\mathbf{C}_k$, would be positive semi-definite. Before passing to `fitgmdist`, transformations were applied to the posterior samples to put them in the range $(-\infty, \infty)$ – appropriate for Gaussian distributions – followed by scaling and centring to keep the regularisation consistent across analyses, at least at the simple, single overall covariance matrix level.

Having defined $\phi(\cdot)$ for a given analysis (health zone, model combination), $\hat{P}_q$ was calculated (equation 10) using $N = 10\,000$ samples drawn from $q(\boldsymbol{\theta}_m)$. Note that this is an increase from the $2\,000$ samples used previously [2], and is expected to reduce the possible influence of sampling on the model evidence results.

S1.7 Strategies and interventions

↩ Return to the Supplement Table of Contents

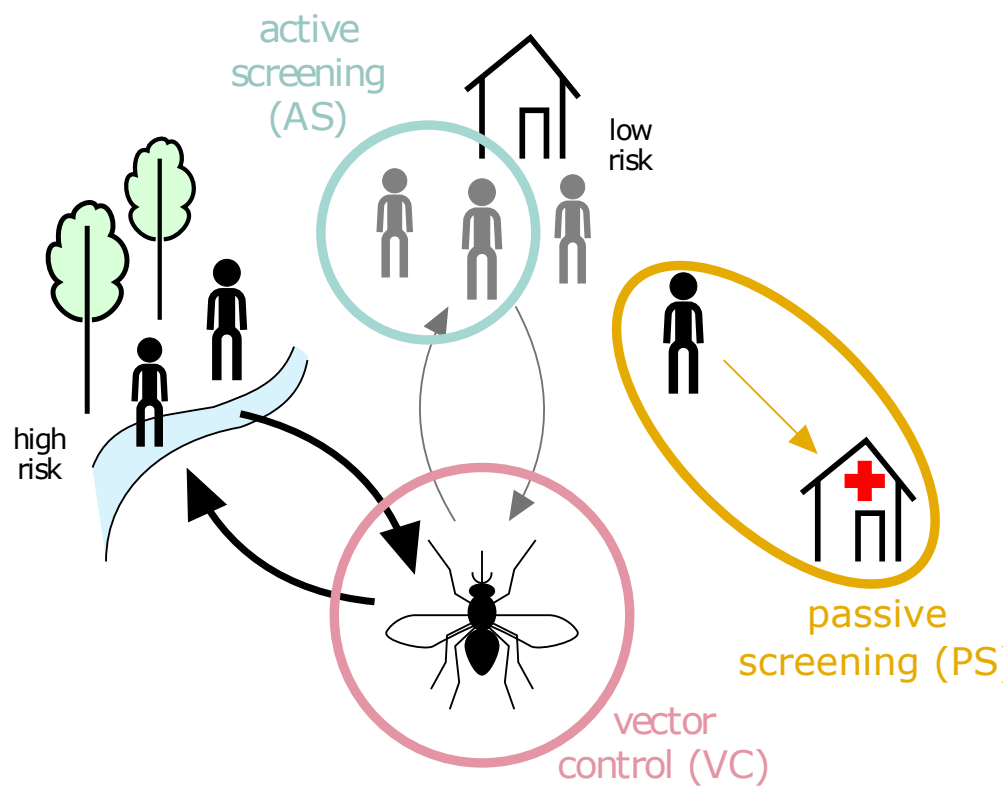

Fig C: Core gHAT intervention toolbox. Abbreviations: AS: active screening, PS: passive screening, RS: reactive screening, VC: vector control. Adapted from Rock et al. [35] under a CC-BY license.

Intervention	Description
**Active screening (AS)**	Mobile teams travelling to at-risk villages to test any person willing to participate
**High risk**	Individuals with greatest risk of gHAT infection
**Intensified (Int.) active screening**	Screening coverage (% people) at either the historic maximum or at 30% if the historic maximum is lower than this value
**Intervention**	Tools, treatments or approaches used to prevent or treat the infection
**Low risk**	Individuals with lowest risk of gHAT infection
**Mean active screening**	Screening coverage (% people) at the mean of the last five years for a region
**Passive screening (PS)**	Testing self-presenting individuals for gHAT at fixed health facilities
**Reactive screening (RS)**	Testing in specific locations in response to cases detected through passive screening
**Treatment**	Treatment of confirmed cases with either fexinidazole (oral drug course) if eligible, or pentamidine or NECT. Acoziborole (oral single-dose cure) may be used in the future if approved but is not considered in this analysis.
**Vector control (VC)**	Methods used to reduce the abundance of the vector, i.e. tsetse, that transmit infection.
**Targeted vector control (VC)**	An adapted method based on that previously used by LSTM to identify areas with high case density at which to focus Tiny Target deployment efforts along large rivers.
**Full Vector control (VC)**	Considers the deployment of Tiny Targets throughout all large rivers in a health zone.

Table H: Intervention components which make up key strategies

S1.7.1 Interventions

The definition of the interventions is shown in Table H and depicted in Fig C. A summary of the levels of each of the interventions are in Table I.

Active Screening

Historically, AS has been the flagship activity of gHAT control, and therefore we simulate AS at two coverage levels: *Mean AS* which is the average coverage of the most recent five years in the data (i.e. 2016–2020), and *Intensified AS* which is the higher of the historical maximum (in 2000–2020) or 30% of the health zone. This *Int. AS* option was selected because the historical maximum is quite low in many health zones (less than 5% of people screened in 45 analysed health zones).

Two levels of active screening coverage are considered in our strategy sets. They are *Mean AS* and *Int. AS* in the general set, and *No AS* (sometimes skipped in the strategy names) and *Int. AS* for the special set for the Bas Uélé region. *Mean AS* represents maintaining AS at a recent level, we used the average numbers of people screened between 2016–2020. *Int. AS* refers to the realistic maximum level of AS that can be achieved in each health zone. For health zones outside the Bas Uélé region, it was assumed to be the higher of the historical maximum (in 2000–2020) or 30% of the health zone population in 2018. For health zones inside the Bas Uélé region, the *Int. AS* is assumed to be the average number of people screened between 2000–2014 because of the absence of recent HAT control activities. The values of *Mean AS* and *Int. AS* are calculated individually in each health zone. Finally, *No AS* means no activity at all in the future.

Although AS is organised at the coordination level, these units are mobile, require limited start-up costs to expand, can be scaled back, and often serve more than one health zone, so considering the resource use by the health zone will accurately reflect the collective costs at the coordination level.

Cessation of active screening. The planning of AS in the DRC is done by the end of the year and follows the WHO guidelines. AS should continue at the village level until 3 consecutive years of no case detection, and then pause for 1 year before a final screening in the fifth year. In other words, this is a two-step cessation algorithm for AS. The first step happens after no case reporting for 3 years, and the second step happens after no case reporting for 5 years. In our simulations, we assumed both steps occur in the future. To ensure *Mean AS* is a distinct strategy from *Int. AS*, we further assumed the earliest possible year for the first step cessation is 2026 and 2027 for strategies with mean and intensified coverage respectively.

Reactive screening to case detections after cessation. Reactive screening (RS) is the screening activities that react to any passive cases reporting after AS ceases. Its cessation also follows the same WHO guidelines explained above. Therefore, RS has a minimum length of 5 years (with 4 screening activities), but the actual duration depends on case finding.

Passive Screening

PS is simulated to continue at estimated 2020 levels into the future; this level reflects some improvements over the period of 2000-2020, estimated by model fitting. We do not include additional future improvements to the PS system. We used the 2019 WHO survey of clinics to calculate the cost of PS with two exceptions: for coordinations Bandundu Nord and Bandundu Sud. In those coordinations, in the provinces of Mai-Ndombe, Kwilu, and Kwango, there were ongoing trials at the time to expand the network of health posts that would have RDTs available for serological diagnosis of HAT. Patients would then be referred to the health zone's health centre for confirmation, staging (when necessary) and treatment [28]. However, that programme has since ceased, and most of those clinics are no longer getting support. Therefore, we have used the number of PS clinics that were listed for Bandundu Nord and Sud in the survey that was published for the year 2012 [36].

Passive screening, including post-elimination surveillance. Although we did not consider cessation of PS in the transmission model, we assumed that PS scales back to one health centre per health zone in the cost model after 5 years of no case reporting and this assumed post-elimination surveillance is equally effective as current PS (ignoring the possible impact of losing skilled clinicians and population awareness as the disease becomes rare).

Future VC

VC activities have been shown to decrease both tsetse populations and case reports in other locations [26, 37, 38], and therefore we simulate the addition of VC activities at two levels. In this analysis, any strategies including *Targeted VC* use an adapted algorithm based on that previously used by the Liverpool School of Tropical Medicine (LSTM) to identify areas with high case density at which to focus Tiny Target deployment efforts along large rivers. Strategies including *Full VC*, by contrast, involve a substantial expansion of VC interventions, considering the deployment of Tiny Targets throughout all large rivers in a health zone.

For our future strategies, we consider three possible levels of VC: *No VC*, *Targeted VC* and *Full VC*. The *Full VC* strategy consists of treating the banks of all candidate rivers in a health zone with tiny targets. Candidate rivers are either “large rivers", which we defined as those with an average long-term discharge estimate for river reach of $> 20m^3/s$, or any other rivers where VC has historically been deployed.

To select rivers for the *Targeted VC* strategy, we use a similar method to our coverage calculations, buffering each river segment by 5 km in every direction and taking the intersection of this buffer and the health zone in question. In all areas other than Bas Uélé, we then take all geolocated cases from 2016–2020 and intersect them with these buffered river segment areas. The number of cases falling in each buffered area is then counted; any cases that fall into multiple health buffers are split fractionally and equally between them so that they are counted as one case in total. We count "river length to be treated" as any river segment with a case density of at least 1 case per 10 km of treated riverbank. In the Bas Uélé region, we instead use cases from 2010 to 2014 since there was no capacity for detecting cases in this region between 2016–2020.

If our *Targeted VC* and *Full VC* scenarios have the same length of riverbank and case coverage, and VC has historically been deployed in the area, then instead of using the above calculations for *Targeted VC*, we use the maximum historical deployment length and case coverage of VC.

To compute the case coverage for the *Targeted VC* and *Full VC* strategies, we select the relevant rivers as above, then split them at any junctions, and prune any dead-end segments of less than 10 km. The rivers are then recombined and split again at health zone boundaries or any junctions between remaining river segments.

To calculate the length of the riverbank treated for each river segment, we apply the following steps. If a segment falls entirely along or within 5km of a health zone boundary (inside or outside), we treat it as if one bank (the interior bank) is being treated. If a segment falls entirely on the interior of the health zone (more than 5km from the boundary), we count it as having both banks treated. If it lies both near a boundary and also on the interior of the health zone, we count both banks on the portion in the interior, and one bank in the portion near the boundary. We then sum the length of the banks as above to estimate the total length of the riverbank where control is deployed under the strategy.

Cessation of vector control. Cessation of vertical interventions (which include vector control) was modelled as stopping based on consecutive years of zero detected cases by any screening modality. For VC, we used three years of no case detections (without the requirement for the fifth year of activity like with AS). Reactive VC is not considered in our simulations.

In addition to the criterion based on case reporting, we also considered a minimum of 3 years of VC to reflect the operational constraints such as training new people and sensitization to reach its maximum impact. In other words, we assume VC cessation can occur when there are no reported cases for three consecutive years. As the earliest possible year to scale back VC varies depending on the history of VC in individual health zones, our simulations do not allow cessation to happen before the analytical present and earlier than 2025 in order to ensure strategies with VC in the name have at least 1 year of VC. In our simulations, we checked simulated case numbers by the end of each year to decide whether the cessation criterion has been met by 2025 (for health zones with VC already, listed in Table G) or 2028 (for rest health zones). We assumed VC will stop from the following year if the cessation criterion is met and no reactive VC will take place to react to any simulated case reporting after scaling back.

Treatment

Historically, gHAT treatment could take place only in these referral hospitals and in many cases, the only health facilities where one would self-report for diagnosis with serological and parasitological tests and staging of the disease. In 2020, with the availability of the simpler oral treatment of fexinidazole treatment could be administered within the health areas or villages where the individual cases live for those individuals that do not need hospitalization according to recommendations [39].

In this analysis, fexinidazole is simulated as being available immediately, but acoziborole – which is not yet approved for use outside clinical trials – is not included in this analysis. Further details on the treatment classifications and costs are described in Section S1.10.4.

Coordination	PS clinics	Sum PS clinics	Sum PS clinics, in analysis	AS mean	AS historical maximum	AS total (thou-sands)	VC bank targeted (km)	VC bank full (km)
Bandundu Nord	8 [2-21]	169	54	0.18 [0-0.45]	0.31 [0.02-0.64]	621	83 [0-409]	279 [0-916]
Bandundu Sud	5 [0-25]	111	39	0.09 [0-0.61]	0.17 [0-0.74]	699	11 [0-417]	272 [26-483]
Equateur Nord	1 [0-4]	28	34	0.02 [0-0.12]	0.22 [0.05-0.41]	186	0 [0-0]	161 [0-394]
Equateur Sud	1 [0-4]	12	13	0.01 [0-0.06]	0.1 [0-0.2]	22	0 [0-15]	181 [25-892]
Isangi - Bas-Uélé	0 [0-0]	0	2	0 [0-0]	0.21 [0.21-0.22]	0	377 [0-753]	890 [320-1460]
Isangi - Tschopo	2 [0-11]	16	17	0.09 [0.01-0.11]	0.17 [0.04-0.21]	61	108 [0-142]	237 [197-300]
Kasai Occidental	1 [0-3]	17	23	0.02 [0-0.17]	0.06 [0-0.23]	143	0 [0-113]	185 [0-639]
Kasai Oriental	2 [0-8]	54	57	0.02 [0-0.13]	0.05 [0.02-0.23]	182	0 [0-89]	94 [0-329]
Kinshasa	2 [0-6]	24	28	0 [0-0.07]	0.01 [0-0.1]	37	11 [0-69]	21 [11-542]
Kongo Central	4 [0-11]	81	82	0.01 [0-0.12]	0.05 [0.01-0.27]	53	0 [0-25]	72 [0-379]
Maniema Katanga	1 [0-2]	9	14	0.02 [0-0.08]	0.06 [0-0.12]	94	0 [0-144]	235 [0-628]
Sankuru	1 [0-3]	10	12	0.03 [0-0.23]	0.08 [0-0.27]	64	0 [0-190]	258 [94-564]
**Total**	**2 [0-25]**	**531**	**375**	**0.02 [0-0.61]**	**0.09 [0-0.74]**	**2162**	**0 [0-753]**	**175 [0-1460]**

Table I: Summary of the screening and vector control activities in health zones in the analysis, stratified by the coordinations delineated by the programme national de lutte contre la Trypanosomiase humaine africaine (PNLTHA-RDC). Distributions are the median followed by the minimum and maximum values for health zones. The number of PS clinics in the analysis and the number in the 2019 WHO survey differ for two reasons. First, in Bandundu Nord and Bandundu Sud, TrypElim had expanded the network of clinics with capacity for serological confirmation followed by a referral to the main hospital, but the referral system did not work as most patients were lost-to-follow-up. For more information, see Snijders et. al [28]. Therefore, the clinics we assume can still diagnose and treat patients were equivalent to the clinics reported before the TrypElim project in Bandundu: 54 clinics in Bandundu Nord and 40 clinics in Bandundu Sud [36]. Second, we have assumed all health zones have at least one clinic that can screen for HAT with an RDT or a CATT test; even if the clinic is not present in that health zone there is a clinic in a nearby health zone committing resources to screen patients from the health zone in question. Abbreviations: HZ: health zone, PS: passive screening, AS: active screening, VC: vector control.

S1.7.2 Strategies

There are two sets of strategies considered from the analytical present (i.e. from 2026 onward) for the special set for health zones in the Bas Uélé region and the general set for the rest of the analysed health zones (see Figure 1(b) and 1(c) in the main text). The Bas Uélé region had a very distinct strategy history (i.e. no HAT control activities such as active and PS since MSF left in 2015) due to operational feasibility, and therefore the general strategies are not suitable nor feasible for health zones in the Bas Uélé region.

The strategies are listed in Fig 1B for 163 health zones, and in Fig 1C for the two health zones of Bas Uélé region that required different strategies. The health zones are characterized by six typologies, which determine the strategies simulated and the comparator:

Category	Strategies	Comparator	No. HZ
1	All six	*Mean AS*	25
2	All six	*Mean AS + Targeted VC*	12
3	No Targeted VC	*Mean AS*	116
4	No VC	*Mean AS*	10
5	Alternative eight	*No AS, PS, nor VC*	1
6	Alternative six	*No AS, PS, nor VC*	1

Six typologies of health zones, depending on the strategies simulated and the comparator. Abbreviations: AS: active screening, PS: passive screening, VC: vector control.

1. **All six strategies, *Mean AS* is the comparator.** Health zones where there is no current VC but where there is potential for targeted VC based on recent case clustering near large rivers have a status quo strategy of *Mean AS* which includes PS in fixed health facilities and mobile screening activities that screen a number of the population equal to the mean number screened in AS in 2016–2020. One of these health zones, Bolobo, had VC in the recent past but has no ongoing VC. The five additional strategies are made up of different combinations of AS and VC – *Int. AS*, *Mean AS* + *Targeted VC*, *Int. AS* + *Targeted VC*, *Mean AS* + *Full VC*, and *Intensified AS* + *Full VC* – are simulated to compare the health benefits and the costs against this status quo strategy. The number of health zones in this category is 25.
2. **All six strategies, *Mean AS* + *Targeted VC* is the comparator.** Health zones where there is ongoing VC, will have a status quo strategy of *Mean AS* + *Targeted VC* but we still run the six strategies. The *Mean AS* and *Intensified AS* strategies would imply cessation of the current VC activities. Health zones with historical and ongoing deployments are Bagata, Bandundu, Bokoro, Bolobo, Kwamouth and Yumbi in Bandundu Nord and Bulungu, Kimputu Masi Manimba, Mokala in Bandundu Sud. Yasa Bonga in Bandundu Sud has had historical VC but the extent has approximated Full VC, so the comparator is *Mean AS* + *Full VC*. Kikongo started as VC in mid-2019 and switched to 'community' deployment in 2022, but we ran it as if nothing had changed. The total number of health zones in this category is 12.
3. **Four strategies; no Targeted VC strategies.** In health zones where there is no clear way to perform targeted VC, as the recent cases are not sufficiently geographically clustered– but where there are large rivers that have a status quo strategy equivalent to *Mean AS*. We still simulate VC along all large rivers and so the three additional strategies are: *Intensified AS*, *Mean AS* + *Full VC*, and *Intensified AS* + *Full VC*. The number of health zones in this category is 116.
4. **Two strategies; no VC strategies.** For health zones where no major rivers were detected during the analysis, we only simulated the two strategies that exclude VC. *Mean AS* is the comparator. These health zones were: Ntand Embelo in Bandundu Nord; Gemena and Tandala in Équateur Nord; Katende in Kasaï Occidental; Kabinda, Mpokolo, and Nzaba in Kasaï Orientale; Muanda and Seke Banza Kongo Central; and Mbulula in Maniema-Katanga for a total of 10 health zones.
5. **Bas Uélé region; eight and six alternative strategies.** Health zones in the Bas Uélé region including Ango and Doruma have an alternative set of strategies as shown in Fig 1C. These health zones have an alternative set of strategies because activities there were formerly run by an international organization, Médecins sans frontières (MSF), rather than under the purview of the national programme. MSF ran screening with alternative diagnostic algorithms [3] and therefore the data are interpreted separately. Since the departure of MSF, due to challenges in access, the national programme has not had the opportunity to run activities there, and

therefore, there is less data and more uncertainty about transmission in this region. As a result, we model activities that would keep the status quo (*No AS, PS, nor VC*) as the comparator, putting into place activities similar to the rest of the country, and conducting additional VC:

(a) in Ango: *Int. AS* + *no PS*, *Restart PS*, *Int. AS* + *restart PS*, *Targeted VC* + *restart PS*, *Int. AS* + *Targeted VC* + *restart PS*, *Full VC* + *restart PS*, *Int. AS* + *Full VC* + *restart PS*.

(b) Doruma has cases that are so diffuse throughout the health zone that the algorithm to determine VC extents was not able to find any hotspots. Therefore, no Targeted VC strategies were simulated.

Note on the interpolation for the period of 2021-2025. For those years of 2021-2025, for which decision analysis does not make sense but for which we did not have data at the time of analysis, we do not assume alternative strategies. In other words, we assumed that PS and VC carried on as they were, and actively screened covered populations at the average level from 2016–2020 during the period between the end of the data period and the analytical present (i.e. in 2021–2025).

S1.8 Cost-effectiveness analysis

↩ Return to the Supplement Table of Contents

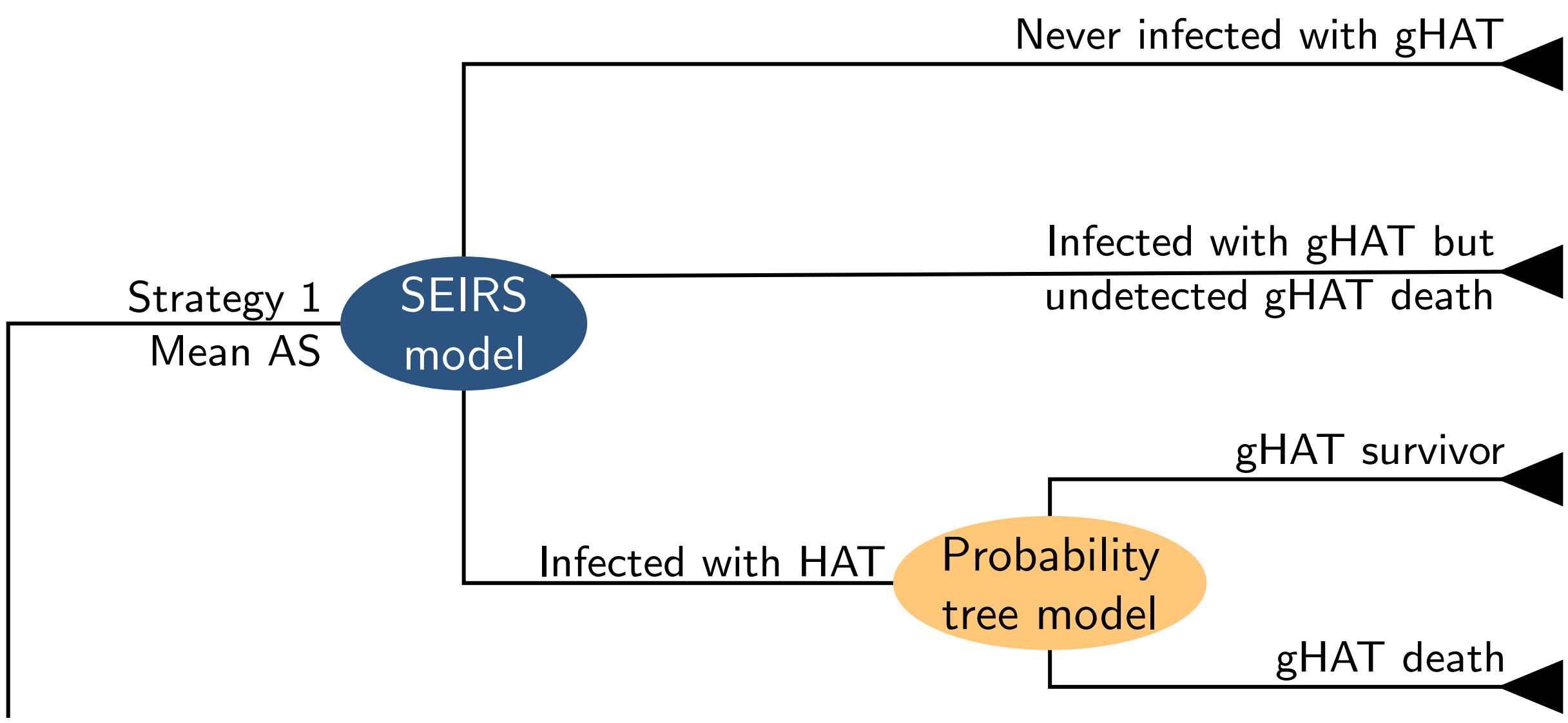

Fig D: Decision tree, close up of Fig E. Decision nodes are square, and probabilistic nodes are ellipses. The transmission model is depicted in Fig B and the probability tree model is depicted in Fig F. Abbreviations: SEIRS: susceptible-exposed-infected-recovered-susceptible dynamic model of transmission, AS: active screening, PS: passive screening, RS: reactive screening, VC: vector control.

Lastly, we calculate costs, treatment outcomes and disease burden to estimate the cost-effectiveness of each of the strategies according to a decision tree shown in Fig D and shown in more full in Fig E. The costs here are denominated in 2024 US$, and health burden and benefits are denominated in disability-adjusted life-years, although we also present cases and deaths as intermediate outcomes. We have selected to show costs, benefits, and cost-effectiveness taking into account a 2040 horizon to calculate the costs of activities until 2030, the goal date for elimination of transmission, and to be able to take into account any savings from scale-back of activities that may stretch into the decade following 2030.

(a) A) General set of strategies. **(b)** B) Special set of strategies (for Bas Uélé region).

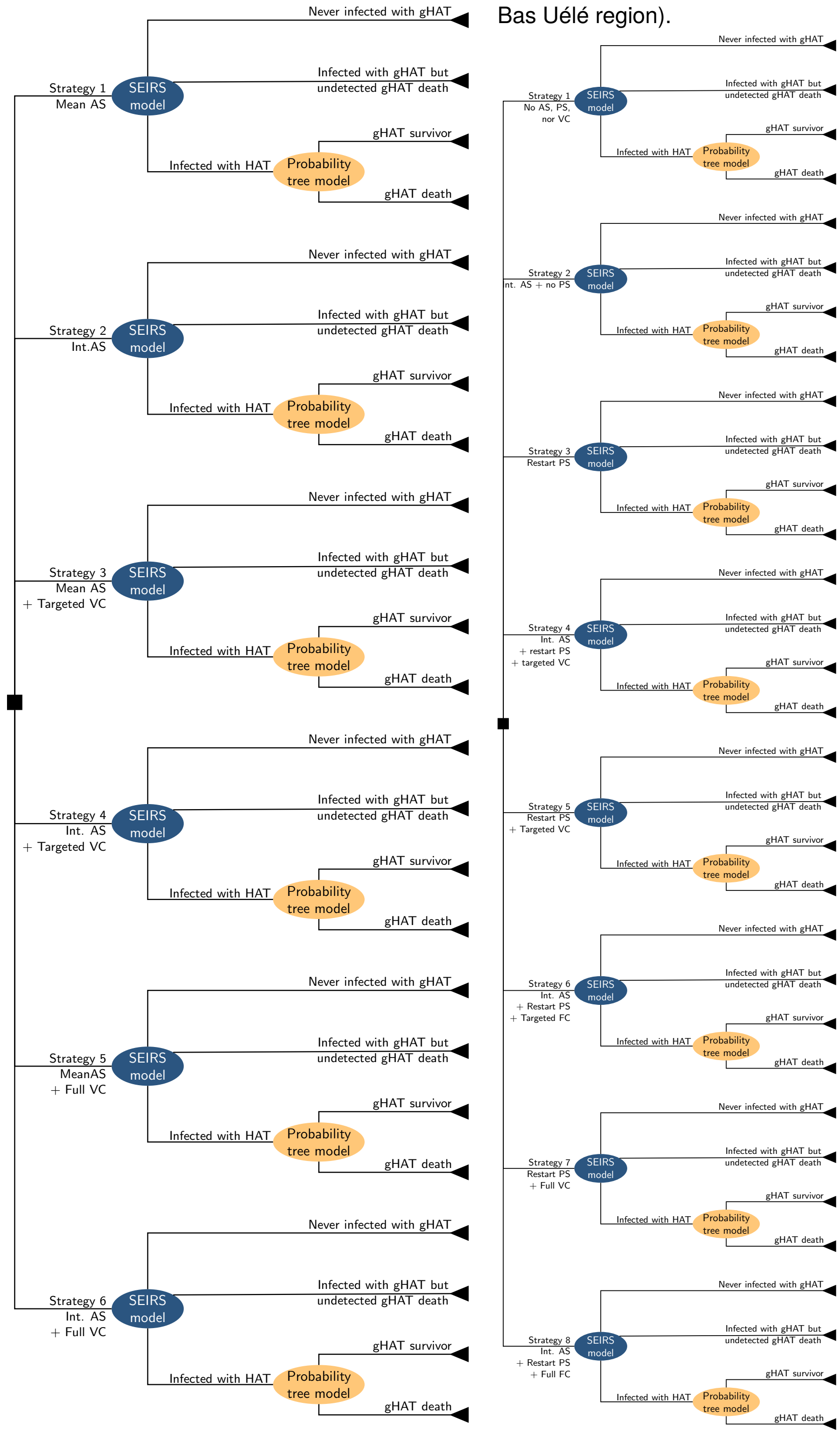

Fig E: Decision tree of disease suppression and prevention strategies. Decision nodes are square, and probabilistic nodes are ellipses. One branch (one strategy) is depicted in detail in Fig D. The transmission model is depicted in Fig B and the probability tree model is depicted in Fig F. A) Strategies against gHAT, including active screening (AS) by mobile teams, passive screening (PS) in fixed health facilities, and vector control (VC). In three strategies (*Mean AS*, *Mean AS + Targeted VC*, and *Mean AS + Full VC*) the proportion screened equalled the mean number screened during 2016–2020. In the three other strategies (*Int. AS*, *Int. AS + Targeted VC*, *Int. AS + Full VC*), the coverage is the maximum number screened during 2000–2020. In strategies 3–6, vector control (VC) is simulated assuming a reduction as described in the Supplementary Methods, Section S1.7.1. PS is in place under all strategies. B) Strategies against gHAT in Bas Uélé region. Abbreviations: SEIRS: susceptible-exposed-infected-recovered-susceptible dynamic model of transmission, AS: active screening, PS: passive screening, VC: vector control.

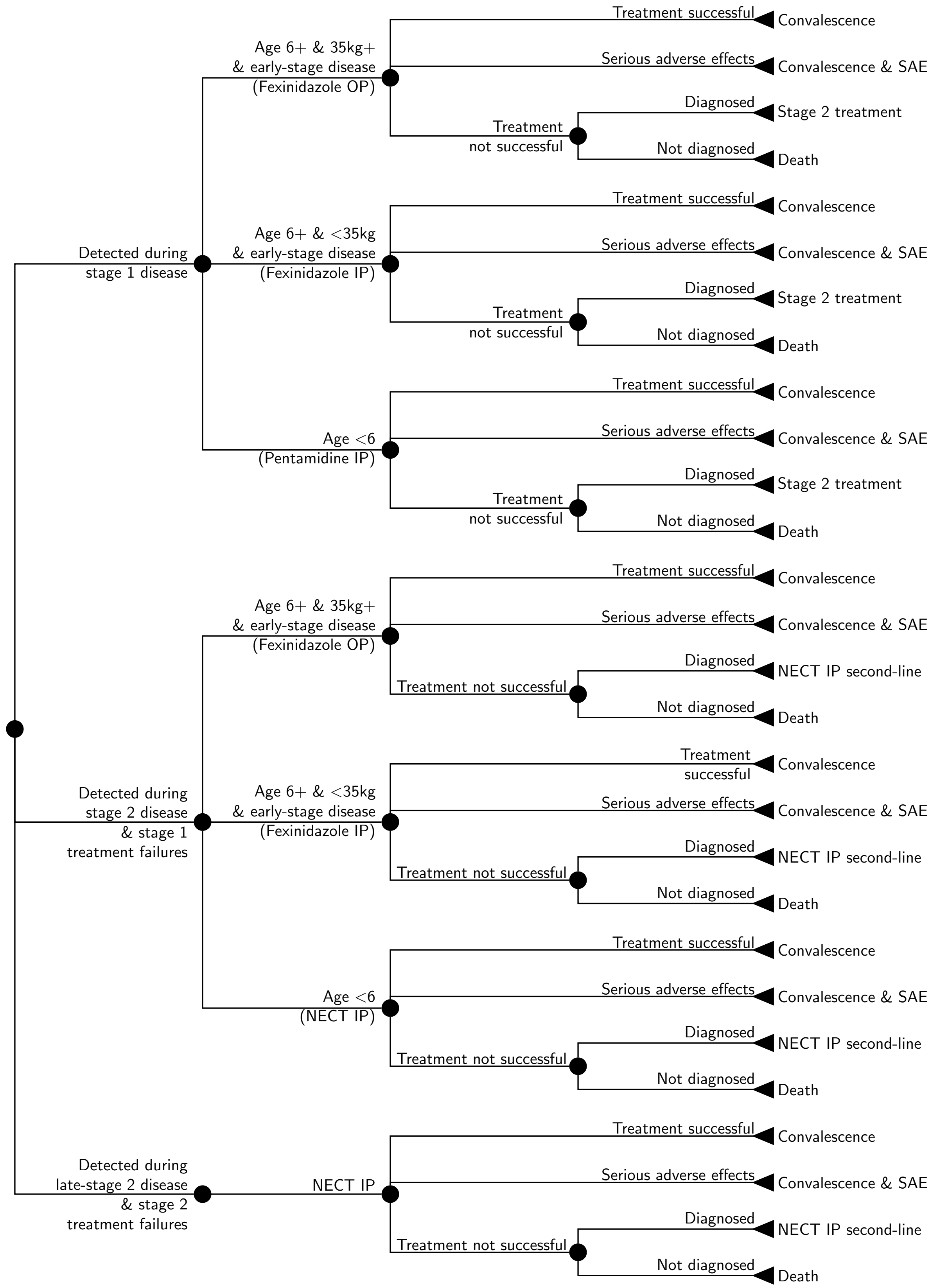

Fig F: Treatment model. Treatment for diagnosed gHAT patients is modelled as a branching tree process of possible health outcomes, including eligibility for novel Fexinidazole. Abbreviations: SAE: Serious adverse events, IP: inpatient care, OP: outpatient care, NECT: nifurtimox-eflornithine combination therapy.

S1.9 Health outcomes denominated as disability-adjusted life-years

↩ Return to the Supplement Table of Contents

(Please note that sections S1.9-S1.10.4 contain some amount of recycled text from the authors' previous publications [6, 27].)

For incremental cost-effectiveness calculation, we define the health effects of gHAT interventions in terms of disability-adjusted life-years (DALYs) [40, 41]. Using DALYs is in line with the recommendations of the Bill and Melinda Gates Foundation's reference case and WHO's guidelines for the conduct of cost-effectiveness analyses [40–42]. DALYs allow policy-makers to compare interventions across different disease programs with one common metric.

DALYs were discounted at a rate of 3% per year [41, 42]. We follow established conventions to calculate DALYs and evaluate the estimates in present-day terms (after applying discounting) [41–43].

A more general discussion of this is found in [6] (Supplementary Methods, pages 10-11), but we provide a brief description here for convenience.

$$\text{Disability-adjusted life-years (DALY)} = \text{Years of life lost to disability (YLD)} + \text{Years of life lost due to death (YLL)}$$

$$\text{YLD} = (\text{YLDs before detection} + \text{YLDs during treatment} + \text{YLDs due to side effects}) \times \text{DALY weight}$$

where the DALY weight is a metric to measure the relative severity of gHAT compared to living with other diseases.

$$\text{YLL} = \text{life-expectancy at age of death} - \text{age of death from gHAT}$$

One important difference between the method described in [6] and the current study is that here we use the life-table method of accounting to calculate the years of life that a person with a fatal case would have lived absent gHAT. The previous study used the life-of-years lived at birth to less the age at death. In 2019 (the most recent year with data from the WHO), the life expectancy in DRC was 62.4 and the age of death from gHAT is 26.62 (See Supplementary Note 3: Parameter Glossary, Section S4.6.15). The life expectancy at age 25-29 is about 70 in DRC (usually, life expectancy is higher in older ages after surviving the risky first few years of life). Therefore, the current method estimates a slightly higher number of YLLs, and by consequence, DALYs, than the previous method.

S1.10 Treatment outcomes and cost functions

↩ Return to the Supplement Table of Contents

The cost functions are described in this section separately for active screening, passive screening, vector control, and treatment. The health zone of Kikongo is used as an example to show intermediate calculations and final costs.

The total costs can be characterised by the expression

$$\text{Total costs} = \sum_{i \in \text{all sub-categories}} \left(U_i \times C_i \right)$$

i is the cost sub-category. Where U is the units of a resources(i.e. people screened, teams in operation, testing centres, etc) and C is the cost per resource unit. All costs were denominated in 2024 US$. The process to update costs from the literature is detailed in Supplementary Note 3: Parameter Glossary, Section S4.1.

S1.10.1 Active screening

Yearly costs of AS were calculated as a function of two groups of expenses: capital and management costs, as well as the costs of screening the population and confirming suspected cases. Because gHAT program activities are not combined with activities of any other disease programs, we employed a full costing, rather than an incremental costing, method. A more thorough explanation of active screening campaigns and their costs is available from Snijders and colleagues [44].

Notably, we do not include the costs of lumbar punctures in this portion of the analysis; rather, we include it in the costs of treatment. Because many patients are eligible for fexinidazole treatment, which does not require lumbar punctures, we include lumbar puncture costs in the treatment portion of the analysis for those patients who are not eligible for fexinidazole. See Section S1.10.4.

- Overhead costs: overhead costs are split between capital costs and recurrent (management) costs to run an active screening team. AS teams serve a “coordination”– a subnational designation of the PNLTHA program that manages a set of health zones. Therefore, a health zone where fewer than 60,000 people are targeted for screening does not necessarily incur larger overhead costs for active screening than a health zone where the coverage is closer to a multiple of the yearly capacity of a team.
  - Capital costs consist of vehicles, medical equipment, energy (solar panels) and training (which occurs once every few years).
  - Recurrent costs consist of management and consumables that are spent on the team: fuel, staff time, etc.
- Costs related to the population screened:
  - Card Agglutination Trypanosomiasis Tests (CATT) are scaled up according to the number of people that are screened per year, and a factor of wastage of CATT tests is included.
  - Confirmation tests are counted for all of those who are positive according to the CATT test, including false positives (which are estimated as 1-specificity of the test) and true positives, calculated by the dynamic model.
- For all costs, the national PNLTHA is assumed to consume resources, and there is a mark-up to account for the central management at the national program headquarters. We have made the same assumption as Snijders and colleagues [44] who estimate that PNLTHA management equals approximately 15% of costs.

The parameters for AS are described in Supplementary Note 3: Parameter Glossary, Section S4.5 and the cost parameters are described in Supplementary Note 3: Parameter Glossary, Section S4.8.

The cost for Kikongo health zone per year, given the number of people screened in that health zone under Mean and Intensified AS, is therefore:

Variable Name	Parameterization	Summary
AS coverage per year	Fixed	See Table I
Wastage factor for CATT administration in AS context	Beta(8, 92)	0.08 (0.04, 0.14)
CATT specificity	Beta(4523, 22)	1.00 (0.99, 1.00)
AS capital costs (annualized)	Gamma(820, 20.1)	16,525 (15,405, 17,693)
AS recurrent costs (annualized)	Gamma(4153.88, 17.21)	71,486 (69,309, 73,689)
Cost of CATT test	Gamma(1140, 0.0006897)	0.79 (0.74, 0.83)
Cost confirmation (microscopy)	Gamma(8.47, 1.71)	14.59 (6.49, 26.08)
Cost of delivery for CATT (markup)	Fixed, 0.10	0.10

Table J: Components of active screening costs

Item	Units (U)	Cost (C)
Capital (annualized)	AS coverage per year ÷ patients screened by a team	AS capital × (1+PNLTHA markup)
Management/recurrent expenses	AS coverage per year ÷ patients screened by a team	AS recurrent × (1+PNLTHA markup)
CATT testing (See Note 1)	AS coverage per year (traditional) × (1+wastage factor for CATT in AS context)	CATT × (1+delivery mark-up) × (1+PNLTHA markup)
Microscopy/confirmation	(1-CATT specificity) × (AS coverage per year × Population)	Microscopy × (1+PNLTHA markup)

[1] Ideally, CATT tests would be used for active screening and RDT tests would be used for passive screening because of the high wastage of CATT tests in the context of passive screening settings. We simulate screening costs as if screening in the context of AS takes place with CATT only.

Table K: Active screening: cost function. Abbreviations: AS: active screening, CATT: card agglutination trypanosomiasis test, PNLTHA: Programme National de Lutte contre la Trypanosomiase Humaine, the national control program housed at the Ministry of Health.

Item	Units (U)	Cost per unit (C)	Cost per category
**Mean AS**			
Capital (annualized)	0.74 (0.54, 1.07)	16,525 (15,405, 17,693)	12,245 (8,881, 17,789)
Recurrent expenses	0.74 (0.54, 1.07)	71,486 (69,309, 73,689)	52,973 (38,583, 76,864)
Microscopy	208 (131, 305)	14.59 (6.49, 26.08)	3,042 (1,173, 6,048)
CATT testing	46,479 (44,558, 49,057)	1.14 (1.04, 1.24)	46,478 (44,557, 49,056)
**Total**			**114,737 (96,617, 144,383)**
**Int. AS**			
Capital (annualized)	1.23 (0.90, 1.78)	16,525 (15,405, 17,693)	20,341 (14,753, 29,551)
Recurrent expenses	1.23 (0.90, 1.78)	71,486 (69,309, 73,689)	88,001 (64,096, 127,690)
Microscopy	346 (217, 506)	14.59 (6.49, 26.08)	5,053 (1,949, 10,048)
CATT testing	77,213 (74,021, 81,496)	1.14 (1.04, 1.24)	77,212 (74,021, 81,495)
**Total**			**190,607 (160,504, 239,856)**

Table L: Cost breakdown for active screening activities for 1 year in Kikongo health zone.

S1.10.2 Passive screening

The costs of passive screening each year were calculated as a function of two groups of expenses: 1) overhead costs, and 2) the number of consultations, screening and confirmation tests that are performed in any clinic in the health zone capable of performing a serological or confirmatory test for a person that comes in with suspected gHAT according to the 2019 WHO survey of clinics in DRC [45]. The process of passive screening and its costs is described in more detail by Snijders and colleagues [28].

- Overhead costs: overhead costs include capital costs and recurrent (management) costs to equip a health centre to perform serological screening for HAT and microscopic confirmation, as well as to keep personnel in the clinics trained. These costs are scaled by the number of health clinics in the health zone capable of doing serological testing for gHAT [45].
  - Capital costs consist of medical equipment, energy (solar panels) and training (which occurs periodically every few years). These costs are scaled by the number of facilities that can perform serological confirmation.
  - Recurrent/management costs consist of health zone and provincial management and supervision.
- Costs that scale by population served:
  - Rapid diagnostic tests (RDT) are scaled up according to the number of people that are screened per year, including a markup to account for the wastage of tests.
  - Confirmation tests are accounted for all of those who are positive according to the RDT test: both false positives (which are modelled as a factor equal to 1-specificity of the test) and the true positives outputted by the dynamic model.
  - Lumbar punctures are not depicted as part of the passive surveillance diagnosis costs but are included as part of the treatment costs. Because many patients are eligible for fexinidazole treatment, which does not require lumbar punctures, we include lumbar puncture costs in the treatment portion of the analysis for those patients who are not eligible for fexinidazole. See Supplementary Methods, Section S1.10.4.
- For all costs, there is a PNLTHA mark-up added for the central management at the national program headquarters. Snijders *et al* [44] estimate that PNLTHA management equals approximately 15% of costs, and we have made the same assumption.

The parameters for PS are described in Supplementary Note 3: Parameter Glossary, Section S4.5 and the cost parameters are described in Supplementary Note 3: Parameter Glossary, Section S4.8.

Variable Name	Parameterization	Summary
PS coverage per year per population per clinic	Fixed, by coordination, see S4.5.2. Gamma(55.53, 1.55) for Bandundu Sud.	85.96 (64.80, 110.05)
Wastage factor for RDT	Beta(1, 99)	0.01 (<0.01, 0.04)
RDT specificity	Beta(1134, 11)	0.99 (0.98, 1.00)
PS capital costs (annualized, per facility)	Gamma(8.475, 283.46)	2,400 (1,069, 4,251)
PS management costs (annualized, per health zone)	Gamma(8.475, 145)	1,228 (548, 2,169)
Cost of RDT test	Gamma(1140, 0.002205)	2.51 (2.37, 2.66)
Cost confirmation (microscopy)	Gamma(8.475, 1.71)	14.59 (6.49, 26.08)
Cost of delivery (markup)	1.10	1.10

Table M: Components of passive screening costs

The cost for each health zone per year, given the number of people screened per health zone and the number of health centres available for PS, is therefore:

Item	Units (U)	Cost (C)
Capital - clinics	Number of facilities capable of screening and confirmation within the focus	Capital costs (clinic) × (1+PNLTHA markup)
District management	Per district	District management costs × (1+PNLTHA markup)
OP visit	PS coverage per year per clinic × Clinics in the focus	OP visit cost × (1+PNLTHA markup)
RDT testing	PS coverage per year per clinic × Clinics in the focus × (1+wastage for RDT)	RDT × (1+delivery mark-up) × (1+PNLTHA markup)
Microscopy/confirmation (suspects first identified in screening-only sites)	(1-RDT specificity) × (PS coverage per year per clinic × Clinics in the focus)	Microscopy × (1+PNLTHA markup)

[1] We assume that all testing in passive screening is done with RDT tests. This is not always true, but the costs will be approximately similar if the testing is done with CATT.

Table N: Passive screening: cost function. Abbreviations: PS: passive screening, RDT: rapid diagnostic test, OP: outpatient, PNLTHA: Programme National de Lutte contre la Trypanosomiase Humaine, the national control program housed at the Ministry of Health.

Item	Units (U)	Cost per unit (C)	Cost per category
Capital - clinics	3	2,400 (1,069, 4,251)	7,199 (3,207, 12,754)
Management	1	1,228 (548, 2,169)	1,228 (548, 2,169)
OP visit	258 (194, 330)	3 (2, 4)	811 (475, 1,243)
RDT	261 (196, 333)	2.64 (2.49, 2.79)	687 (514, 887)
Microscopy for false positives	2.48 (1.16, 4.40)	14.59 (6.49, 26.08)	36.10 (11.83, 79.59)
**Total**			**9,961 (5,842, 15,572)**

Table O: Cost breakdown for passive screening activities for 1 year in Kikongo health zone. The same calculation were made and shown in Antillon et. al [6] and [27], but these calculations feature specific parameters for Kikongo (and every other health zone in this analysis) as well as costs updated to 2024 USD values. Abbreviations: RDT: rapid diagnostic test, OP: outpatient.

S1.10.3 Vector control

The costs of vector control (VC) were calculated as a function of two features: 1) the extent of the rivers where VC is deployed, and 2) the number of targets per kilometre of river where the targets were deployed. The costs of entomological surveys (tsetse monitoring), sensitisation of the population (information campaigns), and district management were assumed to scale with the extent of the health zone where VC would be deployed. The materials and labour time related to target deployment were scaled according to the number of targets deployed. A full costing method was used. A more detailed account of vector control in DRC is given in publications by Tirados and colleagues [30] and the costs are detailed in Snijders and colleagues' publication [46].

The extent of the riverbank that is necessary to cover for adequate coverage is determined by the case reports of the previous five years. The method is further explained in Section S1.7.1. Thirty targets per kilometre are assumed to be used, per current practice in DRC.

The parameters for VC are described in Supplementary Note 3: Parameter Glossary, Section S4.7 and the VC cost parameters are described in Supplementary Note 3: Parameter Glossary, Section S4.10.

Variable Name	Parameterization	Summary
Linear km of river bank	Fixed	Varies by health zone. See section S4.7.1. For Kikongo, it is 79 km for targeted VC and 601 km for full VC.
Units per km of river bank	Fixed	15
Deployments per year	Fixed	2
Cost for entomological surveys, sensitisation and district management per kilometer	Gamma(8.475, 57.7)	487 (216, 868)
Cost per target deployment per target	Gamma(8.475, 0.50)	4.23 (1.88, 7.49)
PNLTHA markup	Uniform(0.1, 0.2)	0.15 (0.10, 0.20)

Table P: Components of vector control costs

Item	Units (U)	Cost (C)
Entomological surveys, sensitization and management	Kilometers of river covered	Cost for entomological surveys, sensitisation and district management per kilometer × (1+PNLTHA markup)
Target deployment	Kilometers of river covered × Targets per kilometer × Number of deployments per year	Cost for target deployment per target × (1+PNLTHA markup)

Table Q: Vector control: cost function. Abbreviation: PNLTHA: Programme National de Lutte contre la Trypanosomiase Humaine, the national control program housed at the Ministry of Health.

Per year, the simulated costs according to the above formulation and parameters result in the following estimates:

Item	Units (U)	Cost (C)
**Targeted VC**		
Entomological surveys, sensitization and management	79 km	44,222 (19,548, 78,966)
Target deployment	4740 targets	23,046 (10,188, 40,814)
**Total**		**61,502 (35,541, 95,120)**
**Full VC**		
Entomological surveys, sensitization and management	601 km	336,422 (148,713, 600,741)
Target deployment	36060 targets	175,327 (77,508, 310,497)
**Total**		**467,883 (270,382, 723,635)**

Table R: Cost breakdown for vector control activities.

S1.10.4 Treatment

Traditionally, the stage of disease determined by microscopic examination of the cerebro-spinal fluid, which is extracted via lumbar puncture for cases that have been confirmed by visualisation of the trypanosome (e.g. cases in which trypanosomes are present in the blood). If trypanosomes are present in the cerebro-spinal fluid, the patient is considered to have stage 2 disease; if not, he or she is considered to have stage 1 disease. According to the stage of disease, patients are referred to the appropriate health centre or health district hospital for treatment.

In the context of fexinidazole treatment, which has been present in DRC since 2020, lumbar punctures will not be performed by the active screening team, but once patients are referred to a health centre, the health centre will determine eligibility for fexinidazole treatment.

We assumed the treatment algorithm based on the WHO interim recommendations of 2019 and the update from 2024 [39, 47].

- **Step 1, Group A. Patients without clinical symptoms of severe gHAT.** These patients would be eligible for fexinidazole treatment if their presentation fulfils the following criteria:
  - **Patient age** $<$ **6 years or weight** $<$ **20 kg.** These patients would be ineligible for fexinidazole treatment. For simplicity, we assumed that all patients over 6 years old were also over 20 kg due to scant data on patient characteristics. See Step 2, Group A.
  - **Patient age** $>$ **6 years old and weight** $>$ **20 kg.** These patients would be eligible for fexinidazole treatment. See Step 2, Group B. WHO recommendations stipulated that a doctor ought to be certain of the adherence on the part of the patient in order to prescribe fexinidazole on an outpatient basis. For simplicity, we have assumed that this is not an issue because it doesn't make a substantial difference in the total costs or effects of this particular analysis.
- **Step 1, Group B. Patients with clinical symptoms of severe HAT.** Patients whose clinical assessment would be consistent with severe gHAT (see page 7 WHO treatment guidelines [47]) would undergo a lumbar puncture to determine the concentration of white blood cells (WBCs) in the cerebrospinal fluid. For a concentration $<$ 100 WBC per microlitre (μL), the patient would be considered eligible for Fexinidazole treatment, depending on age and weight, as detailed in Step 1, Group A. In our model, we assumed that no stage 1 patient would show more than 100 WBC/μL of CSF as no trypanosomes should be present in the CSF. Moreover, we assumed that some proportion of stage 2 patients will be in late-stage disease (see Tables S-T).
- **Step 2, Group A. Patients ineligible for fexinidazole treatment.** Patients age $<$ 6 years old or weight $<$ 20 kg are assumed to submit to a lumbar puncture to determine disease stage with 100% adherence. In our treatment model, we consider the cost of a lumbar puncture in these patients (see Table-W) but we take the outcome of the lumbar puncture (stage 1 or stage 2) from the transmission model, the stage of disease is a critical output of the transmission model.
  - WHO recommendations stipulate the following criteria: if there is no trypanosoma in the CSF, then the patient undergoes pentamidine treatment on an inpatient basis. If there are more than 5 leukocytes (or WBC)/μL then the patient undergoes NECT treatment on an inpatient basis.
  - Pentamidine treatment would consist of intra-muscular infections for 7 days.
  - NECT (Nifurtimox-eflornithine combination therapy): nifurtimox is administered orally for 10 days, while eflornithine is administered intravenously for 7 days [47].
- **Step 2, Group B. Patients eligible for fexinidazole treatment.** We assumed that patients would be treated on an inpatient basis if age $>$ 6 years old and 20 kg $<$ weight $<$ 35 kg, otherwise, they would be treated on an outpatient basis as directly observed therapy.

Uncertainty Uncertainty in the treatment and cost parameters is parameterized according to the standard errors of estimates in the literature (see Supplementary Note 3: Parameter Glossary, Sections S4.6 and S4.9).

We show here the components of the costs per case treated, depending on the stage and the treatment. The parameters for the above table are available in Supplementary Note 3: Parameter Glossary, Section S4.9 and eligibility distributions are described in Table T.

Patient Characteristic	Parameterization	Summary
Under 6 years old	Beta(152.5, 2427.9)	0.06 (0.05, 0.07)
Under 35 kg of weight	Beta(8.3, 359.6)	0.02 (<0.01, 0.04)
Late stage-2 disease	Beta(76.9, 44.9)	0.63 (0.54, 0.72)

Table S: Parameters for treatment eligibility. Reproduced with permission from [6] under a CC-BY license.

Eligibility	Rationale	Summary
**Stage 1**		
Pentamidine	Under 6 years old (1)	0.06 (0.05, 0.07)
Fexinidazole-inpatient	Over 6 years old but under 35 kg of weight	0.02 (<0.01, 0.04)
Fexinidazole-outpatient	Over 6 years old and over 35 kg of weight	0.92 (0.90, 0.93)
**Stage 2**		
NECT	Under 6 years old or late-stage disease	0.65 (0.57, 0.73)
Fexinidazole-inpatient	Over 6 years old but under 35 kg of weight and early stage-2 disease	<0.01 (<0.01, 0.01)
Fexinidazole-outpatient	Over 6 years old, over 35 kg of weight, and early stage-2 disease	0.34 (0.26, 0.42)

[1] For simplicity, all patients over 6 years old were assumed to be over 20 kg in weight.

Table T: Eligibility for treatment

Treatment	Outcomes	Estimate
**Stage 1**		
Pentamidine	Cured	0.05 (0.05, 0.06)
	Cured with SAEs	<0.01 (<0.01, <0.01)
	Rescue treatment	<0.01 (<0.01, <0.01)
	Death	<0.01 (<0.01, <0.01)
Fexinidazole - inpatient	Cured	0.02 (<0.01, 0.04)
	Cured with SAEs	<0.01 (<0.01, <0.01)
	Rescue treatment	<0.01 (<0.01, <0.01)
	Death	<0.01 (<0.01, <0.01)
Fexinidazole - outpatient	Cured	0.89 (0.87, 0.91)
	Cured with SAEs	0.01 (<0.01, 0.02)
	Rescue treatment	0.02 (<0.01, 0.03)
	Death	<0.01 (<0.01, <0.01)
**All treatments**	**Cured**	**0.97 (0.95, 0.98)**
	**Cured with SAEs**	**0.01 (<0.01, 0.02)**
	**Rescue treatment**	**0.02 (0.01, 0.03)**
	**Death**	**<0.01 (<0.01, <0.01)**
**Stage 2**		
NECT	Cured	0.56 (0.49, 0.64)
	Cured with SAEs	0.06 (0.04, 0.08)
	Rescue treatment	0.03 (0.01, 0.04)
	Death	<0.01 (<0.01, <0.01)
Fexinidazole - inpatient	Cured	<0.01 (<0.01, 0.01)
	Cured with SAEs	<0.01 (<0.01, <0.01)
	Rescue treatment	<0.01 (<0.01, <0.01)
	Death	<0.01 (<0.01, <0.01)
Fexinidazole - outpatient	Cured	0.33 (0.25, 0.41)
	Cured with SAEs	<0.01 (<0.01, <0.01)
	Rescue treatment	<0.01 (<0.01, 0.01)
	Death	<0.01 (<0.01, <0.01)
**All treatments**	**Cured**	**0.90 (0.88, 0.92)**
	**Cured with SAEs**	**0.07 (0.05, 0.09)**
	**Rescue treatment**	**0.03 (0.02, 0.05)**
	**Death**	**<0.01 (<0.01, <0.01)**

Table U: Treatments and outcomes distributions for stage 1 and 2 patients, calculated according to the probability tree in F. SAE: severe adverse events.

Variable Name	Parameterization	Summary
Lumbar puncture and laboratory exam - cost	Gamma(2.42, 3.66)	13.72 (3.32, 31.10)
Duration of hospital stay for NECT treatment in days	Fixed	10
Duration of hospital stay fexinidazole for stage 1 or 2 disease in days	Fixed	10
Duration of severe adverse events in days	Gamma (1.219, 2.377)	2.91 (0.13, 9.84)
Probability of serious adverse events - pentamidine	Beta(1,499)	0.0026 (0.0002, 0.0083)
Probability of serious adverse events - NECT	Beta(11.6,226.4)	0.10 (0.07, 0.13)
Probability of serious adverse events - fexinidazole	Beta(3,261)	0.01 (<0.01, 0.03)
Outpatient consultation - cost	Gamma(24.2, 0.13)	3.14 (2.02, 4.48)
Hospital day - cost	Gamma(5.81, 0.59)	3.42 (1.24, 6.74)
Course of pentamidine - cost	Fixed	54
Course of NECT - cost	Fixed	360
Course of fexinidazole - cost	Fixed	50
Delivery mark-up	Beta(45,55)	0.45 (0.36, 0.55)
PNLTHA markup	Uniform(0.1, 0.2)	NA (NA, NA)

Table V: Parameters for treatment costs

	Pentamidine	NECT	Fexinidazole - inpatient	Fexinidazole - outpatient
Staging	15.09 (3.65, 34.21)	15.09 (3.65, 34.21)	0	0
Doctor's consult	34.57 (22.23, 49.24)	3.46 (2.22, 4.92)	3.46 (2.22, 4.92)	34.57 (22.23, 49.24)
Inpatient care	0	37.64 (13.63, 74.18)	37.64 (13.63, 74.18)	0
Medicine	86.16 (80.51, 91.96)	574.38 (536.75, 613.05)	79.78 (74.55, 85.15)	79.78 (74.55, 85.15)
Treatment for SAE	<0.01 (<0.01, 0.03)	1 (0, 5)	0 (0, 1)	0 (0, 1)
**Total**	**136 (117, 160)**	**632 (583, 688)**	**110 (87, 144)**	**114 (101, 130)**

Table W: Cost per person for different gHAT treatments. Because these are costs averaged over all patients and SAEs are rare, the average cost per patient for SAE is low.

S1.10.5 Treatment and deaths in the pre-2026 period

To calculate the DALYs and deaths from 2000-25, which are featured in GUI as well as in one selection of our supplemental results, we made certain assumptions about the ubiquity of certain treatments from during that period. The following is a description of the data and assumptions we took.

Untreated

- Before 2013, we have no records of how many people were untreated, so we assume 1% of people were untreated. Starting in 2013, we know from the fourth stakeholders' meeting report that 91%, 97%, 98%, 100%, 99% of all cases in the DRC were treated between 2013-17 [48], then from the program, we know that 100%, 92%, 96%, 85%, 94% from 2018-2022. We will assume that 2023-5 had the same proportion untreated as 2022. We suspect that there was not 100% treatment adherence in any year, so for those years that indicate 100% treatment, we assume that 1% of cases remain untreated.

Stage 1

- From 2000-2012, there was no development for stage 1 drugs, so we put in that 99% of all cases were treated by Pentamidine, the standard of care since the 1940's [21].
- In 2013, the fexinidazole trials started, so some proportion of S1 cases were then treated with Fexinidazole through trials [49]. Therefore, we assume 2% treatment with Fexinidazole in those trials in the period of 2013-16. From Nov 2016-Aug 2019, 7 clinics ran a prospective cohort with acoziborole treatment which was published along with data from 1 clinic in Guinea. We do not know how many of the 174 patients in that study were from DRC vs Guinea, but we know that fewer than 174 of the 4141 cases detected in DRC in those years cumulatively according to the HAT Atlas, which would equate to at most 4% of cases. If we only count the 1/6 of the cases in 2016, since the trial only ran for two months that year, and 8/12 cases in 2019, since the trial only ran for 8 months that year, we would consider a cumulative 2463 cases, so 174 cases would have been at most 7% of cases, which will be the proportion we will assume. Then we assume Fexinidazole again starting in 2020 when it was adopted as standard treatment. We will discuss below the process to parse the Fexinidazole treatments (which are unclassified between stage 1 and stage 2).
- See below for counts of S1 treated by Acoziborole during the trials.
- See below for post-2020 estimates.

Stage 2

- From 1948 until 2003, only melarsoprol was available for the treatment of stage 2. In the 1990's a short course was developed, so for 2000-02, 99% of cases were treated this way. Rescue treatment consisted of a long course of melarsoprol [21].
- In 2003-2006, eflornithine monotherapy was approved, but it was rarely used due to needs in training and complicated protocols. Therefore, by 2006, only 20% of cases (anywhere) were treated by eflornithine monotherapy, so I will assume that the prevalence was 5%, 10%, 15%, and 20% in 2003-06. The rest would be treated with melarsoprol short-course protocol [21].
- In 2006, the WHO prepared a kit to administer eflornithine monotherapy more easily, so its use increased to 64% by 2009 [21]. The rest would be assumed to be treated by melarsoprol. Therefore, from 2007-2009, we assumed that the percent of cases treated by eflornithine monotherapy would be 33%, 49%, 64% (essentially a linear interpolation from 20-64%). The rest would be treated by a short course of melarsoprol.
- From 2003-2006 there were about 240 patients enrolled in NECT trials in the DRC [50, 51]. We are going to ignore those proportions because they constitute less than half of a percent of all cases in that period.
- In 2009, nifurtimox-eflornithine combination therapy (NECT) was endorsed for use by the WHO, and they developed a kit for delivery. NECT then became the dominant treatment for stage 2 starting in 2010, such that 88% of cases in that year were treated by NECT and the remaining 12% by melarsoprol [21]. We will assume 88% of cases treated with NECT, 11% with melarsoprol, and 1% remained untreated.

- Fexinidazole trials did not begin until 2013, so from 2010-2012 we assumed the use of NECT increased even further, to 94% and then 99% in 2011 and 2012. The rest would be treated with melarsoprol.
- In 2013, fexinidazole trials began, so we apportion 2% of cases to be treated with that drug for 2013-16, and 7% for 2016-2019 (see stage 1 for reasoning) until fexinidazole is rolled out in 2020 [49, 52]. In the meantime, S2 cases are assumed to be treated with NECT.
- We will discuss below the process to parse the fexinidazole treatments (which are unclassified between stage 1 and stage 2).
- See below for counts of stage 1 patients treated by acoziborole during the trials.
- See below for post-2020 estimates.

Acoziborole trial in 2016-2019

- Between October 2016 and March 2019, there were 174 patients enrolled in DRC in the acoziborole trial [53]. That would be 10 trimesters. Therefore, we assumed that 1/10 were enrolled in 2016, 1/10 in 2019, and 4/10 in 2017 and 2018. So 17 cases were treated in 2016 with acoziborole, 70 cases in 2018, 70 cases in 2018, and 17 cases in 2019. Most of these were assumed to be in S2 of the disease, as only 20% of the cases enrolled were in “early or intermediate-stage of disease”, which was defined as an absence of trypanosome and white blood cells in the CSF; in other words, the definition of stage 1.
  - Therefore, we assumed that in 2016-2019, 3, 12, 12, and 3 S1 cases were treated with acoziborole, and 14, 58, 58, and 14 S2 cases were treated with acoziborole.
  - Therefore, we assume about 2% of all S2 cases in 2016 and 2019 and 8% of all S2 cases in 2017 and 2018 were treated by acoziborole, but we ignore the S1 cases treated by acoziborole because these are too few.

Calculating treatments as a proportion of S1 and S2 cases after 2020

- In 2020, fexinidazole was rolled out as a standard treatment. Because fexinidazole is prescribed for both stages, much of the data we now have about cases is not stratified by disease stage.
- We know there were 660, 613, 395, 424, and 516 cases in 2020-2022, and when one breaks those cases down by stages, we get:

Year	Cases, Stage UK	Cases, S1	Cases, S2	Total Cases
2018	21	305	334	660
2019	24	289	296	609
2020	65	127	203	395
2021	151	81	190	424
2022	287	75	154	516

Cases in DRC during the period of 2018-2022. Abbreviations: UK: unknown stage, S1: stage 1 disease, S2: stage 2 disease.

- For 2018 and 2019, the proportion of cases that are not staged is marginal, and about 46-47% of cases are in stage 1.
- We have data that 123, 84, and 34 cases from 2020-2022 were treated with pentamidine (PNLTHA program information). If we consider that 46% of all cases were in S1, then these pentamidine treatment account for 68%, 43% and 14% of all S1 cases in 2020-2022.
- We have data that 161, 142, and 132 cases from 2020-2022 were treated with NECT (PNLTHA program information). If we consider that 54% of all cases were in S2, then these NECT treatments account for 75%, 62% and 47% of all S2 cases in 2020-2022.

- The remaining cases were treated with fexinidazole, after accounting for the untreated. In S1, that would be 28%, 32%, and 80% for 2020-2022. In S2, that would be 21%, 23%, and 47% for 2020-2022. We assume a larger proportion of S1 cases are treated with fexinidazole than S2 cases because fexinidazole is contraindicated for late/severe disease.
- We assume that 2023-25 proportions are equal to 2022 proportions because we do not yet have any data on treatments in those years.

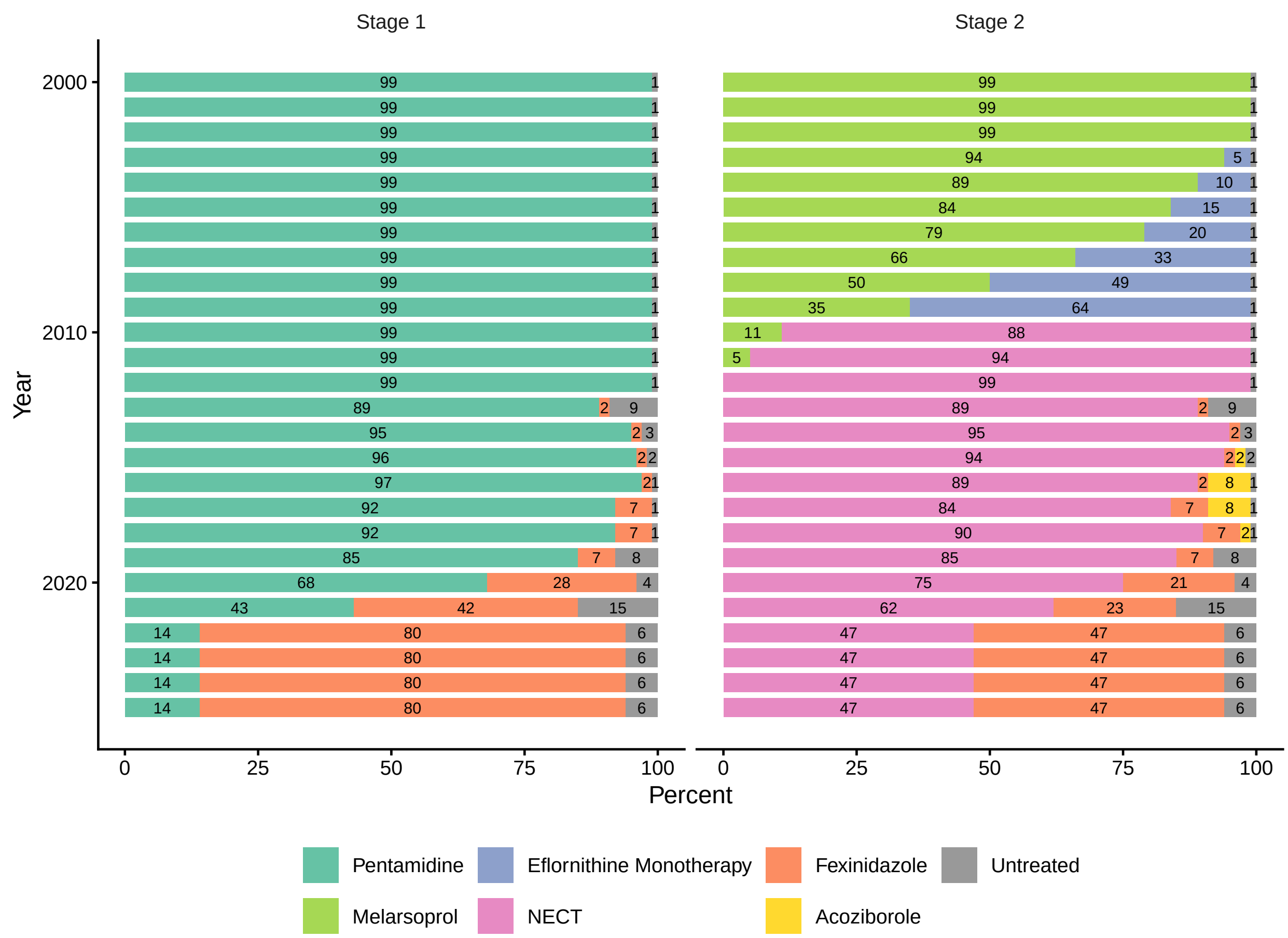

Treatments 2000-25. Since we have no data yet for what happened in 2023-25, we have assumed the same values as 2022. Abbreviations: NECT: nifurtimox-eflornithine combination therapy.

Parameter	Distribution	Summary	Sources	Supplementary Note 3 Section
Treatment failure for Pentamidine, S1	Beta(50.3, 665.48)	0.07 (0.05-0.09)	[54–59]	S4.6.7
SAEs for Pentamidine, S1	Beta(1.43, 551.42)	0.0026 (0.0002-0.0081)	[55, 58, 59]	S4.6.10
Treatment failure for Melarsoprol, S2	Beta(3, 247)	0.01 (0.003-0.03)	[60]	
SAEs for Melarsoprol, S2	Beta(47, 203)	0.19 (0.14-0.24)	[60]	
CFR for Melarsoprol	Beta(12, 408)	0.03 (0.02-0.05)	[60, 61]	
Treatment failure for Eflornithine, S2	Beta(14, 149)	0.09 (0.05-0.13)	[61]	
SAEs for Eflornithine, S2	Beta(41, 102)	0.29 (0.22-0.36)	[51]	
CFR for Eflornithine	Beta(9, 351)	0.02 (0.01-0.04)	[61]	
Treatment failure for NECT, S2	Beta(15.87, 378.55)	0.04 (0.02-0.06)	[49–51, 62–64]	S4.6.8
SAEs for NECT, S2	Beta(40.88, 367.8)	0.10 (0.07-0.13)	[49–51, 62–64]	S4.6.11
CFR for NECT	0	0	[49–51, 62–64]	
Treatment failure for Fexinidazole, S1 & S2-early	Beta(9.49, 497)	0.02 (<0.01–0.03)	[47, 49]	S4.6.9
SAEs for Fexinidazole, S1 & S2-early	Beta(3.00, 261)	0.01 (<0.01–0.03])	[47, 49]	S4.6.12
CFR for Fexinidazole	0	0	[47, 49]	
Treatment failure for Acoziborole, S1 & S2	Beta(8, 167)	0.05 (0.02-0.08)	[53]	
SAEs for Acoziborole, S1 & S2	Beta(1, 208)	0.005 (0.0001-0.02)	[53]	
CFR for Acoziborole, S1 & S2	0	0	[53]	
Sensitivity of RDT	Beta(230, 1)	1.00 (0.98-1.00)	[65]	S4.5.7
Age of death from infection (for years of life lost per fatal case)	Gamma(148, 0.18)	26.65 (22.56, 31.07)	[49–51, 54–59, 62–64, 66–69]	S4.6.15
Average years lost at age of death	Interpolation of life expectancy at ages 20–35	45.4 [41.4–49.9]	[70]	S4.6.16
DALY weight, S1	Beta(22.96, 147.21)	0.14 (0.09, 0.19)	[71]	S4.6.17
DALY weight, S2	Beta(18.37, 15.63)	0.54 (0.37, 0.70)	[71]	S4.6.18
DALY weight, SAE	Uniform(0.04, 0.11)	0.08 (0.04, 0.11)	[71]	S4.6.19
Duration S1 treatment	Fixed	7	[47]	S4.6.4
Duration S2 treatment	Fixed	10	[47]	S4.6.5 & S4.6.5
Duration SAE	Gamma(1.22, 2.38)	2.88 (0.14, 9.65)	[69]	S4.6.13

Parameters for calculation of treatments, DALYs, and deaths 2000-23. Parameters are discussed in more detail in Supplemental Note 3. Parameters that are not features in Supplemental Note 3 are not part of the main analysis (e.g. drugs that are no longer used in 2024 such as melarsoprol and eflornithine, or acoziborole which has only been used as part of a trial). Abbreviations: S1: stage 1 disease, S2: stage 2 disease, SAE: severe adverse events, CFR: case fatality rate, NECT: nifurtimox-eflornithine combination therapy, DALY: disability-adjusted life-year.

Summary of treatment outcomes for historical treatments. FP = treatments and outcomes given to false positives.

Drug	Outcome	Summary
Pentamidine	Convalescence & SAE	0.002 (<0.001, 0.008)
	Convalescence & no SAE	0.927 (0.907, 0.945)
	Detected relapse	0.070 (0.052, 0.090)
	Undetected relapse	<0.001 (<0.001, 0.001)
	Treatment-related death	0
Pentamidine, FP	Convalescence & SAE	0.003 (<0.001, 0.008)
	Convalescence & no SAE	0.997 (0.992, 1.000)
	Detected relapse	0
	Undetected relapse	0
	Treatment-related death	0
Melarsoprol	Convalescence & SAE	0.180 (0.136, 0.229)
	Convalescence & no SAE	0.779 (0.727, 0.826)
	Detected relapse	0.012 (0.002, 0.028)
	Undetected relapse	<0.001 (<0.001, <0.001)
	Treatment-related death	0.029 (0.015, 0.047)
Melarsoprol, FP	Convalescence & SAE	0.182 (0.138, 0.232)
	Convalescence & no SAE	0.789 (0.738, 0.836)
	Detected relapse	0
	Undetected relapse	0
	Treatment-related death	0.029 (0.015, 0.047)
Eflornithine	Convalescence & SAE	0.255 (0.191, 0.325)
	Convalescence & no SAE	0.634 (0.560, 0.706)
	Detected relapse	0.086 (0.047, 0.133)
	Undetected relapse	<0.001 (<0.001, 0.001)
	Treatment-related death	0.025 (0.011, 0.043)
Eflornithine, FP	Convalescence & SAE	0.280 (0.211, 0.354)
	Convalescence & no SAE	0.695 (0.620, 0.765)
	Detected relapse	0
	Undetected relapse	0
	Treatment-related death	0.025 (0.011, 0.043)
NECT	Convalescence & SAE	0.096 (0.070, 0.127)
	Convalescence & no SAE	0.864 (0.828, 0.895)
	Detected relapse	0.040 (0.023, 0.062)
	Undetected relapse	<0.001 (<0.001, <0.001)
	Treatment-related death	0
NECT, FP	Convalescence & SAE	0.100 (0.073, 0.132)
	Convalescence & no SAE	0.900 (0.868, 0.927)
	Detected relapse	0
	Undetected relapse	0
	Treatment-related death	0
Fexinidazole	Convalescence & SAE	0.011 (0.002, 0.027)
	Convalescence & no SAE	0.970 (0.950, 0.984)
	Detected relapse	0.019 (0.009, 0.032)
	Undetected relapse	<0.001 (<0.001, <0.001)
	Treatment-related death	0
Fexinidazole, FP	Convalescence & SAE	0.011 (0.002, 0.027)
	Convalescence & no SAE	0.989 (0.973, 0.998)
	Detected relapse	0
	Undetected relapse	0
	Treatment-related death	0
	Convalescence & SAE	0.005 (<0.001, 0.017)

Summary of treatment outcomes for historical treatments. FP = treatments and outcomes given to false positives. *(continued)*

Drug	Outcome	Summary
Acoziborole	Convalescence & no SAE	0.947 (0.913, 0.973)
	Detected relapse	0.048 (0.023, 0.081)
	Undetected relapse	<0.001 (<0.001, <0.001)
	Treatment-related death	0
Acoziborole, FP	Convalescence & SAE	0.005 (<0.001, 0.018)
	Convalescence & no SAE	0.995 (0.982, 1.000)
	Detected relapse	0
	Undetected relapse	0
	Treatment-related death	0
Untreated	Convalescence & SAE	0
	Convalescence & no SAE	0
	Detected relapse	0
	Undetected relapse	0
	Treatment-related death	1.000 (1.000, 1.000)
Melarsoprol, second-line	Convalescence & SAE	0.180 (0.136, 0.229)
	Convalescence & no SAE	0.779 (0.727, 0.826)
	Detected relapse	0
	Undetected relapse	0
	Treatment-related death	0.041 (0.022, 0.064)
NECT, second-line	Convalescence & SAE	0.096 (0.070, 0.127)
	Convalescence & no SAE	0.864 (0.828, 0.895)
	Detected relapse	0
	Undetected relapse	0
	Treatment-related death	0.040 (0.023, 0.062)

S1.11 Sensitivity analysis

↩ Return to the Supplement Table of Contents

Our GUI (https://hatmepp.warwick.ac.uk/DRCCEA/v8/) provides health impact, cost calculations, and cost-effectiveness results for alternative horizons of 2026–2030 and 2026–2050 with and without 3% discounting so the impact of these choices can be seen. By exploring the "Optimal Strategy" map it is noted that changing the time horizon or discounting only results in minor changes to a few health zones if the objective is elimination of transmission by 2030. Longer time horizons and lack of discounting do change recommended strategies to more intensive interventions in more health zones (such as adding vector control or increasing screening coverage) when we consider the minimum cost objective or objectives based on different WTP thresholds.

References for S1 Text

1. Crump RE, Huang CI, Knock ES, Spencer SEF, Brown PE, Mwamba Miaka E, Shampa C, Keeling MJ, and Rock KS. Quantifying epidemiological drivers of gambiense human African Trypanosomiasis across the Democratic Republic of Congo. PLOS Computational Biology 2021 Jan; 17. Ed. by Perkins A:e1008532. DOI: 10.1371/journal.pcbi.1008532. Available from: https://dx.plos.org/10.1371/journal.pcbi.1008532
2. Crump RE, Huang CI, Spencer SE, Brown PE, Shampa C, Miaka EM, and Rock KS. Modelling to infer the role of animals in gambiense human African trypanosomiasis transmission and elimination in the DRC. PLoS Neglected Tropical Diseases 2022; 16:1–23. DOI: 10.1371/JOURNAL.PNTD.0010599
3. Van Nieuwenhove S. Défis en matière de diagnostic de la Trypanosomiase Humaine Africaine: Evalution du projet de MSF OCG à Dingila, RDC. Tech. rep. Médecins sans Frontières, 2015
4. Rock KS, Torr SJ, Lumbala C, and Keeling MJ. Predicting the Impact of Intervention Strategies for Sleeping Sickness in Two High-Endemicity Health Zones of the Democratic Republic of Congo. PLOS Neglected Tropical Diseases 2017; 11:1–17. DOI: 10.1371/journal.pntd.0005162
5. Huang CI, Crump RE, Brown PE, Spencer SE, Miaka EM, Shampa C, Keeling MJ, and Rock KS. Identifying regions for enhanced control of gambiense sleeping sickness in the Democratic Republic of Congo. Nature Communications 2022; 13:1–11. DOI: 10.1038/s41467-022-29192-w
6. Antillon M, Huang CI, Crump RE, Brown PE, Snijders R, Miaka EM, Keeling MJ, Rock KS, and Tediosi F. Cost-effectiveness of sleeping sickness elimination campaigns in five settings of the Democratic Republic of Congo. Nature Communications 2022 Dec; 13:1051. DOI: 10.1038/s41467-022-28598-w. Available from: https://doi.org/10.1101/2020.08.25.20181982%20https://www.nature.com/articles/s41467-022-28598-w
7. Franco JR, Cecchi G, Paone M, Diarra A, Grout L, Ebeja AK, Simarro PP, Zhao W, and Argaw D. The elimination of human African trypanosomiasis: Achievements in relation to WHO road map targets for 2020. PLoS Neglected Tropical Diseases 2022; 16:1–19. DOI: 10.1371/JOURNAL.PNTD.0010047. Available from: http://dx.doi.org/10.1371/journal.pntd.0010047
8. American Red Cross. DRC Health Zone and Health Area boundaries. 2020 Feb. Available from: https://data.humdata.org/dataset/drc-health-data
9. Office for the Coordination of Humanitarian Affairs. Journees Nationales de Vaccination (JNV) activities de vaccination supplementaire, RDC. 2015. Available from: https://data.humdata.org/dataset/rdc-statistiques-des-populations
10. The World Factbook. Congo, Democratic Republic of the -. Available from: https://www.cia.gov/the-world-factbook/countries/congo-democratic-republic-of-the/#people-and-society
11. World Population Prospects 2022, Online Edition. Tech. rep. United Nations, Department of Economic and Social Affairs, Population Division, 2022
12. Davis CN, Crump RE, Sutherland SA, Spencer SEF, Corbella A, Chansy S, Lebuki J, Miaka EM, and Rock KS. Comparison of stochastic and deterministic models for gambiense sleeping sickness at different spatial scales: A health area analysis in the DRC. PLOS Computational Biology 2024 Apr; 20:1–25. DOI: 10.1371/journal.pcbi.1011993. Available from: https://doi.org/10.1371/journal.pcbi.1011993
13. The World Bank. Data:Democratic Republic of Congo. Accessed: 2015. 2015. Available from: https://data.worldbank.org/country/congo-dem-rep?view=chart
14. Rogers D. A general model for the African trypanosomiases. Parasitology 1988; 97:193–212
15. Checchi F, Filipe JAN, Barrett MP, and Chandramohan D. The natural progression of *Gambiense* sleeping sickness: What is the evidence? PLoS Neglected Tropical Diseases 2008 Dec; 2:e303
16. Checchi F, Funk S, Chandramohan D, Haydon DT, and Chappuis F. Updated estimate of the duration of the meningo-encephalitic stage in gambiense human African trypanosomiasis. BMC Research Notes 2015; 8:8–10. DOI: 10.1186/s13104-015-1244-3

17. Mpanya A, Hendrickx D, Vuna M, Kanyinda A, Lumbala C, Tshilombo V, Mitashi P, Luboya O, Kande V, Boelaert M, Lefèvre P, and Lutumba P. Should I get screened for sleeping sickness? A qualitative study in Kasai province, Democratic Republic of Congo. PLOS Neglected Tropical Diseases 2012 Jan; 6:e1467

18. Checchi F, Chappuis F, Karunakara U, Priotto G, and Chandramohan D. Accuracy of Five Algorithms to Diagnose *Gambiense* Human African Trypanosomiasis. PLoS Neglected Tropical Diseases 2011 Jul; 5:e1233–15

19. Davis S, Aksoy S, and Galvani A. A global sensitivity analysis for African sleeping sickness. Parasitology 2011; 138:516–26

20. Ravel S, Grébaut P, Cuisance D, and Cuny G. Monitoring the developmental status of *Trypanosoma brucei gambiense* in the tsetse fly by means of PCR analysis of anal and saliva drops. Acta Tropica 2003; 88:161–5

21. WHO Expert Committee on human African trypanosomiasis. Control and surveillance of human African trypanosomiasis: report of a WHO expert committee. Tech. rep. 2013 :237. Available from: https://www.who.int/trypanosomiasis_african/surveillance/en/

22. Rock KS, Torr SJ, Lumbala C, and Keeling MJ. Quantitative evaluation of the strategy to eliminate human African trypanosomiasis in the Democratic Republic of Congo. Parasites and Vectors 2015; 8:1–13. DOI: 10.1186/s13071-015-1131-8. Available from: http://dx.doi.org/10.1186/s13071-015-1131-8

23. Clausen PH, Adeyemi I, Bauer B, Breloeer M, Salchow F, and Staak C. Host preferences of tsetse (Diptera: Glossinidae) based on bloodmeal identifications. Medical and Veterinary Entomology 1998 Mar; 12:169–80

24. Mitashi P, Hasker E, Mbo F, Van Geertruyden JP, Kaswa M, Lumbala C, Boelaert M, and Lutumba P. Integration of diagnosis and treatment of sleeping sickness in primary healthcare facilities in the democratic republic of the congo. Tropical Medicine and International Health 2015; 20:98–105. DOI: 10.1111/tmi.12404

25. Mulenga P, Boelaert M, Lutumba P, Kelen CV, Coppieters Y, Chenge F, Lumbala C, Luboya O, and Mpanya A. Integration of Human African trypanosomiasis control activities into primary health services in the democratic republic of the Congo: A qualitative study of stakeholder perceptions. American Journal of Tropical Medicine and Hygiene 2019; 100:899–906. DOI: 10.4269/ajtmh.18-0382

26. Rock KS, Huang CI, Crump RE, Bessell PR, Brown PE, Tirados I, Solano P, Antillon M, Picado A, Mbainda S, Darnas J, Crowley EH, Torr SJ, and Peka M. Update of transmission modelling and projections of gambiense human African trypanosomiasis in the Mandoul focus, Chad. Infectious Diseases of Poverty 2022; 11:1–13. DOI: 10.1186/s40249-022-00934-8. Available from: https://doi.org/10.1186/s40249-022-00934-8

27. Antillon M, Huang CI, Sutherland SA, Crump RE, Bessell PR, Shaw APM, Tirados I, Picado A, Biéler S, Brown PE, Solano P, Mbainda S, Darnas J, Wang-Steverding X, Crowley EH, Peka M, Tediosi F, and Rock KS. Health economic evaluation of strategies to eliminate gambiense human African trypanosomiasis in the Mandoul disease focus of Chad. PLOS Neglected Tropical Diseases 2023 Jul; 17. Ed. by Mireji PO:e0011396. DOI: 10.1371/journal.pntd.0011396. Available from: https://dx.plos.org/10.1371/journal.pntd.0011396

28. Snijders R, Fukinsia A, Claeys Y, Hasker E, Mpanya A, Miaka E, Meheus F, and Boelaert M. Costs and outcomes of integrated human African trypanosomiasis surveillance system using rapid diagnostic tests, Democratic Republic of the Congo. Emerging Infectious Diseases 2021; 27:2144–53. DOI: 10.3201/eid2708.202399

29. Castaño MS, Ndeffo-Mbah ML, Rock KS, Palmer C, Knock E, Mwamba Miaka E, Ndung'u JM, Torr S, Verlé P, Spencer SEF, Galvani A, Bever C, Keeling MJ, and Chitnis N. Assessing the impact of aggregating disease stage data in model predictions of human African trypanosomiasis transmission and control activities in Bandundu province (DRC). PLOS Neglected Tropical Diseases 2020 Jan; 14:1–16. DOI: 10.1371/journal.pntd.0007976. Available from: https://doi.org/10.1371/journal.pntd.0007976

30. Tirados I, Hope A, Selby R, Mpembele F, Miaka EM, Boelaert M, Lehane MJ, Torr SJ, and Stanton MC. Impact of tiny targets on Glossina fuscipes quanzensis, the primary vector of human African trypanosomiasis in the Democratic Republic of the Congo. PLOS Neglected Tropical Diseases 2020 Oct; 14. Ed. by Matovu E:e0008270. DOI: 10.1371/journal.pntd.0008270. Available from: https://dx.plos.org/10.1371/journal.pntd.0008270

31. Aliee M, Rock KS, and Keeling MJ. Estimating the distribution of time to extinction of infectious diseases in mean-field approaches. Journal of the Royal Society Interface 2020; 17:20200540

32. Davis CN, Crump RE, Sutherland SA, Spencer SEF, Corbella A, Chansy S, Lebuki J, Miaka EM, and Rock KS. Comparison of stochastic and deterministic models for *gambiense* sleeping sickness at different spatial scales: A health area analysis in the DRC. MedRxiv 2023

33. Touloupou P, Alzahrani N, Neal P, Spencer SEF, and McKinley TJ. Efficient Model Comparison Techniques for Models Requiring Large Scale Data Augmentation. Bayesian Anal. 2018 Jun; 13:437–59. DOI: 10.1214/17-BA1057. Available from: https://doi.org/10.1214/17-BA1057

34. Hesterberg T. Weighted Average Importance Sampling and Defensive Mixture Distributions. Technometrics 1995; 37:185–94. Available from: https://www.jstor.org/stable/1269620

35. Rock KS, Ndeffo-Mbah ML, Castaño S, Palmer C, Pandey A, Atkins KE, Ndung'U JM, Hollingsworth TD, Galvani A, Bever C, Chitnis N, and Keeling MJ. Assessing strategies against gambiense sleeping sickness through mathematical modeling. Clinical Infectious Diseases 2018; 66:S286–S292. DOI: 10.1093/cid/ciy018

36. Simarro PP, Cecchi G, Franco JR, Paone M, Diarra A, Ruiz-Postigo JA, Mattioli RC, and Jannin JG. Mapping the capacities of fixed health facilities to cover people at risk of gambiense human African trypanosomiasis. International Journal of Health Geographics 2014; 13. DOI: 10.1186/1476-072X-13-4

37. Mahamat MH, Peka M, Rayaisse JB, Rock KS, Toko MA, Darnas J, Brahim GM, Alkatib AB, Yoni W, Tirados I, Courtin F, Brand SP, Nersy C, Alfaroukh IO, Torr SJ, Lehane MJ, and Solano P. Adding tsetse control to medical activities contributes to decreasing transmission of sleeping sickness in the Mandoul focus (Chad). PLoS Neglected Tropical Diseases 2017; 11:1–16. DOI: 10.1371/journal.pntd.0005792

38. Courtin F, Camara M, Rayaisse JB, Kagbadouno M, Dama E, Camara O, Traoré IS, Rouamba J, Peylhard M, Somda MB, Leno M, Lehane MJ, Torr SJ, Solano P, Jamonneau V, and Bucheton B. Reducing human-tsetse contact significantly enhances the efficacy of sleeping sickness active screening campaigns: A promising result in the context of elimination. PLOS Neglected Tropical Diseases 2015; 9:1–12. DOI: 10.1371/journal.pntd.0003727

39. WHO Department of Control of Neglected Tropical Diseases. WHO interim guidelines for the treatment of gambiense human African trypanosomiasis. Tech. rep. Geneva, Switzerland: World Health Organization, 2019. Available from: https://www.who.int/trypanosomiasis_african/resources/9789241550567/en/

40. Murray CJ. Quantifying the burden of disease: The technical basis for disability-adjusted life years. Bulletin of the World Health Organization 1994; 72:429–45. DOI: 10.1016/S0140-6736(96)07495-8

41. World Health Organization. Making Choices in Health: WHO guide to cost-effectiveness analysis. Tech. rep. Geneva, Switzerland, 2003 :318

42. Claxton KP, Revill P, Sculpher M, Wilkinson T, Cairns J, and Briggs A. The Gates Reference Case for Economic Evaluation. Tech. rep. April. The Bill and Melinda Gates Foundation, 2014 :1–68. Available from: https://pure.york.ac.uk/portal/en/publications/the-gates-reference-case-for-economic-evaluation(2663ebb2-e102-48c1-af2f-3a482a2eaae0)/export.html

43. Rushby JA and Hanson K. Calculating and presenting disability adjusted life years (DALYs) in cost-effectiveness analysis. Health Policy and Planning 2001 Sep; 16:326–31. DOI: 10.1093/heapol/16.3.326. Available from: https://academic.oup.com/heapol/article-lookup/doi/10.1093/heapol/16.3.326

44. Snijders R, Fukinsia A, Claeys Y, Mpanya A, Hasker E, Meheus F, Miaka E, and Boelaert M. Cost of a new method of active screening for human African trypanosomiasis in the Democratic Republic of the Congo. PLOS Neglected Tropical Diseases 2020 Dec; 14. Ed. by Ndung'u JM:e0008832. DOI: 10.1371/journal.pntd.0008832. Available from: https://dx.plos.org/10.1371/journal.pntd.0008832

45. Franco JR, Cecchi G, Priotto G, Paone M, Diarra A, Grout L, Simarro PP, Zhao W, and Argaw D. Monitoring the elimination of human African trypanosomiasis at continental and country level: Update to 2018. PLOS Neglected Tropical Diseases 2020 May; 14. Ed. by Matovu E:e0008261. DOI: 10.1371/journal.pntd.0008261. Available from: https://dx.plos.org/10.1371/journal.pntd.0008261

46. Snijders R, Shaw APM, Selby R, Tirados I, Bessell PR, Fukinsia A, Miaka E, Tediosi F, Hasker E, and Antillon M. The cost of sleeping sickness vector control in Yasa Bonga, a health district in the Democratic Republic of the Congo. PLOS Neglected Tropical Diseases 2024 Nov; 18. Ed. by Recuenco S:e0011959. DOI: 10.1371/journal.pntd.0011959. Available from: https://dx.plos.org/10.1371/journal.pntd.0011959
47. World Health Organization. Guidelines for the treatment of human African trypanosomiasis. Tech. rep. Geneva, 2024. Available from: https://iris.who.int/server/api/core/bitstreams/4da65589-ada0-40de-837c-1033af9733c4/content
48. World Health Organization. Report of the fourth WHO stakeholders meeting on gambiense and rhodesiense human African trypanosomiasis elimination. Tech. rep. Geneva: World Health Organization, 2022 Jun :1–80
49. Betu Kumesu VK, Kalonji WM, Bardonneau C, Mordt OV, Blesson S, Simon F, Delhomme S, Bernhard S, Kuziena W, Lubaki JPF, Vuvu SL, Ngima PN, Mbembo HM, Ilunga M, Bonama AK, Heradi JA, Solomo JLL, Mandula G, Badibabi LK, Dama FR, Lukula PK, Tete DN, Lumbala C, Scherrer B, Strub-Wourgaft N, and Tarral A. Oral fexinidazole for late-stage African Trypanosoma brucei gambiense trypanosomiasis: a pivotal multicentre, randomised, non-inferiority trial. The Lancet 2018; 391:144–54. DOI: 10.1016/S0140-6736(17)32758-7
50. Priotto G, Kasparian S, Ngouama D, Ghorashian S, Arnold U, Ghabri S, and Karunakara U. Nifurtimox-Eflornithine Combination Therapy for Second-Stage Trypanosoma brucei gambiense Sleeping Sickness: A Randomized Clinical Trial in Congo. Clinical Infectious Diseases 2007; 45:1435–42. DOI: 10.1086/522982
51. Priotto G, Kasparian S, Mutombo W, Ngouama D, Ghorashian S, Arnold U, Ghabri S, Baudin E, Buard V, Kazadi-Kyanza S, Ilunga M, Mutangala W, Pohlig G, Schmid C, Karunakara U, Torreele E, and Kande V. Nifurtimox-eflornithine combination therapy for second-stage African Trypanosoma brucei gambiense trypanosomiasis: a multicentre, randomised, phase III, non-inferiority trial. The Lancet 2009; 374:56–64. DOI: 10.1016/S0140-6736(09)61117-X. Available from: http://dx.doi.org/10.1016/S0140-6736(09)61117-X
52. Betu Kumeso VK, Perdrieu C, Menétrey C, Kyhi MIW, Tete DN, Camara M, Tampwo J, Kavunga P, Camara ML, Kourouma A, Mindele WK, Masa FA, Mahenzi H, Makaya J, Malu TM, Mandula G, Nzambi DMM, Ntoya SL, Reymondier A, Kalonji WM, Scherrer B, and Mordt OV. Effectiveness and safety of fexinidazole for gambiense human African trypanosomiasis and exploration of adherence in outpatients: a phase 3b, prospective, open-label, non-randomised, cohort study. The Lancet Global Health 2025 May; 13:e900–e909. DOI: 10.1016/S2214-109X(24)00526-6
53. Betu Kumeso VK, Kalonji WM, Rembry S, Valverde Mordt O, Ngolo Tete D, Prêtre A, Delhomme S, Ilunga Wa Kyhi M, Camara M, Catusse J, Schneitter S, Nusbaumer M, Mwamba Miaka E, Mahenzi Mbembo H, Makaya Mayawula J, Layba Camara M, Akwaso Massa F, Kaninda Badibabi L, Kasongo Bonama A, Kavunga Lukula P, Mutanda Kalonji S, Mariero Philemon P, Mokilifi Nganyonyi R, Embana Mankiara H, Asuka Akongo Nguba A, Kobo Muanza V, Mulenge Nasandhel E, Fifi Nzeza Bambuwu A, Scherrer B, Strub-Wourgaft N, and Tarral A. Efficacy and safety of acoziborole in patients with human African trypanosomiasis caused by Trypanosoma brucei gambiense: a multicentre, open-label, single-arm, phase 2/3 trial. The Lancet Infectious Diseases 2023 Apr; 23:463–70. DOI: 10.1016/S1473-3099(22)00660-0
54. Balasegaram M, Harris S, Checchi F, Hamel C, and Karunakara U. Treatment outcomes and risk factors for relapse in patients with early-stage human African trypanosomiasis (HAT) in the Republic of the Congo. Bulletin of the World Health Organization 2006; 84:777–82. DOI: 10.2471/BLT.05.028399
55. Eperon G, Schmid C, Loutan L, and Chappuis F. Clinical presentation and treatment outcome of sleeping sickness in Sudanese pre-school children. Acta Tropica 2006 Jan; 101:31–9. DOI: 10.1016/j.actatropica.2006.12.002. Available from: https://linkinghub.elsevier.com/retrieve/pii/S0001706X06002439
56. Ngoyi DM, Lejon V, Pyana P, Boelaert M, Ilunga M, Menten J, Mulunda JP, Van Nieuwenhove S, Muyembe Tamfum JJ, and Büscher P. How to Shorten Patient Follow-Up after Treatment for Trypanosoma brucei gambiense Sleeping Sickness. The Journal of Infectious Diseases 2010; 201:453–63. DOI: 10.1086/649917
57. Bastide S, Priotto G, Ecochard R, and Etard JF. Effectiveness of short vs. long treatment schedules with pentamidine in first-stage HAT: a large field cohort study. Tropical Medicine & International Health 2011; 16:68–9. Available from: https://onlinelibrary.wiley.com/toc/13653156/2011/16/s1

58. Burri C, Yeramian PD, Allen JL, Merolle A, Serge KK, Mpanya A, Lutumba P, Mesu VKBK, Bilenge CMM, Lubaki JPF, Mpoto AM, Thompson M, Munungu BF, Manuel F, Josenando T, Bernhard SC, Olson CA, Blum J, Tidwell RR, and Pohlig G. Efficacy, Safety, and Dose of Pafuramidine, a New Oral Drug for Treatment of First Stage Sleeping Sickness, in a Phase 2a Clinical Study and Phase 2b Randomized Clinical Studies. PLOS Neglected Tropical Diseases 2016; 10:1–18. DOI: 10.1371/journal.pntd.0004362

59. Pohlig G, Bernhard SC, Blum J, Burri C, Mpanya A, Lubaki JPF, Mpoto AM, Munungu BF, N'tombe PM, Deo GKM, Mutantu PN, Kuikumbi FM, Mintwo AF, Munungi AK, Dala A, Macharia S, Bilenge CMM, Mesu VKBK, Franco JR, Dituvanga ND, Tidwell RR, and Olson CA. Efficacy and Safety of Pafuramidine versus Pentamidine Maleate for Treatment of First Stage Sleeping Sickness in a Randomized, Comparator-Controlled, International Phase 3 Clinical Trial. PLOS Neglected Tropical Diseases 2016; 10:1–17. DOI: 10.1371/journal.pntd.0004363

60. Burri C, Nkunku S, Merolle A, Smith T, Blum J, and Brun R. Efficacy of new, concise schedule for melarsoprol in treatment of sleeping sickness caused by Trypanosoma brucei gambiense: a randomised trial. The Lancet 2000 Apr; 355:1419–25. DOI: 10.1016/S0140-6736(00)02141-3. Available from: https://linkinghub.elsevier.com/retrieve/pii/S0140673600021413

61. Lutje V, Seixas J, and Seixas A. Chemotherapy for second-stage Human African trypanosomiasis (Review). The Cochrane Collaboration 2009; 131:149–50. DOI: 10.1002/14651858.CD006201.pub3

62. Priotto G, Fogg C, Balasegaram M, Erphas O, Louga A, Checchi F, Ghabri S, and Piola P. Three Drug Combinations for Late-Stage Trypanosoma brucei gambiense Sleeping Sickness: A Randomized Clinical Trial in Uganda. PLOS Clinical Trials 2006; 1:e39. DOI: 10.1371/journal.pctr.0010039

63. Checchi F, Piola P, Ayikoru H, Thomas F, Legros D, and Priotto G. Nifurtimox plus eflornithine for late-stage sleeping sickness in Uganda: A case series. PLOS Neglected Tropical Diseases 2007; 1:1–6. DOI: 10.1371/journal.pntd.0000064

64. Kansiime F, Adibaku S, Wamboga C, Idi F, Kato CD, Yamuah L, Vaillant M, Kioy D, Olliaro P, and Matovu E. A multicentre, randomised, non-inferiority clinical trial comparing a nifurtimox-eflornithine combination to standard eflornithine monotherapy for late stage Trypanosoma brucei gambiense human African trypanosomiasis in Uganda. Parasites and Vectors 2018; 11:1–11. DOI: 10.1186/s13071-018-2634-x

65. Jamonneau V, Camara O, Ilboudo H, Peylhard M, Koffi M, Sakande H, N'Dri L, Sanou D, Dama E, Camara M, and Lejon V. Accuracy of Individual Rapid Tests for Serodiagnosis of Gambiense Sleeping Sickness in West Africa. PLOS Neglected Tropical Diseases 2015; 9:1–9. DOI: 10.1371/journal.pntd.0003480

66. Priotto G, Chappuis F, Bastard M, Flevaud L, and Etard JF. Early prediction of treatment efficacy in second-stage gambiense human african trypanosomiasis. PLOS Neglected Tropical Diseases 2012; 6. DOI: 10.1371/journal.pntd.0001662

67. Schmid C, Kuemmerle A, Blum J, Ghabri S, Kande V, Mutombo W, Ilunga M, Lumpungu I, Mutanda S, Nganzobo P, Tete D, Mubwa N, Kisala M, Blesson S, and Mordt OV. In-Hospital Safety in Field Conditions of Nifurtimox Eflornithine Combination Therapy (NECT) for T. b. gambiense Sleeping Sickness. PLOS Neglected Tropical Diseases 2012; 6. DOI: 10.1371/journal.pntd.0001920

68. Hasker E, Lutumba P, Chappuis F, Kande V, Potet J, De Weggheleire A, Kambo C, Depoortere E, Pécoul B, and Boelaert M. Human African Trypanosomiasis in the Democratic Republic of the Congo: A Looming Emergency? PLOS Neglected Tropical Diseases 2012; 6:e1950. DOI: 10.1371/journal.pntd.0001950

69. Alirol E, Schrumpf D, Amici Heradi J, Riedel A, De Patoul C, Quere M, and Chappuis F. Nifurtimox-eflornithine combination therapy for second-stage gambiense human African trypanosomiasis: Médecins Sans Frontières experience in the Democratic Republic of the Congo. Clinical Infectious Diseases 2013; 56:195–203. DOI: 10.1093/cid/cis886

70. United Nations Population Division. World Population Prospects 2019. 2019. Available from: https://population.un.org/wpp/

71. GBD 2017 DALYs and HALE Collaborators. Global, regional, and national disability-adjusted life-years (DALYs) for 359 diseases and injuries and healthy life expectancy (HALE) for 195 countries and territories, 1990-2017: A systematic analysis for the Global Burden of Disease Study 2017. The Lancet 2018; 392:1859–922. DOI: 10.1016/S0140-6736(18)32335-3
