# Supplementary material for: Cost-effectiveness of end-game strategies against sleeping sickness across the Democratic Republic of Congo
Source: PLoS Negl Trop Dis. 2026 Oct 22;20(10):e0014777. doi: 10.1371/journal.pntd.0014777 (PMC13719019; doi:10.1371/journal.pntd.0014777)
Supplement: Supplementary file 2 [file pntd.0014777.s002.pdf]

# Supplementary Note 1: Glossary of Technical Terms

Box 1: Glossary (adapted from Antillon et. al 2022 [1] under a CC-BY 4.0 licence.)

### Epidemiology Terms

**Intervention** Interventions are separate activities to address a health need (e.g. active screening (AS) or vector control (VC)).

**Strategy** A strategy combines interventions with specific coverage. In this paper, we simulate strategies with and without an improvement in AS and with and without VC (e.g. Strategy 1 is passive screening (PS) and mean AS, and Strategy 6 is PS, intensified AS and full VC).

**Elimination of transmission (EoT)** Globally this is the 2030 goal for gHAT; here we also consider local EOT for health zones. The feasibility of EoT is expressed as a probability equal to the proportion of our simulations in which new infections are zero before a given year (usually 2030).

**Objective** The objective is the overarching goal of the decision maker; this could be EoT by 2030, minimising costs, or delivering a cost-effective programme compared to other diseases. We assume the objective is country-wide and different regions may need different strategies to meet the objective.

**Disability-adjusted life-year (DALY)** In order to present the burden of disease as one common metric across diseases, DALYs are calculated in cost-effectiveness analyses. This is the sum of the years lived with disability due to the disease and the years of life lost by fatal cases.

### Health Economics Terms

**Parameter uncertainty** Uncertainty in the level of transmission or the costs of interventions due to unknown underlying parameters. See Text S4 for the explanation of our parameterisation of the health outcomes and cost model.

**Willingness-to-pay (WTP) or cost-effectiveness threshold** The amount of money that payers would pay to avert one DALY arising from the disease in the analysis (gHAT). No specific threshold is recommended, but a recent analysis shows that the WTP in DRC is between 5–230 USD per DALY averted [2–4].

**Incremental cost-effectiveness ratio** A ratio of marginal cost for a marginal benefit, calculated as follows:

$$\text{ICER} = \frac{\Delta\text{Costs}}{\Delta\text{DALYs}} = \frac{\text{Costs}_{\text{strategy}} - \text{Costs}_{\text{next best}}}{\text{Effects}_{\text{strategy}} - \text{Effects}_{\text{next best}}}$$

**Cost-effective strategy** The strategy where the ICER is less than the WTP (or cost-effectiveness threshold). We say that the cost-effective strategy is “conditional" on the WTP.

**Dominated strategy** A strategy that costs more than the minimum cost intervention while reducing the burden by a smaller degree. This strategy ought not to be implemented.

**Weakly dominated strategy (or strategies under extended dominance)** A strategy in which the ICER is higher than the next more expensive strategy. This strategy is less efficient than the next more expensive one and should not be implemented. For further illustration of weak dominance, see Supplementary Section, page 46 of Antillon et. al 2022 [1].

**Net monetary benefit** The net benefits (NMB) framework is derived from ICERs but also takes uncertainty into account.

$$\text{NMB}|\text{WTP} = \text{WTP} \times \Delta\text{DALYs} - \Delta\text{Costs}$$

The optimal strategy at a given WTP is the strategy with the highest mean NMB at that value of WTP.

**Optimal strategy** Analogous to the cost-effective strategy when no uncertainty is assumed, this is the strategy that is recommended by the NMB framework.

## References for S2 Text

1. Antillon M, Huang CI, Crump RE, Brown PE, Snijders R, Miaka EM, Keeling MJ, Rock KS, and Tediosi F. Cost-effectiveness of sleeping sickness elimination campaigns in five settings of the Democratic Republic of Congo. Nature Communications 2022 Dec; 13:1051. DOI: 10.1038/s41467-022-28598-w. Available from: https://doi.org/10.1101/2020.08.25.20181982%20https://www.nature.com/articles/s41467-022-28598-w
2. Bertram MY, Lauer JA, Joncheere KD, Edejer T, Hutubessy R, Kieny P, Hill SR, and Bertram MY. Cost – effectiveness thresholds: pros and cons. Bull World Health Organization 2016; 94:925–30. DOI: 10.2471/BLT.15.164418
3. Marseille E, Larson B, Kazi DS, Kahn JG, and Rosen S. Thresholds for the cost–effectiveness of interventions: Alternative approaches. Bulletin of the World Health Organization 2015; 93:118–24. DOI: 10.2471/BLT.14.138206
4. Woods B, Revill P, Sculpher M, and Claxton K. Country-Level Cost-Effectiveness Thresholds: Initial Estimates and the Need for Further Research. Value in Health 2016; 19:929–35. DOI: 10.1016/j.jval.2016.02.017. Available from: http://dx.doi.org/10.1016/j.jval.2016.02.017
