# Supplementary material for: Cost-effectiveness of end-game strategies against sleeping sickness across the Democratic Republic of Congo
Source: PLoS Negl Trop Dis. 2026 Oct 22;20(10):e0014777. doi: 10.1371/journal.pntd.0014777 (PMC13719019; doi:10.1371/journal.pntd.0014777)
Supplement: Supplementary file 3 [file pntd.0014777.s003.pdf]

Supplementary Note 2: Health Zone-Specific Parameters

Health zone-specific parameters, grouped by coordination. For a map of the coordinations, see Fig A in S1 Text.

Health Zone	Province	Population (2023)	AS mean	AS int.	PS clinics	VC targeted (km)	VC full (km)
**Bandundu Nord**							
Bagata	Kwilu	210,271	51,314	73,911	3	395.18	458.60
Bandjau	Mai-Ndombe	147,021	28,749	54,485	1	0.00	49.14
Bandundu	Kwilu	242,325	51,895	103,021	8	133.47	178.93
Bokoro	Mai-Ndombe	276,470	69,388	97,879	3	224.85	444.97
Bolobo	Mai-Ndombe	173,678	77,928	111,073	6	33.33	211.22
Bosobe	Mai-Ndombe	160,728	27,352	52,073	1	0.00	178.06
Djuma	Kwilu	269,689	35,783	69,791	6	88.09	105.62
Inongo	Mai-Ndombe	225,939	1,352	58,469	1	0.00	742.18
Kikongo	Kwilu	239,132	43,035	71,489	3	79.38	600.64
Kiri	Mai-Ndombe	140,587	0	36,381	1	0.00	633.15
Kwamouth	Mai-Ndombe	161,140	62,336	89,363	5	409.29	813.11
Mushie	Mai-Ndombe	159,222	29,415	43,774	5	86.61	345.87
Nioki	Mai-Ndombe	202,109	20,837	60,441	3	355.97	392.12
Ntand Embelo	Mai-Ndombe	109,868	28,561	58,098	2	0.00	0.00
Oshwe	Mai-Ndombe	185,952	4,216	48,121	1	0.00	916.39
Sia	Kwilu	140,256	21,403	36,296	1	139.69	139.69
Vanga	Kwilu	366,524	38,636	94,850	2	93.42	93.42
Yumbi	Mai-Ndombe	149,793	29,297	53,380	2	44.51	76.02
**Bandundu Sud**							
Boko	Kwango	264,635	7,727	68,484	2	10.95	482.91
Bulungu	Kwilu	347,987	60,251	90,053	3	56.09	137.35
Idiofa	Kwilu	358,183	29,260	92,692	1	107.04	152.77
Ipamu	Kwilu	250,413	35,472	64,803	4	232.63	400.63
Kasongo Lunda	Kwango	214,302	17,931	55,458	1	0.00	197.65
Kenge	Kwango	358,461	48,759	92,764	2	130.44	400.96
Kikwit Nord	Kwilu	273,345	51	70,737	1	25.83	25.83
Kimbau	Kwango	227,664	1,511	58,914	2	0.00	429.13
Kimputu	Kwilu	248,679	45,957	73,093	3	102.59	174.77
Koshibanda	Kwilu	233,393	18,874	60,398	1	0.00	384.72
Lusanga	Kwilu	298,567	26,179	77,263	1	0.00	248.57
Masi Manimba	Kwilu	245,840	91,636	107,678	3	161.82	293.46
Moanza	Kwilu	212,771	9,863	55,061	2	0.00	271.92
Mokala	Kwilu	273,648	53,173	70,815	5	54.93	253.37
Mosango	Kwilu	153,827	56,588	76,654	1	5.50	54.89
Mungindu	Kwilu	155,591	1,624	40,264	1	0.00	313.07
Pay Kongila	Kwilu	196,891	1,182	50,951	1	0.00	204.74
Popokabaka	Kwango	229,297	25,945	72,688	1	0.00	354.76
Yasa Bonga	Kwilu	272,929	166,959	201,251	4	417.00	417.00
**Équateur Nord**							
Bangabola	Sud-Ubangi	173,405	282	47,084	1	0.00	106.05
Bogose Nubea	Sud-Ubangi	222,573	23,957	57,598	1	0.00	356.39
Bokonzi	Sud-Ubangi	170,691	300	44,172	2	0.00	266.14
Bominenge	Sud-Ubangi	198,664	10,807	51,411	2	0.00	25.49

Boso Manzi	Mongala	159,168	788	41,190	1	0.00	293.21
Bosobolo	Nord-Ubangi	204,421	1,570	52,901	1	0.00	220.61
Boto	Sud-Ubangi	266,045	14,840	68,848	1	0.00	119.72
Budjala	Sud-Ubangi	164,096	367	44,632	2	0.00	142.19
Bulu	Sud-Ubangi	170,517	814	44,127	1	0.00	87.68
Businga	Nord-Ubangi	158,634	3,685	41,052	2	0.00	393.59
Bwamanda	Sud-Ubangi	289,733	16,481	79,273	3	0.00	232.05
Gbadolite	Nord-Ubangi	179,656	0	46,492	1	0.00	68.26
Gemena	Sud-Ubangi	426,803	23,391	110,449	1	0.00	0.00
Karawa	Nord-Ubangi	310,957	33,788	110,145	4	0.00	78.58
Kungu	Sud-Ubangi	274,325	9,724	83,355	3	0.00	87.13
Libenge	Sud-Ubangi	285,692	0	73,932	1	0.00	273.28
Loko	Nord-Ubangi	156,329	18,065	63,411	1	0.00	168.60
Mawuya	Sud-Ubangi	196,193	0	50,771	1	0.00	301.66
Mbaya	Sud-Ubangi	84,186	0	21,786	1	0.00	155.51
Mobayi	Nord-Ubangi	139,236	0	36,033	1	0.00	256.20
Ndage	Sud-Ubangi	160,806	3,644	41,614	1	0.00	167.03
Tandala	Sud-Ubangi	348,292	23,421	93,818	2	0.00	0.00
**Équateur Sud**							
Befale	Tshuapa	212,378	1,384	54,960	1	0.00	891.60
Bikoro	Équateur	192,101	4,212	49,712	1	0.00	180.53
Bomongo	Équateur	137,605	485	35,610	1	0.00	547.31
Iboko	Équateur	126,985	673	32,862	1	0.00	61.42
Irebu	Équateur	44,625	1,320	11,549	1	0.00	157.94
Lukolela	Équateur	178,878	10,574	46,291	4	15.19	214.43
Mompono	Tshuapa	170,697	1,606	44,173	1	0.00	430.76
Ntondo	Équateur	82,085	2,213	21,242	2	0.00	61.75
Wangata	Équateur	186,862	0	48,358	1	6.16	24.78
**Isangi-Tschopo**							
Isangi	Tshopo	198,596	22,633	51,392	4	107.63	268.80
Yabaondo	Tshopo	208,445	21,831	53,942	11	141.60	204.47
Yahisuli	Tshopo	106,345	1,190	27,520	1	0.00	300.01
Yakusu	Tshopo	198,205	15,378	51,292	1	108.32	196.93
**Kasaï Occidental**							
Bena Leka	Kasaï-Central	357,282	834	92,458	1	0.00	259.52
Bulape	Kasaï	217,982	6,172	56,411	2	0.00	182.50
Demba	Kasaï-Central	420,288	157	108,763	1	0.00	187.33
Dibaya	Kasaï-Central	273,154	0	70,687	1	0.00	22.81
Kakenge	Kasaï	208,410	18,675	53,933	2	0.00	166.24
Katende	Kasaï-Central	117,038	14,606	30,286	1	0.00	0.00
Luambo	Kasaï-Central	349,796	3,422	90,521	1	0.00	286.83
Lubunga 2	Kasaï-Central	128,006	21,383	33,126	1	112.54	133.41
Luebo	Kasaï	311,576	0	80,630	1	0.00	321.49
Luiza	Kasaï-Central	220,576	5,604	57,081	1	0.00	257.85

Masuika	Kasaï-Central	267,402	30,125	69,199	1	109.88	234.58
Mikope	Kasaï	239,645	114	62,016	1	0.00	475.07
Mushenge	Kasaï	209,231	13,078	54,145	2	0.00	27.56
Mweka	Kasaï	259,573	10,770	67,174	1	0.00	124.38
Tshibala	Kasaï-Central	312,867	2,332	80,965	1	0.00	88.52
Tshikula	Kasaï-Central	171,435	12,555	44,364	3	0.00	32.23
Yangala	Kasaï-Central	196,524	3,073	50,857	1	0.00	221.68
Kapanga	Lualaba	188,362	382	48,745	1	0.00	639.29
**Kasaï Oriental**							
Bibanga	Kasaï-Oriental	169,065	22,423	43,750	8	74.10	152.90
Bonzola	Kasaï-Oriental	259,302	3	67,103	2	20.03	20.03
Cilindu	Kasaï-Oriental	234,503	7,303	60,685	1	49.70	98.67
Kabeya Kamuanga	Kasaï-Oriental	210,427	9,046	54,455	2	0.00	132.99
Kabinda	Lomami	353,298	3,763	91,427	1	0.00	0.00
Kalambayi Kabanga	Lomami	201,056	19,411	52,030	2	70.92	280.32
Kalenda	Lomami	255,034	3,165	65,998	4	0.00	241.28
Kanda Kanda	Lomami	299,395	3,544	77,477	2	0.00	172.26
Kasansa	Kasaï-Oriental	265,689	20,311	68,756	3	0.00	138.25
Lubao	Lomami	265,120	1,437	68,608	1	0.00	258.07
Luputa	Lomami	372,027	2,389	96,274	1	0.00	251.76
Miabi	Kasaï-Oriental	188,071	11,256	48,669	2	0.00	40.48
Mpokolo	Kasaï-Oriental	393,264	3,612	101,770	3	0.00	0.00
Mukumbi	Kasaï-Oriental	152,578	12,057	39,484	3	0.00	84.37
Mulumba	Lomami	368,899	10,006	95,465	4	0.00	118.39
Mwene Ditu	Lomami	542,962	7,746	140,508	1	0.00	46.05
Ngandajika	Lomami	399,796	7,133	103,459	7	34.33	78.68
Nzaba	Kasaï-Oriental	377,559	2,033	97,707	1	0.00	0.00
Tshilenge	Kasaï-Oriental	382,087	21,963	98,878	4	83.99	83.99
Tshitenge	Kasaï-Oriental	297,337	7,089	76,946	3	88.72	88.72
Tshitshimbi	Kasaï-Oriental	246,246	6,119	63,724	1	0.00	46.32
Tshofa	Lomami	172,721	372	44,698	1	0.00	329.18
**Kinshasa**							
Kimbanseke	Kinshasa	385,527	144	99,768	3	19.16	19.16
Kingabwa	Kinshasa	287,364	0	74,366	1	0.00	22.75
Kisenso	Kinshasa	593,525	0	153,594	2	0.00	15.67

Maluku 1	Kinshasa	246,514	9,808	63,794	2	69.42	345.91
Maluku 2	Kinshasa	90,243	5,871	23,353	6	0.00	542.18
Masa	Kongo-Central	129,020	693	33,388	1	0.00	111.81
Masina 1	Kinshasa	457,864	0	118,486	1	13.80	13.80
Masina 2	Kinshasa	378,110	0	97,848	1	10.97	10.97
Matete	Kinshasa	395,222	0	102,277	1	0.00	12.02
Mont Ngafula 1	Kinshasa	338,221	6,791	87,525	3	21.15	21.15
Mont Ngafula 2	Kinshasa	198,140	3,479	51,275	3	23.15	23.15
Ndjili	Kinshasa	441,354	0	114,215	1	0.00	14.54
Nsele	Kinshasa	241,160	9,807	62,409	3	50.40	116.90
**Kongo Central**							
Boma	Kongo-Central	251,100	0	64,980	3	24.71	24.71
Boma Bungu	Kongo-Central	105,720	5,158	27,358	4	0.00	91.14
Gombe Matadi	Kongo-Central	132,897	735	34,391	4	0.00	185.77
Inga	Kongo-Central	105,758	12,190	28,177	9	0.00	95.61
Kangu	Kongo-Central	131,965	1,200	34,150	2	0.00	72.35
Kibunzi	Kongo-Central	83,098	3,369	21,504	5	0.00	119.40
Kimpese	Kongo-Central	234,061	10,811	60,571	11	0.00	240.08
Kinkonzi	Kongo-Central	91,565	0	23,695	1	0.00	18.55
Kwilu Ngongo	Kongo-Central	189,452	4,507	49,027	6	0.00	78.91
Lukula	Kongo-Central	240,077	7,480	62,128	9	0.00	56.53
Luozi	Kongo-Central	117,418	831	30,386	6	0.00	93.29
Mangembo	Kongo-Central	93,713	397	24,251	2	0.00	30.45
Matadi	Kongo-Central	242,113	146	62,655	1	0.00	43.33
Muanda	Kongo-Central	176,470	625	45,667	8	0.00	0.00
Nsona Pangu	Kongo-Central	142,257	4,672	36,814	6	0.00	378.61
Seke Banza	Kongo-Central	186,948	675	48,378	4	0.00	0.00
Tshela	Kongo-Central	118,731	0	30,725	1	0.00	51.71
**Maniema Katanga**							
Kabalo	Tanganika	313,960	0	81,247	1	0.00	337.91
Kabambare	Maniema	147,506	0	38,172	1	0.00	407.46
Kalemie	Tanganika	416,591	0	107,807	1	0.00	141.02
Kasongo	Maniema	282,945	20,606	73,221	1	0.00	181.92
Kibombo	Maniema	132,833	7,493	34,376	1	0.00	628.31

Kongolo	Tanganika	416,590	20,434	107,806	1	143.89	184.61
Kunda	Maniema	337,462	8,144	87,328	1	88.78	223.89
Lusangi	Maniema	215,408	1,797	55,744	1	0.00	234.60
Mbulula	Tanganika	236,974	19,509	61,326	1	0.00	0.00
Nyunzu	Tanganika	322,497	7,633	83,457	1	0.00	318.34
Salamabila	Maniema	152,657	5,218	39,505	1	0.00	127.77
Samba	Maniema	183,967	2,760	47,609	2	0.00	342.70
Tunda	Maniema	107,209	197	27,743	1	0.00	359.77
**Sankuru**							
Dikungu	Sankuru	183,360	19,792	47,449	3	0.00	94.21
Katako Kombe	Sankuru	174,648	0	45,196	1	0.00	564.42
Lodja	Sankuru	239,464	0	61,969	1	0.00	236.39
Lusambo	Sankuru	117,517	1	30,410	1	0.00	380.86
Minga	Sankuru	207,498	11,473	53,697	3	0.00	113.85
Pania Mutombo	Sankuru	95,225	611	24,642	1	0.00	233.41
Tshumbe	Sankuru	126,130	6,473	32,640	1	189.89	312.89
Wembo Nyama	Sankuru	109,592	25,408	29,871	1	72.90	279.63
**Bas Uélé**							
Ango	Bas Uélé	132,690	0	10,286	1	753.41	1,460.35
Doruma	Haut Uélé	87,509	0	6,273	1	0.00	319.71
