# Supplementary material for: Cost-effectiveness of end-game strategies against sleeping sickness across the Democratic Republic of Congo
Source: PLoS Negl Trop Dis. 2026 Oct 22;20(10):e0014777. doi: 10.1371/journal.pntd.0014777 (PMC13719019; doi:10.1371/journal.pntd.0014777)
Supplement: Supplementary file 4 [file pntd.0014777.s004.pdf]

Supplementary Materials
Cost-effectiveness of end-game strategies against sleeping sickness across the Democratic Republic of Congo

Supplementary Note 3: Parameter Glossary

This glossary is adapted from the parameter glossaries in previous work [1, 2].

Contents

S4.1 Principles for parameterization . . . 3
S4.2 Organization of parameters . . . 3
S4.3 Summary of health outcome parameters. . . . 4
S4.4 Summary of cost parameters . . . 5
S4.5 Screening parameters . . . 6
S4.5.1 Population . . . 6
S4.5.2 PS: coverage of the population per facility . . . 6
S4.5.3 PS: number of facilities . . . 8
S4.5.4 AS: coverage . . . 8
S4.5.5 AS: capacity per team per year . . . 8
S4.5.6 CATT 1:8 algorithm: diagnostic specificity . . . 8
S4.5.7 RDT algorithm: diagnostic sensitivity . . . 9
S4.5.8 RDT algorithm: diagnostic specificity . . . 9
S4.5.9 CATT algorithm: wastage during AS . . . 9
S4.5.10 RDT algorithm: wastage during PS . . . 10
S4.6 Treatment parameters . . . 10
S4.6.1 Proportion of cases age<6 . . . 10
S4.6.2 Proportion of cases weight<35 kg among age>6 . . . 11
S4.6.3 Proportion of S2 cases that are severe . . . 12
S4.6.4 Length of hospital stay: Pentamidine treatment . . . 13
S4.6.5 Length of hospital stay: NECT treatment . . . 13
S4.6.6 Length of hospital stay: fexinidazole treatment . . . 14
S4.6.7 Pr. of relapse: pentamidine . . . 14
S4.6.8 Pr. of relapse (treatment failure): NECT . . . 15
S4.6.9 Pr. of relapse: fexinidazole . . . 15
S4.6.10 SAE: pentamidine treatment . . . 16
S4.6.11 SAE: NECT treatment . . . 16
S4.6.12 SAE: fexinidazole treatment. . . . 17
S4.6.13 Days lost to disability: due to SAE . . . 18
S4.6.14 Life-years lost (DALY) parameters . . . 18
S4.6.15 Age of death from infection . . . 18
S4.6.16 Life expectancy . . . 18
S4.6.17 Disability weights: S1 disease . . . 20
S4.6.18 Disability weights: S2 disease . . . 20
S4.6.19 Disability weights: SAE . . . 20

S4.7 Vector control parameters 21
S4.7.1 Linear km of targets 21
S4.7.2 Target per km 21
S4.7.3 Replacement rate of targets per year 22
S4.8 Screening cost parameters 22
S4.8.1 AS: capital costs of a team 22
S4.8.2 AS: recurrent & management costs of a team 22
S4.8.3 CATT algorithm: cost per test used 23
S4.8.4 Staging: lumbar puncture & lab exam 23
S4.8.5 Confirmation: microscopy 24
S4.8.6 RDT: costs per test used 24
S4.8.7 Indirect management costs (PNLTHA mark-up) 25
S4.8.8 PS: capital costs of a facility 25
S4.8.9 PS: management costs 25
S4.9 Treatment cost parameters 26
S4.9.1 Hospital stay: cost per day 26
S4.9.2 Outpatient consultation: cost 26
S4.9.3 Course of pentamidine: cost 27
S4.9.4 Course of NECT: cost 27
S4.9.5 Course of fexinidazole: cost 27
S4.9.6 Drug delivery mark-up 27
S4.10 Vector control cost parameters 28
S4.10.1 Operational cost per kilometer of riverbank covered 28
S4.10.2 Deployment cost per target 28

S4.1 Principles for parameterization

(Please note that this Supplemental Note 3 contains recycled text from the authors' previous publications [1, 2]. Parameter values and citations have been updated where appropriate.)

Below are the guidelines our team follows to parameterize the parameters in the treatment and intervention model. The rationale behind the choices in the tables that describe parameters is easier with these guidelines in mind.

- **Transferability of costs across time** Costs from the literature are updated to 2024 USD values by converting to local currency units in the year of the study in the literature, inflated to 2024 values using the consumer price index (CPI) of the country, and then converted to USD using the exchange rate in 2024. It should be noted that the 2003 WHO Guide to Cost-effectiveness recommends that the GDP inflator be used (see 3.2.6 Transferability of costs across time, page 43) but we found that the data on this measure (from the World Bank) were sometimes sparse so we relied on the consumer price index instead (`'NY.GDP.DEFL.KD.ZG'` in the World Bank Development Indicator Database) [3].
- **Transferability of costs across settings** To 'borrow' data from other countries, we follow the 2003 WHO Guide to Cost-effectiveness recommendations in section 3.2.7 Transferability of costs across settings) [3]. For non-traded items (i.e. nurse and doctor time) we convert USD or LCU prices into PPP (international dollars) values in the year of the cost study and then turn the value in international dollars to local currency (still in the year of the study) of the country where a cost estimate is needed. Then, we use the CPI to inflate costs to 2024 levels and then use the exchange rate with USD to get 2024 USD values.
- **Combining multiple sources of information** Values from different publications are combined using meta-analytic methods.
- **Choice of probability distributions** Costs and ratios were modelled via gamma distributions and proportions or probability were modelled with beta distributions. These distributions were parameterized using the method of moments (see Briggs 2006 [4], Chapter 4).
- **Missing information on uncertainty: Gamma distributions.**

1. Option A: Whenever uncertainty was missing for a cost or a ratio in the literature, we assigned a gamma distribution for the parameter that would yield credible intervals between half and double the estimate.
2. Option B: If at least 2 studies listed a cost, then we take the range of the costs to parameterize a gamma distribution in which the range matched the 95 percent confidence interval (e.g. the 2.5th and 97.5th percentile). In these cases, we use a method to parameterize gamma distributions using quantiles rather than using the mean and standard error of a sample (e.g. "method of moments") [5].

- **Missing information on uncertainty: Beta distributions.**

1. Option A: Usually modeled assuming that 100 trials were observed with the **proportion_estimate x 100** as the alpha parameter and **(1-proportion_estimate) x 100** as a beta parameter.
2. Option B: If at least 2 studies listed a probability or a proportion, then we take the range of the costs to parameterize a beta distribution by assuming the range matches the 95 percent confidence interval (the 2.5th and 97.5th percentile). We use a method to parameterize Beta distributions using quantiles rather than the mean and standard error of a sample (method of moments) [6].

S4.2 Organization of parameters

In the code repository, found in https://osf.io/ezjxb/, the parameters are in an sql database: `parameters.sqlite3`.

Additionally, a list named `epi_output` (read in from Matlab output) holds the output from the dynamic model: stage 1 and stage 2 cases detected by passive and active screening, as well as person-time spent in stage 1 and 2 before detection.

S4.3 Summary of health outcome parameters

Below are all the parameters that model health outcomes, as well as a summary of their characteristics. An extended discussion of our choices is featured in the sections annotated in the table.

Variable description	Variable name	Statistical Distribution	Descriptive Summary	Notes
**Screening**				
Population	pop	Fixed value	Varies by health zone	See section S4.5.1
PS: coverage of the population per facility	ps_coverage	Beta(14, 2094)	0.007 (0.004, 0.010)	See section S4.5.2
PS: number of facilities	ps_facilities	Fixed value	Varies by health zone	See section S4.5.3
AS: coverage	as_traditional & as_traditional_int	Fixed value	Varies by health zone	See section S4.5.4
AS: capacity per team per year	as_capacity_traditional	Normal(60000, 10000)	60,055 (40,448, 79,471)	See section S4.5.5
CATT algorithm: diagnostic specificity	dx-spec-catt-1-in-8	Beta(4523, 22)	0.995 (0.993, 0.997)	See section S4.5.6
RDT algorithm: diagnostic sensitivity	dx-sens-rdt	Beta(230, 1)	1.00 (0.98, 1.00)	See section S4.5.7
RDT algorithm: diagnostic specificity	dx_spec_rdt	Beta(226, 31)	0.88 (0.84, 0.92)	See section S4.5.8
CATT algorithm: wastage during AS	dx_wastage_catt_as	Beta(8, 92)	0.08 (0.03, 0.14)	See section S4.5.9
RDT algorithm: wastage during PS	dx_wastage_rdt_ps	Beta(1, 99)	0.01 (<0.01, 0.04)	See section S4.5.10
**Treatment**				
Proportion of cases age<6	treat_prob_under6yo	Beta(152.53, 2427.9)	0.06 (0.05, 0.07)	See section S4.6.1
Proportion of cases weight<35 kg among age>6	treat_prob_under35kg	Beta(8.3, 359.6)	0.02 (<0.01, 0.04)	See section S4.6.2
Proportion of S2 cases that are severe	prob_late_stage2	Beta(76.93, 44.87)	0.63 (0.54, 0.72)	See section S4.6.3
Age of death from infection	age_of_death	Gamma(148, 0.18)	26.63 (22.41, 31.08)	See section S4.6.15
Length, treatment: pentamidine (days)	treat-duration-penta	Fixed value	7	See section S4.6.4
Length of hospital stay: NECT treatment	treat-duration-nect	Fixed value	10	See section S4.6.5
Length of hospital stay: fexinidazole treatment	treat-duration-fexi	Fixed value	10	See section S4.6.6
Pr. of relapse (treatment failure): pentamidine	treat_prob_failure_pent_s1	Beta(50.3, 665.48)	0.07 (0.05, 0.09)	See section S4.6.7
Pr. of relapse (treatment failure): NECT	treat_prob_failure_nect_s2	Beta(15.87, 378.55)	0.05 (0.02, 0.08)	See section S4.6.8
Pr. of relapse: fexinidazole	treat_prob_failure_fexi	Beta(9.49, 496.54)	0.02 (<0.01, 0.03)	See section S4.6.9
SAE: pentamidine treatment	treat-prob-sae-pent-s1	Beta(1.43, 551.42)	0.002 (<0.001, 0.008)	See section S4.6.10
SAE: NECT treatment	treat-prob-sae_nect_s2	Beta(40.88, 367.8)	0.05 (0.03, 0.08)	See section S4.6.11
SAE: fexinidazole treatment	treat_prob_sae_fexi	Beta(3, 261)	0.01 (<0.01, 0.03)	See section S4.6.12
Days lost to disability: due to SAE	treat_duration_sae	Gamma(1.22, 2.38)	2.96 (0.14, 9.99)	See section S4.6.13
**Life-years lost (DALY)**				
Life expectancy	life_expectancy	Fixed values, interpolated	Varies	See section S4.6.16
Disability weights: S1 disease	disability_weighting_s1	Beta(22.96, 147.21)	0.14 (0.09, 0.19)	See section S4.6.17
Disability weights: S2 disease	disability_weighting_s2	Beta(18.37, 15.63)	0.54 (0.37, 0.70)	See section S4.6.18
Disability weights: SAE	disability_weighting_sae	Uniform(0.04, 0.11)	0.08 (0.04, 0.11)	See section S4.6.19
**Vector control**				
Linear km of targets	vc_length_default & vc_length_enhanced	Fixed value	Varies by health zone	See section S4.7.1
Targets per km	vc_density_linear	Fixed value	Varies by health zone	See section S4.7.2
Replacement rate of targets per year	vc_deployments_yr	Fixed value	2	See section S4.7.3

Table A: Health outcome parameters

S4.4 Summary of cost parameters

Below are all the cost parameters and a summary of their characteristics in three tables for screening, treatment, and vector control costs. An extended discussion of our choices is featured in the sections annotated in the tables.

Variable description	Variable name	Statistical Distribution	Descriptive Summary	Notes
**Screening**				
AS: capital costs of a team	`as_cost_team_capital`	Gamma(820, 20.1)	16,514 (15,410, 17,671)	See section S4.8.1
AS: fixed management costs of a team	`as_cost_team_management`	Gamma(4154, 17.21)	71,472 (69,311, 73,658)	See section S4.8.2
CATT algorithm: cost per test used	`dx_cost_catt`	Gamma(1140, 0.0006897)	0.79 (0.74, 0.83)	See section S4.8.3
Staging: lumbar puncture & lab exam	`dx_cost_lumbar_exam`	Gamma(3.73, 2.96)	13.76 (3.37, 31.08)	See section S4.8.4
Confirmation: microscopy	`dx_cost_microscopy`	Gamma(8.47, 1.27)	10.68 (4.70, 18.84)	See section S4.8.5
RDT algorithm: costs per test used	`dx_cost_rdt`	Gamma(1140, 0.002205)	$2.51 (2.37, 2.66)	See section S4.8.6
Indirect management costs (PNLTHA mark-up)	`program-markup`	Uniform(0.1, 0.2)	0.15 (0.10, 0.20)	See section S4.8.7
PS: capital costs of a facility	`ps_cost_facility_capital`	Gamma(8.47, 226)	2393 (1067, 4269)	See section S4.8.8
PS: management costs	`ps_cost_management`	Gamma(8.47, 145)	1223 (560, 2157)	See section S4.8.9
**Treatment**				
Hospital stay: cost per day	`treat_cost_ip_day`	Gamma(5.81, 0.59)	1.39 (0.50, 2.71)	See section S4.9.1
Outpatient consultation: cost	`treat_cost_op_visit`	Gamma(24.2, 0.13)	3.13 (2.02, 4.47)	See section S4.9.2
Course of pentamidine: cost	`rx_cost_pentamidine`	Fixed value	54	See section S4.9.3
Course of NECT: cost	`rx_cost_nect`	Fixed value	360	See section S4.9.4
Course of fexinidazole: cost	`rx_cost_fexinidazole`	Fixed value	50	See section S4.9.5
Drug delivery mark-up	`rx_delivery_markup`	Beta(45, 55)	0.45 (0.35, 0.55)	See section S4.9.6
**Vector control**				
Operational cost per kilometer of riverbank covered	`vc_cost_management`	Gamma(8.47, 57.7)	491 (217, 869)	See section S4.10.1
Deployment cost per target	`vc_cost_deployment`	Gamma(8.47, 0.50)	4.21 (1.88, 7.50)	See section S4.10.2

Table B: Cost parameters

S4.5 Screening parameters

S4.5.1 Population

↩ Return to the Summary of Health Outcome Parameters.

- Name in the code: `pop`
- Source: [7]
- Country of estimate: DRC
- Statistical distribution and parameters: Fixed value
- Summary statistics (mean and 95% CI or fixed value): Varies by health zone

Notes

Our population data comes from the United Nations Office for the Coordination of Humanitarian Affairs censuses for national vaccine days [7]. Determined by taking the population from [7] and assuming a 3% population growth. See population summaries for the health zones included and excluded in Table B in S1 Text. In Supplementary Note 2 (S3 Text), we provide the population for each health zone.

S4.5.2 PS: coverage of the population per facility

↩ Return to the Summary of Health Outcome Parameters.

- Name in the code: `ps_coverage`
- Source: PNLTHA administrative data.
- Country of estimate: DRC
- Statistical distribution and parameters: Specific per coordination, see table below.
- Summary statistics (mean and 95% CI or fixed value): Varies by coordination, see table below.

Notes

For a summary of the clinics per coordination and nationwide, see Table I in S1 Text, Section S1.3. Data from 2019-2021 comes from the national program, and for 2022 from the annual report.

	Kongo Central	Bandund Nord	Bandund Sud	Equateu Nord	Equateu Sud	Kasai Oriental	Kasai Occidental	Maniema - Katanga	Kinshasa	Isangi	Sankuru	Whole
**Clinics in 2019 WHO Survey**												
Clinics	81	169	111	28	12	54	17	9	24	16	7	528
**Screened**												
2019	7,187	98,589	23,167	179,984	14,282	35,371	26,623	18,043	5,355	9,473	8,006	426,080
2020	27,079	151,914	23,915	141,817	5,831	5,985	24,290	13,932	3,028	6,391	11,739	415,912
2021	4	104,309	22,342	244,177	3,743	13,836	13,418	9,589	3,162	12,560	8,086	435,226
2022	10,313	88,789	276,553	762	5,461	12,077	24,951	4,921	3,000	6,819	5,889	459,535
**Population in health zones with clinics (millions)**												
2019	2.27	3.16	4.14	3.23	1.14	4.74	2.55	1.49	2.45	0.54	0.75	26.47
2020	2.34	3.26	4.27	3.32	1.18	4.88	2.63	1.53	2.52	0.55	0.78	27.26
2021	2.41	3.36	4.39	3.42	1.21	5.03	2.71	1.58	2.60	0.57	0.80	28.08
2022	2.48	3.46	4.53	3.53	1.25	5.18	2.79	1.63	2.68	0.59	0.82	28.92
**Screened per site**												
2019	89	583	209	6,428	1,190	655	1,566	2,005	223	592	1,144	807
2020	334	899	215	5,065	486	111	1,429	1,548	126	399	1,677	788
2021	0	617	201	8,721	312	256	789	1,065	132	785	1,155	824
2022	127	525	2,491	27	455	224	1,468	547	125	426	841	870
**Screened per site per 10K pop**												
2019	7	33	10	438	94	30	110	175	12	77	121	51
2020	24	50	10	335	37	5	98	131	6	50	173	48
2021	0	33	9	561	23	11	52	88	7	96	116	49
2022	9	27	105	2	33	9	95	44	6	51	82	50
**Mean and standard error in 2019-2022**												
Mean	10	36	33	334	47	14	89	109	8	69	123	49
St. Err.	5	5	24	120	16	6	13	28	1	11	19	1
**Distribution simulated**												
Shape	3.72	55.53	1.93	7.75	8.58	6.24	49.74	15.00	32.09	38.06	42.70	6,860.98
Rate	0.37	1.55	0.06	0.02	0.18	0.45	0.56	0.14	4.14	0.55	0.35	139.12
Summary	9 (3, 22)	36 (27, 46)	28 (4, 93)	320 (142, 607)	45 (21, 83)	13 (5, 27)	88 (66, 115)	107 (61, 171)	8 (5, 11)	68 (49, 92)	122 (89, 162)	49 (48, 50)

Table C: People screened in passive screening, 2019-22, and the parameters for simulations for the future. In the analysis, both Isangi - Tschopo and Isangi - Bas-Uelé will have the same parameters. The summary shows the median and 95% confidence intervals of the distribution used to simulate the coverage in the future.

S4.5.3 PS: number of facilities

↩ Return to the Summary of Health Outcome Parameters.

- Name in the code: `ps_facilities`
- Source: [8–10]; see Table I in S1 Text for summary by coordination.
- Country of estimate: DRC
- Statistical distribution and parameters: Fixed value
- Summary statistics (mean and 95% CI or fixed value): Varies by health zone

Notes

These values were retrieved from Simarro et. al. (see the supplement) and WHO surveys [8, 9]. These are facilities that can perform serological tests (CATT or RDT), microbiological confirmation, and/or treatment. In Supplementary Note 2, we provide the number of facilities for each health zone.

S4.5.4 AS: coverage

↩ Return to the Summary of Health Outcome Parameters.

- Name in the code: `as_traditional` & `as_traditional_int`
- Source: HAT Atlas data
- Country of estimate: DRC
- Statistical distribution and parameters: Fixed value
- Summary statistics (mean and 95% CI or fixed value): Varies by health zone

Notes

The percent of the population that is screened by mobile teams in their villages each year. These were determined by the average percent of the population in each health zone that was screened over the years 2016-2020 (for *Mean AS*) and the maximum that was screening over the years in 2000-2020 (for *Int. AS*). See Supplementary Note 2 for the number of AS people screened per health zone.

S4.5.5 AS: capacity per team per year

↩ Return to the Summary of Health Outcome Parameters.

- Name in the code: `as_traditional_capacity`
- Source: [11–13]
- Country of estimate: DRC
- Statistical distribution and parameters: Normal(60,000, 10,000)
- Summary statistics (mean and 95% CI or fixed value): 60,055 (40,448, 79,471)

Notes

The capacity of an active screening team in DRC has a mean of 60,000, a lower bound of 40,000 [11] and an upper bound of 80,000 with a work year of 220 days [13]. Teams are managed by the coordination and serve a span of multiple health zones and dozens of villages.

To parameterize the model, we chose a normal distribution with upper and lower bounds of 40-80 thousand people, therefore the parameters are Normal(60,000, 10,000).

S4.5.6 CATT 1:8 algorithm: diagnostic specificity

↩ Return to the Summary of Health Outcome Parameters.

- Name in the code: `dx_spec_catt_1_in_8`
- Source: [14]
- Country of estimate: Various
- Statistical distribution and parameters: Beta(4523, 22)

- Summary statistics (mean and 95% CI or fixed value): 0.995 (0.993, 0.997)

Notes

Lumbala *et al*'s 2017 publication, which reported a CATT 1:8 specificity of 99.5% [99.3%, 99.7%] for active screening in the DRC. This was in line with their 2018 publication which reported a specificity of 99.5% [99.5%; 99.6%] in active screening and 97.6% [97.3%; 97.9%] for passive screening in the DRC. The beta distribution that corresponds with that (99.3, 99.7) as the 95% confidence interval is Beta(4523, 22).

As a form of validation, in a screening of 1.4M people (the total in 2022), that would equal 7,000 [4,200–9,800] false positives. In 2022, there were 7195 seropositives in AS, mostly done with CATT testing (Annual Report).

S4.5.7 RDT algorithm: diagnostic sensitivity

↩ Return to the Summary of Health Outcome Parameters.

- Name in the code: `dx_sens_rdt`
- Source: [15]
- Country of estimate: Guinea and Côte d'Ivoire
- Statistical distribution and parameters: Beta(230, 1)
- Summary statistics (mean and 95% CI or fixed value): 1.00 (0.98, 1.00)

Notes

Based on a study in Guinea and Côte d'Ivoire [15], there was 1 sample from a gHAT patient that tested negative out of 231. Therefore, the parameter distribution for specificity is Beta(230, 1).

S4.5.8 RDT algorithm: diagnostic specificity

↩ Return to the Summary of Health Outcome Parameters.

- Name in the code: `dx_spec_rdt`
- Source:
- Country of estimate: Guinea and Cote d'Ivoire
- Statistical distribution and parameters: Beta(1134, 11)
- Summary statistics (mean and 95% CI or fixed value): 0.990 (0.984, 0.995)

Notes

Over the last 5 years, the positivity rate for RDTs in PS is between 98.40 (in 2018, Fourth Stakeholders' meeting report) to 99.52 in 2022 (Annual Report). We have assigned that range as the 95% confidence interval of a beta distribution describing the specificity of the RDTs. When we derived a Beta distributions with that 95% confidence interval, we arrived at parameters of (1134, 11), or a distribution of 0.990 (0.984, 0.995).

Previous studies assigned a lower specificity, but the positivity rate would indicate specificity is now better. Based on a study in Guinea and Côte d'Ivoire [15], there were 31 samples from non-HAT patients that tested positive out of 257. Therefore, the parameter distribution for specificity is Beta(226, 31), or a specificity of 88% (95% CI: 84-92%). However, if we consider that about 400,000 people are tested in PS and a false positivity rate of 12% (the complement of the specificity), then that means that we have to confirm 48,000 patients. In 2018, 531,863 people were tested in DRC and only 8,485 RDT-positives were identified (for microscopic confirmation), and in 2022 459,535 people were tested and only 2181 people came out positive.

S4.5.9 CATT algorithm: wastage during AS

↩ Return to the Summary of Health Outcome Parameters.

- Name in the code: `dx_wastage_catt_as`
- Source: [13]
- Country of estimate: DRC
- Statistical distribution and parameters: Beta(8, 92)
- Summary statistics (mean and 95% CI or fixed value): 0.08 (0.03, 0.14)

Notes

CATT tests come in packs of 50, and the list cost is assumed to consider that a pack is used on 50 patients. Once a pack is opened, one test is used as a positive control and one test is used as a negative control, so wastage is at least 4 percent. The shelf life of the test is one week in refrigeration and wastage in active screening activities is relatively low; generally, wastage of CATT tests in the context of active screening occurs at the end of the day when there are tests remaining in an open pack. To be conservative, we doubled the 4-percent lower bound for wastage and assigned the parameter a distribution of Beta(8, 92).

S4.5.10 RDT algorithm: wastage during PS

↩ Return to the Summary of Health Outcome Parameters.

- Name in the code: `dx_wastage_rdt_ps`
- Source: [16]
- Country of estimate: DRC
- Statistical distribution and parameters: Beta(1, 99)
- Summary statistics (mean and 95% CI or fixed value): 0.01 (<0.01, 0.04)

Notes

We followed the same assumption as Snijders and colleagues that less than 1 percent of RDT tests would not be used [16]. Because there was no sense of uncertainty in this parameter, we assumed a Beta (1,99) distribution.

S4.6 Treatment parameters

S4.6.1 Proportion of cases age<6

↩ Return to the Summary of Health Outcome Parameters.

- Name in the code: `treat_prob_under6yo`
- Source: [17, 18]
- Country of estimate: South Sudan
- Statistical distribution and parameters: Beta(152.53, 2427.9)
- Summary statistics (mean and 95% CI or fixed value): 0.06 (0.05, 0.07)

Notes

There were only two studies where the number of children under 5 or 6 years of age was stated explicitly [17, 18].

Study	Groups	Under 6 y.o.	Total	Est.	95% CI
Schmid 2012	Single arm	35	629	0.06	(0.04−0.08)
Eperon 2006	Pentamidine	56	850	0.07	(0.05−0.08)
Eperon 2006	Melarsoprol	62	1108	0.06	(0.04−0.07)
**Common effect model**			**2587**	**0.06**	**(0.05−0.07)**
**Random effects model**				**0.06**	**(0.05−0.07)**

Heterogeneity: $I^2 = 0\%$, $\tau^2 = 0$, $p = 0.60$

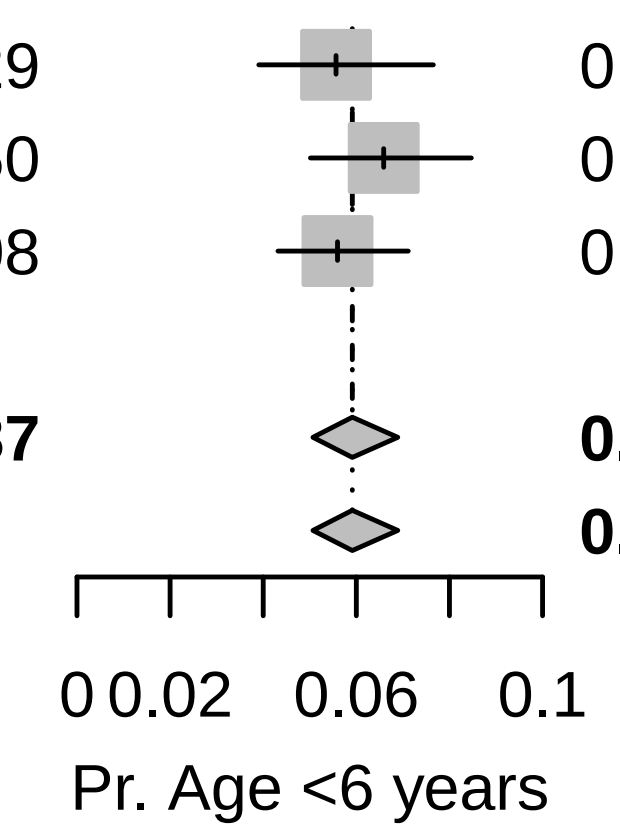

Because the data showed non-significant heterogeneity according to the tau-squared test for heterogeneity, we have chosen to use the fixed-effects combined estimate: 0.059 (0.051, 0.069). The beta parameters of the random-effects estimate Beta(153.53, 2427.90) for the probability of that a patient is under 6 years old.

Citation	Group	Age Group	Mean weight	Measure of spread	No. of observations	Gamma distr. alpha par.	Gamma distr. beta par.	Prop. <35kg	Simulated No. <35kg
Priotto 2006	Melarsoprol and Nifurtimox	All ages	49.20	SD = 14.4	18	11.67	4.21	0.16	3
Priotto 2006	Melarsoprol and Eflornithine	All ages	50.00	SD = 10.3	19	23.56	2.12	0.06	1
Priotto 2006	NECT	All ages	51.40	SD = 8.4	17	37.44	1.37	0.02	0
Priotto 2007	NECT	Over 15 years old	51.70	SD = 7.4	52	48.81	1.06	0.01	0
Priotto 2007	Eflornithine	Over 15 years old	53.10	SD = 7.2	51	54.39	0.98	0.00	0
Checchi 2007	NECT	All ages	44.80	SD = 15.1	31	8.80	5.09	0.28	9
Priotto 2009	NECT	Over 15 years old	53.00	SD = 8.7	143	37.11	1.43	0.01	2
Priotto 2009	Eflornithine	Over 15 years old	53.90	SD = 8.3	143	42.17	1.28	0.01	1
Ngoyi 2010	Pentamidine and Melarsoprol	Over 12 years old	56.00	SD = 10.0	360	31.36	1.79	0.01	3
Priotto 2012	Single arm	All ages	49.00	IQR: 40-56	2190	16.37	2.96	0.12	265
Schmid 2012	Single arm	All ages	45.00	SD = 16.0	629	7.91	5.69	0.29	182
Burri 2016	Pentamidine	Over 15 years old and >35 kg	48.50	SD = 7.6	40	40.83	1.19	0.03	1
Pohlig 2016	Pentamidine	Over 12 years old and >30 kg	45.70	SD = 7.8	137	34.15	1.34	0.08	10
Pohlig 2016	Pafuramidine	Over 12 years old and >30 kg	44.70	SD = 7.9	136	32.02	1.40	0.10	14
Kansiime 2018	All	Over 15 years old	51.69	SD = 9.7	109	28.22	1.83	0.03	3
Mesu 2018	NECT	Over 15 years old	50.70	SD = 9.6	130	27.89	1.82	0.04	5
Mesu 2018	Fexinidazole	Over 15 years old	50.50	SD = 8.2	264	37.93	1.33	0.02	5

S4.6.2 Proportion of cases weight<35 kg among age>6

↩ Return to the Summary of Health Outcome Parameters.

- Name in the code: `treat_prob_under35kg`
- Source: [18–27]
- Country of estimate: Various
- Statistical distribution and parameters: Beta(8.3, 359.6)
- Summary statistics (mean and 95% CI or fixed value): 0.02 (<0.01, 0.04)

Notes

To determine whether a patient is eligible for fexinidazole treatment, we could not find any studies that would tell us the number of HAT patients who weighed less than 35 kg, but we have estimated the number of people who might weigh less than 35 kg by examining the distribution of weight among patients in the trials in the literature. Furthermore, we have examined how this variable is related to potential selection by age of the study population. We are interested in the proportion of older children and adults that might weigh less than 35 kg, as age under 6 is a contraindication for fexinidazole.

We fit a gamma distribution by the method of moments to the reported mean and standard deviations of each of the studies. For Priotto 2012, no SD was reported, but an interquartile range was reported, so we fit a gamma distribution by the method of Cook [5].

We then took the expected number of people under 35 kg, and then performed a single-proportion meta-analysis with the expected number of people in each study under and over the 35 kg threshold.

Study	Arm	Events	Total	Est.	95% CI
**all_ages_lbl = Adolescents and adults**					
Priotto 2007	NECT	0	52	0.00	(0.00–0.07)
Priotto 2007	Eflornithine	0	51	0.00	(0.00–0.07)
Priotto 2009	NECT	2	143	0.01	(0.00–0.05)
Priotto 2009	Eflornithine	1	143	0.01	(0.00–0.04)
Ngoyi 2010	Pentamidine and Melarsoprol	3	360	0.01	(0.00–0.02)
Burri 2016	Pentamidine	1	40	0.03	(0.00–0.13)
Pohlig 2016	Pentamidine	10	137	0.07	(0.04–0.13)
Pohlig 2016	Pafuramidine	14	136	0.10	(0.06–0.17)
Kansiime 2018	All	3	109	0.03	(0.01–0.08)
Mesu 2018	NECT	5	130	0.04	(0.01–0.09)
Mesu 2018	Fexinidazole	5	264	0.02	(0.01–0.04)
**Common effect model**			**1565**	**0.03**	**(0.02–0.04)**
**Random effects model**				**0.02**	**(0.01–0.04)**
Heterogeneity: $I^2 = 70\%$, $\tau^2 = 0.8$, $p < 0.01$					
**all_ages_lbl = All ages**					
Priotto 2006	Melarsoprol and Nifurtimox	3	18	0.17	(0.04–0.41)
Priotto 2006	Melarsoprol and Eflornithine	1	19	0.05	(0.00–0.26)
Priotto 2006	NECT	0	17	0.00	(0.00–0.20)
Checchi 2007	NECT	9	31	0.29	(0.14–0.48)
Priotto 2012	Single arm	265	2190	0.12	(0.11–0.14)
Schmid 2012	Single arm	182	629	0.29	(0.25–0.33)
**Common effect model**			**2904**	**0.16**	**(0.15–0.17)**
**Random effects model**				**0.15**	**(0.08–0.27)**
Heterogeneity: $I^2 = 95\%$, $\tau^2 = 0.4$, $p < 0.01$					

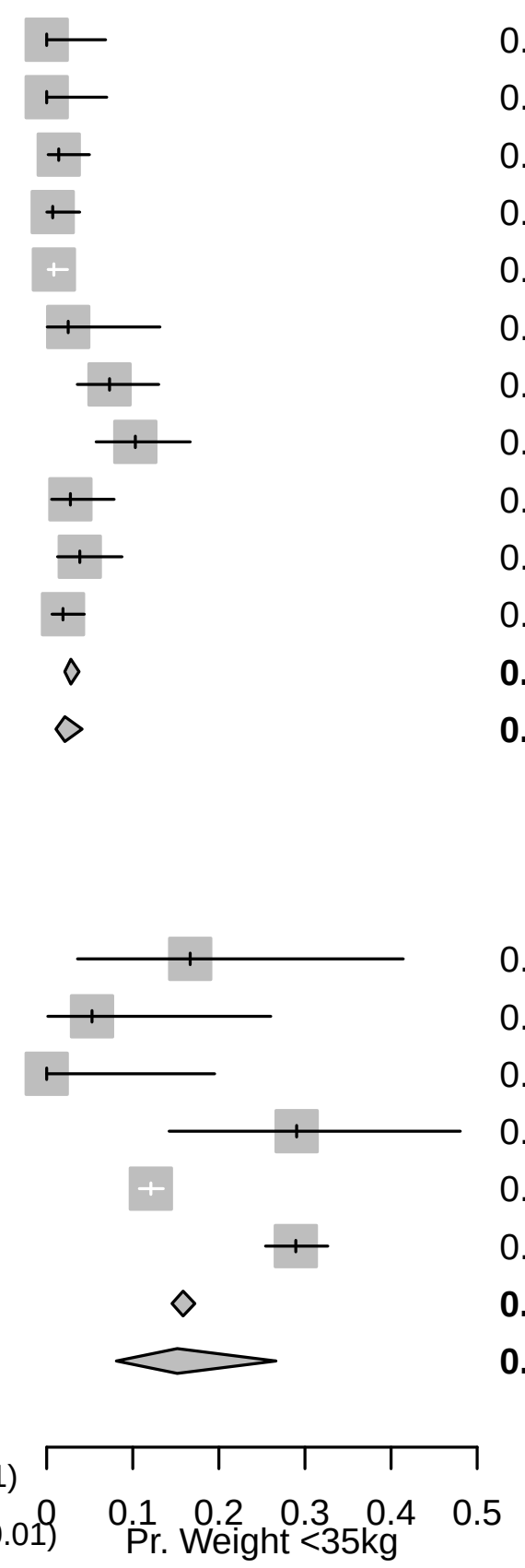

Test for subgroup differences (fixed effect): $\chi^2_1 = 135.06$, df = 1 ($p < 0.01$)
Test for subgroup differences (random effects): $\chi^2_1 = 17.61$, df = 1 ($p < 0.01$)

Because the data showed significant heterogeneity according to the tau-squared test for heterogeneity, we have chosen to use the random-effects estimate: 0.02 (0.01-0.04), represented by probability distribution: Beta(8.30, 359.61).

S4.6.3 Proportion of S2 cases that are severe

↩ Return to the Summary of Health Outcome Parameters.

- Name in the code: `prob_late_stage2`
- Source: [17–23, 28]
- Country of estimate: Various
- Statistical distribution and parameters: Beta(76.93, 44.87)
- Summary statistics (mean and 95% CI or fixed value): 0.63 (0.54, 0.72)

Notes

The definition of severe stage 2 gHAT disease by the WHO is when there are more than 100 white blood cells (WBC, leukocytes) per micro-litre in the cerebrospinal fluid. We have searched the clinical trials for the proportion of stage 2 patients that have high concentrations of leukocytes upon admission to treatment.

Study	Arm	Events	Total	Est.	95% CI
Priotto 2006	Melarsoprol and Nifurtimox	9	18	0.50	(0.26−0.74)
Priotto 2006	Melarsoprol and Eflornithine	12	19	0.63	(0.38−0.84)
Priotto 2006	NECT	6	17	0.35	(0.14−0.62)
Eperon 2006	Melarsoprol	407	1108	0.37	(0.34−0.40)
Priotto 2007	NECT	31	52	0.60	(0.45−0.73)
Priotto 2007	Eflornithine	39	51	0.76	(0.63−0.87)
Checchi 2007	NECT	18	31	0.58	(0.39−0.75)
Priotto 2009	NECT	107	143	0.75	(0.67−0.82)
Priotto 2009	Eflornithine	115	143	0.80	(0.73−0.87)
Ngoyi 2010	Pentamidine and Melarsoprol	209	272	0.77	(0.71−0.82)
Priotto 2012	Single arm	1373	2190	0.63	(0.61−0.65)
Schmid 2012	Single arm	397	629	0.63	(0.59−0.67)
**Common effect model**			**4673**	**0.58**	**(0.57−0.60)**
**Random effects model**				**0.63**	**(0.55−0.71)**

Heterogeneity: $I^2 = 96\%$, $\tau^2 = 0.3$, $p < 0.01$

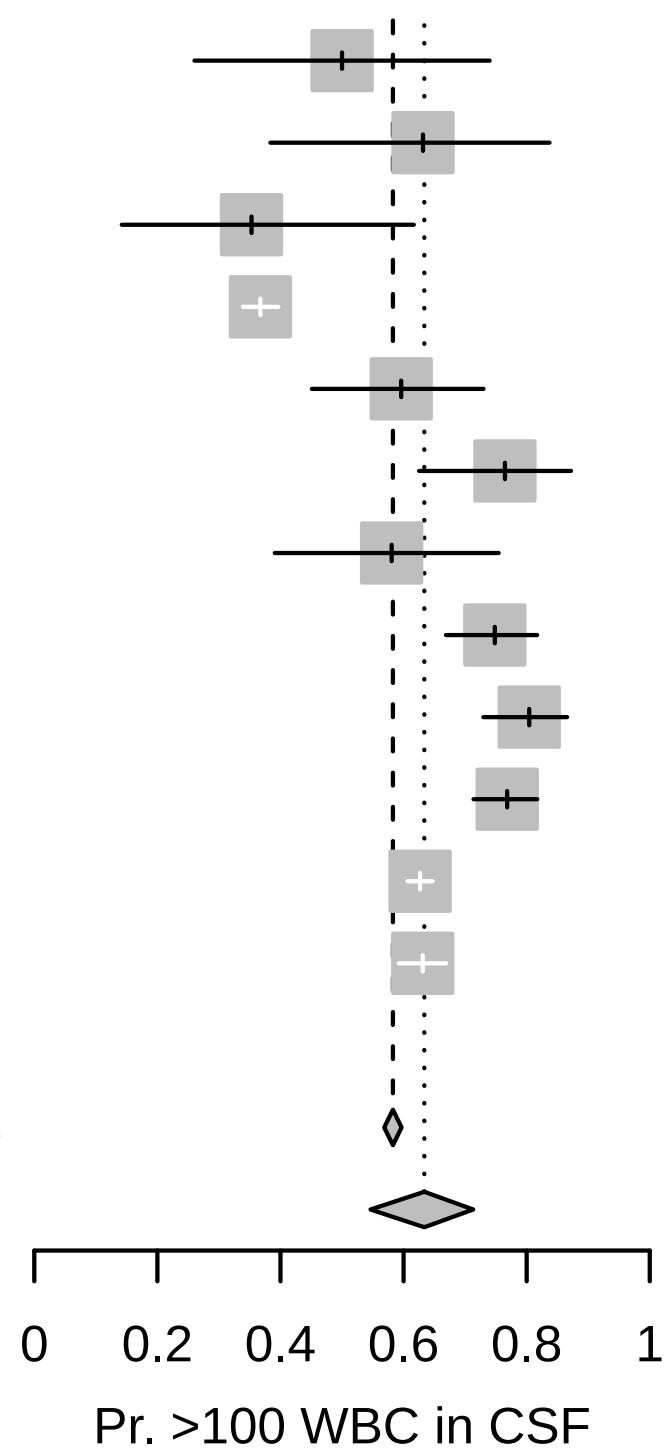

Because the data showed significant heterogeneity according to the tau-squared test for heterogeneity, we have chosen to use the random-effects combined estimate: 0.634 (0.546, 0.713), represented by the probability distribution Beta(76.93, 44.87).

S4.6.4 Length of hospital stay: Pentamidine treatment

↩ Return to the Summary of Health Outcome Parameters.

- Name in the code: `treat_duration_penta`
- Source: [28]
- Country of estimate: Global recommendations
- Statistical distribution and parameters: Fixed value
- Summary statistics (mean and 95% CI or fixed value): 7

Notes

For NECT treatment, patients must stay in inpatient care for a minimum of 7 days for the eflornithine infusions, and whether they stay for a total of 10 days for nifurtimox administration is unclear. For the most recent clinical trial [27], NECT patients were released on days 13-18 after admission, but we have assumed that for the most recent trial, the average patient can be released from care after 10 days in the hospital.

S4.6.5 Length of hospital stay: NECT treatment

↩ Return to the Summary of Health Outcome Parameters.

- Name in the code: `treat_duration_nect`
- Source: [27, 28]
- Country of estimate: Global recommendations
- Statistical distribution and parameters: Fixed value
- Summary statistics (mean and 95% CI or fixed value): 10

Notes

For NECT treatment, patients must stay in inpatient care for a minimum of 7 days for the eflornithine infusions, and whether they stay for a total of 10 days for nifurtimox administration is unclear. For the most recent clinical trial [27], NECT patients were released on days 13-18 after admission, but we have assumed that for the most recent trial, the average patient can be released from care after 10 days in the hospital.

S4.6.6 Length of hospital stay: fexinidazole treatment

↩ Return to the Summary of Health Outcome Parameters.

- Name in the code: `treat_duration_fexi`
- Source: [27, 28]
- Country of estimate: Global recommendations
- Statistical distribution and parameters: Fixed value
- Summary statistics (mean and 95% CI or fixed value): 10

Notes

For the only trial that is published [27], patients were released on days 13-18 after the initiation of treatment, although the treatment only took 10 days, so we have assumed that in routine care the average patient will be in inpatient care for 10 days.

S4.6.7 Pr. of relapse: pentamidine

↩ Return to the Summary of Health Outcome Parameters.

- Name in the code: `treat_prob_failure_pent_s1`
- Source: [17, 23–25, 29, 30]
- Country of estimate: Various
- Statistical distribution and parameters: Beta(50.3, 665.48)
- Summary statistics (mean and 95% CI or fixed value): 0.07 (0.05, 0.09)

Notes

The Appendix to the WHO guidelines for the treatment of HAT in 2019 [28] presented existing data on treatment failure of pentamidine treatment (a briefer, updated guideline was published in 2024 [31], but without this list of studies, hence we refer to the interim guidelines). To produce one comprehensive estimate of treatment failure, we performed a meta-analysis on proportions within a single group.

Study	Events	Total	Estimate	95% CI
Balasegaram 2006	33	692	0.05	(0.03−0.07)
Eperon 2006	37	652	0.06	(0.04−0.08)
Ngoyi 2010	6	39	0.15	(0.06−0.31)
Bastide 2011	368	4597	0.08	(0.07−0.09)
Burri 2016	1	32	0.03	(0.00−0.16)
Pohlig 2016	14	137	0.10	(0.06−0.17)
**Common effect model**		**6149**	**0.07**	**(0.07−0.08)**
**Random effects model**			**0.07**	**(0.05−0.09)**

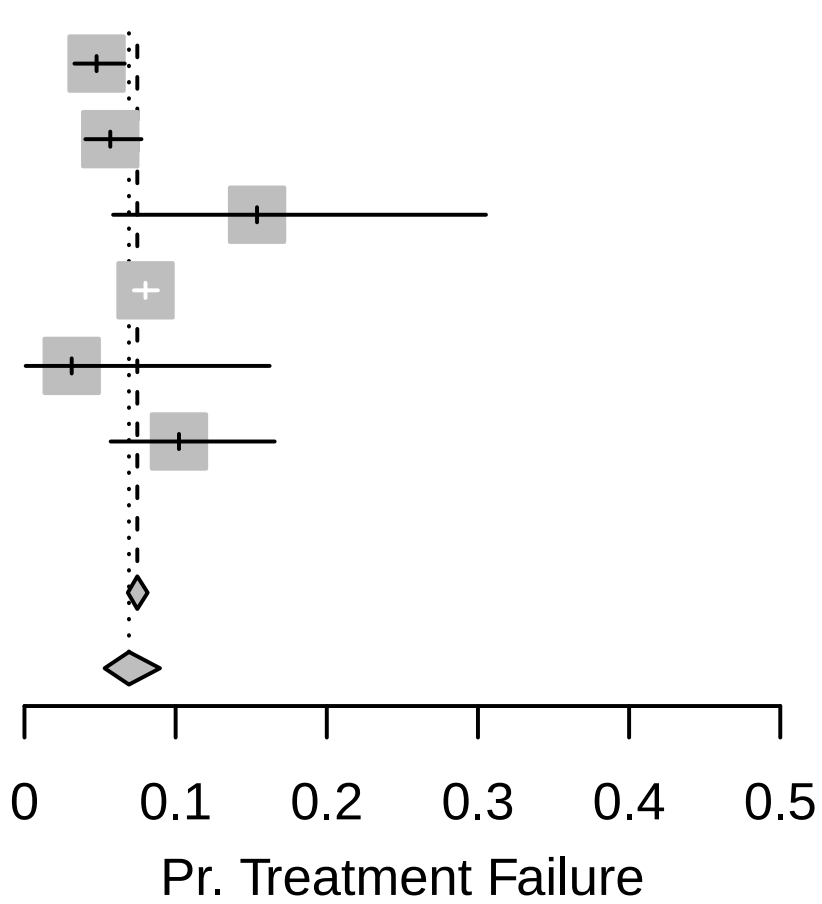

Heterogeneity: $I^2 = 72\%$, $\tau^2 < 0.1$, $p < 0.01$

Because the data showed significant heterogeneity according to the tau-squared test for heterogeneity, we have chosen to use the random-effects estimate, to which we assigned a distribution of Beta(50.30, 665.47).

S4.6.8 Pr. of relapse (treatment failure): NECT

↩ Return to the Summary of Health Outcome Parameters.

- Name in the code: `treat_prob_failure_nect_s2`
- Source: [19–22, 26, 27]
- Country of estimate: Various
- Statistical distribution and parameters: Beta(15.87, 378.55)
- Summary statistics (mean and 95% CI or fixed value): 0.05 (0.02, 0.08)

Notes

The WHO guidelines for the treatment of HAT in 2019 [28] presented existing data on treatment failure of NECT (a briefer, updated guideline was published in 2024 [31], but without this list of studies, hence we refer to the interim guidelines). Kansiime and colleagues [26] also performed a systematic review of studies estimating the outcomes of NECT treatment. To produce one comprehensive estimate of treatment failure, we performed a meta-analysis on proportions within single groups.

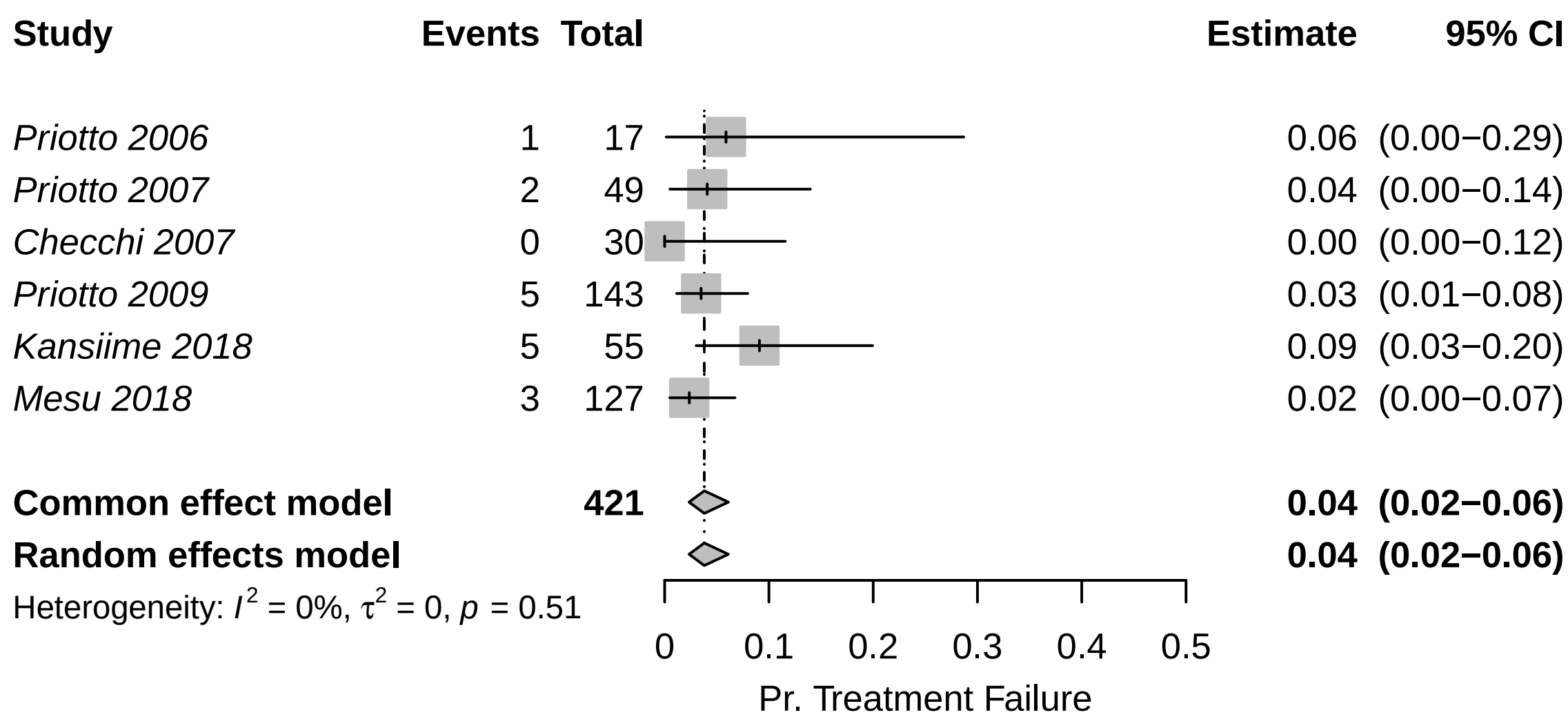

Because the data showed non-significant heterogeneity according to the tau-squared test for heterogeneity, we have chosen to use the fixed-effects estimate, to which we assigned a distribution of Beta(15.87, 378.55)

S4.6.9 Pr. of relapse: fexinidazole

↩ Return to the Summary of Health Outcome Parameters.

- Name in the code: `treat_prob_failure_fexi`
- Source: [28]
- Country of estimate: DRC
- Statistical distribution and parameters: Beta(9.49, 496.54)
- Summary statistics (mean and 95% CI or fixed value): 0.02 (<0.01, 0.03)

Notes

Mesu and colleagues [27] have published the only study on fexinidazole treatment effectiveness in late-stage 2 cases. Moreover, the accompanying meta-analysis for the WHO treatment guidelines released in 2019 shows the outcomes of an additional extension study on stage 1, both early and late-stage 2 disease as well for the data from Mesu et al stratified by the concentration of WBC in the CSF [31].

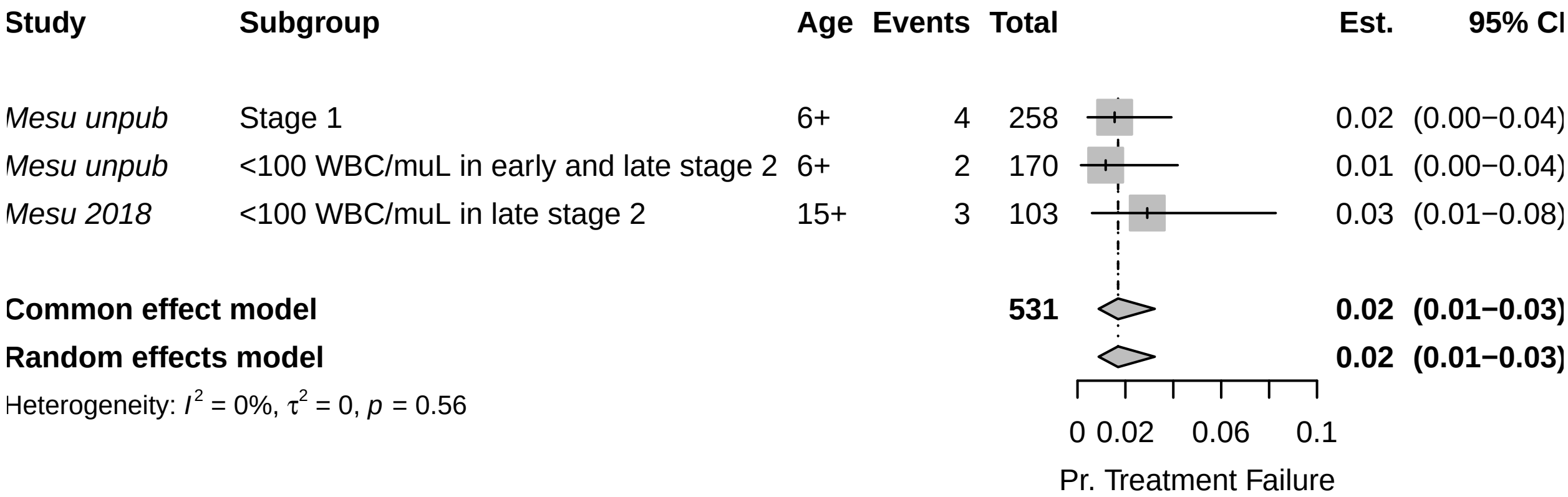

Because the data showed non-significant heterogeneity according to the tau-squared test for heterogeneity, we have chosen to use the fixed-effects combined estimate: 0.017 (0.009, 0.032), for which we assigned a distribution of Beta(9.49, 496.54).

S4.6.10 SAE: pentamidine treatment

↩ Return to the Summary of Health Outcome Parameters.

- Name in the code: `treat_prob_sae_pent_s1`
- Source: [17, 24, 25]
- Country of estimate: DRC and South Sudan
- Statistical distribution and parameters: Beta(1.43, 551.42)
- Summary statistics (mean and 95% CI or fixed value): 0.002 (<0.001, 0.008)

Notes

As part of the WHO guidelines for the treatment of HAT in 2019 ([28]), Cochrane performed a systematic review of studies that evaluated the efficacy of NECT compared to fexinidazole studies and presented the probability of serious adverse events. A briefer, updated guideline was published in 2024, but without this list of studies, hence we refer to the interim guidelines [31]. Severe or serious adverse events in studies for S1 treatment were defined as "significant hazard, contra-indication, side effect, or precaution" [17, 24, 25].

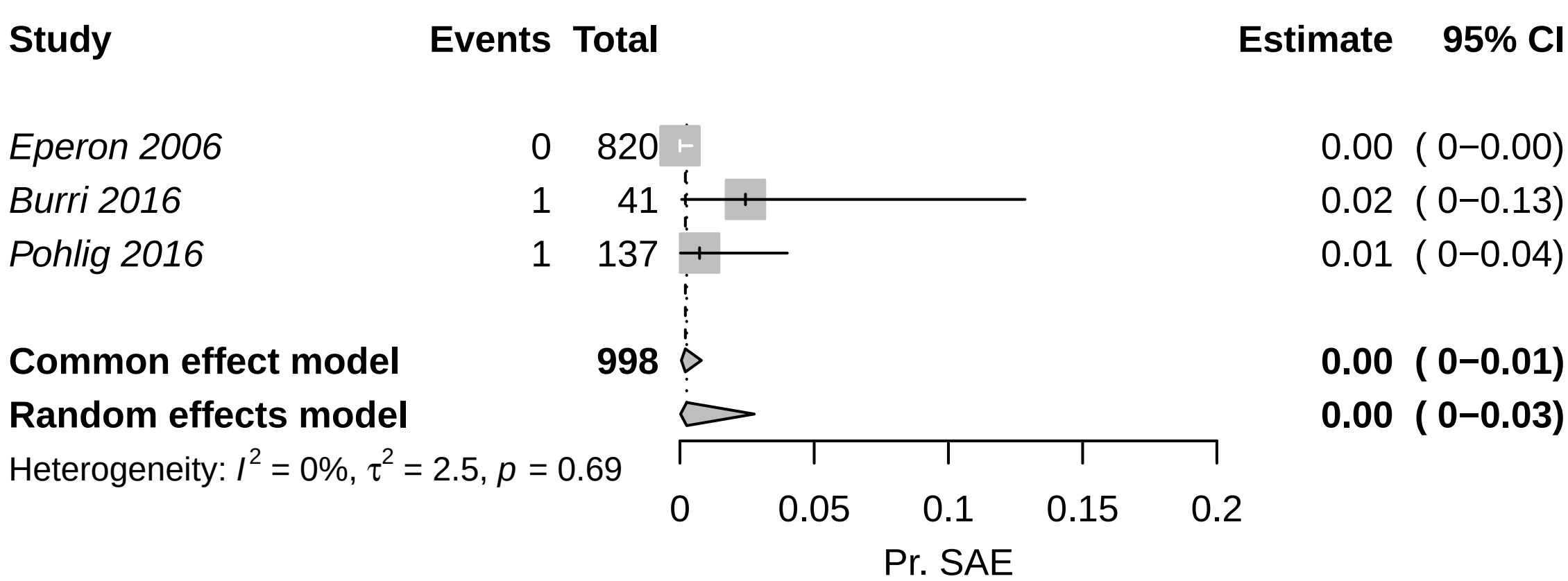

Because the results do not contain evidence of significant heterogeneity, we have chosen to use the fixed (pooled) estimate of 0 (0-0.01), which would result from a beta distribution of Beta(1.43, 551.42).

S4.6.11 SAE: NECT treatment

↩ Return to the Summary of Health Outcome Parameters.

- Name in the code: `treat_prob_sae_nect_s2`
- Source: [19–22, 26, 27]
- Country of estimate: DRC
- Statistical distribution and parameters: Beta(40.88, 367.8)
- Summary statistics (mean and 95% CI or fixed value): 0.05 (0.03, 0.08)

Notes

The WHO guidelines for treatment of gHAT in 2019 [28], presented all NECT studies to date, as did Kansiime and colleagues [26]. A briefer, updated guideline was published in 2024, but without this list of studies, hence we refer to the interim guidelines [31]. We searched through these studies for evidence of the probability of severe adverse vents (SAEs), described as events of Grade 3 or higher according to the National Cancer Institute Common Toxicity Criteria for Adverse Events (CTCAE).

To produce one comprehensive estimate of the probability of SAE, we performed a meta-analysis on proportions within single groups.

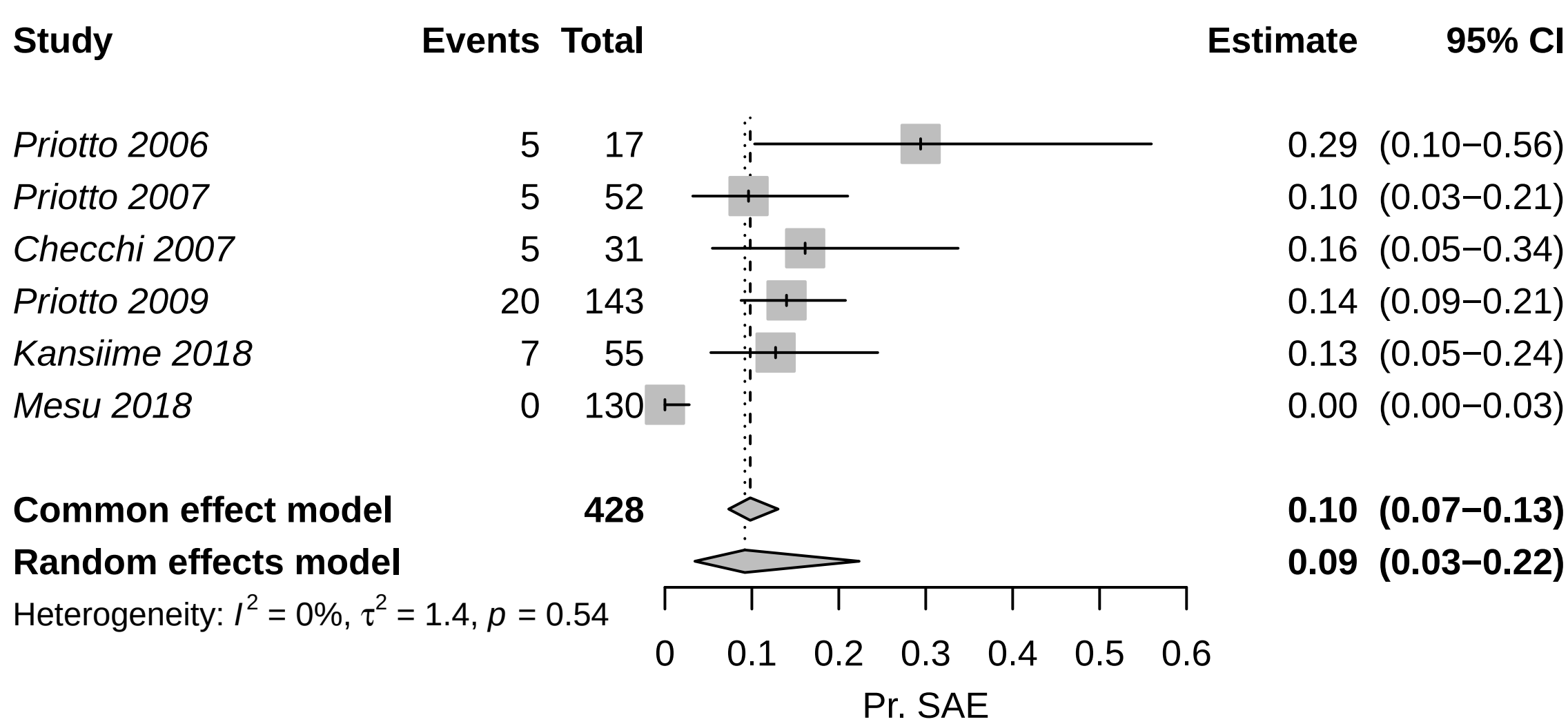

Reproduced from Antillon *et al* [1] with permission under a CC-BY license.

Because the data showed non-significant heterogeneity according to the tau-squared test for heterogeneity, we have chosen to use the fixed-effects estimate: 0.098 (0.073, 0.130), which would result from a beta distribution of Beta(40.88, 367.80).

S4.6.12 SAE: fexinidazole treatment

↩ Return to the Summary of Health Outcome Parameters.

- Name in the code: `treat_prob_sae_fexi`
- Source: [27]
- Country of estimate: DRC
- Statistical distribution and parameters: Beta(3, 261)
- Summary statistics (mean and 95% CI or fixed value): 0.01 (<0.01, 0.03)

Notes

There is only one published study on fexinidazole, so the probability of serious adverse events will be parameterized with the observations from that study: 4 adverse events attributable to fexinidazole in 3 people among 264 people, so we have assigned a distribution of Beta(3, 261).

S4.6.13 Days lost to disability: due to SAE

↩ Return to the Summary of Health Outcome Parameters.

- Name in the code: `treat_duration_sae`
- Source: [32]
- Country of estimate: DRC
- Statistical distribution and parameters: Gamma(1.22, 2.38)
- Summary statistics (mean and 95% CI or fixed value): 2.96 (0.14, 9.99)

Notes

Our only source of information for the duration of severe adverse events (SAEs) is Alirol 2013 [32], which lists the most common adverse events and the median duration of these events. Most events last a median of 1-2 days (with interquartile ranges reaching up to 4 days).

For simplicity, we have fit a gamma distribution with interquartile range of 1-4 days. Our distribution is therefore Gamma(1.22, 2.38) with a mean and 95% confidence interval of 2.92 (0.12, 9.95), which provides a sufficiently large range of values in light of the scarce information we have.

S4.6.14 Life-years lost (DALY) parameters

↩ Return to the Summary of Health Outcome Parameters.

S4.6.15 Age of death from infection

↩ Return to the Summary of Health Outcome Parameters.

- Name in the code: `age_of_death`
- Source: [17–27, 29, 32, 33]
- Country of estimate: DRC and South Sudan
- Statistical distribution and parameters: Gamma(148, 0.18)
- Summary statistics (mean and 95% CI or fixed value): 26.63 (22.41, 31.08)

Notes

No good registry of the age of infection exists, so we have searched through the literature that we have used to parameterize the model for the average age of HAT patients. Among the studies that we have used to inform other parameters, eight studies reported age information in a sample of patients of all ages, and nine studies reported the age information in a sample of older children or adults (12-15 years and older).

However, the data exists in a state that is difficult to synthesize:

Therefore, we have fit a gamma distribution to the means and medians of the studies that included patients of all ages. We have omitted the median from Alirol et al. 2013 [32] as this median seems unusually high – even higher than the mean age of patients in studies where only adults (over the age of 15) were recruited. Our distribution is therefore Gamma(147.93, 0.18) with a mean and 95% confidence interval of 27 (23, 31), which provides a sufficiently large bound of uncertainty in lieu of the information we have.

S4.6.16 Life expectancy

- Name in the code: `life_expectancy`
- Source: [34]
- Country of estimate: DRC
- Statistical distribution and parameters: Interpolation
- Summary statistics (mean and 95% CI or fixed value): See below.

Notes

We took age-specific life expectancy at around the time when people die of HAT in DRC for 2019, the last year for which there are estimates.

We see here that the expected years of life left at each of the ages is:

Using these data, we made an interpolating function in R (function: `approxfun`) that would calculate the life years left for each age of death (see previous parameter, `age_of_death`).

Citation	Group	Age Group	Summary
Priotto 2006	Melarsoprol and Nifurtimox	All ages	Mean: 29.1 range: 5-56
Priotto 2006	Melarsoprol and Eflornithine	All ages	Mean: 28.1 range: 11-61
Priotto 2006	NECT	All ages	Mean: 29.1 range: 9-62
Balasegaram 2006	Pentamidine	All ages	148 under 15 and 504 over 15
Eperon 2006	Pentamidine	All ages	56 patients 0-5, 226 patients 6-15, 568 patients 15+
Eperon 2006	Melarsoprol	All ages	63 patients 0-5, 249 patients 6-15, 796 patients 15+
Priotto 2007	NECT	Over 15 years old	Mean: 33.1 range: 15-69
Priotto 2007	Eflornithine	Over 15 years old	Mean: 36.1 range: 15-70
Checchi 2007	NECT	All ages	Mean: 23.9 range: 4-45
Priotto 2009	NECT	Over 15 years old	Mean: 32.8 SD: 12.5
Priotto 2009	Eflornithine	Over 15 years old	Mean: 34.6 SD: 13.5
Ngoyi 2010	Pentamidine	Over 12 years old	Mean: 35 SD: 13
Ngoyi 2010	Pentamidine and Melarsoprol	Over 12 years old	Mean: 34 SD: 12
Priotto 2012	Single arm	All ages	Median: 24 IQR: 15-35
Schmid 2012	Single arm	All ages	35 patients 0-4 yo, 65 patients 5-11 yo, and 529 patients 12 yo or more.
Hasker 2012	All	All ages	Median: 27 IQR: 16-40
Alirol 2013	Single arm	All ages	Median: 36 IQR: 20-50
Burri 2016	Pentamidine	Over 15 years old and >35 kg	Median: 31 range: 15-50
Pohlig 2016	Pentamidine	Over 12 years old and >30 kg	Median: 31 range: 13-75
Pohlig 2016	Pafuramidine	Over 12 years old and >30 kg	Median: 30 range: 12-64
Kansiime 2018	NECT	Over 15 years old	Mean: 27.23 SD: 12.07
Kansiime 2018	Eflornithine	Over 15 years old	Mean: 27.33 SD: 8.59
Mesu 2018	NECT	Over 15 years old	Mean: 35.2 SD: 13.2
Mesu 2018	Fexinidazole	Over 15 years old	Mean: 34.5 SD: 12.6

Age	Years left
15-19	52.4
20-24	48.1
25-29	44.0
30-34	39.9
35-39	35.7
40-44	31.6
45-49	27.6
50-54	23.6

Life-years left for each age group. Source: https://www.who.int/data/gho/data/indicators/indicator-details/GHO/gho-ghe-life-tables-by-country. Variable: *expectation of life at age x*.

S4.6.17 Disability weights: S1 disease

- Name in the code: `disability_weighting_s1`
- Source: [35]
- Country of estimate: GBD
- Statistical distribution and parameters: Beta(22.96, 147.21)
- Summary statistics (mean and 95% CI or fixed value): 0.14 (0.09, 0.19)

Notes

The Global Burden of Disease listed the impact of sleeping sickness as equivalent to the health state labelled "Motor plus cognitive impairments, severe" and their estimate for a disability weight is 0.542 (0.374-0.702), using the 2013 weight values. No distinction was made between stage 1 and 2 of the disease.

While this seems appropriate for the second stage of sleeping sickness, for stage 1 disability we chose to use the disability weights for equivalent to "infectious disease, acute episode, severe", which is described as "has a high fever and pain, and feels very weak, which causes great difficulty with daily activities" and has a much lower disability weight equivalent to 0.133 (0.088-0.190). The distribution for this parameter is therefore Beta(22.96, 147.21).

It should be noted that other cost-effectiveness analyses have used different values for disability weights [12, 36, 37]. These values arise from the 1994 Global Burden of Disease Study but we prefer to consider updated values. Since most of the disability is due to deaths rather than illness during life, we do not believe that this difference is cause for concern.

S4.6.18 Disability weights: S2 disease

- Name in the code: `disability_weighting_s2`
- Source: [35]
- Country of estimate: GBD
- Statistical distribution and parameters: Beta(18.37, 15.63)
- Summary statistics (mean and 95% CI or fixed value): 0.54 (0.37, 0.70)

Notes

The Global Burden of Disease listed the impact of sleeping sickness as equivalent to the health state labeled "Motor plus cognitive impairments, severe" and their estimate for a disability weight is 0.542 (0.374-0.702), using the 2013 weight values. No distinction was made between stage 1 and 2 of the disease. The distribution for the parameter is Beta(18.37, 15.63).

It should be noted that other cost-effectiveness analyses have used different values for disability weights [12, 36, 37]. These values arise from the 1994 Global Burden of Disease Study but we prefer to consider updated values. Since most of the disability is due to deaths rather than illness during life, we do not believe that this difference is cause for concern.

S4.6.19 Disability weights: SAE

↩ Return to the Summary of Health Outcome Parameters.

- Name in the code: `disability_weighting_sae`
- Source: [35]
- Country of estimate: GBD
- Statistical distribution and parameters: Uniform(0.04, 0.11)
- Summary statistics (mean and 95% CI or fixed value): 0.08 (0.04, 0.11)

Notes

As far as we are aware, no one has considered the disability due to severe adverse events attributable to gHAT treatment, but the most common adverse events are gastrointestinal problems and headaches.

We consulted the Global Burden of Disease for disability weights. The health state labeled "symptomatic tension-type headache" was described as "moderate headache that also affects the neck, which causes difficulty in daily activities" and was estimated to have a disability weight equal to 0.037 (0.022–0.057). The health state labeled "moderate symptomatic gastritis and duodenitis without anaemia" was described as "abdominopelvic problem, moderate has pain in the belly and feels nauseous; the person has difficulties with daily activities" and was estimated to have a disability weight equal to 0.114 (0.078–0.159).

Our distribution is therefore Uniform 0.037-0.114. Since most of the disability is due to death rather than illness during life, we do not believe that the uncertainty in this parameter is cause for concern for the purpose of the conclusions of this analysis.

S4.7 Vector control parameters

↩ Return to the Summary of Health Outcome Parameters.

S4.7.1 Linear km of targets

- Name in the code: `vc_length_default` and `vc_length_enhanced`
- Source: [38]
- Country of estimate: DRC
- Statistical distribution and parameters: Fixed value
- Summary statistics (mean and 95% CI or fixed value): Varies by health zone

Notes

The parameter for the extent of the riverbank covered by vector targets is a fixed value. The values for each health zone are detailed in the Supplementary Methods, section S1.7.1, and see Supplementary Note 2 for the length of rivers covered by VC per health zone.

S4.7.2 Target per km

↩ Return to the Summary of Health Outcome Parameters.

- Name in the code: `vc_density_linear`
- Source: [38]
- Country of estimate: DRC
- Statistical distribution and parameters: Fixed value, varies by health zone
- Summary statistics (mean and 95% CI or fixed value): Varies by health zone

Notes

In this analysis, we are using bank length, because, in some health zones, we do not cover with targets on both sides. In DRC, on average, while the goal is to cover rivers with 40 targets per kilometre, placing them 25m apart on alternating sides, or 50m apart on each side, records have shown that approximately 30 targets are used per kilometre. We parameterize the targets per kilometre as half that since we are considering the bank length, rather than the river length.

We used a fixed value for the parameter for the extent of the riverbank covered by vector targets.

S4.7.3 Replacement rate of targets per year

↩ Return to the Summary of Health Outcome Parameters.

- Name in the code: `vc_deployments_yr`
- Source: [38, 39]
- Country of estimate: DRC
- Statistical distribution and parameters: Fixed value
- Summary statistics (mean and 95% CI or fixed value): 2

Notes

We have set this parameter as a fixed number, as this is the number of times that one must replace a set of targets in order to provide continuous protection throughout the year [38, 39].

S4.8 Screening cost parameters

S4.8.1 AS: capital costs of a team

↩ Return to the Summary of Cost Parameters.

- Name in the code: `as_cost_team_capital`
- Source: [11–13]
- Country of estimate: DRC
- Distribution and parameters: Gamma(820, 20.1)
- Summary statistics (mean and 95% CI or fixed value): 16,514 (15,410, 17,671)

Notes

Capital costs are denominated in 2024 US dollars.

To our knowledge, no active surveillance costs have been estimated via a detailed costing study [40]. We looked at the three studies that calculated costs using an ingredients approach, but decided on using the estimates from the more recent studies only [11–13].

Lutumba and colleagues [11] calculated that the total cost of screening 40,000 patients in 2003 was 46,734.29 Euros (1 Euro = 0.86 USD in 2003) (see table 3 of [11]). Of that value, 21 percent of the costs were capital costs, or a cost of 32,913 in 2024 USD. The publication did not indicate whether that value was annualized or not.

According to Bessel and colleagues [12] the cost for a mobile team that screens 250 patients per day for 220 days a year has capital investments (annualized for five years) of $12,000 for a team that administers CATT and $12,781 for a team that administers RDT (in 2013 USD) (see [12] Table S1). Adjusting for inflation, that would equal 17,671 in 2024 USD.

According to Snijders et al [13], the capital cost of an active surveillance team was $11,406 in 2018 for a team that administers CATT. Adjusting for inflation, that would equal 15,410 in 2024 USD.

Although no publication gave us a sense of the uncertainty in capital costs, we had three studies, but we felt that the Lutumba publication was too old, the costs were too high, and the question of annualization could influence the comparability of that cost. Nevertheless, we assumed that the range was equivalent to the 95% confidence interval. Therefore, our probability distribution is Gamma(820, 20.1), which yields a distribution with mean and confidence intervals of 16,514 (15,410, 17,671).

S4.8.2 AS: recurrent & management costs of a team

↩ Return to the Summary of Cost Parameters.

- Name in the code: `as_cost_team_management`
- Source: [11–13]
- Country of estimate: DRC
- Distribution and parameters: Gamma(4153.88, 17.21)
- Summary statistics (mean and 95% CI or fixed value): 71,472 (69,311, 73,658)

Notes

Recurrent management costs are denominated in 2022 US dollars.

To our knowledge, no active surveillance costs have been estimated via a detailed costing study [40]. We looked at the three studies that calculated costs using an ingredients approach, but decided on using the estimates from the more recent studies only [11–13].

Lutumba and colleagues [11] calculated that the total cost of screening 40,000 patients in 2003 was 46,734.29 Euros (1 Euro = 0.86 USD in 2003, for an equivalent of $40,191) (see table 3 of [11]). Of that value, 79 percent of the costs were recurrent fixed costs ($31,751), or a cost of 123,817 in 2024 USD after adjusting for inflation and changes in the exchange rate.

According to Bessel and colleagues [12] the cost for a mobile team that screens 250 patients per day for 220 days a year has annual recurrent costs of $30,307 and daily recurrent costs of $97 in 2013 values (see [12], Table S1). Summing those costs ($51,647) and adjusting for inflation and changes in the exchange rate, would equal 73,658 in 2024 USD. Although Bessel and colleagues considered the cost of RDT use in AS, they assumed that the teams that would be using RDTs were traditional mobile units (with sport-utility vehicles) rather than the mini-mobile units with motorcycles that constitute the majority of RDT use in AS nowadays.

According to Snijders and colleagues [16], the management and recurrent costs of an active surveillance team were $42,408 in 2018, and $7,961 for the management costs from the provincial and central level PNLTHA (including training and supervision) for a total of $51031. Adjusting for inflation and changes in the exchange rate, would equal 69,311 in 2024 USD. It should be noted that these were the costs for a traditional team using sport utility vehicles and CATT tests, rather than a 'mini-team' using motorcycles and RDTs.

Although no publication gave us a sense of the uncertainty in capital costs, we had three studies, but we felt that the Lutumba publication was too old. If we assumed that the range of the other two observations was equal to the 95% confidence interval (69,311, 73,658), our probability distribution is Gamma(4153.88, 17.21), which yields a distribution with mean and confidence intervals of 71,472 (69,311, 73,658).

S4.8.3 CATT algorithm: cost per test used

↩ Return to the Summary of Cost Parameters.

- Name in the code: `dx_cost_catt`
- Source: [41]
- Country of estimate: international market (Belgium)
- Distribution and parameters: Gamma(1140, 0.0006897)
- Summary statistics (mean and 95% CI or fixed value): $0.79 (0.74, 0.83)

Notes

The CATT test is sold in the international market by Institute of Tropical Medicine in Antwerp.

A kit of reagents is 280.18€ for 500 tests and accessories are 35.82€ for 250 tests, which yields a total of 0.70€ per person [41]. The capital necessary to carry out the test (the rotator field kit) is considered under the parameter for capital costs.

The price of the CATT varies due to uncertainty in the exchange with the USD. Judging by variation in the five years previous to and including 2024 (0.85-0.95), the range of the cost of the test can be between US$0.74-0.83, which is the 95% confidence interval of a distribution given by Gamma(1140, 0.0006897). The final distribution is given by $0.79 (0.74, 0.83) in USD.

Although delivery costs (described in S4.9.6) are considered to be about 45% of the cost, after conversations with ITM colleagues, we decided to assume that the delivery cost for this is equal to a 10% markup per year. The markup is higher than the RDT tests because the CATT test shipping may need a cold-chain, whereas the RDT shipping would not (CATT reagents must be stored in temperatures of 2-8C; see S4.8.6). The 10% estimate was also the assumption taken in a previous costing paper by Snijders and colleagues [13].

S4.8.4 Staging: lumbar puncture & lab exam

↩ Return to the Summary of Cost Parameters.

- Name in the code: `dx_cost_lumbar_exam`

- Source: [13, 42]
- Country of estimate: DRC and Chad
- Distribution and parameters: Gamma(3.73, 2.96)
- Summary statistics (mean and 95% CI or fixed value): 13.76 (3.37, 31.08)

Notes

To our knowledge, costs for lumbar puncture tests were listed in detail only by Bessel and colleagues [12] and will be featured as part of an upcoming publication by Snijders and colleagues [13]. The cost listed by Bessel et al was 2.38 in terms of 2013 USD, equivalent to 3.39 in 2024 USD. Snijders and colleagues report a cost of 23.02 in 2018 USD, or 31.10 in 2024 USD. Irurzun-Lopez [42] have reported a similar value, so we will assume a value equal to that of Snijders and colleagues.

Because we had more than two studies to inform this parameter, we chose a gamma distribution where the 2.5th and the 97.5th percentiles would match the minimum and maximum values in the literature, so our parameter distribution is Gamma(3.59, 3.82), which yields a distribution with a mean and confidence interval of 13.76 (3.37, 31.08).

S4.8.5 Confirmation: microscopy

↩ Return to the Summary of Cost Parameters.

- Name in the code: `dx_cost_microscopy`
- Source: [16]
- Country of estimate: DRC
- Distribution and parameters: Gamma(8.47, 1.71)
- Summary statistics (mean and 95% CI or fixed value): 13.92 (6.44, 25.75)

Notes

The per-patient price to confirm a patient with a full microscopy procedure is reported in an upcoming publication by Snijders et al [16]. They report that a microscopy procedure consisting of mAECT and LNA (lymph node aspiration) costs 9.53 in 2018 USD, or 12.88 in 2024 USD. Because we had no report of the standard error around that estimate, we assigned a gamma distribution that had a confidence interval that spanned half the estimate and twice the estimate, yielding a distribution of Gamma(8.47, 1.71), and a mean and confidence interval of 13.92 (6.44, 25.75).

S4.8.6 RDT: costs per test used

↩ Return to the Summary of Cost Parameters.

- Name in the code: `dx_cost_rdt`
- Source: Co-authors from ITM
- Country of estimate: international market (Belgium)
- Distribution and parameters: Gamma(1140, 0.002205)
- Summary statistics (mean and 95% CI or fixed value): $2.51 (2.37, 2.66)

Notes

If one takes the Snijders et al estimate from the micro-costing analysis the cost is between 0.85 and 1.97 in 2018 USD, from Abbott and Coris, respectively. The Abbott RDT is no longer available, and therefore, one can only purchage tests from Coris. Those tests costs would be equivalent to 1.50€. However, as of 2025, Coris Sero-K-Set RDTs 2.25€ per test.

The price of the RDT varies due to uncertainty in the exchange with the USD. Judging by variation in the five years previous to and including 2024 (0.85-0.95), the range of the cost of the test can be between US$1.904-2.128, which is the 95% confidence interval of a distribution given by Gamma(1139.58093, 0.002205). The final distribution is given by $2.51 (2.37, 2.66).

Although delivery costs (described in S4.9.6) are considered to be about 45% of the cost, after conversations with ITM colleagues, we decided to assume that the delivery cost for this is equal to a 5% markup per year. The markup is lower than the CATT tests because the CATT test shipping may need a cold-chain, whereas the RDT shipping would not (CATT reagents must be stored in temperatures of 2-8C; see S4.8.3).

S4.8.7 Indirect management costs (PNLTHA mark-up)

↩ Return to the Summary of Cost Parameters.

- Name in the code: `program_markup_vc`
- Source: [16]
- Country of estimate: DRC
- Distribution and parameters: Uniform(0.1, 0.2)
- Summary statistics (mean and 95% CI or fixed value): 0.15 (0.10, 0.20)

Notes

Snijder's and colleagues [16] have assumed that there is a component of management at the national programme (PNLTHA) level that is approximately 15% of the expenses at the local and coordination level (both fixed costs and variable/consumable costs for both active screening and passive screening in fixed health posts). However, the mark-up was not applied to the consult in the fixed health post, as these were consultations paid for by patients for symptoms in general, but testing and confirmation for HAT specifically is administered and paid for by PNLTHA, so we have included a mark-up for these items.

In this analysis, this was only included in this paper for VC costs and treatment, as the overhead for AS and PS activities was included in the general management of those activities.

S4.8.8 PS: capital costs of a facility

↩ Return to the Summary of Cost Parameters.

- Name in the code: `ps_cost_facility_capital`
- Source: [16]
- Country of estimate: DRC
- Distribution and parameters: Gamma(8.475, 283.46)
- Summary statistics (mean and 95% CI or fixed value): 2393 (1067, 4269)

Notes

Capital costs are denominated in 2024 USD and apply to each health centre or hospital that is capable of HAT diagnosis.

We took into account the results of a micro-costing study in Yasa Bonga and Mosango [16], two health zones of Kwilu Province (Bandundu Sud coordination). The study reported a cost of 1580 USD in 2018 values, for an equivalent of 2135 in 2024 USD.

To parameterize the model, we assign a distribution with 95% confidence intervals equal to half and double the costs. The distribution is Gamma(8.47, 283.46), which yields a distribution with mean and confidence intervals of 2393 (1067, 4269).

S4.8.9 PS: management costs

↩ Return to the Summary of Cost Parameters.

- Name in the code: `ps_cost_management`
- Source: [16]
- Country of estimate: DRC
- Distribution and parameters: Gamma(8.475, 145)
- Summary statistics (mean and 95% CI or fixed value): 1223 (560, 2157)

Notes

In 2025, the program costs for coordination management (which is the only management for PS) was $650,000 for the whole country for 179 health zones for support of both AS and PS activities. The micro-costing study in Yasa Bonga and Mosango [16] noted that about 30% of coordination effort is spent on support of fixed health facilities.

We do not adjust the cost between years because this has stayed roughly stable over the years, and the funding is all external (denominated in Euros or US Dollars, not in Congolese Francs).

$650K divided by 179 and taking only 30% of that would equal to $1090. Because we do not have a sense of how much this may change over the next few years, we still know there is some uncertainty in this estimate, so we apply a Gamma distribution where the 95% CI equals half and double our estimate: Gamma(8.475, 145) with mean estimate and PI of 1223 (560, 2157).

S4.9 Treatment cost parameters

S4.9.1 Hospital stay: cost per day

↩ Return to the Summary of Cost Parameters.

- Name in the code: `treat_cost_ip_day`
- Source: [3, 43, 44]
- Country of estimate: DRC
- Distribution and parameters: Gamma(5.81, 0.59)
- Summary statistics (mean and 95% CI or fixed value): 3.42 (1.22, 6.76)

Notes

We got the estimates of inpatient treatment costs from the 2010 WHO CHOICE cost estimates (recently updated by [43, 44]). In 2010, a consult at a primary hospital in DRC would be 2.41 (0.90, 5.73) I$. In 2010, a consult at a secondary hospital in DRC would be 2.59 (0.98, 5.81) I$, and a consult at a tertiary hospital in DRC would be 3.25 (1.32, 7.20) I$.

The equivalent estimates in 2010 USD are 1.40 (0.52, 3.33) per day at a primary hospital, 1.51 (0.57, 3.38) per day at a secondary hospital, and 1.89 (0.77, 4.19) per day at a tertiary hospital. After converting to local currency, applying the inflation index, and converting to 2024 USD, the estimates are 2.25 (0.84, 5.36) per day at a primary hospital, 2.42 (0.92, 5.43) per day at a secondary hospital, and 3.04 (1.23, 6.73) per day at a tertiary hospital.

At the moment, we do not know how many of each kind of hospital the population of HAT patients attend, nor do we understand how costs at district hospitals, referral hospitals, etc resemble those of the two kinds of hospitals under analysis by the WHO CHOICE program. Therefore, we take the estimate with a higher mean (hospital day in a tertiary hospital) in an effort not to under-state the costs of treatment and interventions.

To parameterize the model, we assign a gamma distribution with 95% confidence intervals equal to those reported by WHO CHOICE [3]. The distribution is Gamma(5.81, 0.59), which yields a distribution with median and confidence intervals of 3.42 (1.22, 6.76). Although this yields a higher mean, the uncertainty is adequately characterized.

S4.9.2 Outpatient consultation: cost

↩ Return to the Summary of Cost Parameters.

- Name in the code: `treat_cost_op_visit`
- Source: [16, 45]
- Country of estimate: DRC
- Distribution and parameters: Gamma(24.2, 0.13)
- Summary statistics (mean and 95% CI or fixed value): 3.13 (2.02, 4.47)

Notes

We got the estimates of outpatient consultation costs from two sources:

1) Laokri and colleagues [45] presented an estimate with mean $2.33 and standard deviation 0.27 in 2013 values, or 2.01 (0.23) in 2024 USD values.
2) Snijders and colleagues [16] reported that the cost of a consultation is $3.33 in 2018 USD values, or $4.50 in 2024 values.

We will use the two mean estimates as the range of a gamma distribution, so we get Gamma(24.2, 0.13) and a sample with mean and 95% CI of 3.13 (2.02, 4.47)

S4.9.3 Course of pentamidine: cost

↩ Return to the Summary of Cost Parameters.

- Name in the code: `rx_cost_pentamidine`
- Source: [40]
- Country of estimate: WHO
- Distribution and parameters: Fixed value
- Summary statistics (mean and 95% CI or fixed value): 54

Notes

The cost of pentamidine, for stage 1 disease. Because it is available on the international market, where is it sold in USD, and not subject to the inflationary pressures of any particular country, we have not inflated the cost or converted them to any other currency.

In the future pentamidine treatment it may be replaced with fexinidozole treatment, which would circumvent the need for a lumbar puncture.

S4.9.4 Course of NECT: cost

↩ Return to the Summary of Cost Parameters.

- Name in the code: `rx_cost_nect`
- Source: [46]
- Country of estimate: WHO
- Distribution and parameters: Fixed value
- Summary statistics (mean and 95% CI or fixed value): 360

Notes

This represents the cost of NECT to the capital for stage 2 disease. Simarro and colleagues listed a cost of 1440 USD for the treatment of four patients.

Because it is available on the international market, where is it sold in USD, and not subject to the inflationary pressures of any particular country, we have not inflated the cost or converted them to any other currency.

In the future, it may be replaced with fexinidozole and this would be the drug for treatment failures or very severe patients.

S4.9.5 Course of fexinidazole: cost

↩ Return to the Summary of Cost Parameters.

- Name in the code: `rx_cost_fexinidazole`
- Source: [37]
- Country of estimate: WHO
- Distribution and parameters: Fixed value
- Summary statistics (mean and 95% CI or fixed value): 50

Notes

The cost of fexanidozole, for stage 1 and 2 disease. In the near future this will be the drug of choice for first-line treatment for both stages of disease. It may require hospitalization, but eventually, it should be taken on an outpatient basis.

S4.9.6 Drug delivery mark-up

↩ Return to the Summary of Cost Parameters.

- Name in the code: `rx_delivery_markup`
- Source: [43, 44]

- Country of estimate: DRC
- Distribution and parameters: Beta(45, 55)
- Summary statistics (mean and 95% CI or fixed value): 0.45 (0.35, 0.55)

Notes

Because we do not know the delivery price of drugs for each country, we have applied the standard value for the mark up of traded goods recommended by the WHO CHOICE programme for AFRO E: https://www.who.int/teams/health-systems-governance-and-financing/economic-analysis/costing-and-technical-efficiency/quantities-and-unit-prices-(cost-inputs).

S4.10 Vector control cost parameters

S4.10.1 Operational cost per kilometer of riverbank covered

↩ Return to the Summary of Cost Parameters.

- Name in the code: `vc_cost_management`
- Source: [47]
- Country of estimate: DRC
- Distribution and parameters: Gamma(8.47, 57.7)
- Summary statistics (mean and 95% CI or fixed value): 491 (217, 869).

Notes

Vector control operational costs are denominated in 2024 US dollars on a per-kilometer basis.

To our knowledge, only one vector control micro-costing study has been performed in DRC by Snijders and colleagues [47]. In that study, centred in Yasa Bonga, Kwilu Province, targets were laid out across 210 km of river length (or 420km of river bank). The operational costs came to 61,796 USD, or 294.27 USD (in 2016 values) per km. The equivalent cost is 435 in 2024 USD per kilometer.

As there was no sense of the uncertainty in VC operational costs, we assigned a distribution with confidence intervals equal to half and double the costs. The distribution is Gamma(8.47, 57.7), which yields a distribution with mean and confidence intervals of 491 (217, 869).

S4.10.2 Deployment cost per target

↩ Return to the Summary of Cost Parameters.

- Name in the code: `vc_cost_deployment`
- Source: [47]
- Country of estimate: DRC
- Distribution and parameters: Gamma(8.47, 0.50)
- Summary statistics (mean and 95% CI or fixed value): 4.21 (1.88, 7.50)

Notes

Target deployment costs are denominated in 2024 US dollars on a per-target basis.

To our knowledge, only one vector control micro-costing study has been performed in DRC by Snijders and colleagues [47]. In that study, centred in Yasa Bonga, Kwilu Province, 22,622 targets were laid out across 210 km of river length (or 420 of river bank). The target deployment activities cost 57,571 USD, or per target 2.54 USD (in 2016 values). The equivalent cost is 3.75 in 2024 USD per target.

Uncertainty: as there was no sense of the uncertainty in target deployment costs, we assigned a distribution with confidence intervals equal to half and double the costs. The distribution is Gamma(8.47, 0.50), which yields a distribution with mean and confidence intervals of 4.21 (1.88, 7.50).
