# Supplementary material for: Cost-effectiveness of end-game strategies against sleeping sickness across the Democratic Republic of Congo
Source: PLoS Negl Trop Dis. 2026 Oct 22;20(10):e0014777. doi: 10.1371/journal.pntd.0014777 (PMC13719019; doi:10.1371/journal.pntd.0014777)
Supplement: Supplementary file 5 [file pntd.0014777.s005.pdf]

Supplementary Materials
Cost-effectiveness of end-game strategies against sleeping sickness across the Democratic Republic of Congo

Supplementary Results

S5.1 Modeling results for sample health zone: Kikongo . . . 2
S5.2 Model selection . . . 2
S5.3 Detected cases and undetected deaths . . . 6
S5.4 Cost-effectiveness for example health zones: Kikongo and Kwamouth . . . 7
S5.5 Cost-effectiveness in Bas Uélé region: Ango and Doruma health zones . . . 10
S5.6 Results by coordination . . . 13
S5.7 Resource forecasts . . . 23

S5.1 Modeling results for sample health zone: Kikongo

↩ Return to the Supplement Table of Contents

Here we briefly show the results for one health zone to give a sense of the outputs for the fitting step for each health zone. All outcomes, AS and PS cases as well as new infections decrease over time thanks for screening. Active and Passive detections are usually a subset of 'new infections' from previous years. There is no 'validation' of New Infections against the data because new infections are an unobserved process. The new infections that are never detected are assumed to result in death, which is further discussed in Section S5.3. Similar results are available for all health zones in the graphical user interphase https://hatmepp.warwick.ac.uk/DRCCEA/v8/.

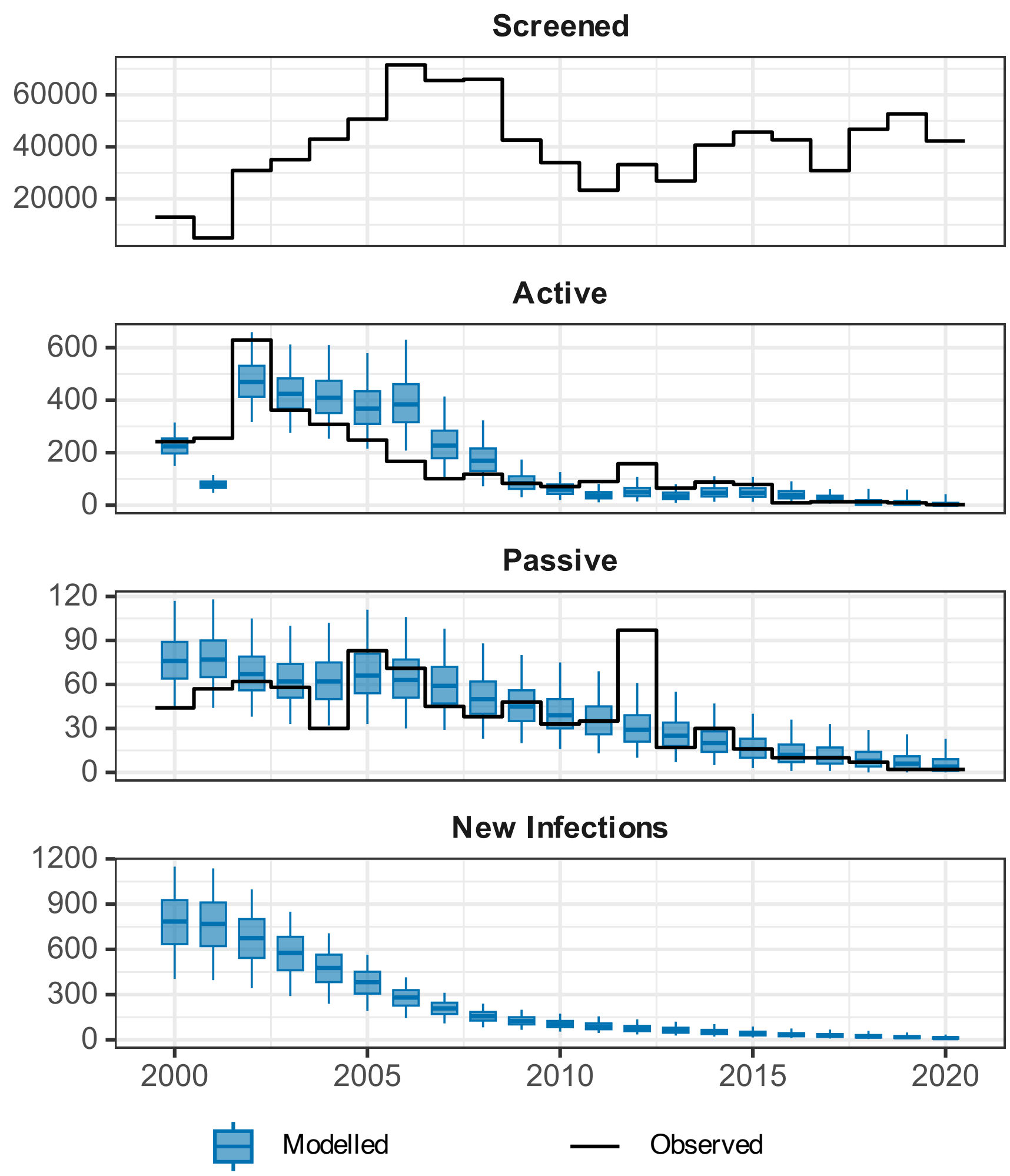

Fig A: Fit to historical case data (2000–2020) from active and passive screening in the Kikongo health zone of the DRC. Outputs include estimating unobservable new infections per year (bottom row). Blue box-and-whiskers present within-year summaries of model fits (median for centre line; and 50% and 95% credible intervals for the box and whiskers, respectively).

S5.2 Model selection

↩ Return to the Supplement Table of Contents

We take this opportunity to update some of the results presented in our previous paper on the fitting of the model including animal transmission [1]. We do this in light of the availability of a considerable amount of new data (2000–2020 rather than the previous 2000–2016) and modifications made to the modelling process.

To obtain the statistical support for the two model variants in each health zone following fitting we categorised the Bayes factors (BF), where BF may be used to describe the statistical support for either model relative to the other, in a widely accepted way [2]:

Support for Model	Bayes Factor
Weak ('Barely worth mentioning')	$10^0 < \mathrm{BF} < 10^{\frac{1}{2}}$
Substantial	$10^{\frac{1}{2}} < \mathrm{BF} < 10^1$
Strong	$10^1 < \mathrm{BF} < 10^{\frac{3}{2}}$
Very Strong	$10^{\frac{3}{2}} < \mathrm{BF} < 10^2$
Decisive	$\mathrm{BF} > 10^2$

If BF for the model with animal transmission was greater than 1, the categorisation indicating statistical support for the model with animal transmission was used, otherwise, the categorisation used was for statistical support for the model without animal transmission [1]. Fig B shows the results from the current analyses and can be compared to Figure 3 of the previous article [1]. There was less support for the model with animal transmission than there was in our previous analyses [1]. In the previous article, 24 health zones were found to have 'Substantial' to 'Decisive' support for the model with animal transmission, whereas as a result of additional data and modifications to the modelling, there is now only a single health zone in this category.

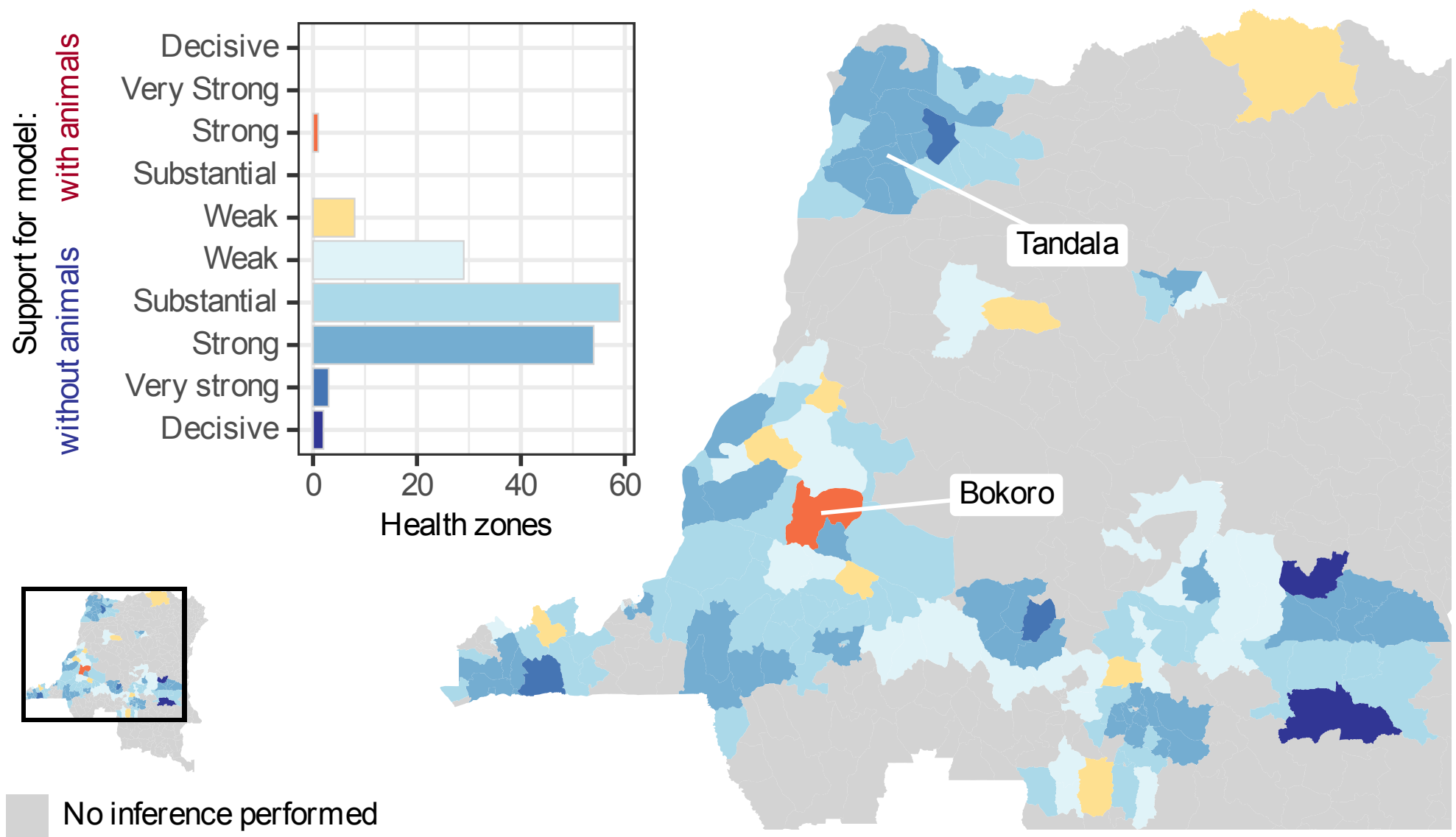

Fig B: Support for the model either with or without animals contributing to the transmission of gHAT. Levels of support taken from whichever of the two Bayes Factors (BF, with either evidence for the model with or without animal transmission as the denominator) exceeded 1. Weak, $10^0 < \mathrm{BF} < 10^{\frac{1}{2}}$; Substantial, $10^{\frac{1}{2}} < \mathrm{BF} < 10^1$; Strong, $10^1 < \mathrm{BF} < 10^{\frac{3}{2}}$; Very Strong, $10^{\frac{3}{2}} < \mathrm{BF} < 10^2$; and Decisive, $\mathrm{BF} > 10^2$. Health zones used as examples in Crump et al (2022), Bokoro and Tandala, are indicated. Shapefiles used to produce this map were provided by Nicole Hoff and Cyrus Sinai under a CC-BY licence (current versions can be found at https://data.humdata.org/dataset/drc-health-data).

Fig C is a bivariate choropleth map which combines support for models with and without animal transmission with differences in the probability of achieving the end of transmission by 2030 under the strategy where active screening in the health zone continues at the mean level observed in the period 2012–2016 ($P_d = \mathbb{P}(\text{EOT by 2030}|\text{Model without animal transmission}) - \mathbb{P}(\text{EOT by 2030}|\text{Model with animal transmission})$. This is an

update of Figure B in the Supplementary Information of the previous animal transmission modelling paper [1]. For presentation in Fig C both variables were put into three categories:

Choice of Model	Bayes Factor Values
without animals	$\mathrm{BF_{wo}} > 10^{\frac{1}{2}}$
	or $\mathrm{BF_{wo}} < 10^{\frac{1}{2}} \wedge \mathrm{BF_{w}} < 10^{\frac{1}{2}}$
with animals	$\mathrm{BF_{w}} > 10^{\frac{1}{2}}$

for model support, where $\mathrm{BF_{wo}}$ is the Bayes Factor value for support for the model without animals, and $\mathrm{BF_{w}}$ is the Bayes Factor value for support for the model with animals. For the difference in probability of achieving EoT by 2030 (P_d):

Category	Δ Pr. EoT by 2030
low	$P_d \leq 0.05$
medium	$0.05 < P_d \leq 0.1$
high	$P_d > 0.1$

In Fig C there are 31 health zones (dark purple) with (i) more than 10% reduction in the probability of meeting the EoT goal under the model with animal transmission compared to the model without animal transmission and (ii) with weak support for either model variant. In the previous article, this category contained 18 health zones. This discrepancy likely reflects both the additional data included and a change to the priors associated with the animal transmission model to remove the nesting of the models. In these 31 health zones, there is considerable uncertainty in whether animals contribute to transmission and this could alter policy recommendations for future strategy based on model predictions. The single health zone with strong support for the model with animal transmission also has a $> 10\%$ reduction in the probability of reaching the 2030 EoT goal.

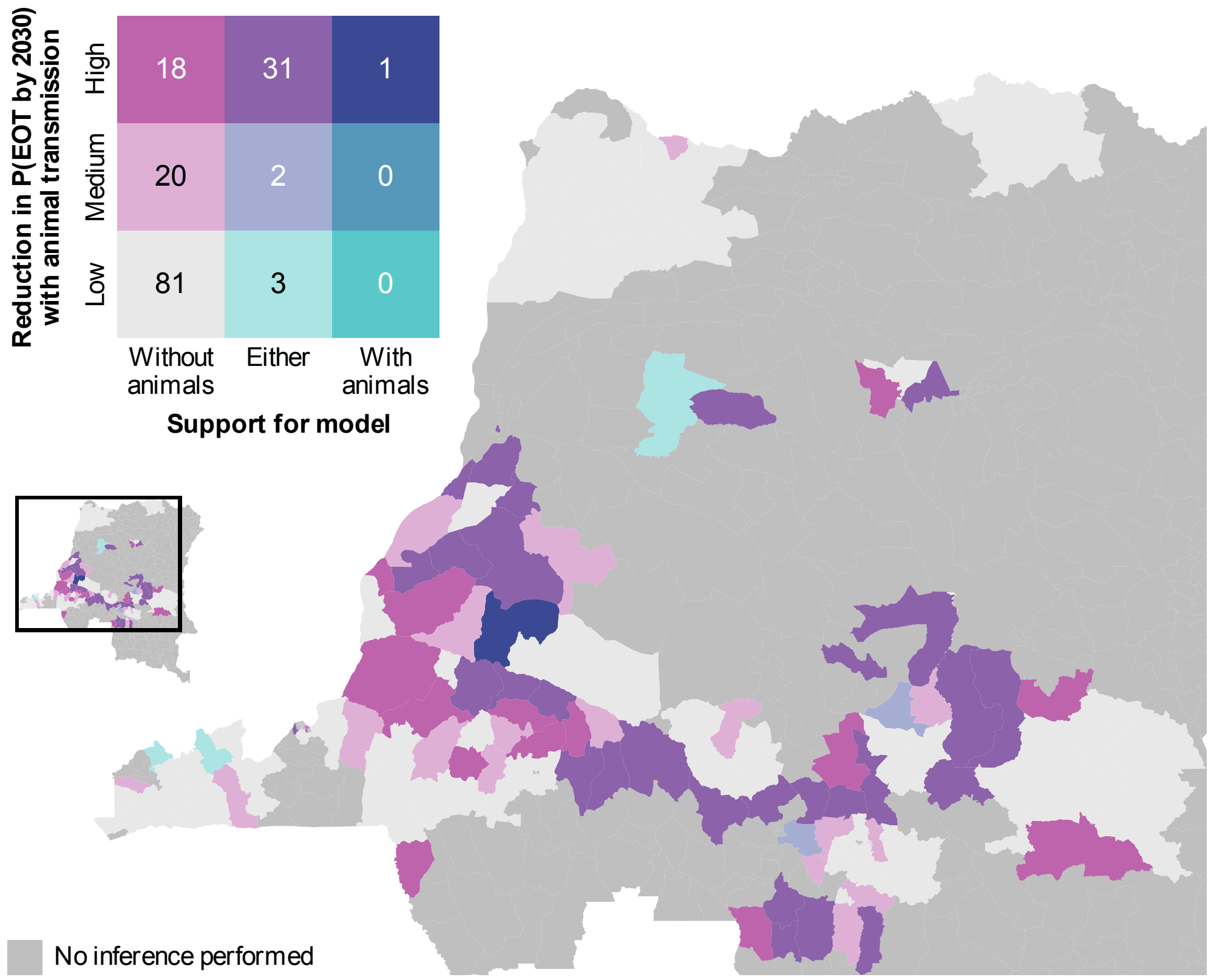

Fig C: Bivariate choropleth showing support for the models with or without animals contributing to transmission and the difference in the probability (P_d) of achieving EoT to humans by 2030 from these two models ("High" is more than 10% difference, "Medium" is 5-10% difference, and "Low" is less than 5% difference). Shapefiles used to produce this map were provided by Nicole Hoff and Cyrus Sinai under a CC-BY licence (current versions can be found at https://data.humdata.org/dataset/drc-health-data).

S5.3 Detected cases and undetected deaths

↩ Return to the Supplement Table of Contents

As there is no data available to inform this projection in the DRC, we have to infer the unreported deaths. In the model, the number of unreported cases (assumed to all result in deaths) are triangulated by the number of active cases screened, the presence or absence of VC, the cases detected and the proportion of cases detected that are S1 vs S2 cases. For a more extended discussion of the subject of detected and undetected cases in the model, see the S1 Text for Antillon et. al (2023) [3], pages 11-14. An illustration of the cases (reported, unreported, and false-positives) as well as the proportion of cases reported is illustrated in Fig D.

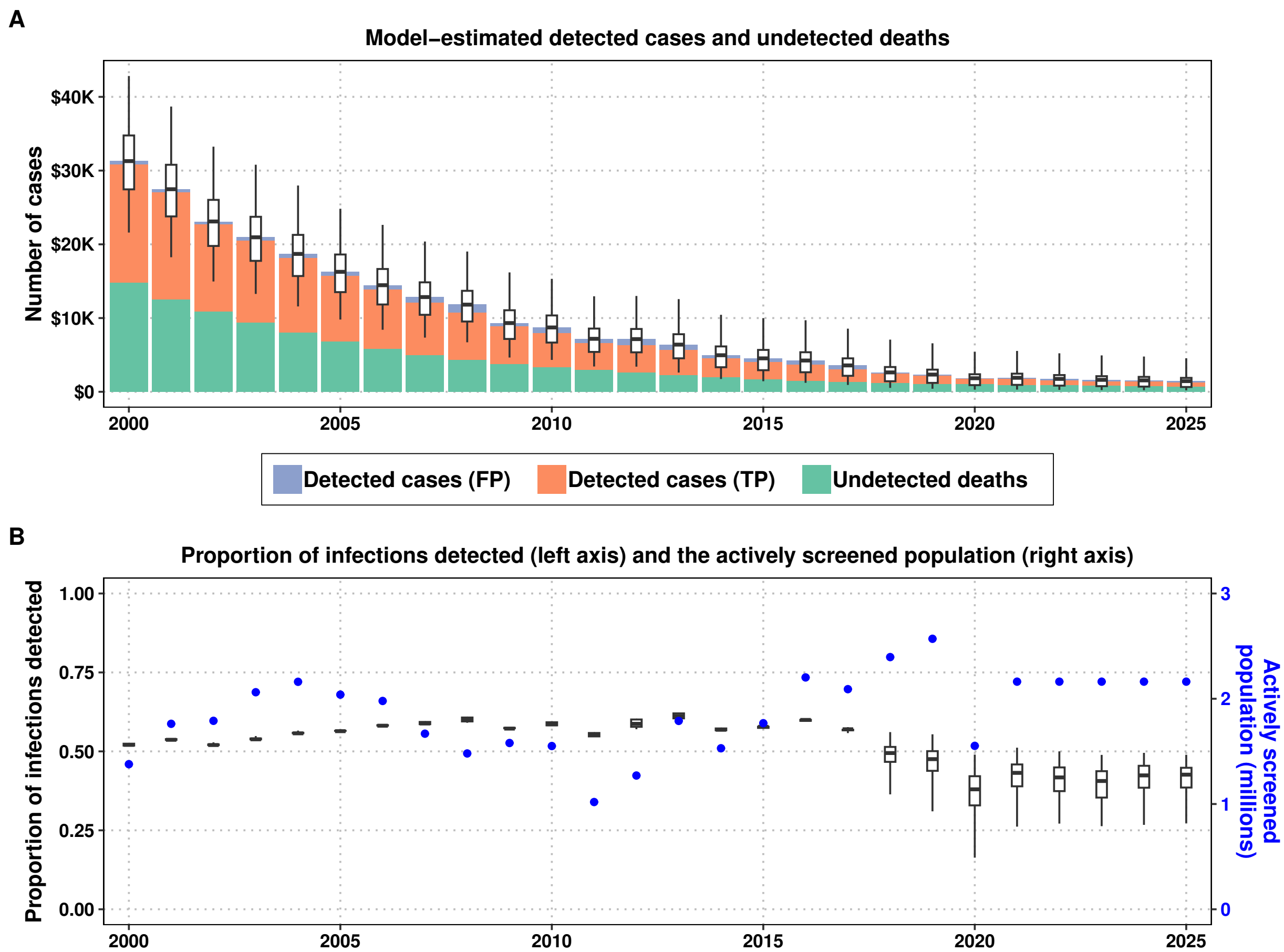

Fig D: Comparison of detected and undetected infections generated by the model and their relationship to active screening intensity. A) detected cases, undetected cases – which result in deaths – and false positive cases. Box-and-whisker plots show the mean estimate, interquartile range, and 95% prediction intervals of the total number of cases. B) the proportion of cases detected, and the population tested by mobile screening teams. Abbreviations: TP: true positives, FP: false positives.

S5.4 Cost-effectiveness for example health zones: Kikongo and Kwamouth

↩ Return to the Supplement Table of Contents

	Mean AS	Int. AS	Mean AS + Targeted VC	Int. AS + Targeted VC	Mean AS + Full VC	Int. AS + Full VC
**Health effects**						
Reported cases	23 (0, 103)	22 (0, 109)	14 (0, 61)	15 (0, 76)	12 (0, 53)	12 (0, 67)
Deaths undetected	25 (0, 111)	18 (0, 76)	15 (0, 59)	12 (0, 48)	12 (0, 47)	10 (0, 38)
Cases total	47 (0, 197)	40 (0, 168)	29 (0, 106)	27 (0, 107)	24 (0, 86)	22 (0, 91)
Deaths detected[a]	0 (0, 0)	0 (0, 0)	0 (0, 0)	0 (0, 0)	0 (0, 0)	0 (0, 0)
YLD	29 (0, 113)	21 (0, 84)	18 (0, 69)	15 (0, 56)	15 (0, 58)	12 (0, 48)
YLL	1,101 (0, 4,988)	792 (0, 3,424)	677 (0, 2,616)	534 (0, 2,112)	550 (0, 2,114)	448 (0, 1,698)
DALYs	1,129 (0, 5,086)	813 (0, 3,487)	696 (0, 2,669)	548 (0, 2,154)	565 (0, 2,155)	460 (0, 1,730)
ΔDALYs	0 (0, 0)	317 (-1,475, 3,113)	434 (-1,039, 3,443)	581 (-711, 3,754)	565 (-708, 3,752)	669 (-495, 3,987)
**Costs, in thousands US$ (not discounted)**						
AS costs	990 (204, 1926)	2436 (1801, 3332)	816 (203, 1629)	2343 (1795, 3147)	738 (201, 1479)	2303 (1785, 3058)
PS costs	681 (451, 958)	668 (444, 945)	658 (443, 934)	646 (438, 917)	643 (439, 908)	633 (433, 897)
VC costs	0 (0, 0)	0 (0, 0)	169 (26, 428)	158 (26, 389)	1182 (198, 2837)	1123 (199, 2659)
Treatment	12 (0, 55)	12 (0, 57)	8 (0, 33)	8 (0, 40)	6 (0, 29)	7 (0, 36)
Costs total	1683 (728, 2758)	3116 (2336, 4130)	1651 (756, 2735)	3155 (2386, 4160)	2569 (964, 4757)	4066 (2634, 6037)
ΔCosts	0 (0, 0)	1,433 (449, 2,476)	-33 (-1,111, 1,049)	1,471 (497, 2,486)	886 (-599, 2,815)	2,383 (1,032, 4,135)
**EoT**						
Year of EoT	2035 (2023, 2057)	2033 (2023, 2054)	2032 (2023, 2053)	2031 (2023, 2050)	2030 (2023, 2050)	2030 (2023, 2047)
Prob EoT 2030	0.37	0.44	0.53	0.61	0.67	0.72
Prob EoT 2040	0.80	0.87	0.91	0.93	0.94	0.95
**Cost-effectiveness without uncertainty (discounted)[b]**						
ΔDALYs	0	148	199	273	262	315
ΔCosts	0	1,129,905	8,734	1,189,285	883,821	2,054,185
ICER	Min Cost	Dominated	44	Weakly Dominated	13,839	22,072
**Cost-effectiveness with uncertainty (discounted), conditional on WTP[c].**						
WTP: $0	0.55(p)	0	0.41	0	0.03	0
WTP: $250	0.51	0	0.45(p)	0	0.04	0
WTP: $500	0.48	0	0.47(p)	0	0.04	0
WTP: $750	0.46	0	0.49(p)	0	0.05	0
WTP: $1000	0.45	0.01	0.49(p)	0.01	0.05	0
WTP: $1500	0.42	0.01	0.49(p)	0.02	0.06	0

[a] Detected deaths are those that occur due to treatment failure of loss-to-follow-up.
[b] Cost-effectiveness results are given for discounted DALYs and costs as per convention
[c] (p) is the preferred strategy; the strategy with the highest mean net monetary benefits

Table A: Summary of effects, costs, elimination of transmission (EoT) 2030, and cost-effectiveness with and without uncertainty in Kikongo health zone. Means are given along with 95% prediction intervals (PIs). YLL: years of life lost (to fatal disease), YLD: years of life lost to disability, DALYs: disability-adjusted life-years, PS: passive screening, AS: active screening, VC: vector control, ICER: incremental cost-effectiveness ratio, WTP: willingness to pay (USD per DALY averted), EoT: elimination of transmission.

	Mean AS	Int. AS	Mean AS + Targeted VC	Int. AS + Targeted VC	Mean AS + Full VC	Int. AS + Full VC
**Health effects**						
Reported cases	10 (0, 76)	10 (0, 78)	4 (0, 27)	4 (0, 29)	4 (0, 27)	4 (0, 28)
Deaths undetected	3 (0, 24)	3 (0, 21)	1 (0, 9)	1 (0, 8)	1 (0, 9)	1 (0, 8)
Cases total	14 (0, 97)	13 (0, 95)	5 (0, 32)	5 (0, 34)	5 (0, 32)	5 (0, 33)
Deaths detected[a]	0 (0, 0)	0 (0, 0)	0 (0, 0)	0 (0, 0)	0 (0, 0)	0 (0, 0)
YLD	5 (0, 34)	4 (0, 29)	2 (0, 9)	2 (0, 9)	2 (0, 9)	2 (0, 8)
YLL	146 (0, 1,086)	125 (0, 930)	59 (0, 387)	53 (0, 358)	58 (0, 382)	53 (0, 365)
DALYs	151 (0, 1,111)	129 (0, 960)	61 (0, 394)	55 (0, 364)	60 (0, 388)	55 (0, 372)
∆DALYs	0 (0, 0)	21 (-627, 780)	90 (-187, 928)	96 (-177, 942)	91 (-185, 941)	96 (-176, 938)
**Costs, in thousands US$ (not discounted)**						
AS costs	862 (273, 2438)	3030 (2410, 4038)	678 (273, 1496)	2959 (2405, 3822)	676 (273, 1468)	2958 (2405, 3818)
PS costs	700 (468, 1059)	694 (468, 1049)	664 (463, 952)	661 (464, 941)	663 (464, 947)	660 (463, 939)
VC costs	0 (0, 0)	0 (0, 0)	522 (117, 1410)	509 (116, 1357)	1037 (231, 2769)	1010 (232, 2681)
Treatment	5 (0, 40)	5 (0, 41)	2 (0, 15)	2 (0, 15)	2 (0, 14)	2 (0, 15)
Costs total	1567 (799, 3415)	3729 (3001, 4930)	1866 (960, 3549)	4131 (3189, 5593)	2378 (1101, 4808)	4630 (3346, 6743)
∆Costs	0 (0, 0)	2,162 (599, 3,181)	299 (-1,146, 1,621)	2,564 (1,039, 3,723)	811 (-687, 2,802)	3,063 (1,536, 4,771)
**EoT**						
Year of EoT	2027 (2021, 2040)	2027 (2021, 2039)	2026 (2021, 2032)	2026 (2021, 2031)	2026 (2021, 2031)	2026 (2021, 2031)
Prob EoT 2030	0.81	0.83	0.96	0.97	0.97	0.97
Prob EoT 2040	0.98	0.98	>0.99	>0.99	>0.99	>0.99
**Cost-effectiveness without uncertainty (discounted)[b]**						
∆DALYs	0	11	44	47	44	47
∆Costs	0	1,677,738	328,875	2,083,904	818,036	2,561,643
ICER	Min Cost	Dominated	7,504	514,693	Weakly Dominated	Dominated
**Cost-effectiveness with uncertainty (discounted), conditional on WTP[c].**						
WTP: $0	0.83(p)	0	0.14	0	0.03	0
WTP: $250	0.82(p)	0	0.14	0	0.03	0
WTP: $500	0.82(p)	0	0.15	0	0.03	0
WTP: $750	0.81(p)	0	0.15	0	0.03	0
WTP: $1000	0.81(p)	0	0.16	0	0.03	0
WTP: $1500	0.8(p)	0	0.16	0	0.03	0

[a] Detected deaths are those that occur due to treatment failure of loss-to-follow-up.
[b] Cost-effectiveness results are given for discounted DALYs and costs as per convention
[c] (p) is the preferred strategy; the strategy with the highest mean net monetary benefits

Table B: Summary of effects, costs, elimination of transmission (EoT) 2030, and cost-effectiveness with and without uncertainty in Kwamouth health zone. Means are given along with 95% prediction intervals (PIs). YLL: years of life lost (to fatal disease), YLD: years of life lost to disability, DALYs: disability-adjusted life-years, PS: passive screening, AS: active screening, VC: vector control, ICER: incremental cost-effectiveness ratio, WTP: willingness to pay (USD per DALY averted), EoT: elimination of transmission.

S5.5 Cost-effectiveness in Bas Uélé region: Ango and Doruma health zones

↩ Return to the Supplement Table of Contents

We also show detailed results for the two special health zones, Ango (Table C) and Doruma (Table D).

Because there are no activities simulated in these health zones, the status quo would detect zero cases and incur no costs. In Ango and Doruma, we estimate 939 [95% PI: 0–3098] and 980 [95% PI: 2–3249] deaths, respectively, leading to 42,516 [95% PI: 0–140,203] and 44,368 [95% PI: 89–148,286] DALYs, respectively. The lack of activities not only yields a high expected number of undetected cases, which are almost all resulting in deaths, but also a large degree of uncertainty.

If only one intervention were to be instituted, in terms of detecting cases *Restart PS* would be cost-effective for Ango and Doruma, in both health zones dominating the re-start of AS, therefore allowing more cases to be reported under PS at a lower cost. However, combinations of interventions are best, and the addition of *Int. AS* to any combination would substantially lower the number of total cases. Unfortunately, the probability of EoT by 2030 for both health zones is exceedingly low: 5%–13% in Ango and 5%–16% in Doruma, depending on the strategy. By 2040, there is an 8% and 10% chance of reaching EoT if nothing is done, but there is up to a 50% and 79% change of reaching EoT with the most ambitious strategy (*Int. AS* + *Full VC* + *Restart PS*).

In terms of cost-effectiveness in Ango and Doruma, no strategy would minimize costs, since nothing is assumed to be done and therefore nothing is being spent. However, the model indicates that *Restart PS* has a low ICER of $20/DALY averted in Ango and $7/DALY averted in Doruma. In Ango, *Int. AS* + *Restart PS*, would have an ICER of 356 and *Targeted VC* + *Restart PS* would have an ICER of $457/DALY, indicating cost-effectiveness at a moderately low WTP. In Ango, the only other efficient strategy would constitute the addition of AS (*Int. AS* + *Targeted VC* + *Restart PS*) has an ICER of $1,082/DALY–still under the threshold equivalent to 3 GDPs per capita. In Doruma, the only other sensible strategy after *Restart PS* is *Int. AS* + *Restart PS*, which has an ICER of %809/DALY averted.

The strategies that would maximize EoT in Ango are dominated because Full VC seems to spend more money without an advantage in terms of DALYs averted. In Doruma, such strategies are either dominated because they lack Int. AS that treats current cases, or are decidedly not cost-effective with an ICER of $24,713/DALY averted (*Int. AS* + *Full VC* + *Restart PS*).

Accounting for uncertainty in Ango, *Restart PS* would be the optimal strategy at a WTP <$250, and *Targeted VC* + *Restart PS* would be optimal at higher threholds. In Doruma, *Restart PS* would be the optimal strategy at $250<WTP<$1000; and above that, *Int. AS* + *Restart PS* would be optimal.

	No AS, PS, nor VC	Int. AS + no PS	Restart PS	Int. AS + Restart PS	Targeted VC + Restart PS	Int. AS + Targeted VC + Restart PS	Full VC + Restart PS	Int. AS + Full VC + Restart PS
**Health effects**								
Reported cases	0 (0, 0)	175 (0, 563)	214 (0, 594)	270 (0, 754)	57 (0, 152)	84 (0, 241)	57 (0, 154)	84 (0, 240)
Deaths undetected	939 (0, 3098)	555 (0, 1679)	484 (0, 1525)	307 (0, 946)	129 (0, 374)	89 (0, 259)	129 (0, 371)	89 (0, 256)
Cases total	939 (0, 3098)	730 (0, 2178)	698 (0, 2059)	576 (0, 1658)	186 (0, 503)	173 (0, 473)	186 (0, 505)	173 (0, 473)
Deaths detected[a]	0 (0, 0)	0 (0, 0)	0 (0, 1)	0 (0, 1)	0 (0, 0)	0 (0, 0)	0 (0, 0)	0 (0, 0)
YLD	556 (0, 1943)	333 (0, 1081)	320 (0, 1035)	207 (0, 656)	83 (0, 254)	58 (0, 174)	83 (0, 253)	58 (0, 172)
YLL	41,960 (0, 138,144)	24,808 (0, 75,258)	21,638 (0, 68,248)	13,719 (0, 42,248)	5,775 (0, 16,750)	3,987 (0, 11,553)	5,778 (0, 16,655)	3,991 (0, 11,461)
DALYs	42,516 (0, 140,203)	25,141 (0, 76,399)	21,958 (0, 69,203)	13,926 (0, 42,746)	5,858 (0, 17,001)	4,046 (0, 11,715)	5,861 (0, 16,903)	4,049 (0, 11,619)
ΔDALYs	0 (0, 0)	17,375 (-9,983, 80,409)	20,558 (-3,410, 86,446)	28,590 (0, 106,753)	36,658 (0, 124,071)	38,470 (0, 128,964)	36,655 (0, 124,191)	38,467 (0, 128,948)
**Costs, in thousands US$ (not discounted)**								
AS costs	0 (0, 0)	1437 (197, 2101)	0 (0, 0)	1537 (207, 2119)	0 (0, 0)	1230 (206, 1887)	0 (0, 0)	1231 (205, 1897)
PS costs	0 (0, 0)	0 (0, 0)	89 (60, 125)	89 (60, 125)	89 (60, 125)	89 (60, 125)	89 (60, 125)	89 (60, 125)
VC costs	0 (0, 0)	0 (0, 0)	0 (0, 0)	0 (0, 0)	3331 (850, 6645)	3259 (877, 6584)	6454 (1655, 12946)	6315 (1679, 12805)
Treatment	0 (0, 0)	86 (0, 280)	125 (0, 352)	145 (0, 403)	33 (0, 90)	45 (0, 127)	33 (0, 91)	45 (0, 127)
Costs total	0 (0, 0)	1523 (197, 2251)	214 (80, 443)	1771 (299, 2444)	3454 (943, 6792)	4624 (1278, 8369)	6576 (1752, 13087)	7681 (2110, 14593)
ΔCosts	0 (0, 0)	1,523 (197, 2,251)	214 (80, 443)	1,771 (299, 2,444)	3,454 (943, 6,792)	4,624 (1,278, 8,369)	6,576 (1,752, 13,087)	7,681 (2,110, 14,593)
**EoT**								
Year of EoT	2054 (2022, 2057)	2053 (2022, 2057)	2053 (2022, 2057)	2051 (2022, 2057)	2044 (2022, 2057)	2042 (2022, 2057)	2044 (2022, 2057)	2042 (2022, 2057)
Prob EoT 2030	0.05	0.06	0.06	0.08	0.10	0.13	0.10	0.13
Prob EoT 2040	0.08	0.12	0.13	0.19	0.44	0.50	0.44	0.50
**Cost-effectiveness without uncertainty (discounted)[b]**								
ΔDALYs	0	7,385	8,667	12,190	15,553	16,462	15,551	16,461
ΔCosts	0	1,231,854	170,039	1,425,562	2,961,219	3,945,076	5,641,839	6,578,235
ICER	Min Cost	Dominated	20	356	457	1,082	Dominated	Dominated
**Cost-effectiveness with uncertainty (discounted), conditional on WTP[c].**								
WTP: \$0	1(p)	0	0	0	0	0	0	0
WTP: \$250	0.1	0.02	0.63(p)	0.16	0.06	0.01	0	0
WTP: \$500	0.08	0.03	0.35	0.24(p)	0.21	0.09	0	0
WTP: \$750	0.07	0.03	0.21	0.21	0.28(p)	0.19	0	0
WTP: \$1000	0.06	0.03	0.15	0.18	0.29(p)	0.28	0	0
WTP: \$1500	0.05	0.02	0.1	0.13	0.27	0.41(p)	0.01	0.01

[a] Detected deaths are those that occur due to treatment failure of loss-to-follow-up.
[b] Cost-effectiveness results are given for discounted DALYs and costs as per convention
[c] (p) is the preferred strategy; the strategy with the highest mean net monetary benefits

Table C: Summary of effects, costs, elimination of transmission (EoT) 2030, and cost-effectiveness with and without uncertainty in Ango health zone. Means are given along with 95% prediction intervals (PIs). YLL: years of life lost (to fatal disease), YLD: years of life lost to disability, DALYs: disability-adjusted life-years, PS: passive screening, AS: active screening, VC: vector control, ICER: incremental cost-effectiveness ratio, WTP: willingness to pay (USD per DALY averted), EoT: elimination of transmission.

	No AS, PS, nor VC	Int. AS + no PS	Restart PS	Int. AS + Restart PS	Full VC + Restart PS	Int. AS + Full VC + Restart PS
**Health effects**						
Reported cases	0 (0, 0)	103 (0, 411)	117 (0, 397)	114 (0, 369)	113 (0, 374)	111 (0, 360)
Deaths undetected	980 (2, 3249)	630 (1, 2233)	128 (0, 461)	87 (0, 278)	123 (0, 439)	84 (0, 265)
Cases total	980 (2, 3249)	733 (1, 2539)	245 (1, 832)	201 (1, 634)	236 (1, 802)	195 (1, 610)
Deaths detected[a]	0 (0, 0)	0 (0, 0)	0 (0, 0)	0 (0, 0)	0 (0, 0)	0 (0, 0)
YLD	572 (1, 2085)	371 (0, 1403)	83 (0, 309)	57 (0, 194)	80 (0, 298)	55 (0, 187)
YLL	43,796 (86, 146,530)	28,156 (44, 99,475)	5,703 (0, 20,565)	3,895 (0, 12,423)	5,491 (0, 19,694)	3,777 (0, 11,933)
DALYs	44,368 (89, 148,286)	28,527 (44, 100,864)	5,786 (1, 20,846)	3,953 (0, 12,621)	5,571 (1, 20,015)	3,832 (0, 12,080)
ΔDALYs	0 (0, 0)	15,842 (-29,798, 94,177)	38,582 (0, 131,662)	40,416 (0, 138,235)	38,797 (0, 132,572)	40,536 (0, 138,415)
**Costs, in thousands US$ (not discounted)**						
AS costs	0 (0, 0)	847 (179, 1353)	0 (0, 0)	850 (238, 1301)	0 (0, 0)	840 (226, 1297)
PS costs	0 (0, 0)	0 (0, 0)	80 (52, 115)	80 (52, 115)	80 (52, 115)	80 (52, 115)
VC costs	0 (0, 0)	0 (0, 0)	0 (0, 0)	0 (0, 0)	1699 (409, 3262)	1579 (415, 3107)
Treatment	0 (0, 0)	51 (0, 205)	62 (0, 213)	59 (0, 190)	60 (0, 202)	57 (0, 186)
Costs total	0 (0, 0)	898 (179, 1464)	142 (68, 295)	989 (319, 1511)	1839 (489, 3442)	2556 (729, 4414)
ΔCosts	0 (0, 0)	898 (179, 1,464)	142 (68, 295)	989 (319, 1,511)	1,839 (489, 3,442)	2,556 (729, 4,414)
**EoT**						
Year of EoT	2054 (2026, 2057)	2052 (2026, 2057)	2038 (2026, 2057)	2036 (2025, 2051)	2038 (2026, 2057)	2036 (2025, 2051)
Prob EoT 2030	0.05	0.06	0.12	0.16	0.13	0.16
Prob EoT 2040	0.10	0.15	0.66	0.77	0.67	0.79
**Cost-effectiveness without uncertainty (discounted)[b]**						
ΔDALYs	0	6,619	16,140	17,010	16,236	17,064
ΔCosts	0	734,178	118,386	822,180	1,531,753	2,145,123
ICER	Min Cost	Dominated	7	809	Dominated	24,713
**Cost-effectiveness with uncertainty (discounted), conditional on WTP[c].**						
WTP: $0	1(p)	0	0	0	0	0
WTP: $250	0.06	0	0.83(p)	0.1	0	0
WTP: $500	0.05	0.01	0.69(p)	0.24	0.01	0
WTP: $750	0.04	0.01	0.58(p)	0.32	0.02	0.02
WTP: $1000	0.04	0.01	0.51	0.37(p)	0.04	0.04
WTP: $1500	0.04	0.01	0.42	0.4(p)	0.06	0.08

[a] Detected deaths are those that occur due to treatment failure of loss-to-follow-up.
[b] Cost-effectiveness results are given for discounted DALYs and costs as per convention
[c] (p) is the preferred strategy; the strategy with the highest mean net monetary benefits

Table D: Summary of effects, costs, elimination of transmission (EoT) 2030, and cost-effectiveness with and without uncertainty in Doruma health zone. Means are given along with 95% prediction intervals (PIs). YLL: years of life lost (to fatal disease), YLD: years of life lost to disability, DALYs: disability-adjusted life-years, PS: passive screening, AS: active screening, VC: vector control, ICER: incremental cost-effectiveness ratio, WTP: willingness to pay (USD per DALY averted), EoT: elimination of transmission.

S5.6 Results by coordination

↩ Return to the Supplement Table of Contents

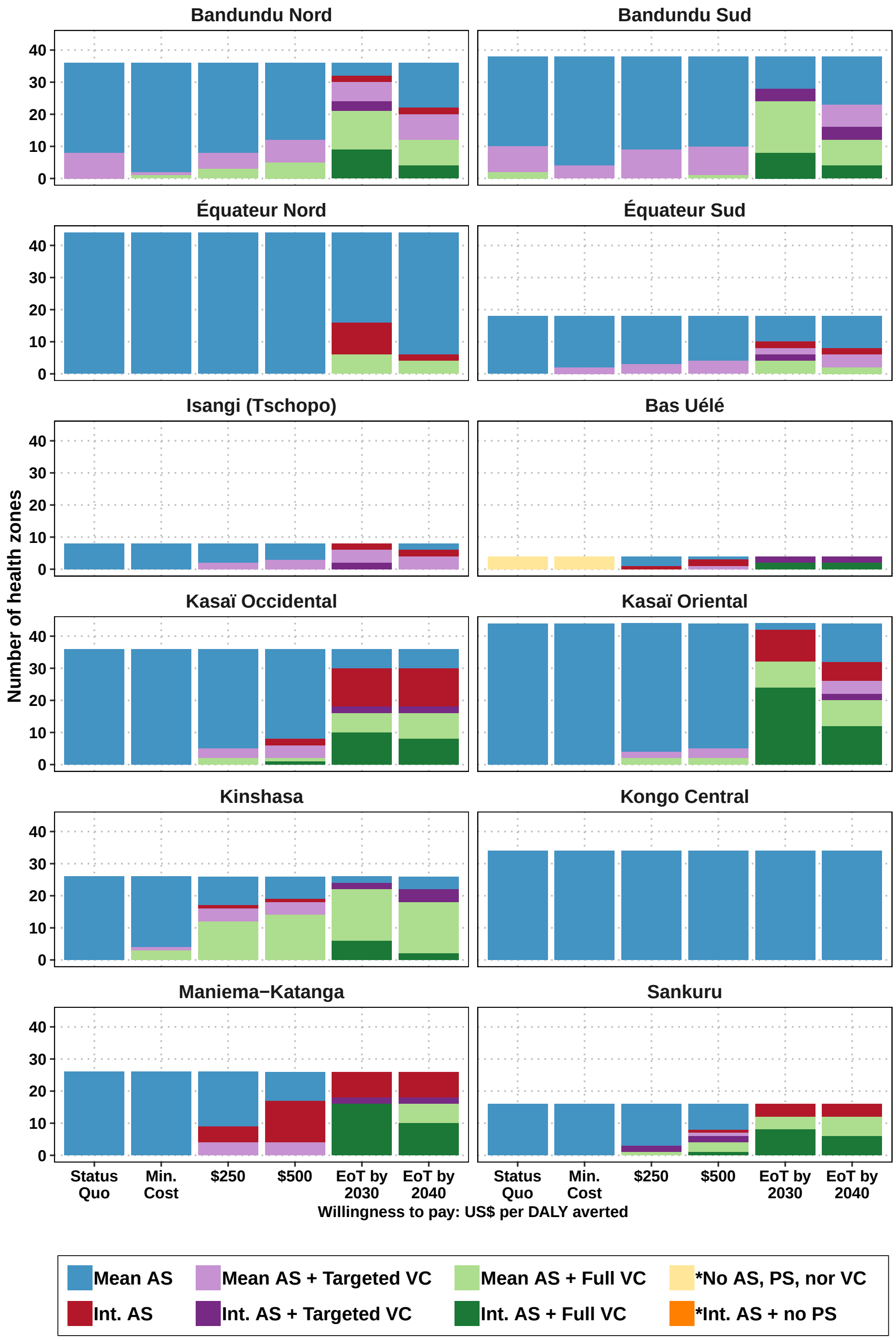

Fig E: Histrogram of optimal strategies by coordination. Abbreviations: AS: active screening, PS: passive screening, VC: vector control, DALY: disability-adjusted life-year, EoT: elimination of transmission.

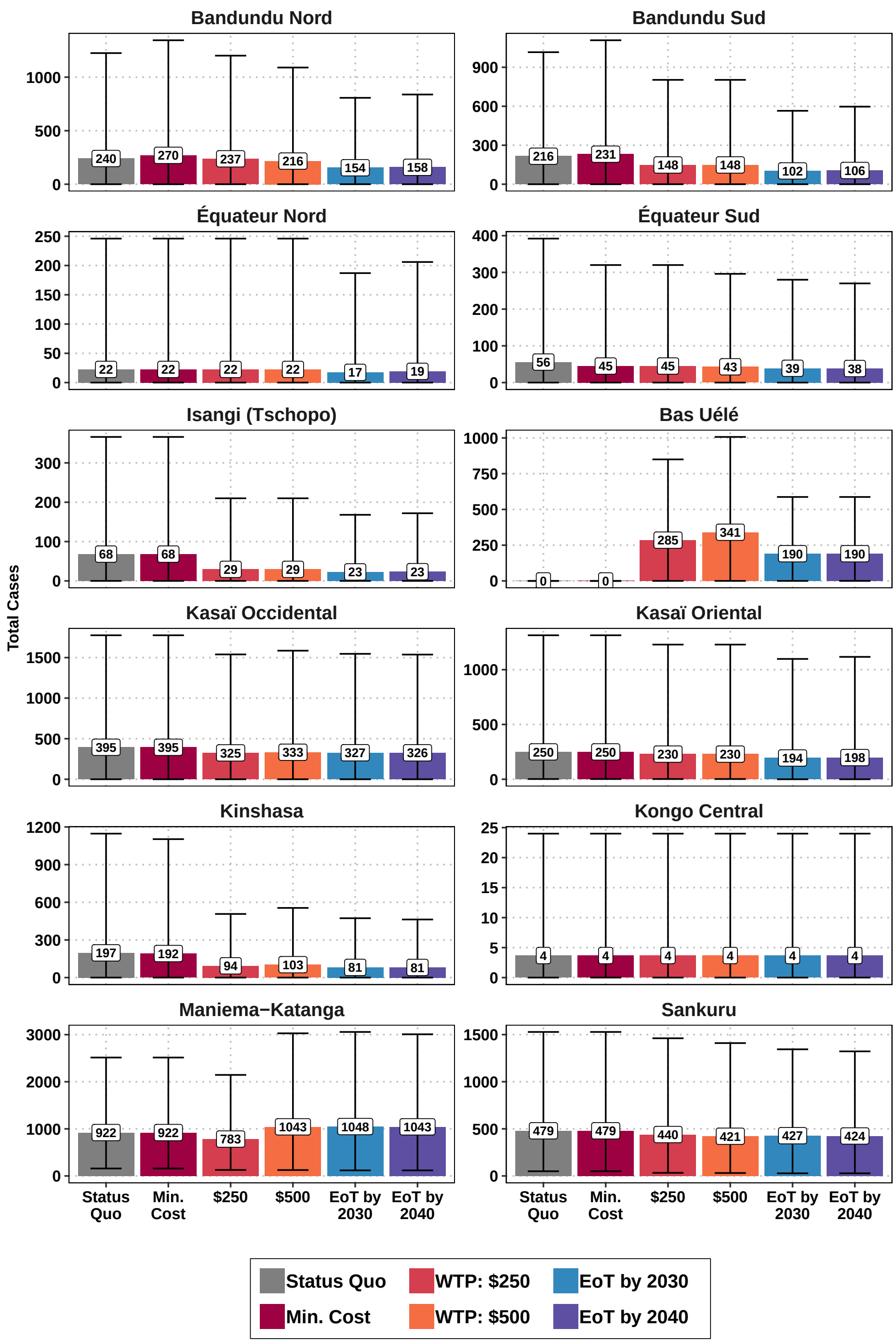

Fig F: Total cases reported 2026-2040 at different levels of investment. Abbreviations: DALY: disability-adjusted life-year, WTP: willingness-to-pay (denominated in USD per DALY averted), EoT: elimination of transmission.

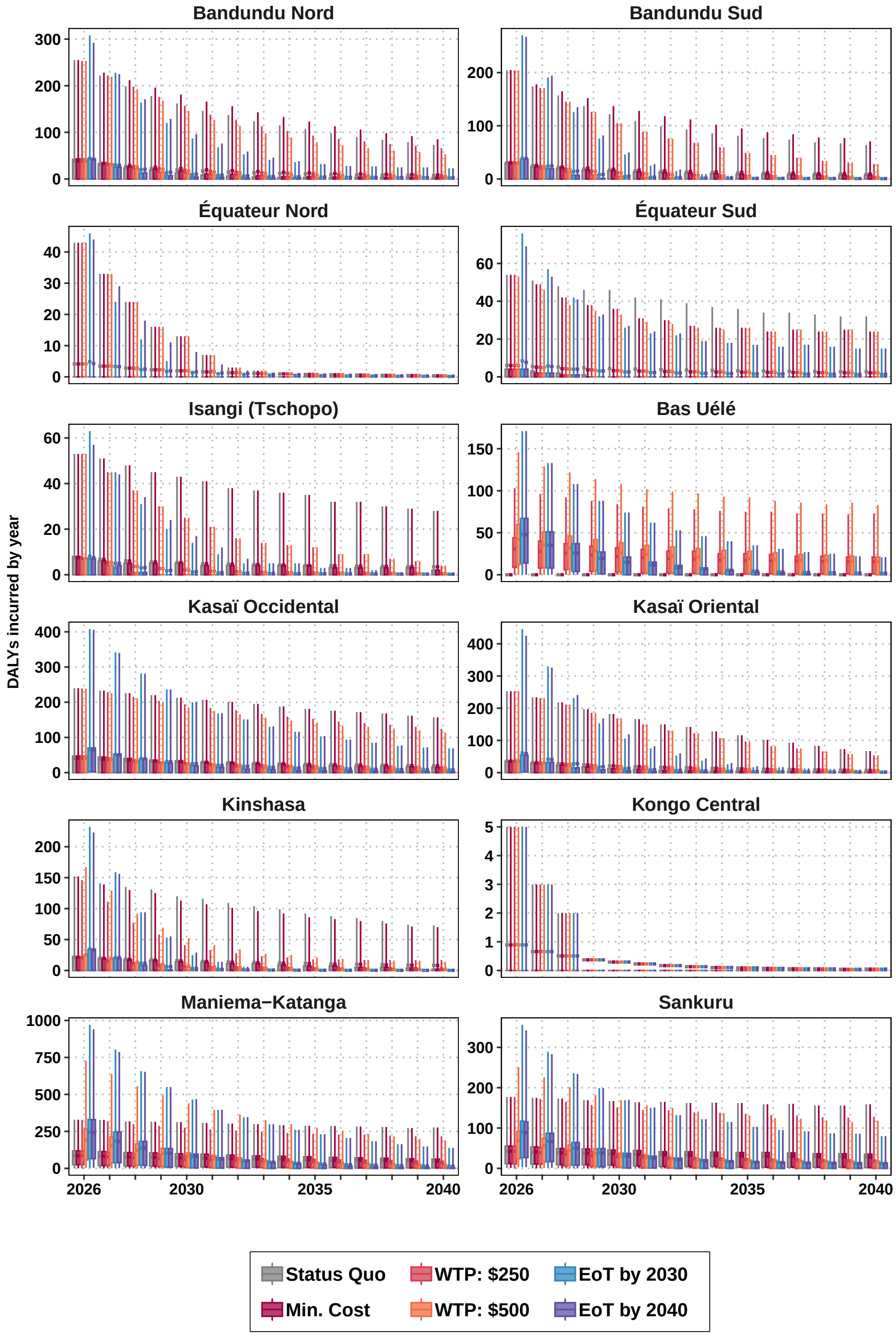

Fig G: Cases by year 2026-2040 at different levels of investment.Abbreviations: DALY: disability-adjusted life-year, WTP: willingness-to-pay (denominated in USD per DALY averted), EoT: elimination of transmission.

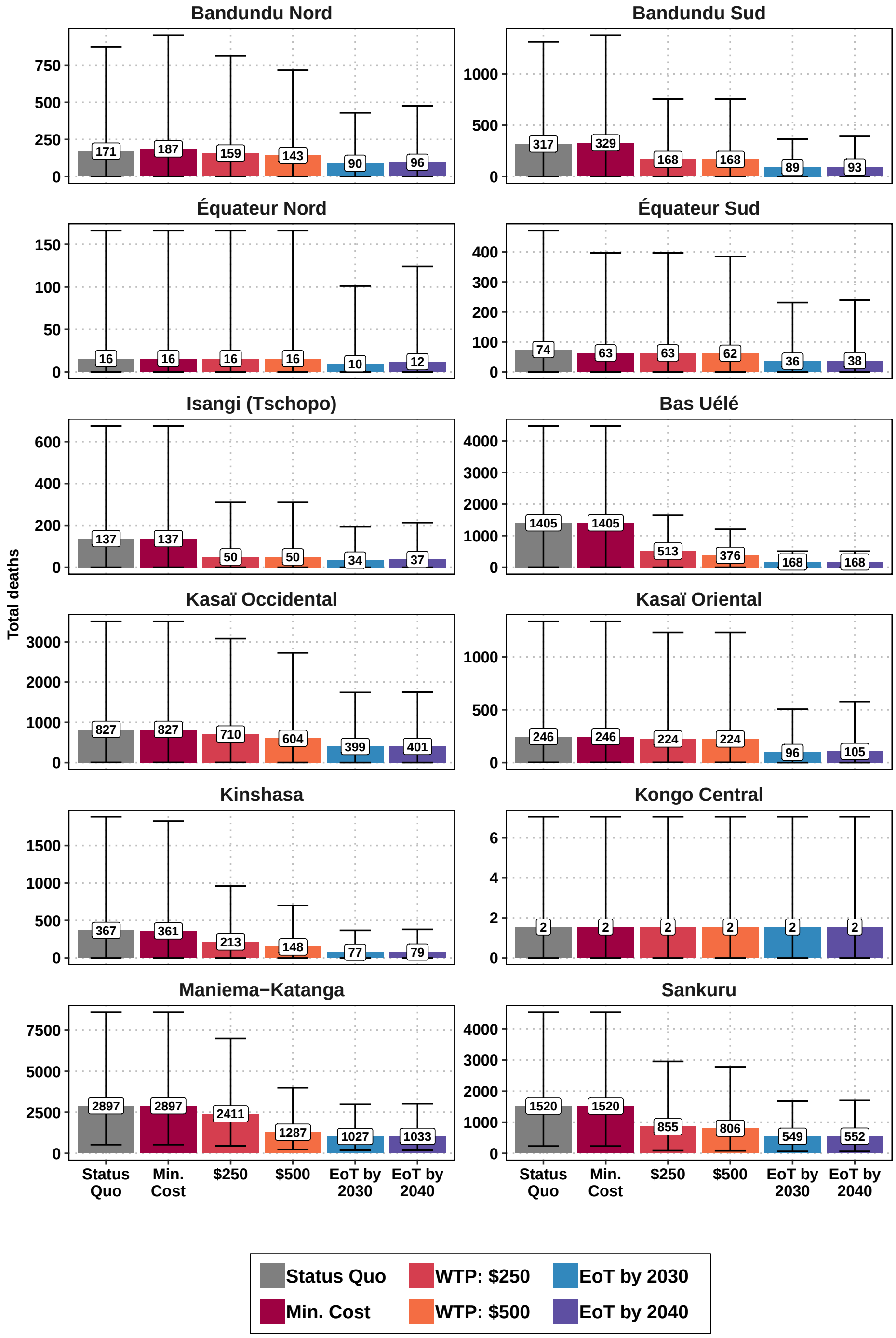

Fig H: Total deaths 2026-2040 at different levels of investment. Abbreviations: DALY: disability-adjusted life-year, WTP: willingness-to-pay (denominated in USD per DALY averted), EoT: elimination of transmission.

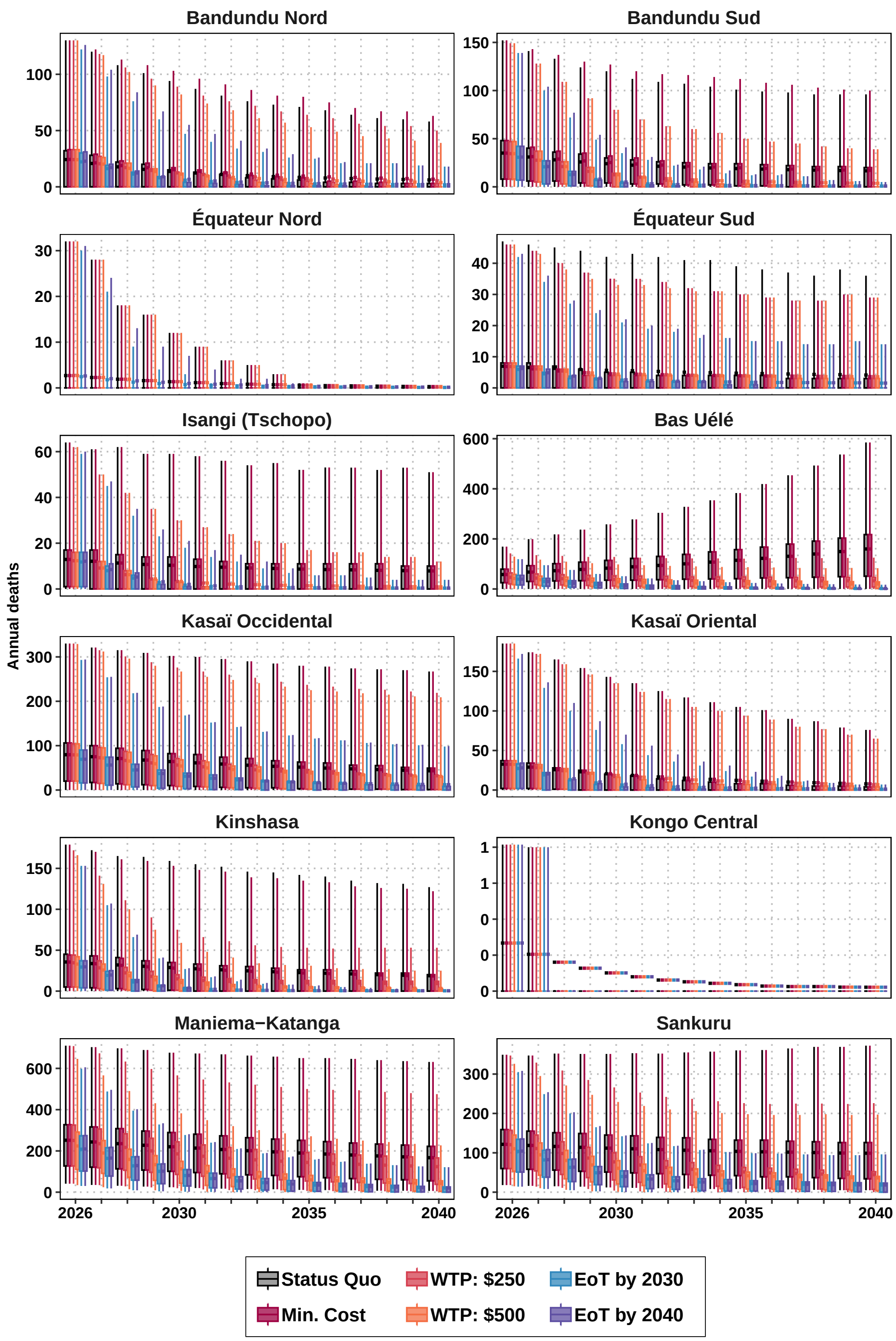

Fig I: Deaths by year 2026-2040 at different levels of investment. Abbreviations: DALY: disability-adjusted life-year, WTP: willingness-to-pay (denominated in USD per DALY averted), EoT: elimination of transmission.

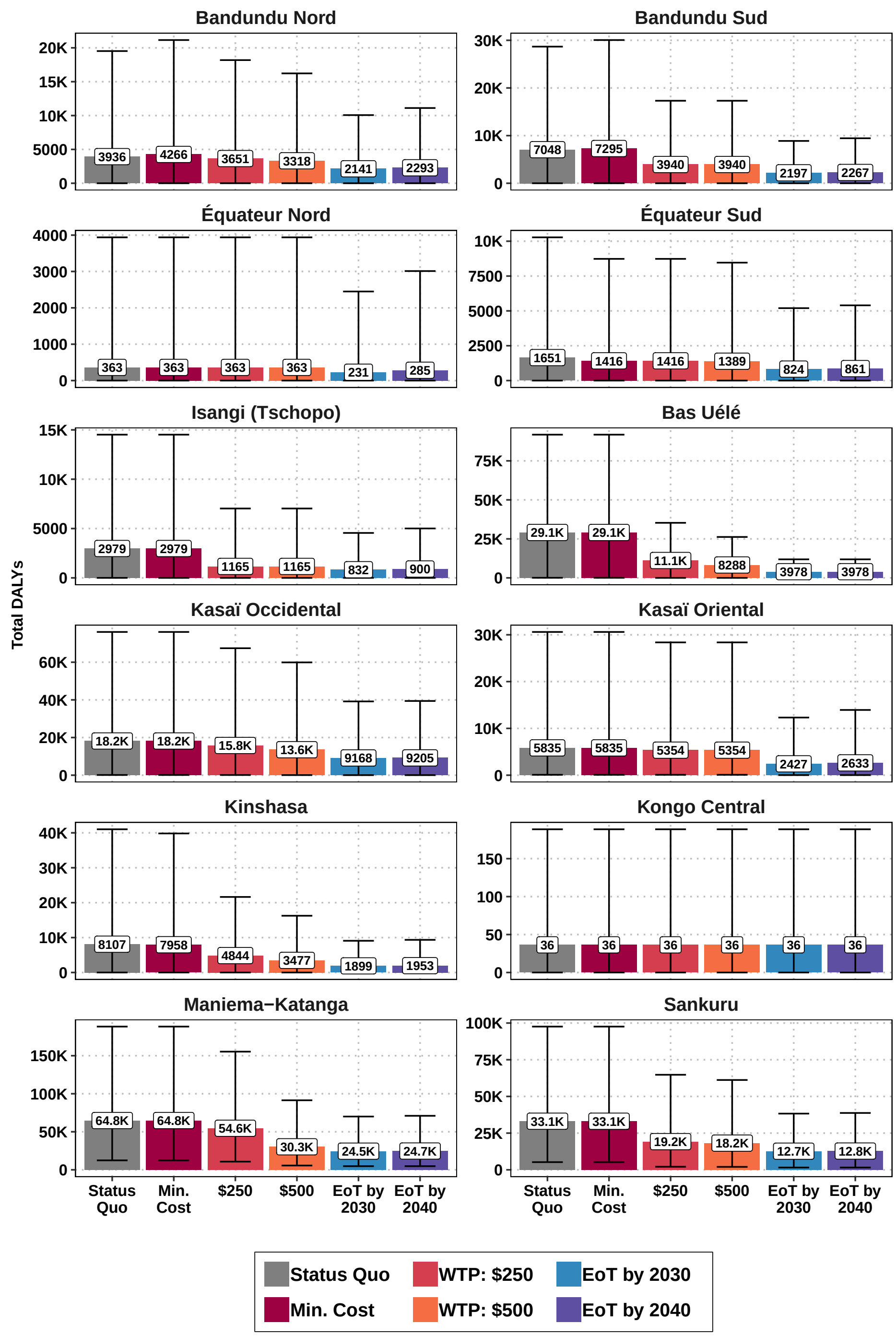

Fig J: Total DALYs 2026-2040 at different levels of investment. Abbreviations: DALY: disability-adjusted life-year, WTP: willingness-to-pay (denominated in USD per DALY averted), EoT: elimination of transmission.

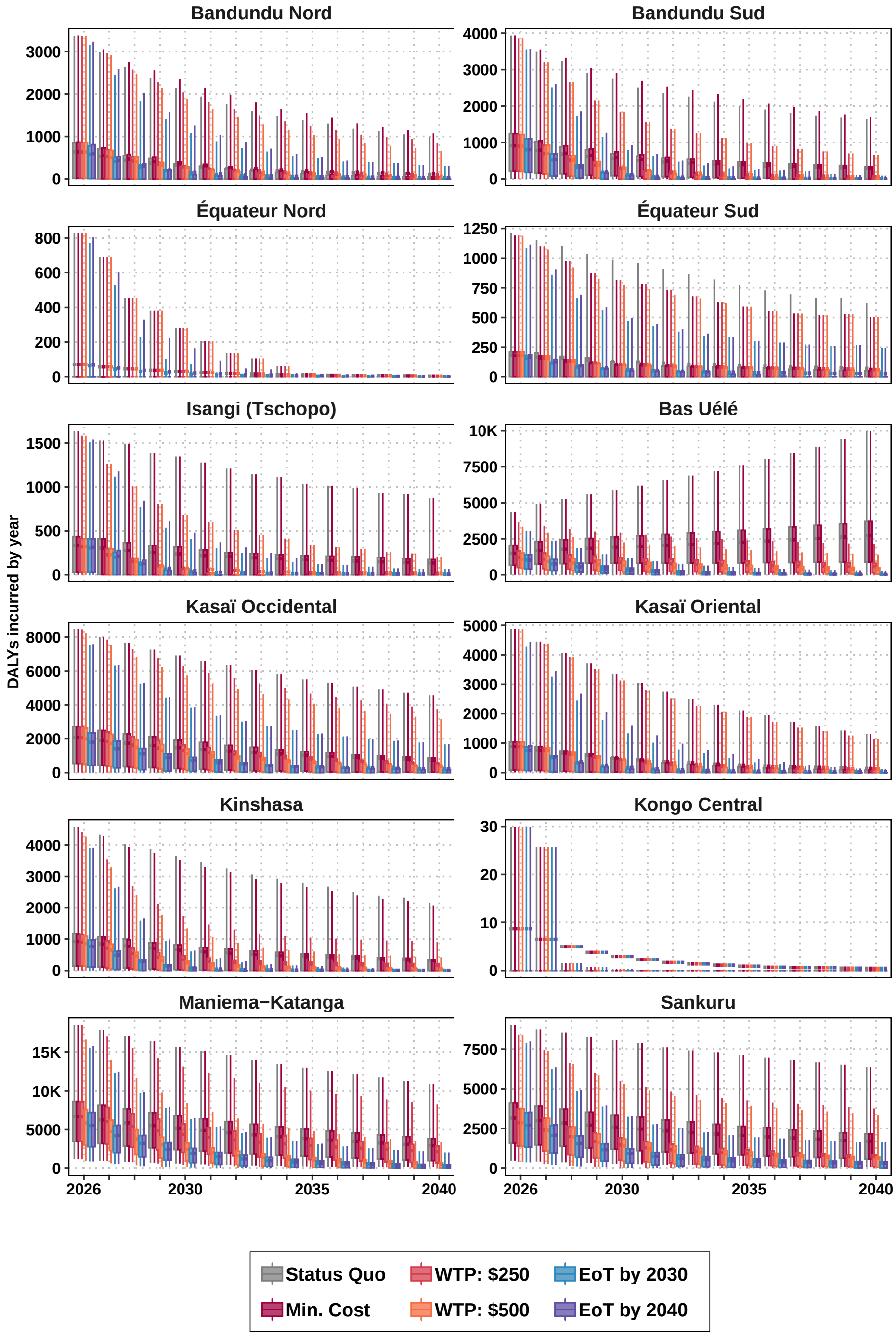

Fig K: DALYs by year 2026-2040 at different levels of investment. Abbreviations: DALY: disability-adjusted life-year, WTP: willingness-to-pay (denominated in USD per DALY averted), EoT: elimination of transmission.

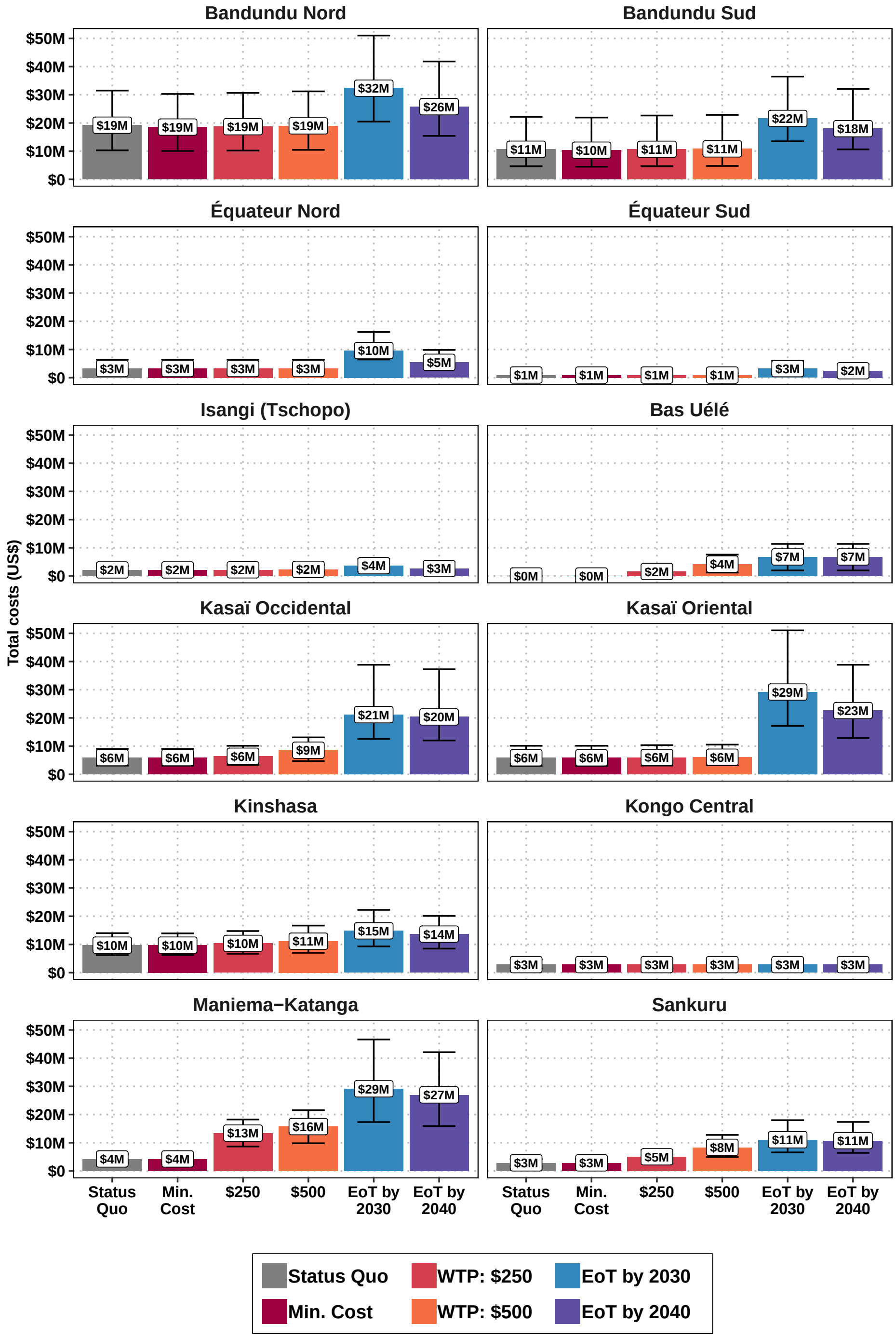

Fig L: Total costs 2026-2040 at different levels of investment. Abbreviations: DALY: disability-adjusted life-year, WTP: willingness-to-pay (denominated in USD per DALY averted), EoT: elimination of transmission.

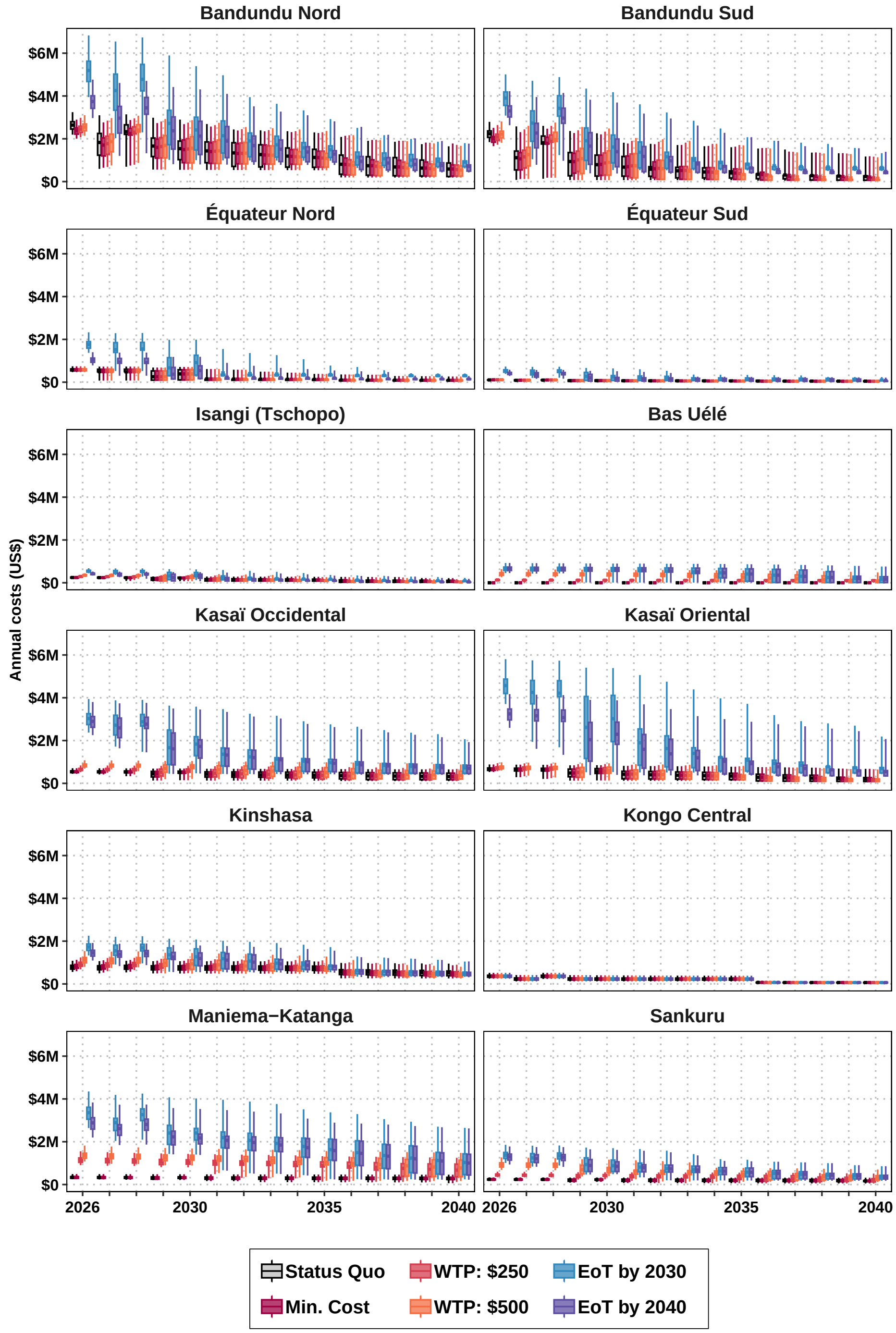

Fig M: Total costs by year 2026-2040 at different levels of investment. Abbreviations: DALY: disability-adjusted life-year, WTP: willingness-to-pay (denominated in USD per DALY averted), EoT: elimination of transmission.

S5.7 Resource forecasts

↩ Return to the Supplement Table of Contents

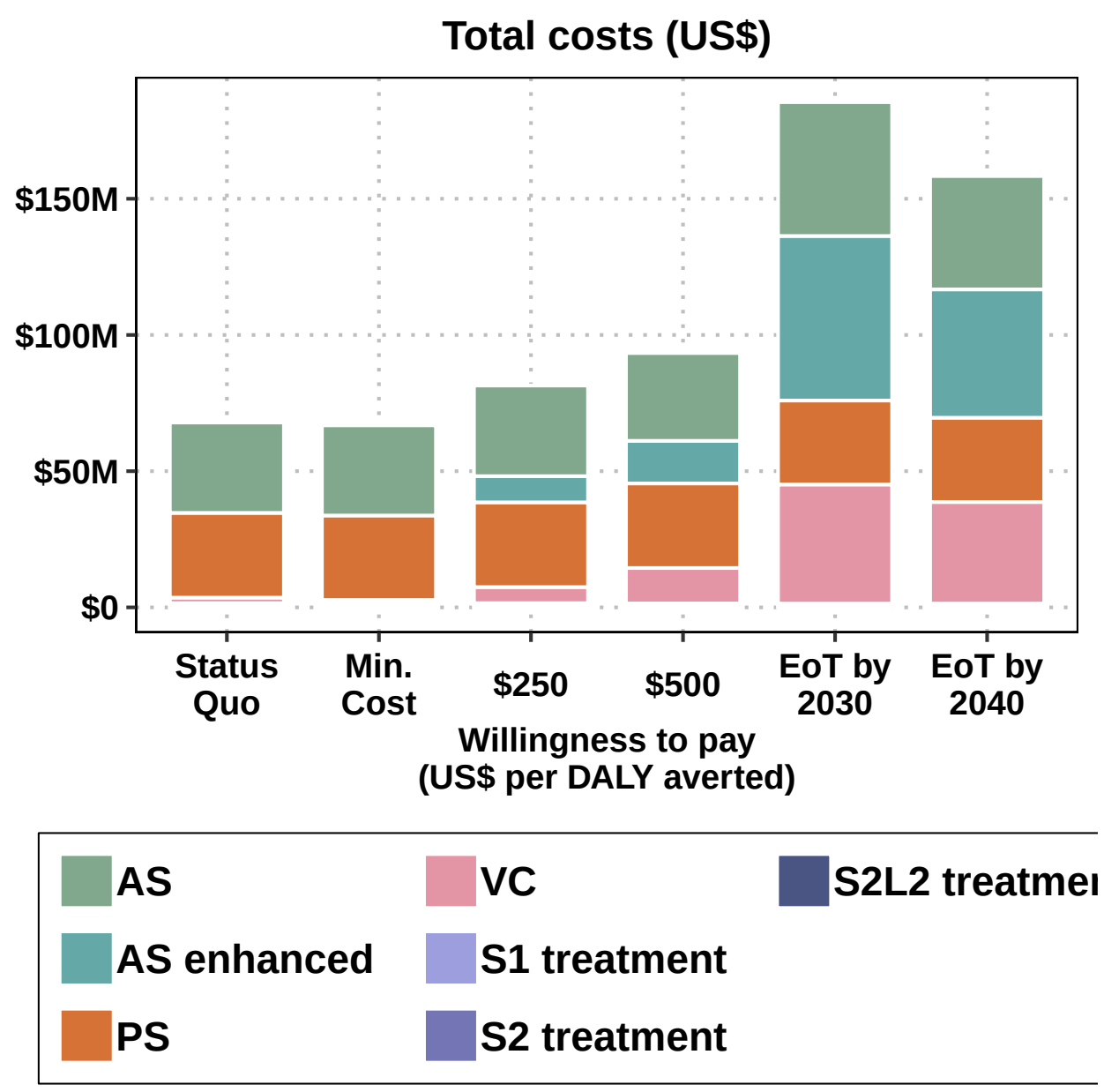

Fig N: Costs allocated to different activities, at different levels of investment, for the period 2026-2040. Abbreviations: AS: active screening, PS: passive screening, VC: vector control, S1: Stage 1 disease, S2: Stage 2 disease, S2L2: Stage 2 rescue medicine, DALY: disability-adjusted life-year, EoT: elimination of transmission.

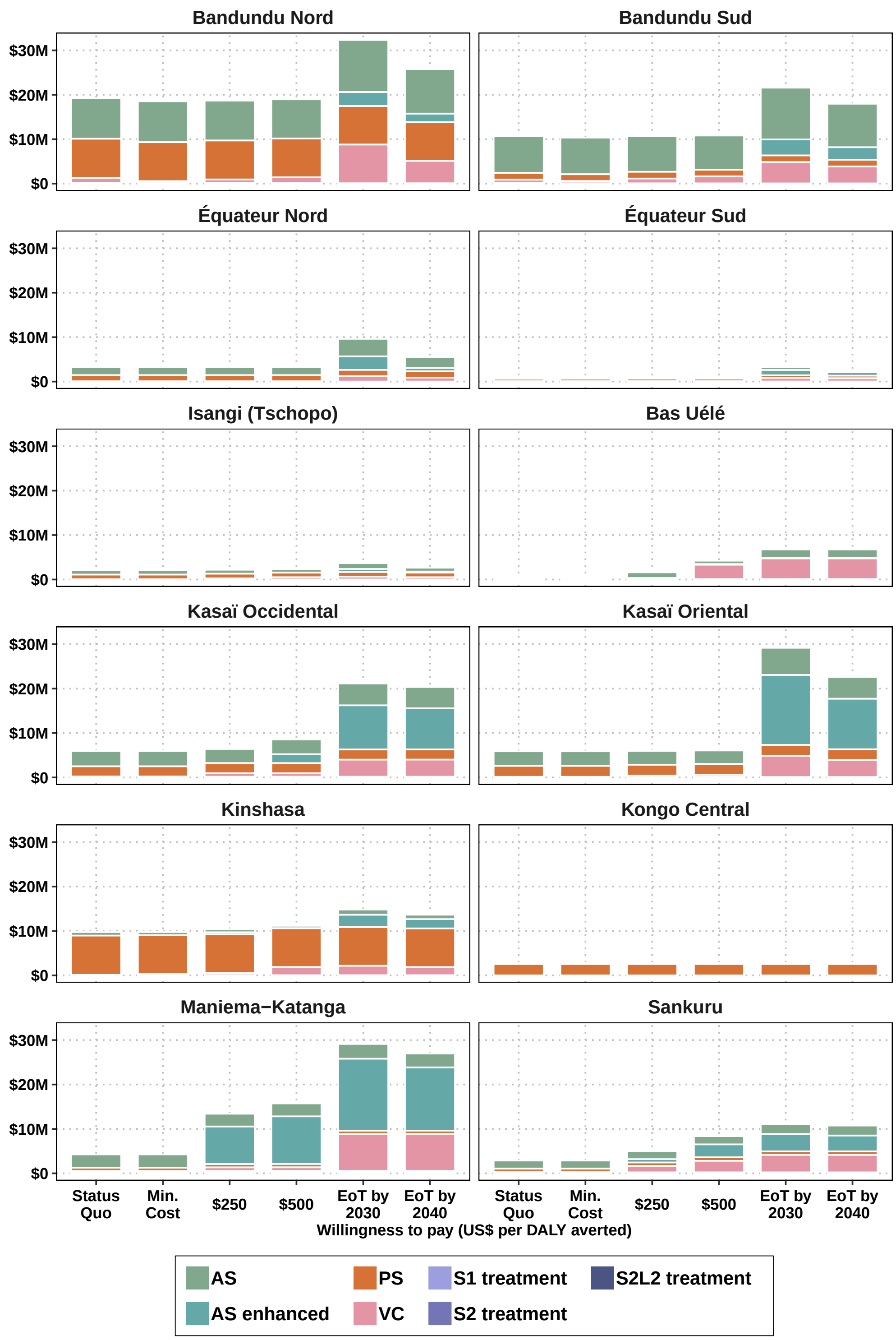

Fig O: Costs allocated to different activities, at different levels of investment by coordination, for the period 2026-2040. Abbreviations: AS: active screening, PS: passive screening, VC: vector control, S1: Stage 1 disease, S2: Stage 2 disease, S2L2: Stage 2 rescue medicine, DALY: disability-adjusted life-year, EoT: elimination of transmission.

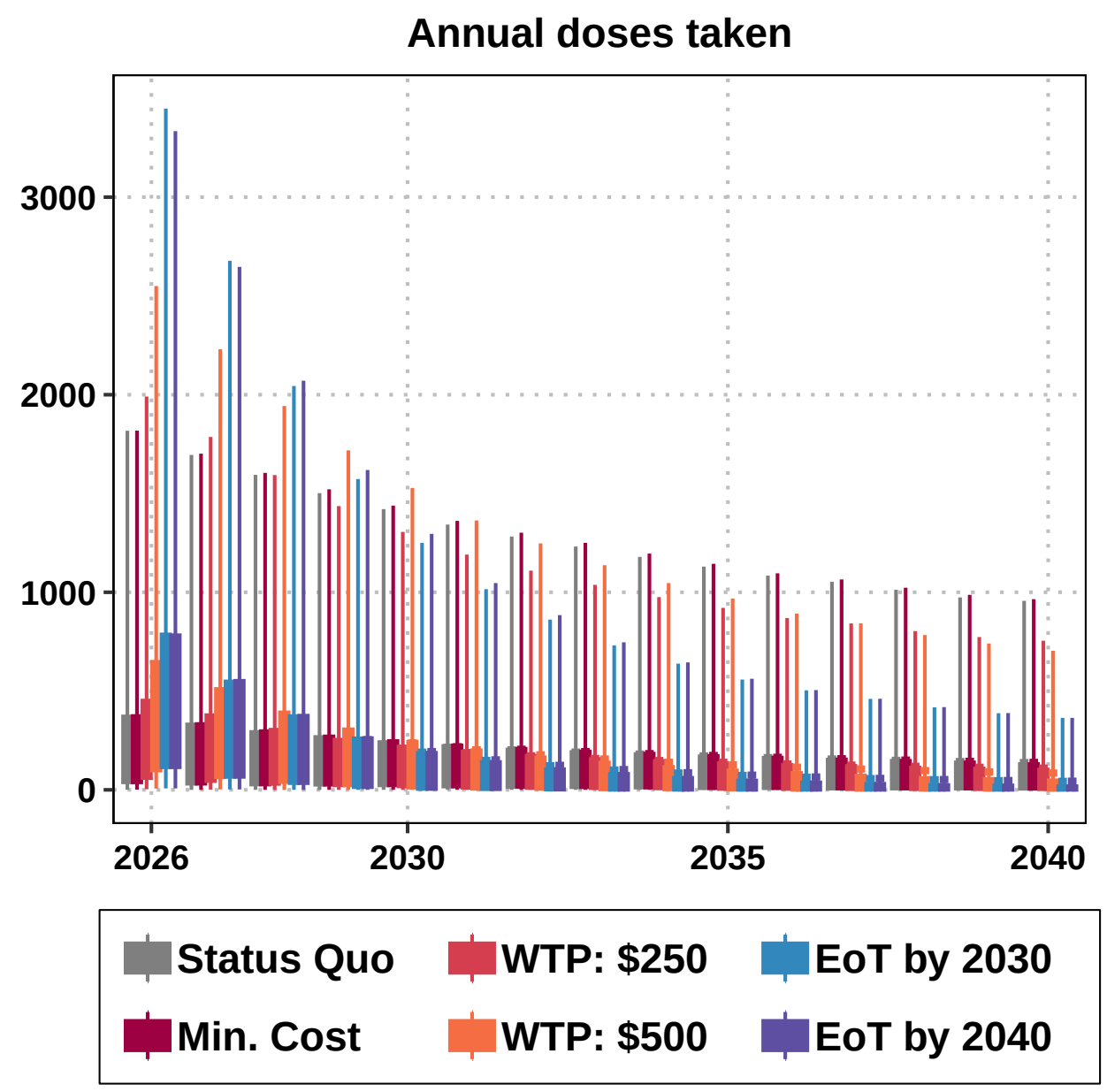

Fig P: Drugs used by year 2026-2040 at different levels of investment. Abbreviations: DALY: disability-adjusted life-year, WTP: willingness-to-pay (denominated in USD per DALY averted), EoT: elimination of transmission.

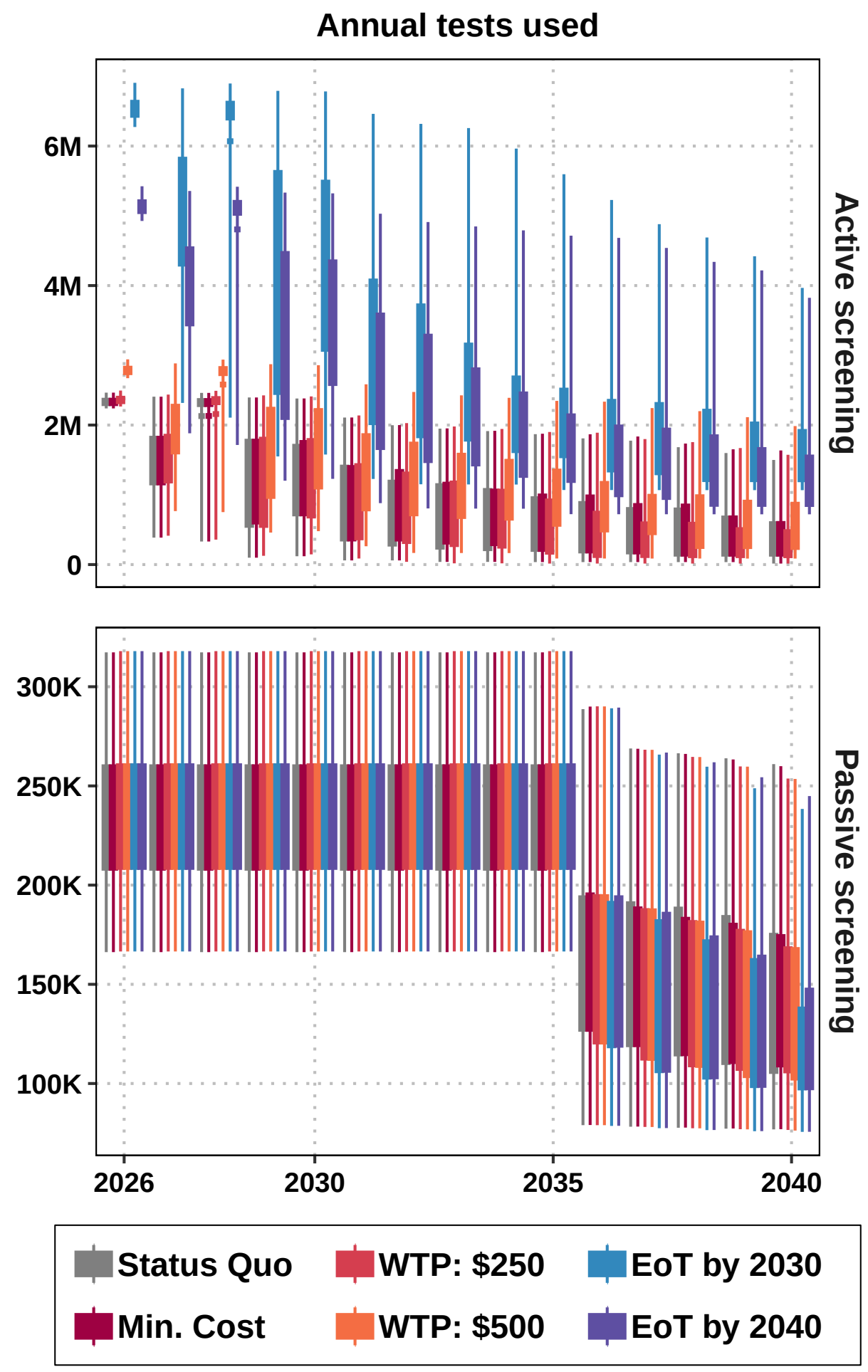

Fig Q: Tests used by year 2026-2040 at different levels of investment. Abbreviations: DALY: disability-adjusted life-year, WTP: willingness-to-pay (denominated in USD per DALY averted), EoT: elimination of transmission.
