# Supplementary material for: Cost-effectiveness of end-game strategies against sleeping sickness across the Democratic Republic of Congo
Source: PLoS Negl Trop Dis. 2026 Oct 22;20(10):e0014777. doi: 10.1371/journal.pntd.0014777 (PMC13719019; doi:10.1371/journal.pntd.0014777)
Supplement: Supplementary file 6 [file pntd.0014777.s006.pdf]

PRIME-NTD Criteria

Principle and what has been done to satisfy the principle?	Where in the manuscript is this described?
**1. Stakeholder engagement**<br>Strategy components were determined along with the country director of PNLTHA, Erick Miaka (co-author). Implementation of simulations of AS and PS costs was aided by Rian Snijders, who has helped run field operations in former Bandundu. Implementation of costs of VC was aided by Andrew Hope, Iñaki Tirados, Sophie Dunkley, and Rian Snijders and collaborators at the Liverpool School of Tropical Medicine, who have run field operations in DRC since 2015.	Authorship list and acknowledgements
**2. Complete model documentation**<br>Full model (including the fitting code) and documentation are available through OpenScienceFramework (OSF). The epidemiological model is fully described in the fitting study of Crump et al. [1] and cost model in the Supplementary Methods.	Description in S1 Text, Sections S1.1–S1.9 access the code via `DRC_wholecountryCEA`[1].
**3. Complete description of data used**<br>Information about the data used for fitting is described in Crump et al. [1]. The data used for clinical outcomes and costs were estimates from the literature. No data from intervention operations was used. Assumptions and estimates were parameterised according to conventions in the economic evaluation literature [2].	For assumptions around intervention, treatment effects and costs, see S1 Text, Sections S1.7, S1.9, and S1.10.
**4. Communicating uncertainty**<br>*Structural uncertainty*:<br>Uncertainty arising from the choice of vector control operation inputs, discounting, and time horizon are shown by re-running the entire analysis with alternative assumptions.<br>*Parameter uncertainty*:<br>The epidemiological parameters are the posterior distributions of a model fitted to time-series data, and full details are available in another publication [1]. For the parameters to model health outcomes and costs, assumptions and estimates were parameterized according to conventions in the economic evaluation literature [2], taking care to sample from large distributions for aspects for which we knew very little.<br>*Prediction uncertainty*:<br>Observational uncertainty in epidemiological model predictions. Figures 4 present both means and 95% prediction intervals. The `GUI`[2] includes box and whisker plots to show uncertainty in cases, deaths and DALYs. We include the probability of meeting EOT by 2030 as well as the expected year of EOT. For cost-effectiveness results, we present the optimal decisions (the probability of each strategy being cost-effective) at different willingness-to-pay thresholds rather than solely providing ICERs using the net benefits framework (see Tables A, B, C, D).	Main text results and discussion. Results with alternative time horizons and discounting are available in our `GUI`[2].<br><br>Epidemiological parameters are available on OSF `DRC_wholecountryCEA` [1]. Health outcome and cost-effectiveness parameters: see S1 Text, Sections S1.9 and S1.10, and Supplementary Note 4.<br><br>The `GUI`[4].
**5. Testable model outcomes**<br>Epidemiological model outputs are routinely reported metrics of the disease course: detected active and passive case detections. Therefore, these predictions can be compared to future data as long as the data is put into context alongside measures of active screening coverage and the number of fixed health posts equipped for passive screening. Some components of cost predictions can be validated against expenditures, but it must be noted that these are economic costs, and so resource use for which there is no explicit invoice is taken into account as well.	Epidemiological projections for the period 2026-2040 are shown in Figure 4 for the whole country, and by coordination (aggregated and by year in Figures F-M. Epidemiological outcomes for each year until 2050 and by coordination and health zone can be viewed in the `GUI`[2]. All the ingredients to the economic costs were shown in detail in the S1 Text, Section S1.10.

[1] `DRC_wholecountryCEA` with full address: `https://osf.io/ezjxb/`.
[2] `GUI` with full address: `https://hatmepp.warwick.ac.uk/DRCCEA/v8/`

PRIME-NTD criteria fulfilment. We summarise how the NTD Modelling Consortium's "5 key principles of good modelling practice" have been met in the present study.
