# Supplementary material for: Cost-effectiveness of end-game strategies against sleeping sickness across the Democratic Republic of Congo
Source: PLoS Negl Trop Dis. 2026 Oct 22;20(10):e0014777. doi: 10.1371/journal.pntd.0014777 (PMC13719019; doi:10.1371/journal.pntd.0014777)
Supplement: Supplementary file 7 [file pntd.0014777.s007.pdf]

CHEERS Checklist

Section/item	Item No	Recommendation	Reported on page no, line no
**Title**			
Title	1	Identify the study as an economic evaluation or use more specific terms such as "cost-effectiveness analysis", and describe the interventions compared.	Cover page
**Abstract**			
Abstract	2	Provide a structured summary of objectives, perspective, setting, methods (including study design and inputs), results (including base case and uncertainty analyses), and conclusions.	Abstract
**Introduction**			
Background and objectives	3	Provide an explicit statement of the broader context for the study. Present the study question and its relevance for health policy or practice decisions.	Introduction section, in particular the second-to-last and the last paragraph.
**Methods**			
Health economic analysis plan	4	Indicate whether a health economic analysis plan was developed and where available.	No previous protocol was published.
Study population	5	Describe characteristics of the study population (such as age range, demographics, socioeconomic, or clinical characteristics).	The population is described in the Supplementary Methods, Section S1.3 and Tables B, and C.
Settings and location	6	Provide relevant contextual information that may influence findings.	The location is described in the Supplementary Methods, Section S1.3 and Tables B, and C.
Comparators	7	Describe the interventions or strategies being compared and state why they were chosen.	Figure 1; second paragraph of the Results section; third section of the methods; and more detail in the Supplementary Methods, Section S1.7.2, Table H, Figure C, and Table I.
Perspective	8	State the perspective(s) adopted by the study and the rationale.	Fourth subsection of the methods, "Cost-effectiveness analysis", in a subsection called "Costs".
Time horizon	9	State the time horizon over and why the horizon is appropriate.	Fourth subsection of the methods, "Cost-effectiveness analysis", in a subsection called "Economic evaluation and investment horizon".
Discount rate	10	Report the discount rate(s) and reason chosen.	Fourth subsection of the methods, "Cost-effectiveness analysis", in a subsection called "Economic evaluation and investment horizon". 3% is the recommended rate by WHO-CHOICE and the Gates Reference Case.
Selection of outcomes	11	Describe what outcomes were used as the measure(s) of benefit(s) and harm(s).	Fourth subsection of the methods, "Cost-effectiveness analysis", in a subsection called "Health outcomes".

(continued)

Section/item	Item No	Recommendation	Reported on page no, line no
Measurement of outcomes	12	Describe how outcomes used to capture benefit(s) and harm(s) were measured.	Outcomes were not measured, but were simulated. See the fourth subsection of the methods, "Outcome metrics". Effectiveness of AS, PS and VC strategies: model-based, treatment was sourced from the literature. Described in detail in Supplementary Methods, Section S1.10.4, Tables T-U, and Supplementary Note 4, Section S4.6.
Valuation of outcomes	13	Describe the population and methods used to measure and value outcomes.	Like in previous analyses [1, 2], see the Supplementary Methods, Sections S1.9 and **??**, which contain detailed explanations of how DALYs are calculated and how the natural history of HAT was considered.
Measurement and valuation of resources and costs	14	If applicable, describe the population and methods used to elicit preferences for outcomes.	The construction of programme costs are detailed in Supplementary Methods, Section S1.10. As can be seen, in a publication of this scope, detailing the cost inputs in the main body would be unfeasible. However, the resulting expected costs are in Figure 4 of the main body of the paper, as well as in N broken down by activity, and in O broken down by coordination. In the GUI, one may find the costs broken down by activity by health zone, coordination, and the country for under a variety of sensitivity analyses.
Currency, price date, and conversion	15	Report the dates of the estimated resource quantities and unit costs, plus the currency and year of conversion.	Fourth subsection of the methods, “Cost-effectiveness analysis”, in a subsection called “Costs”. Our general approach for this is in the section "Principles for parameterization" in Supplementary Note 4, Section S4.1, followed by the specific choices for each parameter.
Rationale and description of model	16	If modelling is used, describe in detail and why used. Report if the model is publicly available and where it can be accessed.	The decision analytic model is illustrated in detail in Figure D and in full in Figure E, but the components models feeding into the decision analytic model are described as follows: the dynamic transmission (SEIRS) model is described briefly in the third methods section and Supplementary Methods, Section S1.1 and pictured in Figure B, and the treatment model is described briefly in Supplementary Methods, Section S1.9 and shown in Figure F.

(continued)

Section/item	Item No	Recommendation	Reported on page no, line no
Analytics & assumptions	17	Describe any methods for analysing or statistically transforming data, any extrapolation methods, and approaches for validating any model used.	The transmission and treatment models are discussed in the Supplementary Methods, Sections S1.1-S1.6. The treatment model is described in detail in Sections S1.9. Assumptions are described in detail in Supplementary Methods, Section S1.10 and the parameter glossary in Supplementary Note 4.
Characterising heterogeneity	18	Describe any methods used for estimating how the results of the study vary for subgroups.	Heterogeneity across health zones and across coordinations was characterised by aggregation and disaggregation, showing how different geographic portions of the country may need different resources and investments to reach disease control and elimination goals.
Characterising distributional effects	19	Describe how impacts are distributed across different individuals or adjustments made to reflect priority populations.	Because there were no subgroups, there were no distributional effects. The differential impacts of treatment on poorer or less poor individuals were beyond the scope of this paper.
Characterising uncertainty	20	Describe methods to characterise any sources of uncertainty in the analysis.	The epidemiological parameters are the posterior distributions of a model fitted to time-series data, and full details are available in the Supplementary Methods, Sections S1.5-S1.6. For the parameters to model health outcomes and costs, assumptions and estimates were parameterized according to conventions in the economic evaluation literature [3], taking care to sample from large distributions for aspects for which we knew very little. Epidemiological parameters are available on OSF `https://osf.io/ezjxb/`. Health outcome and cost-effectiveness parameters: see Table 1 and Supplementary Note 4.

(continued)

Section/item	Item No	Recommendation	Reported on page no, line no
Approach to engagement with patients and others affected by the study	21	Describe any approaches to engage patients or service recipients, the general public, communities, or stakeholders (such as clinicians or payers) in the others affected by the study.	Strategy components were determined along with the country director of PNLTHA, Dr. Erick Miaka and other PNLTHA members, Chancy Shampa, and Junior Lebuki (co-authors). Implementation of simulations of AS and PS costs was aided by information from Rian Snijders and Paul Verlé (co-authors), who has helped with operations in Bandundu Coordination and collaborators at the Liverpool School of Tropical Medicine, Andrew Hope, Iñaki Tirados, and Sophie Dunkley (co-authors) who have run vector control field operations in DRC since 2015.
**Results**			
Study parameters	22	Report the values, ranges, references, and, if used, probability distributions for all parameters. Report reasons or sources for distributions used to represent uncertainty where appropriate. Providing a table to show the input values is strongly recommended.	Table 1 and described in more detail in Supplementary Note 4.
Incremental costs and outcomes	23	For each intervention, report mean values for the main categories of estimated costs and outcomes of interest, as well as mean differences between the comparator groups. If applicable, report incremental cost-effectiveness ratios.	For four sample health zones, the intermediate outcomes are in Tables A-D; for the whole country, the outcomes can be found in Figure 4; for each coordination, the results are in F-M. For the whole country, the cost-effectiveness results are in Figures 2- 3 and the same results can be found per coordination in Figure E.
Effect of uncertainty	24	Model-based economic evaluation: Describe the effects on the results of uncertainty for all input parameters, and uncertainty related to the structure of the model and assumptions.	For four sample health zones, the effect of uncertainty on the cost-effectiveness are shown in Tables A-D. The interpretation is in the Supplementary Methods, Section S5.4. For all other health zones, the results of uncertainty on the CEA is shown in the GUI: GUI. The effect of uncertainty on the Elimination of Transmission goal is shown in Figure 3. The interpretation of the uncertainty is in the Results section.
Effect of engagement with patients and others affected by the study	25	Report on any difference patient/service recipient, the general public, community, or stakeholder involvement made to the approach or findings of the study	Our engagement with the stakeholders (co-authors) was iterative throughout the process
**Discussion**			

(continued)

Section/item	Item No	Recommendation	Reported on page no, line no
Study findings, limitations, generalisability, and current knowledge	26	Summarise key study findings and describe how they support the conclusions reached. Discuss limitations and the generalisability of the findings and how the findings fit with current knowledge.	Discussion.
**Other relevant information**			
Source of funding	27	Describe how the study was funded and the role of the funder in the identification, design, conduct, and reporting of the analysis. Describe other non-monetary sources of support.	Funding statement.
Conflicts of interest	28	Describe any potential for conflict of interest of study contributors in accordance with journal policy. In the absence of a journal policy, we recommend authors comply with the International Committee of Medical Journal Editors recommendations.	Conflict of interest statement.
