# Supplementary material for: Cost-effectiveness of end-game strategies against sleeping sickness across the Democratic Republic of Congo
Source: PLoS Negl Trop Dis. 2026 Oct 22;20(10):e0014777. doi: 10.1371/journal.pntd.0014777 (PMC13719019; doi:10.1371/journal.pntd.0014777)
Supplement: Supplementary file 8 [file pntd.0014777.s009.pdf]

Dear Drs. Mireji, Kamhawi, Brindley and reviewers:

We are grateful for this opportunity to resubmit.

The authors thank the editor and two reviewers for their review and detailed comments on our manuscript. The feedback has indeed made the manuscript clearer. We have attempted to address all comments provided; a full list of our changes in response to the comments can be found in the table below.

Finally, we have made other minor edits which are visible on the track changes version of our manuscript and provided the latex file for the production system formatted using the PLOS template.

**Comments**	**Author Respond**
**Overall (from the editor)**	
The reviewers find this to be a well-conducted and policy-relevant study, with notable strengths including its nationwide scope, transparent methodology, informative presentation of results, and potential value for guiding gHAT elimination strategies.<br><br>Before the manuscript can be accepted, please address the following required revisions:	
>Submit a completed CHEERS 2022 checklist and ensure the manuscript complies with the reporting guideline.	The CHEERS checklist was included in the supplement, but we realize that it was buried in what is a lengthy document. We have now made it into its own separate supplement called S2 Text.
>Clarify the costing methods by stating the price year (2024 USD), currency conversion methods, exchange rates, and any inflation adjustments.	The costs were denominated in 2024 values, as noted in line 294 of the manuscript. We expanded the details:<br><br>Costs were inflated and converted to 2024 US dollars as detailed in Supplementary Note 3. Most costs are collected in USD or EUR, converted back to local costs on the year when they were collected--which varies for different items, inflated to 2024 values, and converted to USD in 2024 using the country exchange

	rates and inflation rate as per the World Bank. More details are available in the parameter glossary, in Supplementary Note 3.
>Improve consistency in the presentation of numerical values (e.g., Table 2), correct the bracket formatting, provide a reference for the 3% discount rate, and review the manuscript for citation order and formatting.	Thank you for noting this. The values were consistently rounded to three significant figures, rather than to a fixed number of decimal places. We have now added a note to the table to clarify this reporting convention:<br><br>*All values given to 3 significant figures and therefore, the number of decimal places varies according to the magnitude of the estimate.*
>Expand the discussion of the limitations associated with the Bas Uélé data and interpret these findings with appropriate caution.	This is a good point. We make the point that the deaths might be driven by Bas Uele (which would bear 17.1% of all deaths, as stated in lines 512-514. While this magnitude is certainly substantial for 2 out of 165 health zones, it is not a major driver of policy at the national level because we are targeting policy at the health zone level. The uncertainty in these two health zones is driving policy in these two health zones only, but even deploying the most ambitious strategies in these regions would constitute a small amount of the total costs of the country; the costs of the EoT-maximising strategies would be $4.62M and $2.56M for Ango and Doruma, respectively, which amounts to 3.88% of the $185M nationwide total to maximise EoT (see Figure 4 and Supplementary Tables 27 and 28).<br><br>We have now added this text to the limitations which already discussed the issue of data in Bas Uélé (lines 446-52):<br><br>*Thankfully, however, the uncertainty in this region only drives uncertainty in the policy recommendations for this region but make only a small difference at the national level in terms of*

	costs. While the deaths might be driven by Bas Uélé, which might bear 17.1\% of all deaths, even deploying the most ambitious strategies in these regions would constitute a small amount of the total costs of the country; the costs of the EoT-maximising strategies would be \$4.62M and \$2.56M for Ango and Doruma, respectively, which amounts to 3.88\% of the \$185M nationwide total to maximise EoT (see Figure 4 and Supplementary Tables 27 and 28).
One reviewer also suggested evaluating resource-constrained budget scenarios. This analysis is encouraged but is not essential for acceptance. If it is beyond the scope of the current study, please acknowledge this limitation and briefly discuss its potential implications.	This is an insightful suggestion. We have added text to the limitations about this (lines 497-505). This is work that we have been pursuing for over 18 months and for such numerous districts and 6-8 strategies requires sophisticated search methods that are beyond the scope of this paper.<br><br>*Traditional cost-effectiveness analysis, like that conducted here, focuses on the economic efficiency of each region being modelled or the minimum cost to meet the target rather than placing constraints on total country budgets or team capacity. As we highlighted in our results, there is currently a substantial gap in the status quo costs and expected costs of strategies that would have a high probability of meeting elimination as well as a need to expand activities such as vector control. Therefore, it would be valuable to also undertake other types of analyses to optimise the selection of strategies with fixed resource constraints. Such an analysis non-trivial additional computational demands compared to the work presented here; an exhaustive search of all possible budgets across the 165 health zones modelled would require 10^100 strategy combinations to search to find the optimal overall budget. Because such a search is computationally infeasible the exercise*

	requires creation and employment of state-of-the-art methods to explore the strategy space efficiently without enumerating the entire space. Ideally, such a future constrained resource analyses will provide gHAT strategy recommendations for a range of budget options and help prioritise available resources.
**Methods (both reviewers interspersed)**	
-Are the objectives of the study clearly articulated with a clear testable hypothesis stated?<br>-Is the study design appropriate to address the stated objectives?<br>-Is the population clearly described and appropriate for the hypothesis being tested?<br>-Is the sample size sufficient to ensure adequate power to address the hypothesis being tested?<br>-Were correct statistical analysis used to support conclusions?<br>-Are there concerns about ethical or regulatory requirements being met?<br><br>Reviewer #1: Yes	The authors thank the reviewer
Reviewer #2: -Are the objectives of the study clearly articulated with a clear testable hypothesis stated?<br>YES<br><br>-Is the study design appropriate to address the stated objectives?<br>YES<br><br>-Is the population clearly described and appropriate for the hypothesis being tested?<br>YES	The authors thank the reviewer

-Is the sample size sufficient to ensure adequate power to address the hypothesis being tested?<br>YES<br><br>-Were correct statistical analysis used to support conclusions?<br>YES<br><br>-Are there concerns about ethical or regulatory requirements being met?<br>YES	
**Results**	
-Does the analysis presented match the analysis plan?<br>-Are the results clearly and completely presented?<br>-Are the figures (Tables, Images) of sufficient quality for clarity?Reviewer #1: Yes	The authors thank the reviewer
Reviewer #2: -Does the analysis presented match the analysis plan?<br>YES<br><br>-Are the results clearly and completely presented?<br>The results are generally well presented. However, the following consider the following:<br><br>From the methods the Bas Uélé region was considered as a special case with MSF screening from 2007–2014 after which there was no surveillance. This region also a very high percentage of predicted deaths (17.6%). The authors should be cautious in interpreting results from such unreliable data bearing the	This is a good point. We make the point that the deaths might be driven by Bas Uélé (which would bear 17.1% of all deaths, as stated in lines 339-41). While this magnitude is certainly substantial for 2 out of 165 health zones, it is not a major driver of policy at the national level because we are targeting policy at the health zone level. The uncertainty in these two health zones is driving policy in these two health zones only, but even deploying the most ambitious strategies in these regions would constitute a small amount of the total costs of the country; the costs of the EoT-maximising strategies would be \$4.62M and \$2.56M for Ango and Doruma, respectively, which amounts to 3.88% of the \$185M nationwide total to maximise EoT (see Figure 4 and Supplementary Tables 27 and 28).

potential magnitude of the results of this study in driving policy.<br><br>-Are the figures (Tables, Images) of sufficient quality for clarity?<br>YES	We have now added this text to the limitations which already discussed the issue of data in Bas Uélé (lines 446-52):<br><br>*Thankfully, however, the uncertainty in this region only drives uncertainty in the policy recommendations for this region but make only a small difference at the national level in terms of costs. While the deaths might be driven by Bas Uélé, which might bear 17.1% of all deaths, even deploying the most ambitious strategies in these regions would constitute a small amount of the total costs of the country; the costs of the EoT-maximising strategies would be $4.62M and $2.56M for Ango and Doruma, respectively, which amounts to 3.88% of the $185M nationwide total to maximise EoT (see Figure 4 and Supplementary Tables 27 and 28).*
**Conclusions**	
-Are the conclusions supported by the data presented?<br>-Are the limitations of analysis clearly described?<br>-Do the authors discuss how these data can be helpful to advance our understanding of the topic under study?<br>-Is public health relevance addressed?<br><br>Reviewer #1: Yes	The authors thank the reviewer
Reviewer #2: -Are the conclusions supported by the data presented?<br>YES<br><br>-Are the limitations of analysis clearly described?<br>YES, though additional limitations stated in the results could be	The authors thank the reviewer

considered by the authors<br><br>-Do the authors discuss how these data can be helpful to advance our understanding of the topic under study?<br>YES<br><br>-Is public health relevance addressed?<br>YES	
**Editorial and Data Presentation Modifications?**<br>Use this section for editorial suggestions as well as relatively minor modifications of existing data that would enhance clarity. If the only modifications needed are minor and/or editorial, you may wish to recommend "Minor Revision" or "Accept".	
Reviewer #1: (No Response)	
Reviewer #2: The authors have used willingness to pay (WTP) which is appreciated. How could modelling using a constrained budget look like based on the uncertainties of funding? How would working on a constrained budget affect EoT? The authors may consider this analysis if possible.	This is an insightful suggestion. We have added text to the limitations about this (lines 493-505). This is work that we have been pursuing for over 18 months and for such numerous districts and 6-8 strategies requires sophisticated search methods that are beyond the scope of this paper.<br><br>Traditional cost-effectiveness analysis, like that conducted here, focuses on the economic efficiency of each region being modelled or the minimum cost to meet the target rather than placing constraints on total country budgets or team capacity. As we highlighted in our results, there is currently a substantial gap in the status quo costs and expected costs of strategies that would have a high probability of meeting elimination as well as a need to expand activities such as vector control. Therefore, it would be valuable to also undertake other types of analyses to optimise the selection of strategies with fixed resource constraints. Such an analysis non-trivial additional

	computational demands compared to the work presented here; an exhaustive search of all possible budgets across the 165 health zones modelled would require 10^{100} strategy combinations to search to find the optimal overall budget. Because such a search is computationally infeasible the exercise requires creation and employment of state-of-the-art methods to explore the strategy space efficiently without enumerating the entire space. Ideally, such a future constrained resource analyses will provide gHAT strategy recommendations for a range of budget options and help prioritise available resources.
**Summary and General Comments**<br>Use this section to provide overall comments, discuss strengths/weaknesses of the study, novelty, significance, general execution and scholarship. You may also include additional comments for the author, including concerns about dual publication, research ethics, or publication ethics. If requesting major revision, please articulate the new experiments that are needed.	
**Reviewer 1**	
Reviewer #1: Thank you for inviting me to review this manuscript titled: “Cost-effectiveness of end-game strategies against sleeping sickness across the Democratic Republic of Congo”. Overall, it is clearly and thoughtfully structured, with a thorough methods section and a comprehensive supplementary appendix. The manuscript is quite lengthy, but this level of detail is a strength. It ensures transparency and reproducibility. Additionally, the maps in Figure 3 are very informative and help convey the findings clearly. Importantly, the interactive (R Shiny) app is an excellent way to present the analysis results.	The authors thank the reviewer
Please include a completed CHEERS 2022 checklist for the cost-effectiveness analysis. Additionally, ensure that both the abstract and the full manuscript meet all essential reporting requirements.	The CHEERS checklist was included in the supplement, but we realize that it was buried in what is a lengthy document. We have now made it into its own separate supplement called S2 Text.

On page 7, please specify the year to which all costs have been adjusted. Please clarify in the main text that all costs have been converted to 2024 USD, as noted in Supplement A.10. If any costs were originally collected in local currency, please specify the exchange rates used for conversion and describe any indexing or inflation adjustments applied.	The costs were denominated in 2024 values, as noted in line 294 of the manuscript. We expanded the details:<br><br>Costs were inflated and converted to 2024 US dollars as detailed in Supplementary Note 3, Section E.1. Most costs are collected in USD or EUR, converted back to local costs on the year when they were collected--which varies for different items, inflated to 2024 values, and converted to USD in 2024 using the country exchange rates and inflation rate as per the World Bank. More details are available in the parameter glossary, in Supplement Note 3.
Minor comments:<br>For Table 2, apply for all numerical entries (e.g., two or three decimals) consistently across the table. Also, please correct bracket style for "0.63 [0.54–0.72)"	Thank you for noting this. The values were consistently rounded to three significant figures, rather than to a fixed number of decimal places. We have now added a note to the table to clarify this reporting convention:<br><br>*All values given to 3 significant figures and therefore, the number of decimal places varies according to the magnitude of the estimate.*
On page 7, please reference the 3% discounting rate	Thanks for the reminder. It is now done (line 185).
**Reviewer 2**	
The substantial expansion of the study by authors building on their earlier work from 5 to 165 health zones gives a nationwide analysis and is a great strength of this study. It is appreciated that the analysis covers areas where 94% of the cases are reported and thus gives a country-wide picture. Further it predicts how sensible utilisation of resources would support of gHAT elimination of transmission by 2030	The authors thank the reviewer

The study would however benefit by improving on the referencing. The references be listed chronologically. The authors start with reference [19] in page 2 line 094. A total review of the references should be done	Thanks for pointing that out. That has now been changed. That was a latex compilation issue.
