# Supplementary material for: Cost-effectiveness of end-game strategies against sleeping sickness across the Democratic Republic of Congo
Source: PLoS Negl Trop Dis. 2026 Oct 22;20(10):e0014777. doi: 10.1371/journal.pntd.0014777 (PMC13719019; doi:10.1371/journal.pntd.0014777)
Supplement: Peer review history

# Peer review: Decision Letter 0

**DOI:** 10.1371/journal.pntd.0014777.r001

**Authors:** Paul Mireji, Robert Opiro

9 Jul 2026

Response to Reviewers Revised Manuscript with Track Changes Manuscript

Guest Editor

Shaden Kamhawi

co-Editor-in-Chief

PLOS Neglected Tropical Diseases

orcid.org/0000-0003-4304-636XX

Paul Brindley

co-Editor-in-Chief

PLOS Neglected Tropical Diseases

orcid.org/0000-0003-1765-0002

**Additional Editor Comments (if provided):**

Before the manuscript can be accepted, please address the following required revisions:

>Submit a completed CHEERS 2022 checklist and ensure the manuscript complies with the reporting guideline.

>Clarify the costing methods by stating the price year (2024 USD), currency conversion methods, exchange rates, and any inflation adjustments.

>Improve consistency in the presentation of numerical values (e.g., Table 2), correct the bracket formatting, provide a reference for the 3% discount rate, and review the manuscript for citation order and formatting.

>Expand the discussion of the limitations associated with the Bas Uélé data and interpret these findings with appropriate caution.

**Journal Requirements:**

1) Please ensure that the CRediT author contributions listed for every co-author are completed accurately and in full.

At this stage, the following Authors/Authors require contributions: Marina Antillón. Please ensure that the full contributions of each author are acknowledged in the "Add/Edit/Remove Authors" section of our submission form.

The list of CRediT author contributions may be found here: https://journals.plos.org/plosntds/s/authorship#loc-author-contributions

2) We ask that a manuscript source file is provided at Revision. Please upload your manuscript file as a .doc, .docx, .rtf or .tex. If you are providing a .tex file, please upload it under the item type u2018LaTeX Source Fileu2019 and leave your .pdf version as the item type u2018Manuscriptu2019.

3) Please provide an Author Summary. This should appear in your manuscript between the Abstract (if applicable) and the Introduction, and should be 150-200 words long. The aim should be to make your findings accessible to a wide audience that includes both scientists and non-scientists. Sample summaries can be found on our website under Submission Guidelines:

https://journals.plos.org/plosntds/s/submission-guidelines#loc-parts-of-a-submission

4) Please upload all main figures as separate Figure files in .tif or .eps format. For more information about how to convert and format your figure files please see our guidelines:

https://journals.plos.org/plosntds/s/figures

5) Please upload a copy of Figures FIG 1 A-C, 2, 3, and 4 which you refer to in your text on pages 3, 5, 11, and 12. Or, if the figure is no longer to be included as part of the submission please remove all reference to it within the text.

6) We have noticed that you have uploaded Supporting Information files, but you have not included a list of legends. Please add a full list of legends for your Supporting Information files after the references list.

7)We have amended your Competing Interest statement to comply with journal style. We kindly ask that you double check the statement and let us know if anything is incorrect.

**Reviewers' comments:**

**Key Review Criteria Required for Acceptance?**

As you describe the new analyses required for acceptance, please consider the following:

**Methods**

-Are the objectives of the study clearly articulated with a clear testable hypothesis stated?

-Is the study design appropriate to address the stated objectives?

-Is the population clearly described and appropriate for the hypothesis being tested?

-Is the sample size sufficient to ensure adequate power to address the hypothesis being tested?

-Were correct statistical analysis used to support conclusions?

-Are there concerns about ethical or regulatory requirements being met?

Reviewer #1: Yes

Reviewer #2: -Are the objectives of the study clearly articulated with a clear testable hypothesis stated?

YES

-Is the study design appropriate to address the stated objectives?

YES

-Is the population clearly described and appropriate for the hypothesis being tested?

YES

-Is the sample size sufficient to ensure adequate power to address the hypothesis being tested?

YES

-Were correct statistical analysis used to support conclusions?

YES

-Are there concerns about ethical or regulatory requirements being met?

YES

**********

**Results**

-Does the analysis presented match the analysis plan?

-Are the results clearly and completely presented?

-Are the figures (Tables, Images) of sufficient quality for clarity?

Reviewer #1: Yes

Reviewer #2: -Does the analysis presented match the analysis plan?

YES

-Are the results clearly and completely presented?

The results are generally well presented. However, the following consider the following:

From the methods the Bas Uélé region was considered as a special case with MSF screening from 2007–2014 after which there was no surveillance. This region also a very high percentage of predicted deaths (17.6%). The authors should be cautious in interpreting results from such unreliable data bearing the potential magnitude of the results of this study in driving policy.

-Are the figures (Tables, Images) of sufficient quality for clarity?

YES

**********

**Conclusions**

-Are the conclusions supported by the data presented?

-Are the limitations of analysis clearly described?

-Do the authors discuss how these data can be helpful to advance our understanding of the topic under study?

-Is public health relevance addressed?

Reviewer #1: Yes

Reviewer #2: -Are the conclusions supported by the data presented?

YES

-Are the limitations of analysis clearly described?

YES, though additional limitations stated in the results could be considered by the authors

-Do the authors discuss how these data can be helpful to advance our understanding of the topic under study?

YES

-Is public health relevance addressed?

YES

**********

**Editorial and Data Presentation Modifications?**

Use this section for editorial suggestions as well as relatively minor modifications of existing data that would enhance clarity. If the only modifications needed are minor and/or editorial, you may wish to recommend “Minor Revision” or “Accept”.

Reviewer #1: (No Response)

Reviewer #2: The authors have used willingness to pay (WTP) which is appreciated. How could modelling using a constrained budget look like based on the uncertainties of funding? How would working on a constrained budget affect EoT? The authors may consider this analysis if possible.

**********

**Summary and General Comments**

Use this section to provide overall comments, discuss strengths/weaknesses of the study, novelty, significance, general execution and scholarship. You may also include additional comments for the author, including concerns about dual publication, research ethics, or publication ethics. If requesting major revision, please articulate the new experiments that are needed.

Reviewer #1: Thank you for inviting me to review this manuscript titled: “Cost-effectiveness of end-game strategies against sleeping sickness across the Democratic Republic of Congo”. Overall, it is clearly and thoughtfully structured, with a thorough methods section and a comprehensive supplementary appendix. The manuscript is quite lengthy, but this level of detail is a strength. It ensures transparency and reproducibility. Additionally, the maps in Figure 3 are very informative and help convey the findings clearly. Importantly, the interactive (R Shiny) app is an excellent way to present the analysis results.

General comments

Please include a completed CHEERS 2022 checklist for the cost‑effectiveness analysis. Additionally, ensure that both the abstract and the full manuscript meet all essential reporting requirements.

On page 7, please specify the year to which all costs have been adjusted. Please clarify in the main text that all costs have been converted to 2024 USD, as noted in Supplement A.10. If any costs were originally collected in local currency, please specify the exchange rates used for conversion and describe any indexing or inflation adjustments applied.

Minor comments:

For Table 2, apply for all numerical entries (e.g., two or three decimals) consistently across the table. Also, please correct bracket style for “0.63 [0.54–0.72)”

On page 7, please reference the 3% discounting rate

Reviewer #2: The substantial expansion of the study by authors building on their earlier work from 5 to 165 health zones gives a nationwide analysis and is a great strength of this study. It is appreciated that the analysis covers areas where 94% of the cases are reported and thus gives a country-wide picture. Further it predicts how sensible utilisation of resources would

support of gHAT elimination of transmission by 2030

The study would however benefit by improving on the referencing. The references be listed chronologically. The authors start with reference [19] in page 2 line 094. A total review of the references should be done

**********

PLOS authors have the option to publish the peer review history of their article (what does this mean?). If published, this will include your full peer review and any attached files.

If you choose “no”, your identity will remain anonymous but your review may still be made public.

**Do you want your identity to be public for this peer review?**  For information about this choice, including consent withdrawal, please see our Privacy Policy.

Reviewer #1: No

Reviewer #2: **Yes:**  Erick Serem

**Figure resubmission:**

**Reproducibility:**

To enhance the reproducibility of your results, we recommend that authors of applicable studies deposit laboratory protocols in protocols.io, where a protocol can be assigned its own identifier (DOI) such that it can be cited independently in the future. Additionally, PLOS ONE offers an option to publish peer-reviewed clinical study protocols. Read more information on sharing protocols at https://plos.org/protocols?utm_medium=editorial-email&utm_source=authorletters&utm_campaign=protocols

# Author comment: Author response to Decision Letter 1

**DOI:** 10.1371/journal.pntd.0014777.r002

24 Aug 2026

Attachment Submitted filename: Whole DRC CEA Reviewer Feedback.pdf

# Peer review: Decision Letter 1

**DOI:** 10.1371/journal.pntd.0014777.r003

**Authors:** Paul Mireji, Robert Opiro

22 Sep 2026

Dear Dr Marina,

We are pleased to inform you that your manuscript 'Cost-effectiveness of end-game strategies against sleeping sickness across the Democratic Republic of Congo' has been provisionally accepted for publication in PLOS Neglected Tropical Diseases.

Before your manuscript can be formally accepted you will need to complete some formatting changes, which you will receive in a follow up email. A member of our team will be in touch with a set of requests.

Please note that your manuscript will not be scheduled for publication until you have made the required changes, so a swift response is appreciated.

IMPORTANT: The editorial review process is now complete. PLOS will only permit corrections to spelling, formatting or significant scientific errors from this point onwards. Requests for major changes, or any which affect the scientific understanding of your work, will cause delays to the publication date of your manuscript.

Should you, your institution's press office or the journal office choose to press release your paper, you will automatically be opted out of early publication. We ask that you notify us now if you or your institution is planning to press release the article. All press must be co-ordinated with PLOS.

Thank you again for supporting Open Access publishing; we are looking forward to publishing your work in PLOS Neglected Tropical Diseases.

Best regards,

Robert Opiro

Guest Editor

PLOS Neglected Tropical Diseases

Paul Mireji

Section Editor

PLOS Neglected Tropical Diseases

Shaden Kamhawi

co-Editor-in-Chief

PLOS Neglected Tropical Diseases

orcid.org/0000-0003-4304-636XX

Paul Brindley

co-Editor-in-Chief

PLOS Neglected Tropical Diseases

orcid.org/0000-0003-1765-0002

***********************************************************

Reviewer's Responses to Questions

**Key Review Criteria Required for Acceptance?**

As you describe the new analyses required for acceptance, please consider the following:

**Methods**

-Are the objectives of the study clearly articulated with a clear testable hypothesis stated?

-Is the study design appropriate to address the stated objectives?

-Is the population clearly described and appropriate for the hypothesis being tested?

-Is the sample size sufficient to ensure adequate power to address the hypothesis being tested?

-Were correct statistical analysis used to support conclusions?

-Are there concerns about ethical or regulatory requirements being met?

Reviewer #1: Yes

Reviewer #2: -Are the objectives of the study clearly articulated with a clear testable hypothesis stated?

Yes

-Is the study design appropriate to address the stated objectives?

Yes

-Is the population clearly described and appropriate for the hypothesis being tested?

yes

-Is the sample size sufficient to ensure adequate power to address the hypothesis being tested?

yes

-Were correct statistical analysis used to support conclusions?

Yes

-Are there concerns about ethical or regulatory requirements being met?

Yes

**********

**Results**

-Does the analysis presented match the analysis plan?

-Are the results clearly and completely presented?

-Are the figures (Tables, Images) of sufficient quality for clarity?

Reviewer #1: Yes

Reviewer #2: -Does the analysis presented match the analysis plan?

yes

-Are the results clearly and completely presented?

Yes

-Are the figures (Tables, Images) of sufficient quality for clarity?

Yes

**********

**Conclusions**

-Are the conclusions supported by the data presented?

-Are the limitations of analysis clearly described?

-Do the authors discuss how these data can be helpful to advance our understanding of the topic under study?

-Is public health relevance addressed?

Reviewer #1: Yes

Reviewer #2: -Are the conclusions supported by the data presented?

Yes

-Are the limitations of analysis clearly described?

Yes

-Do the authors discuss how these data can be helpful to advance our understanding of the topic under study?

Yes

-Is public health relevance addressed?

Yes

**********

**Editorial and Data Presentation Modifications?**

Use this section for editorial suggestions as well as relatively minor modifications of existing data that would enhance clarity. If the only modifications needed are minor and/or editorial, you may wish to recommend “Minor Revision” or “Accept”.

Reviewer #1: Accept

Reviewer #2: The authors have addressed all comments satisfactorily

**********

**Summary and General Comments**

Use this section to provide overall comments, discuss strengths/weaknesses of the study, novelty, significance, general execution and scholarship. You may also include additional comments for the author, including concerns about dual publication, research ethics, or publication ethics. If requesting major revision, please articulate the new experiments that are needed.

Reviewer #1: (No Response)

Reviewer #2: Accept

**********

PLOS authors have the option to publish the peer review history of their article (what does this mean?). If published, this will include your full peer review and any attached files.

If you choose “no”, your identity will remain anonymous but your review may still be made public.

**Do you want your identity to be public for this peer review?**  For information about this choice, including consent withdrawal, please see our Privacy Policy.

Reviewer #1: No

Reviewer #2: **Yes:**  Erick Serem

# Editor report: Acceptance letter

**DOI:** 10.1371/journal.pntd.0014777.r004

**Authors:** Paul Mireji, Robert Opiro

Dear Dr. Antillón,

We are delighted to inform you that your manuscript, "Cost-effectiveness of end-game strategies against sleeping sickness across the Democratic Republic of Congo," has been formally accepted for publication in PLOS Neglected Tropical Diseases.

We have now passed your article onto the PLOS Production Department who will complete the rest of the publication process. All authors will receive a confirmation email upon publication.

The corresponding author will soon be receiving a typeset proof for review, to ensure errors have not been introduced during production. Please review the PDF proof of your manuscript carefully, as this is the last chance to correct any scientific or type-setting errors. Please note that major changes, or those which affect the scientific understanding of the work, will likely cause delays to the publication date of your manuscript. Note: Proofs for Front Matter articles (Editorial, Viewpoint, Symposium, Review, etc...) are generated on a different schedule and may not be made available as quickly.

Soon after your final files are uploaded, the early version of your manuscript will be published online unless you opted out of this process. The date of the early version will be your article's publication date. The final article will be published to the same URL, and all versions of the paper will be accessible to readers.

For Research Articles, you will receive an invoice from PLOS for your publication fee after your manuscript has reached the completed accept phase. If you receive an email requesting payment before acceptance or for any other service, this may be a phishing scheme. Learn how to identify phishing emails and protect your accounts at https://explore.plos.org/phishing.

Thank you again for supporting open-access publishing; we are looking forward to publishing your work in PLOS Neglected Tropical Diseases.

Best regards,

Shaden Kamhawi

co-Editor-in-Chief

PLOS Neglected Tropical Diseases

Paul Brindley

co-Editor-in-Chief

PLOS Neglected Tropical Diseases
